# Exploring the potential mechanism of Xiaojin Pill therapy for benign prostatic hyperplasia through metabolomics and gut microbiota analysis

**DOI:** 10.3389/fmicb.2024.1431954

**Published:** 2024-08-21

**Authors:** Yuying Yang, Yunyun Quan, Yunteng Liu, Juhua Yang, Keyu Chen, Xiaozhou You, Hua Hua, Liangchun Yan, Junning Zhao, Jianbo Wang

**Affiliations:** ^1^School of Pharmacy, Southwest Medical University, Luzhou, China; ^2^Sichuan Institute for Translational Chinese Medicine, Sichuan Academy of Chinese Medicine Sciences, Key Laboratory of Biological Evaluation of Translational Chinese Medicine (TCM) Quality of National Administration of TCM, Sichuan Key Laboratory of Translational Medicine of TCM, Sichuan Authentic Medicine System Development Engineering Technology Research Center, Sichuan Authentic Medicine Formation Principle and Quality Evaluation Engineering Research Center, Chengdu, China; ^3^College of Food and Biological Engineering, Chengdu University, Chengdu, China; ^4^Pharmacology of Chinese Medicine, Shanxi University, Xianyang, China

**Keywords:** benign prostatic hyperplasia (BPH), Xiaojin Pill, metabolomics, gut microbiota, targets

## Abstract

**Background:**

Xiaojin Pill (XJP) is a traditional Chinese medicine prescribed for treating benign prostatic hyperplasia (BPH). It has been proven to have multiple effects, such as regulating sex hormone levels, exhibiting anti-tumor, anti-inflammatory, analgesic, and anti-platelet aggregation properties, and improving immunity. However, the material basis of XJP's therapeutic effect on BPH and its metabolic process *in vivo* remains to be clarified. At the same time, many microorganisms that exist in the urogenital tract, including those related to BPH, can also affect the health of the host.

**Methods:**

Using ultra-performance liquid chromatography-tandem mass spectrometry (UPLC-MS/MS), the chemical components of XJP were identified. A BPH model was created through bilateral testicular ablation and injections of testosterone propionate. A comprehensive evaluation of XJP efficacy was conducted using pathological ELISA, TUNEL, and immunohistochemical techniques. In addition, UPLC-MS metabolomics and 16S rRNA sequencing revealed the serum metabolic profile and intestinal microbiota composition. We performed a Spearman correlation coefficient analysis to highlight the interactions between “intestinal microbiota-serum factors” and “intestinal microbiota-metabolites.”

**Results:**

XJP contains 91 compounds that alleviate pathologies of BPH in rats, decreasing prostate weight, index, and serum levels of Dihydrotestosterone (DHT), Prostate-Specific Antigen (PSA), epidermal growth factor (EGF), basic fibroblast growth factor (bFGF), and vascular endothelial growth factor (VEGF) levels. It inhibits prostatic epithelial cell apoptosis and downregulates Bax, TGF-β1, and IGF-1 proteins in the caspase-3 pathway. Metabolomics studies have revealed 10 upregulated and 10 downregulated metabolites in treated rats, with 5-methylcytosine, uracil, and cytosine enriched in pyrimidine metabolism. L-arginine plays a pivotal role in metabolic pathways encompassing pyrimidine metabolism, arginine biosynthesis, and the mammalian target of rapamycin (mTOR) signaling pathway. 16S rRNA sequencing revealed that XJP optimized the diversity and balance of intestinal flora in BPH rats by decreasing the Bacteroidetes/Firmicutes (B/F) ratio, enhancing the beneficial bacteria, such as *Eggerthellaceae, Anaerovoracaceae*, and *Romboutsia*, and suppressing the dysfunctional bacteria, such as *Atopobiaceae, Prevotellaceae_NK3B31_group, Dorea*, and *Frisingicoccus*. According to the Spearman correlation coefficient analysis, *Lactobacillus* was found to be most associated with serum factors, whereas *Romboutsia* showed the highest correlation with metabolites. This finding suggests that XJP modulates pyrimidine metabolism disorders in BPH rats, a regulation that aligns closely with *Romboutsia, Prevotellaceae_NK3B31_group, Lactobacillus, Chujaibacter*, and *Enterorhabdus*, thereby providing valuable biological insights.

**Conclusion:**

In summary, these findings indicate that XJP possesses a synergistic anti-BHP effect through its multi-component, multi-target, multi-gut microbiota, and multi-metabolic pathway properties. The effect involves the regulation of sex hormone levels, growth factors, and the anti-epithelial cell apoptosis process. The modulation of specific gut microbiota by the host and the involvement of multiple metabolic pathways are likely one of the significant mechanisms of XJP in treating BPH. Notably, pyrimidine metabolism and the intestinal microbial ecosystem are closely intertwined in this process.

## 1 Introduction

Benign prostatic hyperplasia (BPH) is a prevalent chronic urinary disorder that predominantly affects middle-aged and elderly men, with its incidence escalating with age. According to the 2022 “Guide for Diagnosis, Treatment, and Health Management of Benign Prostatic Hyperplasia” published in China, BPH affects nearly 20% of men aged 51–60 years, increasing to 50% in the 61–70 age group and culminating in an astonishing 83% prevalence among those aged 81–90 years (Wang et al., 2022). Anatomically, BPH is characterized by the benign overgrowth of epithelial and stromal tissues within the prostate's transition zone, accompanied by the remodeling of adjacent epithelial and fibromuscular tissues along the urethra (Bauman et al., 2014; Ren et al., 2018). The underlying pathology revolves around the disruption of the intricate balance between cellular proliferation and apoptosis, a phenomenon attributed to a complex interplay of factors, including hormonal imbalances (androgen–estrogen ratio), stromal–epithelial crosstalk, overexpression of growth factors, and inflammatory infiltration (Claus et al., 1997; Wang Q. et al., 2023; Wang R. et al., 2023; Wang S. et al., 2023). The progression of BPH is gradual, transitioning from subtle tissue alterations to overt clinical manifestations. As the prostate enlarges, it compresses the urethra, inducing urinary tract obstruction and stimulating the bladder's detrusor muscle. This, in turn, results in a range of lower urinary tract symptoms (LUTS), encompassing urinary irritation (urgency, frequency, nocturia, reduced urine volume, and urge incontinence), obstruction (thin urine stream, interrupted urination, weak flow, tense urination, or prolonged urination), and post-micturition symptoms (incomplete emptying or post-void dribbling; Yu, 2022). If BPH remains untreated, it can progress to severe complications, such as urinary retention, bladder stones, and renal failure (Thorpe and Neal, 2003). Multiple factors such as metabolic syndrome, cardiovascular disease, diabetes, obesity, hypertension, diet, and depression are linked with the development of BPH (Kim et al., 2016; Zhang et al., 2022). Treatment options vary based on severity, encompassing conservative measures, medications, and surgery (Hollingsworth and Wilt, 2014). Common drugs include 5α-reductase inhibitors (e.g., finasteride and dutasteride) and α-adrenoceptor blockers (e.g., tamsulosin and silodosin; Gacci et al., 2014). BPH significantly impacts patients' physical and mental health as well as their quality of life, emphasizing the need for effective management strategies. China's aging population, which has a higher than average elderly ratio (9.3% aged 65+ years) (Ren, 2023), is projected to exceed 400 million by 2035, accounting for over 30% of the total population (CCTV News, 2022). This severe aging trend highlights how BPH could significantly burden healthcare workers and the national health system, owing to its prevalence among middle-aged and elderly men (Chughtai et al., 2016; Xia and Lin, 2023). Thus, the prevention and treatment of BPH have emerged as a pressing public health concern in China.

Xiaojin Pill (XJP), a renowned traditional Chinese medicine featuring in Wang Hongxu's Qing Dynasty text, targets yin abscess treatment. XJP alleviates nodules, reduces swelling, enhances blood flow, removes stagnation, and eases pain as it comprises 10 herbs, such as musk, *Momordica cochinchinensis*, and processed Aconiti kusnezoffii radix. It addresses early-stage abscesses, swollen/painful lumps, multiple abscesses, scrofula, goiter, and mammary gland issues. In modern medical practice, XJP is primarily used for mammary hyperplasia and has shown promise in addressing urinary disorders, such as BPH, non-bacterial prostatitis, and male urethritis syndrome (Su et al., 2022). Pharmacological studies revealed its anti-inflammatory, analgesic, anti-tumor, hormonal balancing, anti-platelet, and immune-modulatory properties (Xiong et al., 2018). A study by Qu (2008) reported that XJP and finasteride are equally effective in treating BPH; however, XJP surpasses finasteride with its fewer side effects such as sexual dysfunction and gastrointestinal discomfort, thereby making it a safer alternative. While the therapeutic effects of XJP on BPH are evident (Yang et al., 2023), its active components and mechanisms remain elusive.

**Figure 1:**
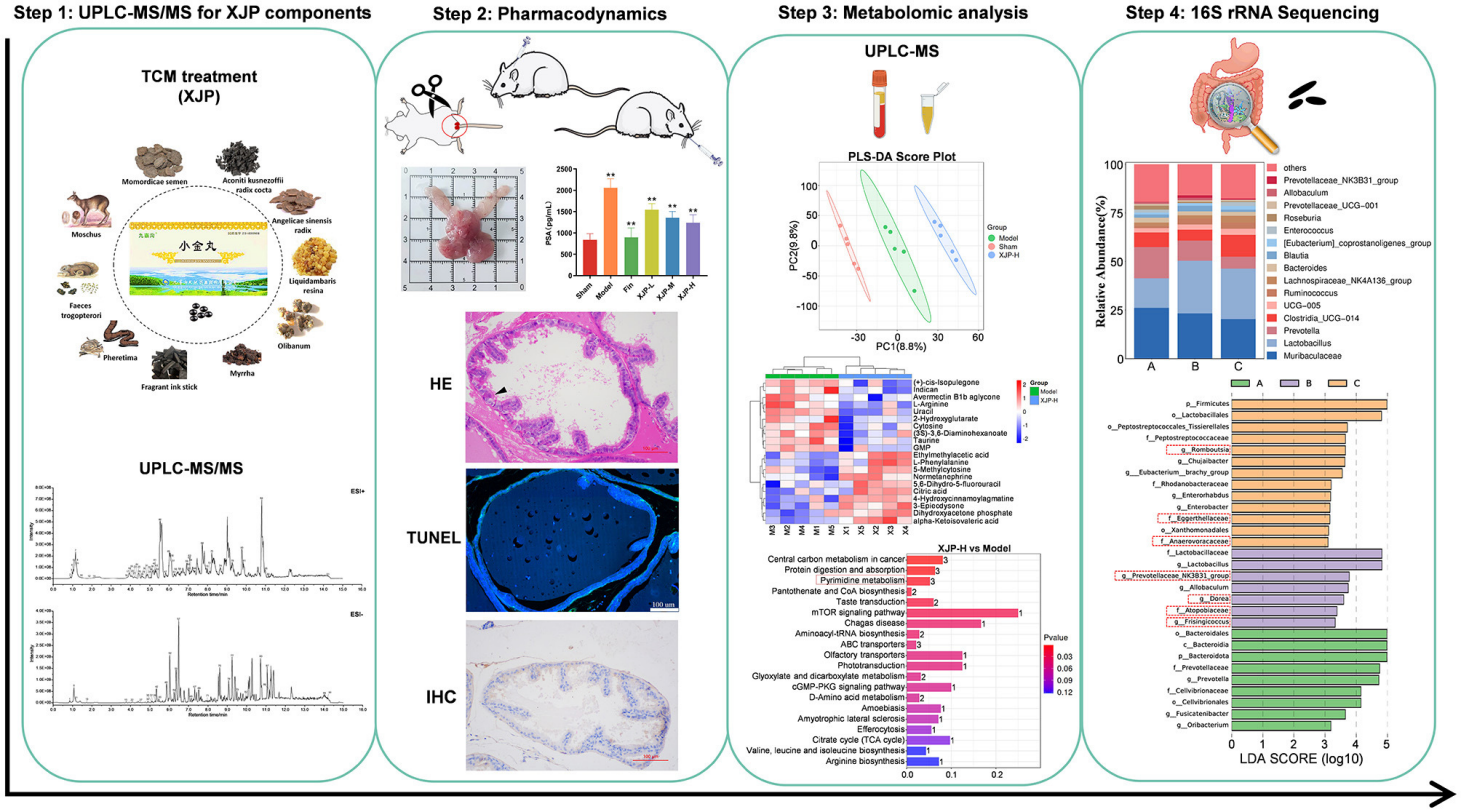
The scheme of investigating anti-BPH efficacy and the potential mechanism of XJP through metabolomics and gut microbiota analysis.

Current studies have shown that gut flora is involved in the metabolism of testosterone and androgens, which are related to prostate diseases (Yu et al., 2022; Bui et al., 2023). Hence, this study initially identified the constituent foundation of XJP via a component analysis. We then evaluated its therapeutic impact on BPH rats. We investigated the interplay between host metabolism and gut microbiota utilizing non-targeted metabolomics and 16S rRNA sequencing, which elucidated the mechanism of XJP in BPH treatment. This research offers a promising therapeutic candidate for BPH management (Figure 1).

## 2 Materials and methods

### 2.1 Regents and drugs

The materials used in this study included XJP supplied by Jiuzhaigou Natural Pharmaceutical Co., Ltd. (Sichuan, China), finasteride tablets sourced from Hangzhou MSD Pharmaceutical Co., Ltd. (Hangzhou, China), testosterone propionate (TP) injection sourced from Ningbo Second Hormone Factory (Ningbo, China), and soybean oil and carboxymethylcellulose sodium sourced from Shanghai-based companies (Macklin Biochemical & Yien Chemical Technology, respectively). ELISA kits for PSA, DHT, epidermal growth factor (EGF), basic fibroblast growth factor (bFGF), and vascular endothelial growth factor (VEGF) were procured from Jiangsu Jingmei Biotechnology (Jiangsu, China). A TUNEL kit was procured from Elabscience Biotechnology (Wuhan, China), while Bax and caspase-3 were procured from Cell Signaling Technology (Danvers, Massachusetts, USA). TGF-β1 and IGF-1 were sourced from ZEN-BIOSCIENCE (Chengdu, China) and Beijing Boaosen Biotechnology (Beijing, China), respectively.

### 2.2 UPLC-MS/MS for the qualitative detection of XJP components

A total of 50 mg of the sample were weighed, and 500 μL of ice-cold 80% methanol was added. The sample was ground with steel balls and kept in a freezer at −20°C for 30 min. It was then centrifuged at 20,000 g for 15 min, and 400 μL of the supernatant was collected. The collected supernatant was freeze-dried to remove the solvent. The sample was redissolved in 100 μL of ice-cold 50% methanol, centrifuged again, and filtered through a 0.22-μm membrane to obtain the test solution. The Thermo UltiMate 3000 UPLC system utilized an ACQUITY UPLC T3 column (100 x 2.1 mm, 1.8 μm) at 0.35 ml/min, 40°C, with an injection volume of 4 μl. Ultrapure water (5 mmol/L ammonium acetate + 5 mmol/L acetic acid) was used as A, and acetonitrile was used as B in a gradient elution (0–1 min: 1% B; 1.0–9.5 min: 1%−99% B; 9.5–11.5 min: 99% B; 11.5–12.0 min: 99%−1% B; and 12.0–15.0 min: 1% B). The Thermo Q-Exactive MS system was set up with sheath/auxiliary gas pressure at 0/10 psi, a capillary temperature of 350°C, ESI ±4.0/−4.5 kV, and in the DDA mode. MS1 (70–1,050 m/z, 70 k res., 100 ms IT) and MS2 (top 5 ions >100 k intensity, 17.5 k res., 50 ms IT, 6 s dynamic exclusion) were acquired. Xcalibur raw data were imported into MSDIAL for peak extraction, matching room temperature (RT) and mass with OrbitrapTCM (errors: 0.01 Da MS1 and 0.05 Da MS2). Compounds with Retention Score (RS) >70 were qualified, and active BPH-interfering components were predicted in the ETCM database.

### 2.3 Animals and treatments

A total of 54 male, specific pathogen-free, 8-week-old Sprague-Dawley rats (280 ± 20 g) from Beijing Huafukang Biotechnology Co., Ltd. [License No.: SCXK (Beijing) 2019-0008] were acclimatized for a week and then randomized into six groups (Xu et al., 2002): Sham (10 ml/kg 0.05% CMC-Na), Model (10 ml/kg 0.05% CMC-Na), Finasteride (0.52 mg/kg), and three XJP groups (low: 320 mg/kg, medium: 630 mg/kg, and high: 1,260 mg/kg) based on human–rat dose conversion. The Sham group underwent sham surgery, while the other groups underwent bilateral orchidectomy (ORC). Starting from day 8 after surgery, the non-Sham groups received a subcutaneous injection of TP (5 mg/kg), while the Sham group was administered soybean oil (1 mL/kg). Oral medications were administered daily for 4 weeks. The rats were housed in an Specific Pathogen Free (SPF) system with a controlled temperature (20–22°C), humidity (40–70%), and a 12-h light–dark cycle. These rats had *ad libitum* access to food and water.

### 2.4 Sample collection

Approximately 3 g of fresh rat feces were collected 12 h after the final administration and subsequently stored at −80°C for 16S rRNA sequencing. On the 2nd day, the rats were anesthetized with 2% pentobarbital sodium IP. After 30 min at room temperature, the serum obtained from the abdominal aorta was centrifuged (3,000 rpm, 10 min, 4°C) and stored at −80°C for ELISA and metabolomics analysis. The rats were euthanized by CO_2_ asphyxiation (KW-AL device, Nanjing Calvin Biotechnology). The prostate tissue was excised to calculate the prostate index (PI) [PI = prostate mass [mg]/body mass [g]] (Jin et al., 2019), and the ventral lobes were fixed in 4% paraformaldehyde for histopathological, TUNEL, and immunohistochemical assessments. All procedures were conducted in strict compliance with the guidelines of the Experimental Animal Ethics Committee of the Sichuan Provincial Hospital of Traditional Chinese Medicine.

### 2.5 Histopathology

Prostate tissues were processed through paraffin sectioning, dewaxing, hematoxylin-eosin (HE) staining, dehydration, and sealing. The images were captured using optical microscopy (Nikon SI, Japan) at 100 × magnification for lesion analysis. The thickness of the epithelial tissues in four glands at 200 × magnification was measured using Image J 6.0 (NIH and LOCI, USA) (de Abreu et al., 2018). The scoring adhered to the criteria outlined in Table 1 (Scolnik et al., 1994; Boehm et al., 2012; Bello et al., 2023).

**Table 1:** Pathology scoring criteria.

**Grade**	**Description**	**Score**
-	The glandular epithelial structure is normal, neatly arranged, and there is no hyperplasia, inflammation, necrosis, or congestion in the stroma.	0
+	There is glandular epithelial hyperplasia, dilated and congested stroma, and a slight inflammatory response.	1
++	There is glandular epithelial hyperplasia, dilated and congested stroma, dilated acini, and a mild inflammatory response in the acinar lumen and stroma.	2
+++	There is glandular epithelial hyperplasia, dilated and congested stroma, dilated acini, and a moderate inflammatory response in the acinar lumen and stroma.	3
++++	There is glandular epithelial hyperplasia, dilated and congested stroma, dilated acini, and a severe inflammatory response in the acinar lumen and stroma.	4

### 2.6 ELISA

The levels of serum PSA, DHT, EGF, bFGF, and VEGF in each group were measured using ELISA, following the manufacturer's instructions.

### 2.7 TUNEL

TUNEL staining was performed in accordance with the manufacturer's protocol to assess cell apoptosis. The images were captured using a Nikon Eclipse Ti-SR microscope and an Olympus VS200 slide scanner. The apoptosis rate was calculated at × 8 magnification using Image J 6.0.

### 2.8 Immunohistochemical assessment

A immunohistochemical assessment involved antigen retrieval, blocking endogenous peroxidase with 3% H2O2 and 3% BSA for 30 min. Primary antibodies (Caspase-3 1:500, Bax 1:500, TGF-β 1:100, and IGF-1 1:500) were incubated overnight at 4°C. Secondary antibodies (HRP-labeled) were added for 50 min at RT. DAB staining yielded brownish-yellow positives. Nuclei were counterstained, dehydrated, and mounted. The images were captured and analyzed using ImageJ to determine the positive area percentage.

### 2.9 Metabolomics analysis

A comprehensive description of the metabolomics method is provided in detail in Supplementary material 1.

### 2.10 16S rRNA sequencing

A comprehensive description of the gut microbiota method is provided in detail in Supplementary material 1.

### 2.11 Statistical analysis

Statistical analysis (IBM SPSS 27) included ANOVA for multi-group comparisons with LSD for significant pairs. The Kruskal–Wallis test was conducted to evaluate non-normal data, including pathological scores. Significance was set at a *p*-value of < 0.05/0.01. Data were presented as mean ± SD. Spearman's correlation analysis was used to analyze relationships between serum indicators, metabolites, and gut microbiota.

## 3 Results

### 3.1 Chemical components of XJP and its active ingredients for treating BPH

**Figure 2:**
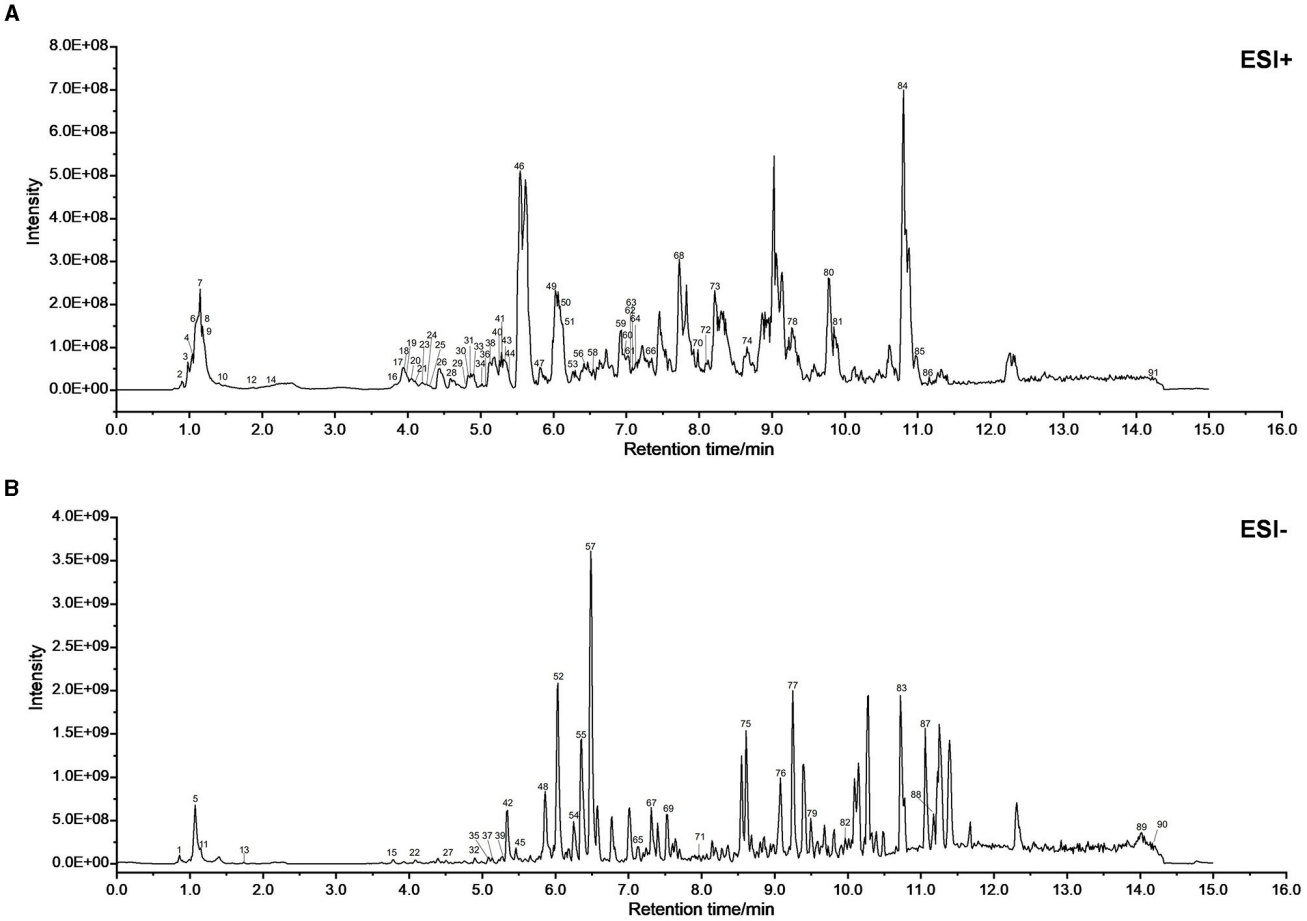
UPLC-MS/MS was used to identify the chemical composition of XJP. **(A)** BPH of XJP in the positive (POS) mode. **(B)** BPH of XJP in the negative (NEG) mode.

Figures 2A, B demonstrate BPH in XJP's positive (POS) and negative (NEG) ion modes. A total of 91 compounds were identified from XJP, including 26 alkaloids, 23 terpenes, nine phenolic acids, eight phenylpropanoids, three steroids, three phenols, two flavonoids, one quinone, and 16 other compounds. Detailed information is provided in Supplementary Table 1 and Supplementary material 2.

### 3.2 Evaluation of the therapeutic effect of XJP on BPH rats

#### 3.2.1 Surface observation and pathological changes

**Figure 3:**
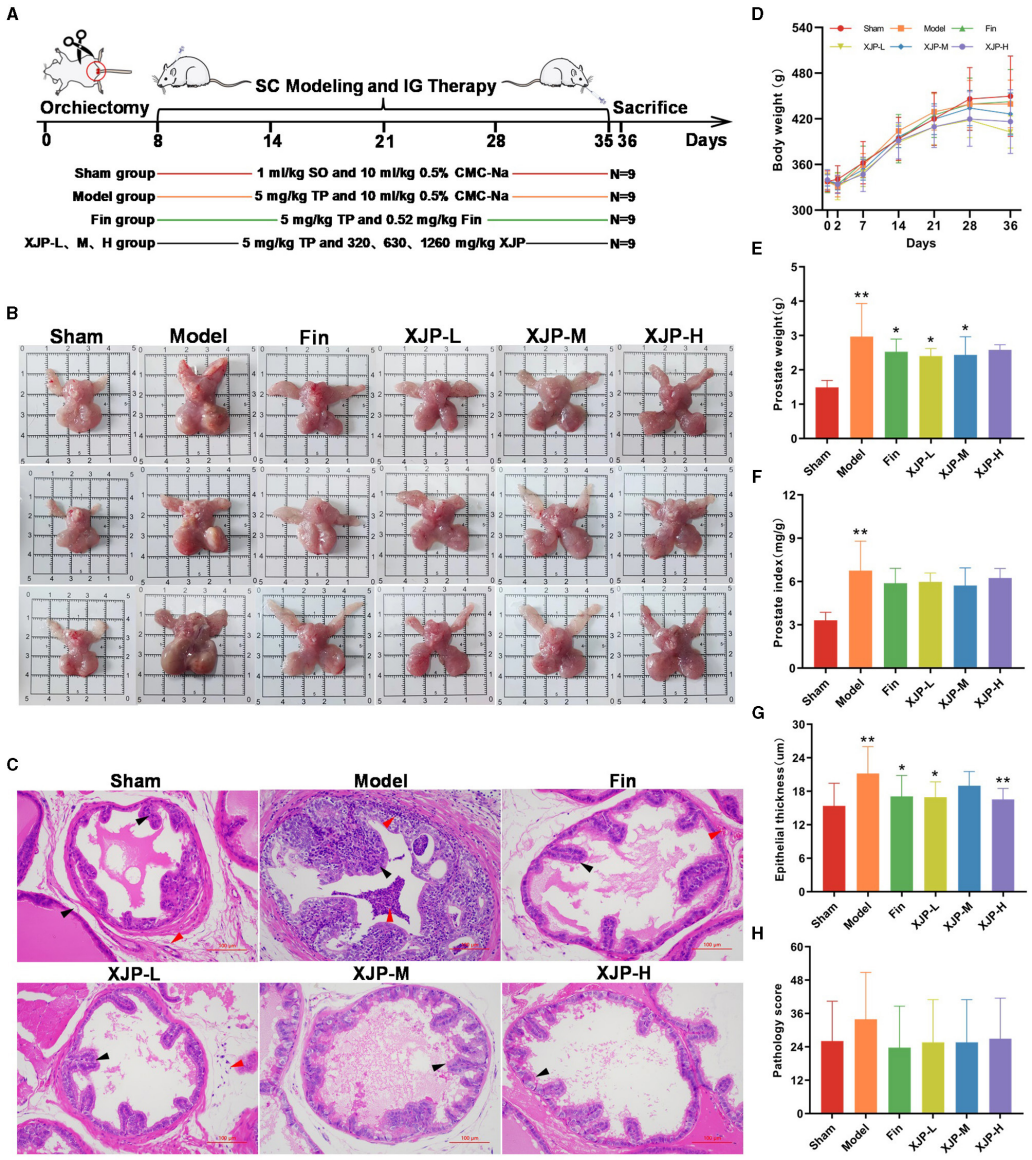
The impact of XJP on the apparent observation and pathological changes in BPH rats. **(A)** A timeline of the experimental design. **(B)** Prostate phenotypes in the different groups. Sham: pale red color, no nodules; Model: enlarged prostate, dark red color, with nodules; and Drug-treated groups: decreased prostate volume, grayish-red color, no nodules. **(C)** Representative images of pathological examination (scale bar: 100 um). Sham: epithelial cells arranged in a single layer of columnar or cuboidal shape (black arrows), no inflammatory reaction in the stroma (red arrows); Model: dilated acini, epithelial hyperplasia protruding into the lumen (black arrows), granulocyte infiltration in the epithelium, lumen, and surrounding stromal tissues (red arrows); Finasteride: epithelial cells arranged neatly, no hyperplasia (black arrows), and stromal vascular congestion (red arrows); XJP-L: epithelial cells arranged neatly, no hyperplasia (black arrows), mild stromal inflammatory reaction (red arrows); XJP-M: slight epithelial hyperplasia (black arrows); and XJP-H: epithelial cells arranged in a single layer of columnar or a cuboidal shape (black arrows). **(D)** A line chart of the body weight changes (*n* = 9, $\bar{x}$ ± SD). **(E)** Prostate weight (*n* = 8–9, mean ± SD). **(F)** Prostate index (*n* = 8–9, mean ± SD). **(G)** Epithelial thickness of the prostate tissue (*n* = 8–9, mean ± SD). **(H)** Pathological score (*n* = 8–9, mean ± SD). *p < 0.05, **p < 0.01.

Androgen depletion was induced in rats using ORC and combined with TP-induced BPH modeling (Huang et al., 2022). The experimental setup is illustrated in Figure 3A. On the second post-operative day (Figure 3D), body weights in all groups, except the Sham group, declined slightly, confirming the success of ORC. Subsequently, weights steadily increased throughout modeling and treatment. Figures 3B, C show the prostate phenotypes and pathologies, respectively. The Model group displayed glandular dilation, epithelial thickening/shedding, and inflammation. The drug-treated groups exhibited improved wound healing. As shown in Figures 3E, F, significant increases in prostate weight and PI were observed in the Model group compared to the Sham group (*p* < 0.01). The Finasteride, XJP-L, and XJP-M groups exhibited significantly reduced prostate weights (*p* < 0.05), while all drug groups showed a declining PI trend. Figure 3G demonstrates the effect of XJP on prostate epithelial thickness in BPH rats, highlighting the hyperplastic state. When compared with Sham rats, Model rats displayed a pronounced elevation in epithelial thickness (*p* < 0.01). However, therapeutic intervention in the Finasteride, XJP-L, and XJP-H groups led to a significant reduction in epithelial thickness compared to Model rats. The Finasteride and XJP-L groups achieved statistical significance with a *p*-value of <0.05 and the XJP-H group achieved a *p*-value of <0.01. Finally, as shown in Figure 3H, lower pathological scores were observed in all treatment groups except the Model group.

#### 3.2.2 Relevant serum factors and the apoptosis status of the prostate tissue

**Figure 4:**
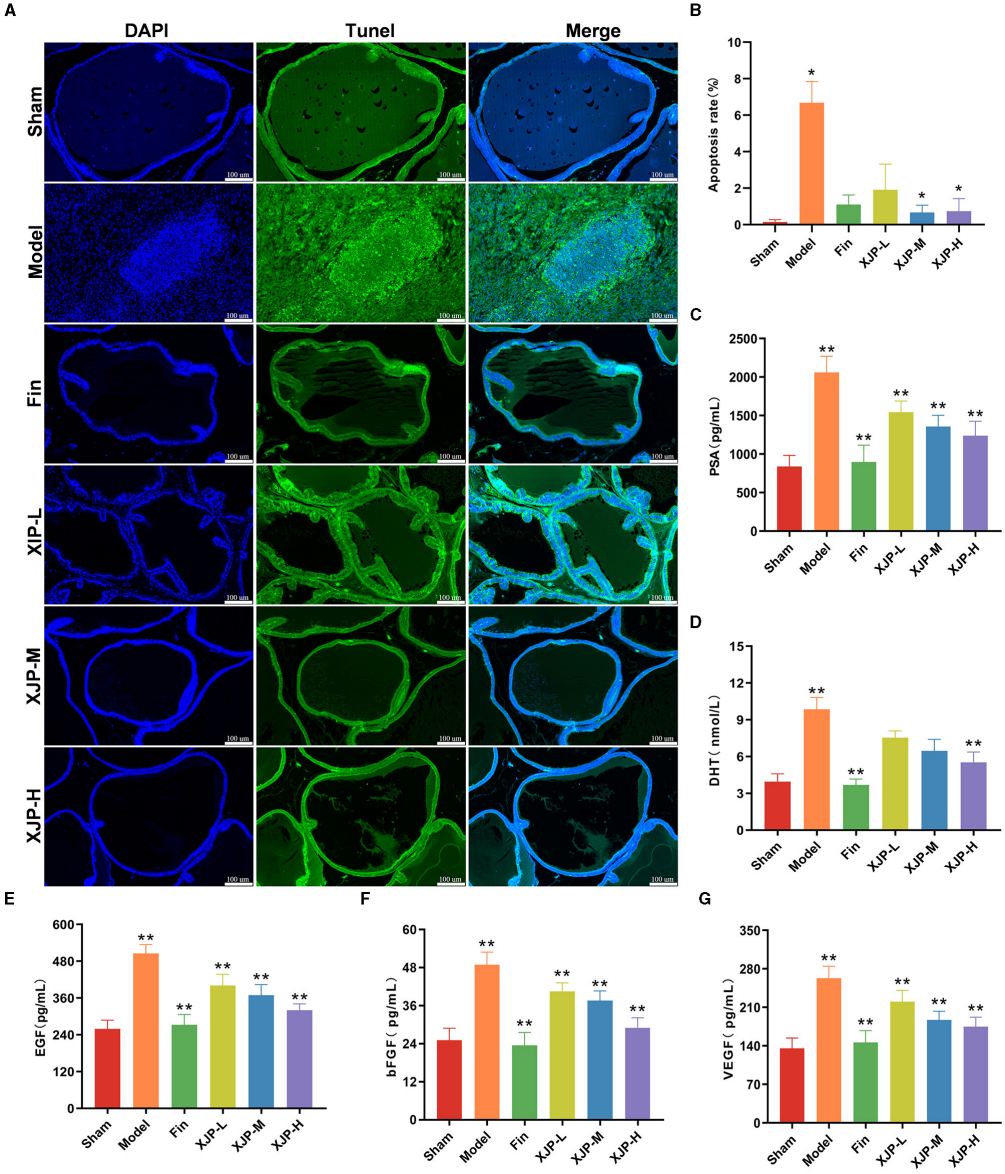
Effects of XJP on relevant serum factors and apoptosis of the prostate tissue in BPH rats. **(A)** Representative images of TUNEL staining (scale bar = 100 μm). DAPI (blue) displays nuclear staining, TUNEL (green) shows apoptotic cells, and the Merge image displays apoptotic cells and all cell nuclei simultaneously. **(B)** Apoptosis rate (*n* = 6, $\bar{x}$ ± SD). **(C)** PSA. **(D)** DHT. **(E)** EGF. **(F)** bFGF. **(G)** DHT (*n* = 8–9, $\bar{x}$ ± SD). *p < 0.05, **p < 0.01.

As shown in Figures 4A, B, a heightened apoptosis rate was observed in the Model group compared to the Sham group (*p* < 0.05), which was mitigated by XJP-M and XJP-H treatments (*p* < 0.05). Consequently, TUNEL staining confirmed the pro-apoptotic effect in BPH epithelial cells, which XJP was able to inhibit. As shown in Figures 4C–G, PSA, EGF, bFGF, and VEGF levels increased in the Model group but significantly reduced in all treatment groups (*p* < 0.01), with Finasteride and XJP-H groups exhibiting further lowering of DHT (*p* < 0.01), suggesting XJP's dose-dependent modulation of BPH-related serum factors.

#### 3.2.3 Expression of related proteins in the prostate tissue

**Figure 5:**
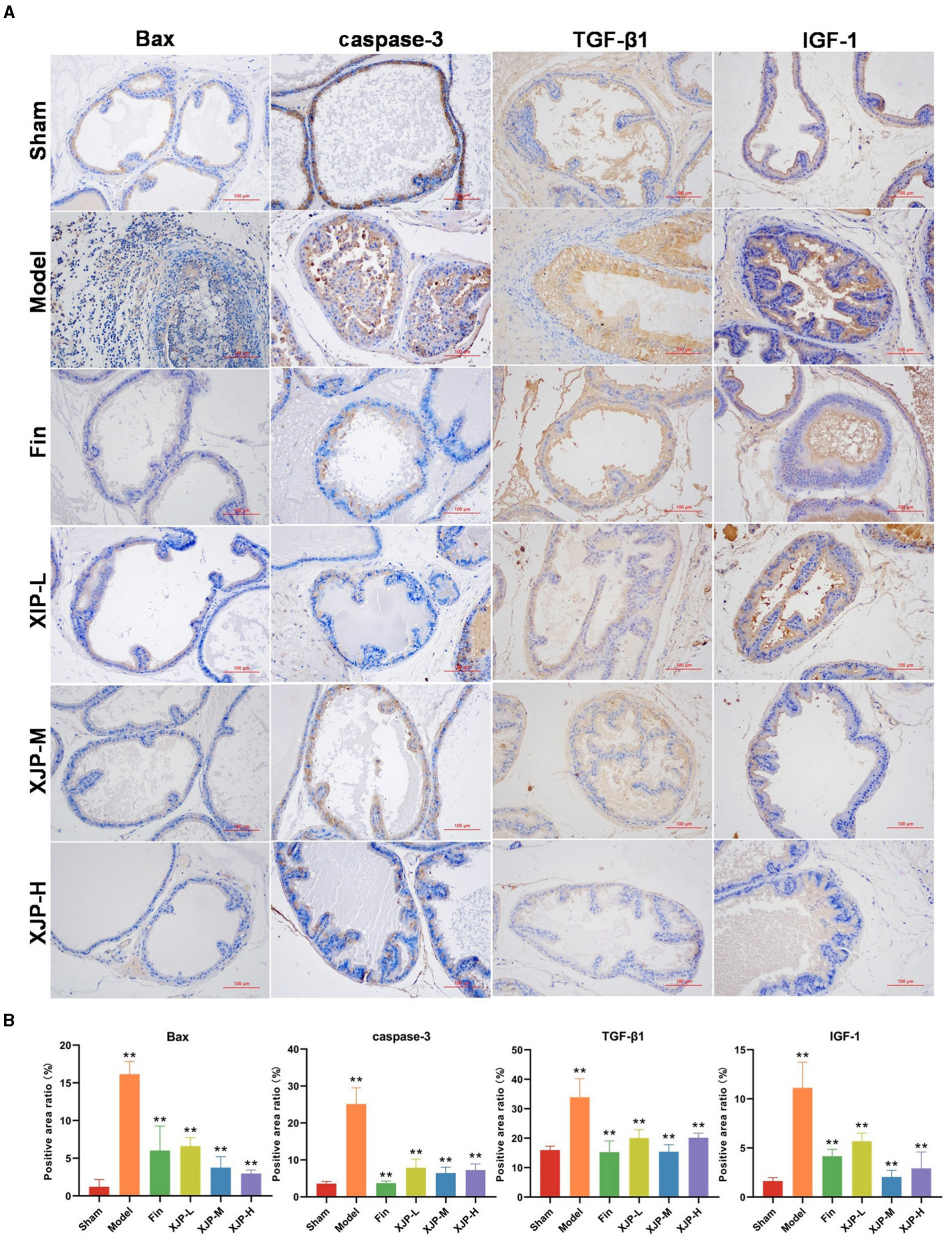
Effects of XJP on the expression of related proteins in the prostate tissue of BPH rats. **(A)** IHC staining of Bax, caspase-3, TGF-β1, and IGF1-1 (scale bar = 100 μm). **(B)** Positive area ratio (*n* = 3, $\bar{x}$ ± SD, **p < 0.01).

As shown in Figures 5A, B, the ICH results indicated that the expression of Bax, caspase-3, TGF-β1, and IGF-1 proteins in the prostate tissue of the Model group was significantly increased compared to the Sham group (*p* < 0.01). In contrast, the expression of these proteins was significantly reduced in the Finasteride, XJP-L, XJP-M, and XJP-H groups compared to the Model group (*p* < 0.01).

### 3.3 The effect of XJP on the level of serum metabolites in BPH rats

#### 3.3.1 Data quality control

**Figure 6:**
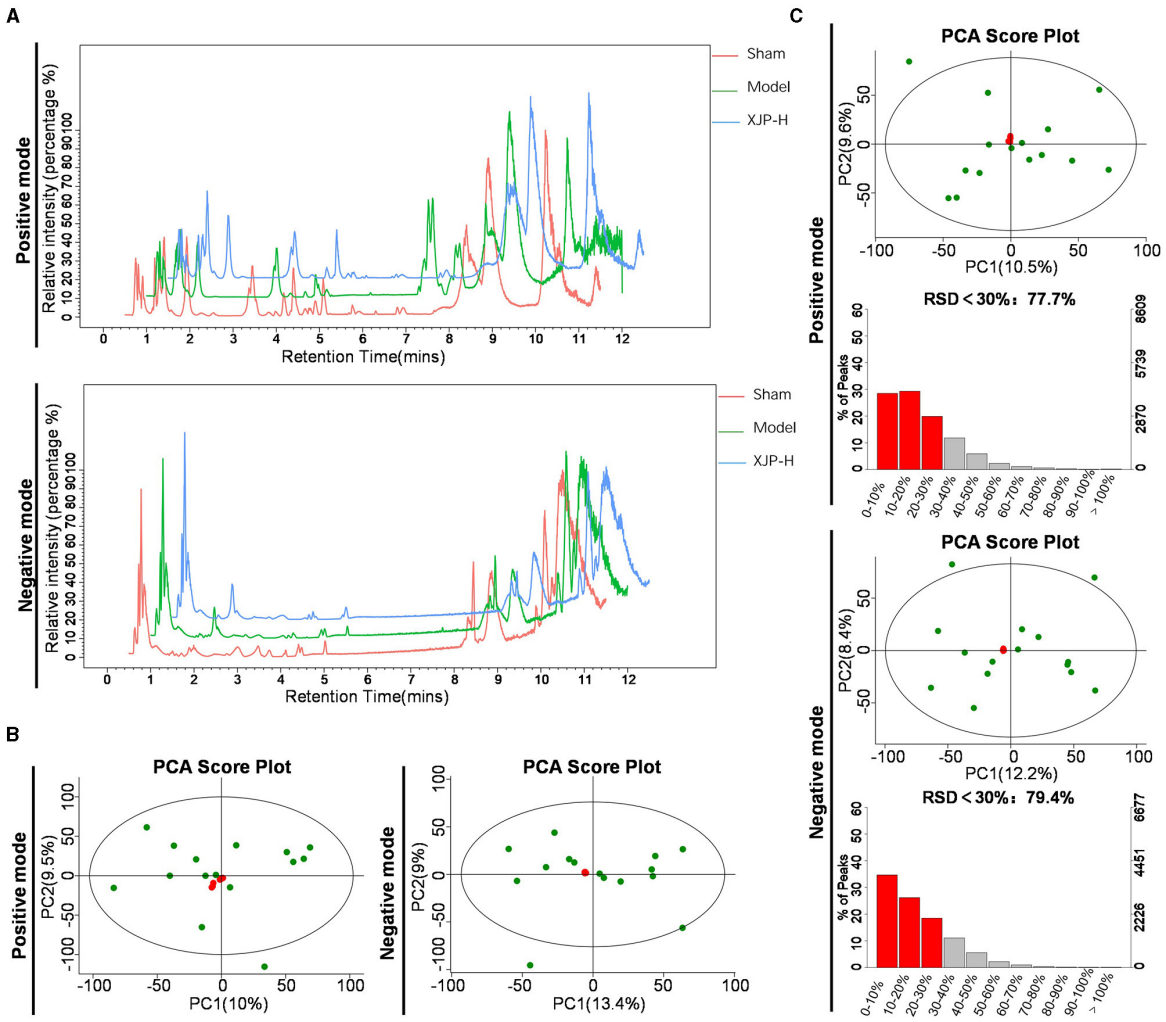
Data quality analysis. **(A)** Typical BPC charts of serum obtained from Sham, Model, and XJP-H rats in both positive (POS) and negative (NEG) modes. **(B)** PCA plots of the overall sample QC in POS and NEG modes, with PC1 accounting for 10% and PC2 for 9.5% in the POS mode and PC1 accounting for 13.4% and PC2 for 9% in the NEG mode. **(C)** QA results of the overall samples in POS and NEG modes, with PC1 accounting for 10.5% and PC2 accounting for 9.6% in the POS mode, with 77.7% of features having Relative Standard Deviation (RSD) <30%. In the NEG mode, PC1 accounted for 12.2% and PC2 accounted for 8.4%, with 79.4% of features having RSD <30%.

Figures 6A–C demonstrate robust reproducibility and system stability for metabolite profiling. As shown in Figure 6A, consistent serum Base Peak Chromatogram (BPC) peaked across POS/NEG modes for Sham, Model, and XJP-H rats. In Figure 6B, the principal component analysis (PCA) highlights tight QC clustering with minimal POS variance and substantial NEG overlap. As shown in QA data of Figure 6C, the PCA exhibits uniform patterns and high-quality features (RSD <30%), affirming system fitness for metabolic analysis.

#### 3.3.2 Analysis of differential metabolites

**Figure 7:**
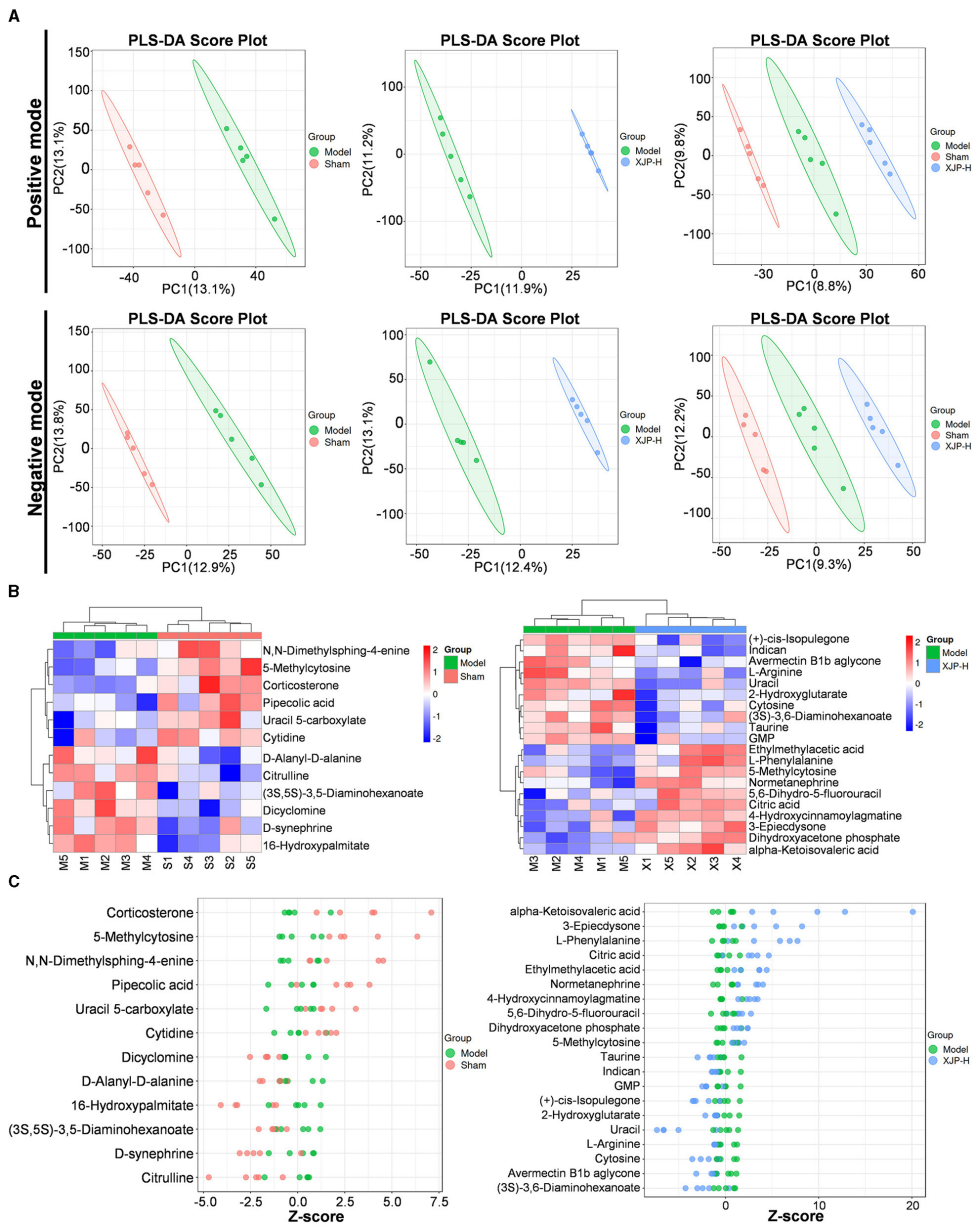
Metabolic characteristics of the rat serum in BPH. **(A)** A PLS-DA score plot comparing different groups under positive (POS) and negative (NEG) modes. **(B)** A cluster heatmap showing differential metabolites between the two groups. The horizontal axis represents samples, and the vertical axis represents metabolites. Redder colors indicate higher expression levels of metabolites, while bluer colors indicate lower expression levels. **(C)** A *z*-score plot of differential metabolites between the two groups. The horizontal axis represents the *z*-score, and the vertical axis represents metabolites.

The partial least squares-discriminant analysis (PLS-DA) analysis (Figure 7A) distinctly separated the profiles of the three sample groups in POS and NEG modes. Within each group, five samples were clustered tightly, exhibiting significant PC1–PC2 differences. Notably, the metabolic disparities between the Sham–Model groups and the XJP-H–Model groups were more evident, with wider 95% CI lateral distances (colored blocks), which emphasized richer metabolic variations. The heatmap (Figure 7B) visually depicts these metabolic differences. The *z*-score plot shown in Figure 7C further confirms the significant variations in metabolic components (v) between the Sham–Model and XJP-H–Model groups (*p* < 0.05; Sreekumar et al., 2009). The statistical analysis (Supplementary material 3) identified 12 differential metabolites (6 up and 6 down) in the Sham–Model and 20 differential metabolites (10 up and 10 down) in the XJP-H–Model groups. Identification diagrams are shown in Supplementary material 4. Notably, cytosine and L-phenylalanine were detected in XJP, which indicated potential entry into the bloodstream in their native forms.

#### 3.3.3 Analysis of differential pathways

**Figure 8:**
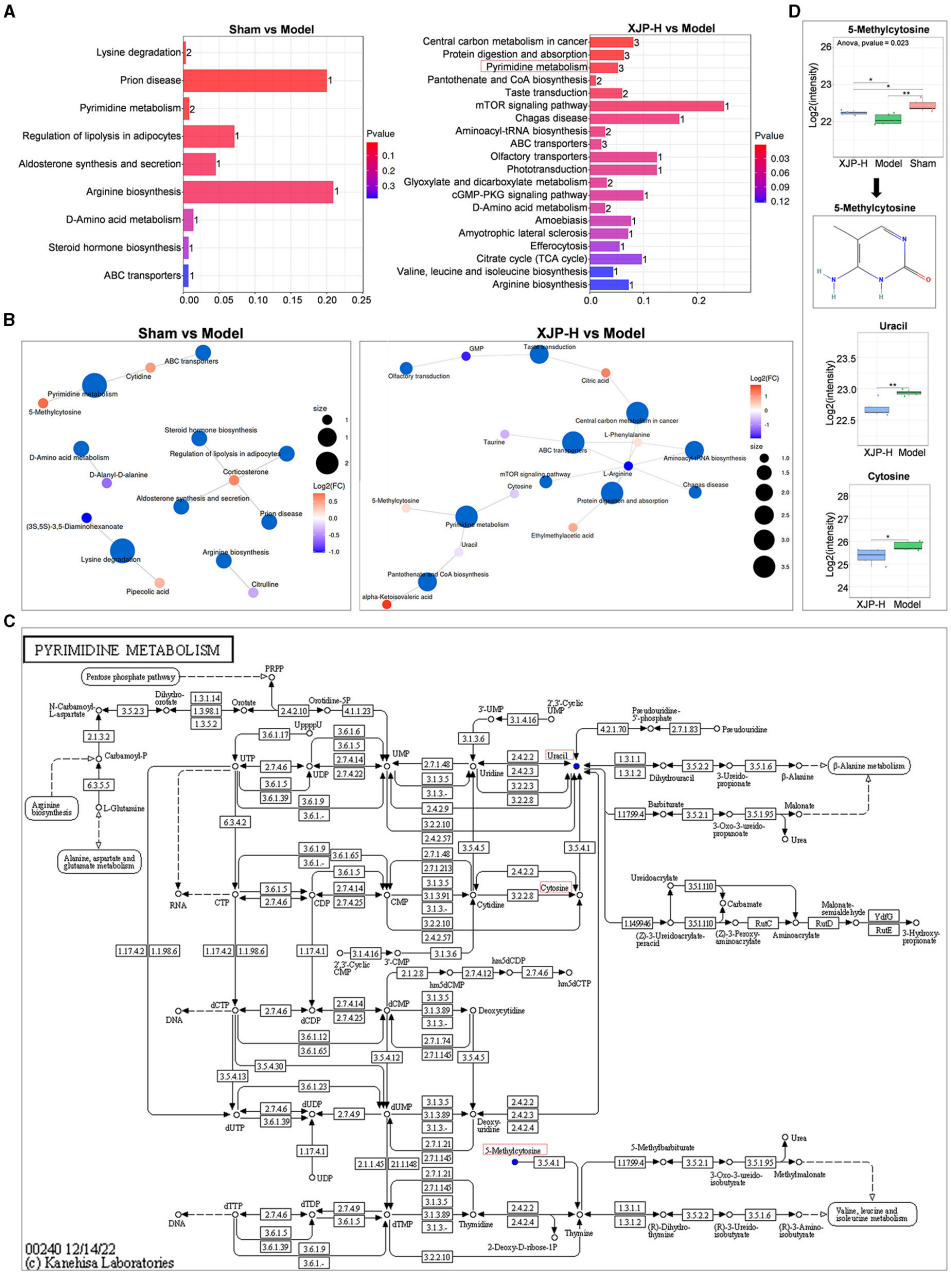
Impact of differential metabolites on metabolic pathways. **(A)** An enrichment map comparing two groups, with the horizontal axis indicating the enrichment factor (higher for greater enrichment) and the vertical axis showing sorted pathway names by *p*-values (redder for more significant enrichment). Bar chart numbers represent the number of enriched compounds per pathway. **(B)** Network visualization of metabolites and their associated metabolic pathways. **(C)** Pyrimidine metabolism pathway. **(D)** The boxplot of the pyrimidine metabolism pathway shows the quantitative results of 5-methylcytosine, uracil, and cytosine.

As shown in Figure 8A, comparisons between the Sham and Model groups, as well as between the XJP and Model groups, revealed common metabolic pathways, including pyrimidine, arginine, D-amino acid, and ABC transporter metabolism. Figure 8B highlights the prominence of 5-methylcytosine, uracil, and cytosine in pyrimidine metabolism, while L-arginine is found to be crucial in multiple pathways. Figure 8C provides details about pyrimidine metabolism. As shown in Figure 8D, further analysis revealed that 5-methylcytosine expression dropped post-modeling (*p* < 0.01) but rose with XJP treatment (*p* < 0.05), suggesting its role as a biomarker for XJP's BPH intervention. Contrarily, uracil and cytosine expressions decreased in the XJP-H group compared to the Model group.

### 3.4 The effect of XJP on the gut microbiota of BPH rats

#### 3.4.1 Data quality control and analysis of gut microbiota diversity and composition

**Figure 9:**
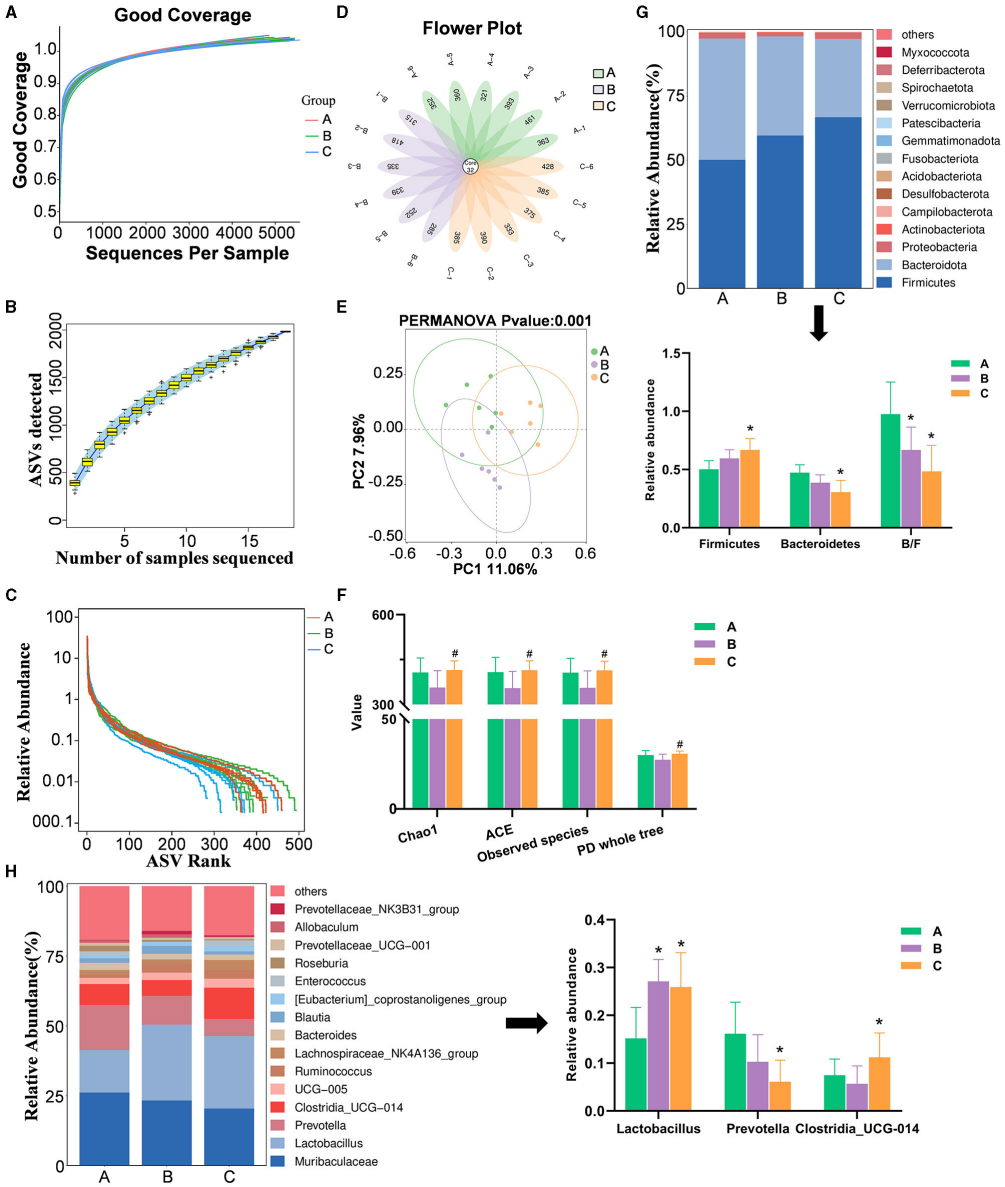
Data quality control, gut microbiota diversity, and composition analysis. **(A)** Sequence coverage curve. **(B)** *Specaccum* species accumulation curve, with the horizontal axis representing the number of samples and the vertical axis representing the number of ASVs. The blue shaded area indicates the confidence interval of the curve. **(C)** Relative abundance rank abundance curve. Each curve represents the ASVs of a sample sorted based on the number of sequences they contain. The vertical axis represents the relative abundance of each ASV, and the sequence number and relative abundance are connected by a broken line. **(D)** Petal plot based on amplicon sequence variants (ASVs). Each petal corresponds to a sample, with the overlapping area indicating the ASVs shared among all 18 samples. The number on a single petal represents the unique ASV count for that sample. **(E)** Impact on alpha diversity indices (*n* = 6, $\bar{x}$ ± SD, ^#^*p* < 0.05 vs. Model). **(F)** Principal component analysis (PCA) plot for beta diversity analysis. **(G)** Bar charts showing species composition at the phylum level for the three groups, along with a significant analysis of phylum-level abundances and B/F ratios (*n* = 6, $\bar{x}$ ± SD, *p < 0.05 vs. Sham). **(H)** Bar charts depicting species composition at the genus level for the three groups, with significant analysis of genus-level abundances (*n* = 6, $\bar{x}$ ± SD, *p < 0.05 vs. Sham). A, B, and C stand for Sham, Model, and XHP-H groups.

The sequencing depth across 18 samples was consistent, with counts ranging from 44,457 to 55,741 (Figure 9A). The amplicon sequence variant (ASV) cumulative curve plateaued at 1984 ASVs, indicating comprehensive species coverage (Figure 9B). The Sham group had the highest sequence count, followed by the XJP-H and Model groups, suggesting an even species distribution and richness (Figure 9C). A total of 132 shared and unique ASVs highlighted species diversity (Figure 9D), validating the adequacy of the data. As shown in Figure 9E, there were significant increases in microbial diversity indices in XJP-H compared to the Model group (*p* < 0.05), but no difference was observed when compared to the Sham group, indicating that XJP reverses BPH-induced microbiota decline. As shown in Figure 9F, significant Beta diversity differences were observed between the groups (*p* < 0.001), with slight variations (PC1: 11.6% and PC2: 7.96%) requiring further investigation. As shown in Figure 9G, significant changes in the microbiota composition at the phylum level were observed: Firmicutes increased, while Bacteroidota decreased in the Model and XJP-H groups, with the XJP-H group showing the most significant alterations (*p* < 0.05). Both the Model and XJP-H groups had lower B/F ratios than the Sham (*p* < 0.05) group. Figure 9H highlights the genus-level variations: *Lactobacillus* increased in both the Model and XJP-H groups, *Clostridia_UCG-014-014* increased only in the XJP-H group, and *Prevotella* decreased in the XJP-H group, thereby altering the microbial ecosystem.

#### 3.4.2 Differential bacterial species

**Figure 10:**
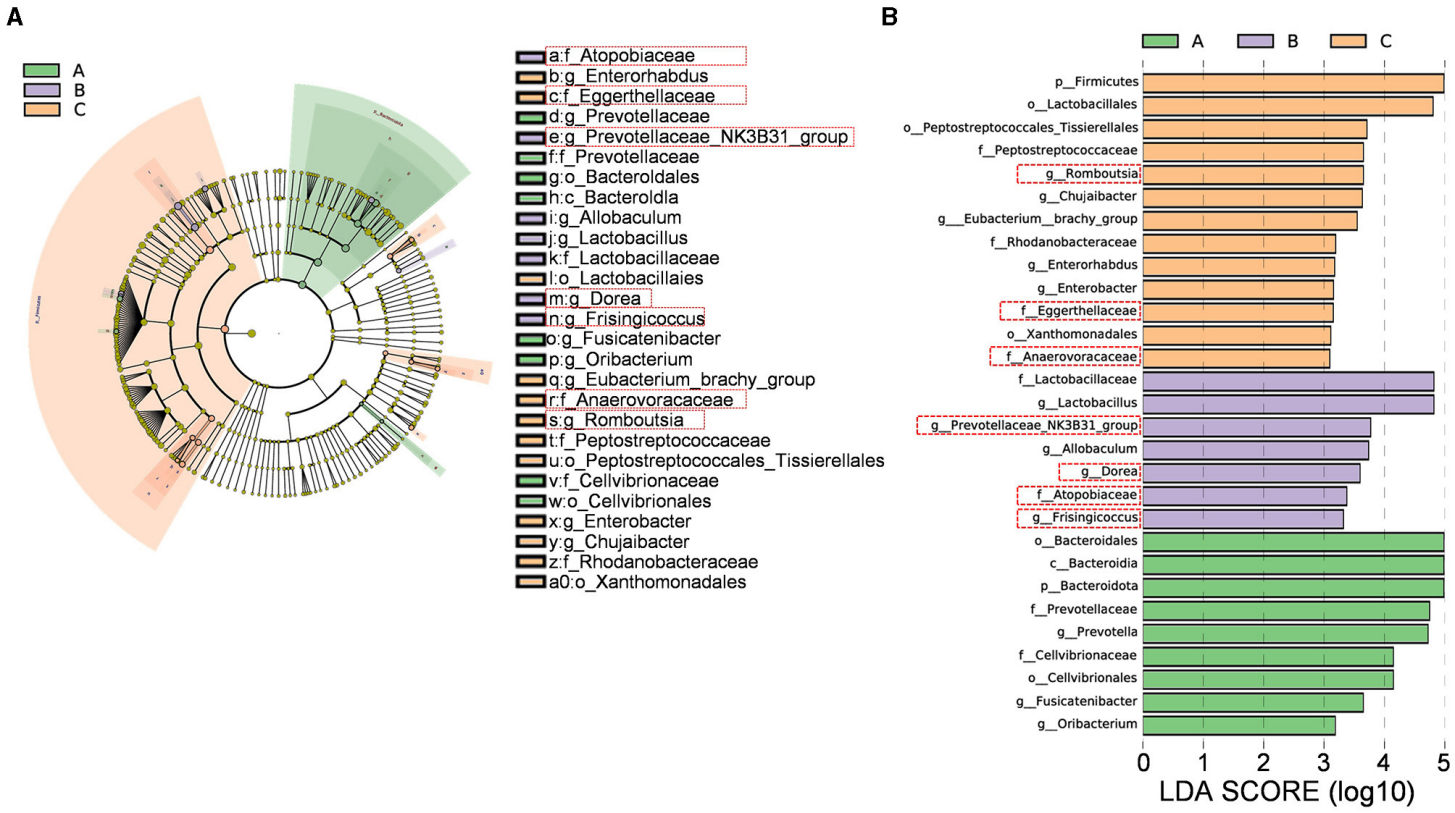
Differential bacterial species analysis. **(A)** Differential species annotation cladogram. **(B)** A histogram of the linear discriminant analysis (LDA) effect size. A, B, and C stand for the Sham, Model, and XHP-H groups, respectively.

The cladogram (Figure 10A) illustrates the relationships between different microbial groups from the phylum level to the genus level. The letters “p,” “c,” “o,” “f,” and “g” represent phylum, class, order, family, and genus, respectively. The diameter of each small circle is proportional to the relative abundance of the corresponding taxonomic unit. As shown in the linear discriminant analysis (LDA) score plot (Figure 10B), significantly different intestinal flora components were identified in the three groups [LDA score (log 10) > 3, *p* < 0.0]: p_Bacteroidota, c_Bacteroidia, o_Cellvibrionales, o_Bacteroidales, f*_Cellvibrionaceae*, f*_Prevotellaceae*, g*_Fusicatenibacter*, g*_Oribacterium*, and g*_Prevotella* in the Sham group; f_*Lactobacillaceae*, f*_Atopobiaceae*, g*_Dorea*, g*_Lactobacillus*, g_*Frisingicoccus*, g_*Prevotellaceae*_*NK3B31*_*group*, and g_*Allobaculum* in the Model group; and c_Firmicutes, o_Peptostreptococcales_Tissierellales, o_Xanthomonadales, o_Lactobacillales, f_*Rhodanobacteraceae*, f_*Peptostreptococcaceae*, f_*Eggerthellaceae*, f_*Anaerovoracaceae*, g_*Romboutsia*, g_*Eubacterium*_brachy_group, g_*Enterobacter*, g_*Chujaibacter*, and g_*Enterorhabdus* in the XJP-H group.

**Figure 11:**
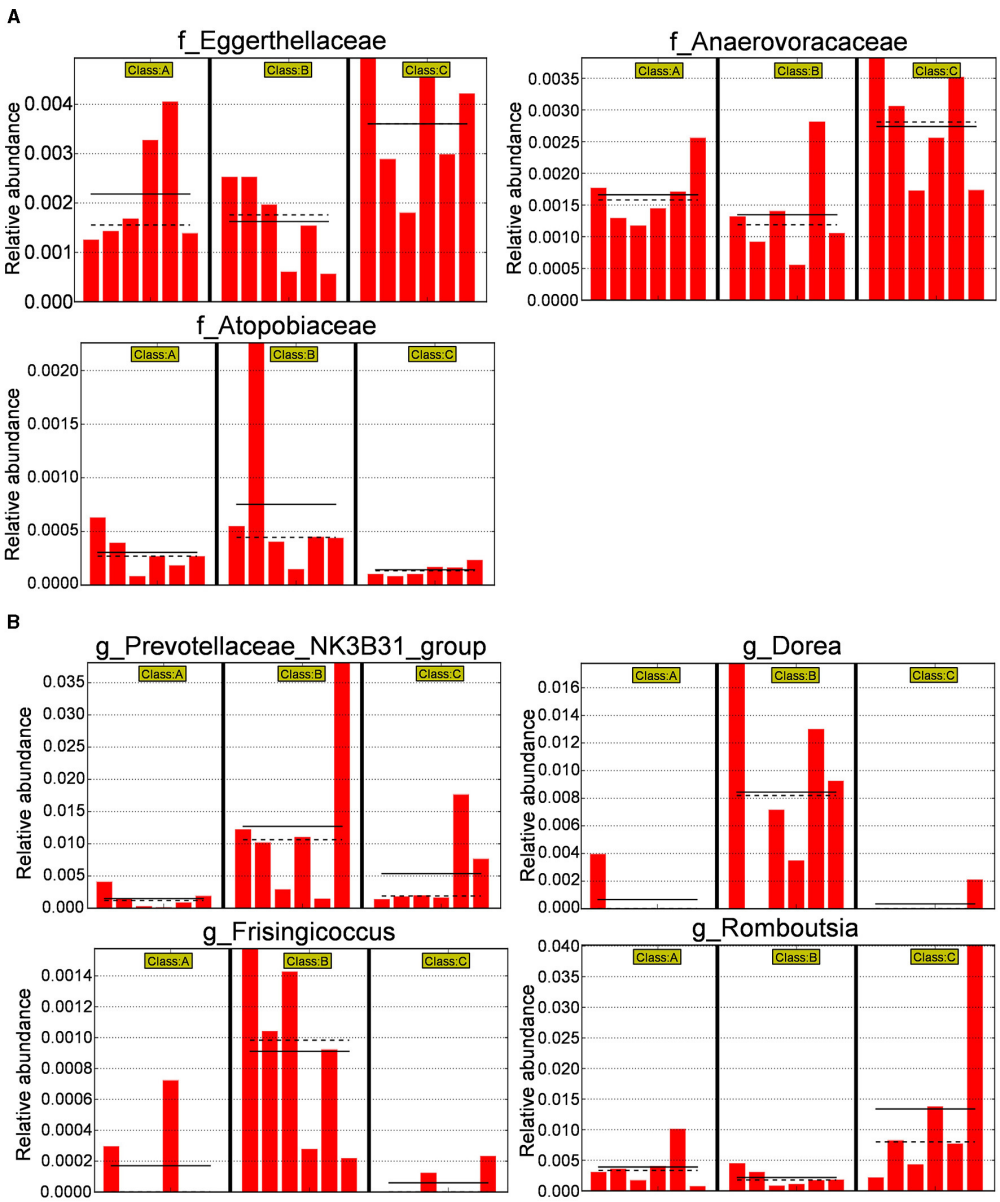
Differential bacterial species histogram. **(A)** A histogram of relative abundance at the family level of representative taxa in the three groups. **(B)** Ahistogram of relative abundance at the genus level of representative taxa in the three groups [the solid line represents the mean, and the dashed line represents the median, *n* = 6, *p < 0.05 (Supplementary material 5). A, B, and C stand for Sham, Model, and XHP-H groups, respectively].

Subsequently, a histogram comparing the relative abundance differences at the family level between the three groups (*p* < 0.05) was plotted (Figure 11A). It was observed that the relative abundance of *Eggerthellaceae* and *Anaerovoracaceae* decreased after modeling and increased after administering XJP, while the relative abundance of *Atopobiaceae* increased after modeling and decreased after administering XJP. In addition, at the genus level (*p* < 0.05), as shown in Figure 11B, it was found that the relative abundance of *Prevotellaceae_NK3B31_group, Dorea*, and *Frisingicoccus* increased after modeling and decreased after administering XJP, while the relative abundance of *Romboutsia* decreased after modeling and increased after administering XJP.

### 3.5 Correlation analysis of serum biochemical markers, serum metabolites, and gut microbiota

**Figure 12:**
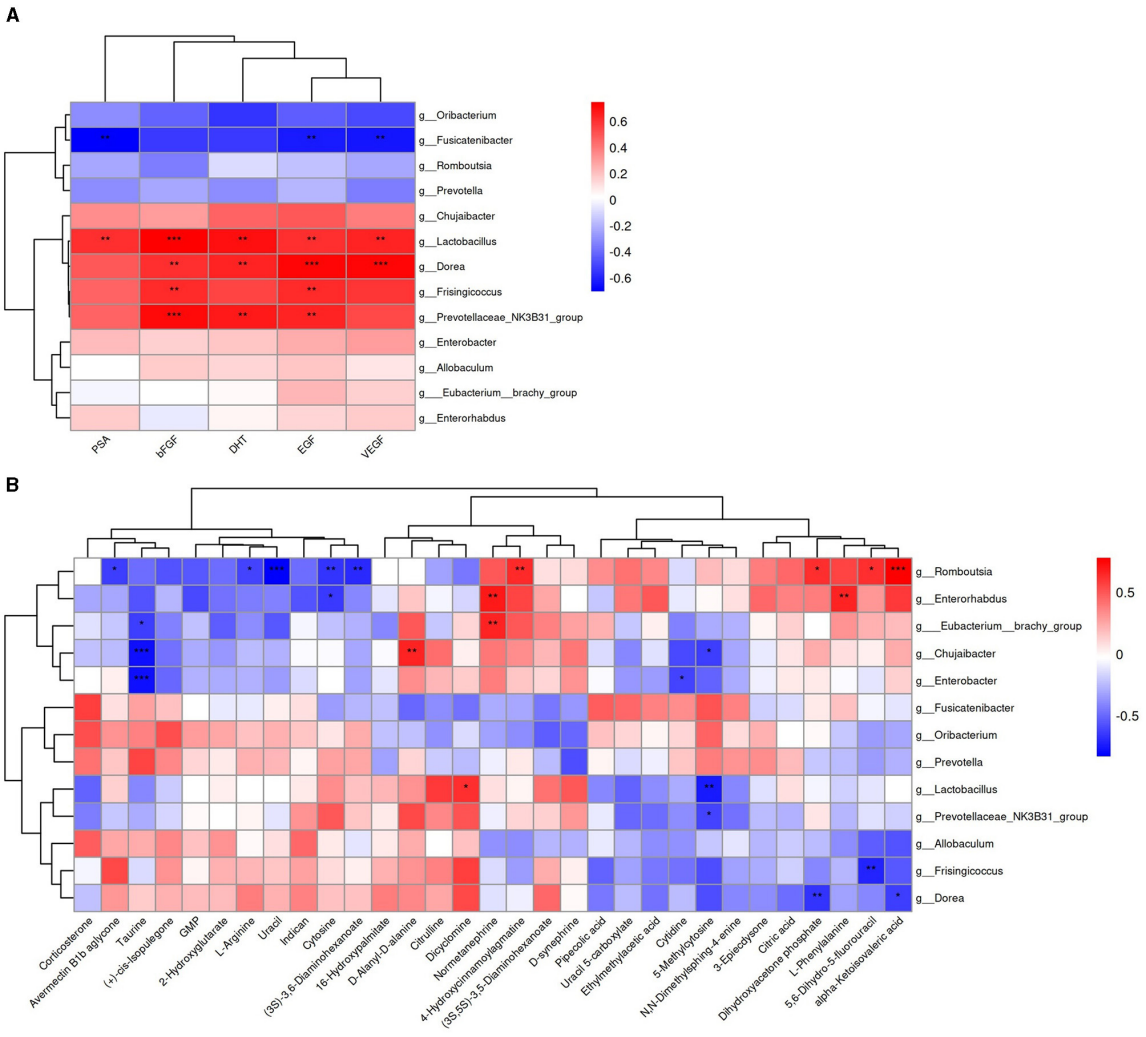
Heatmap of the Spearman correlation analysis. **(A)** Correlation between serum factors and differential bacterial genus. **(B)** Correlation between differential metabolites and differential bacterial genus. Color intensity reflects the correlation strength; *for 0.01 <*p* < 0.05, **for 0.001 <*p* ≤ 0.01, and ***for *p* ≤ 0.001.

We analyzed the functional relationships between serum factors–gut microbiota and metabolites–gut microbiota variations using the Spearman correlation coefficient. Significant correlations (*p* < 0.05, |r| > 0.6) were identified, which revealed 17 serum factor–microbiota pairs (Figure 12A, details in Supplementary material 3), with *Lactobacillus* being the most prevalent bacterium associated. In parallel, 25 metabolite–microbiota pairs showed significant correlations (Figure 12B, details in Supplementary material 3), with *Romboutsia* being most frequently linked. Notably, 5-methylcytosine, influencing pyrimidine metabolism, correlated with *Prevotellaceae_NK3B31_group, Lactobacillus*, and *Chujaibacter*. Uracil correlated with *Romboutsia*, and *Cytosine* correlated with *Romboutsia* and *Enterorhabdus*. Furthermore, L-arginine, which is essential in multiple metabolic pathways, exhibited a significant correlation with *Romboutsia*.

## 4 Discussion

This study clarifies the therapeutic effect of XJP and the pathogenesis of BPH using UPLC-MS metabolomics and 16S rRNA sequencing. PSA, a glycoprotein from prostatic epithelial cells, is crucial for prostate health, which is regulated by androgen/AR signaling. Elevated PSA levels correlate with BPH risk (La Vignera et al., 2016). In prostate tissue, testosterone converts to DHT via 5α-reductase, stimulating cell growth and differentiation. DHT binds the androgen receptor (AR) with a higher affinity, activating androgen response elements in the PSA promoter, which enhances transcription and protein synthesis (Roehrborn et al., 2008; Vignozzi et al., 2014; Lee et al., 2022). This process drives prostatic cell growth and differentiation, contributing to BPH development. Furthermore, the proliferative process of BPH is associated with the aberrant regulation of numerous growth factors (Wei et al., 2012). Vascular endothelial growth factor (VEGF) plays a pivotal role in neovascularization and in increasing vascular permeability. VEGF mainly binds to VEGF receptor 1 and VEGF receptor 2 (VEGFR1/2; Wang Q. et al., 2023; Wang R. et al., 2023; Wang S. et al., 2023). DHT stimulates the expression of VEGF in prostate epithelial RWPE-1 cells, thereby inducing BPH (Kim et al., 2019). Prostatic secretions are enriched with epidermal growth factor (EGF; Fuse et al., 1992), which promotes proliferation in cultures of rat prostate epithelial cells by binding to epidermal growth factor receptors (EGFRs) present in the prostate (Fong et al., 1991). Fibroblast growth factors (FGFs) exhibit mitogenic activity, and their receptors, fibroblast growth factor receptors (FGFRs), are crucial for prostate development. Notably, basic fibroblast growth factor (bFGF), an isoform of FGFs, is abundantly expressed in glandular epithelial and stromal cells (Ma et al., 2005). In prostatic hyperplasia tissue, TGF-β1 plays a pivotal role in facilitating the transformation of fibroblasts into myofibroblasts and inducing apoptosis in prostatic epithelial cells (Cunha et al., 2002). Furthermore, XJP modulates stromal–epithelial interactions, reducing epithelial responsiveness to IGF-1, thereby inhibiting prostatic growth (Gevaert et al., 2014). In apoptosis, Bax forms pores in mitochondrial membranes, disrupting the membrane potential and releasing cytochrome C. This, in turn, activates caspases (Boehm et al., 2012), thereby accelerating cell death. H&E staining showed that XJP reduced prostate wet weight and PI, with serum levels of DHT, PSA, EGF, bFGF, and VEGF significantly lower in rats treated with XJP compared to Model rats. TUNEL staining indicated apoptotic necrosis in the prostate tissue of BPH rats, suggesting that XJP inhibits BPH through apoptosis suppression. Immunohistochemical analysis revealed that XJP downregulates Bax, caspase-3, TGF-β1, and IGF-1, corroborating TUNEL results and reinforcing XJP's therapeutic potential in treating BPH.

The diverse non-targeted metabolomic profiles across the experiment groups aid in identifying specific metabolites and elucidating the mechanism of XJP. Our research revealed 20 serum metabolites with significant differences in Model rats post XJP intervention. The non-targeted metabolomic profiles of different groups can not only help us identify specific metabolites but also provide clues for the mechanism of action of XJP. We found that 20 serum metabolic differences were identified in Model rats after administering XJP. The KEGG enrichment analysis of metabolites between the Model group and the XJP-H group showed that multiple metabolic pathways were involved, including pyrimidine metabolism, mTOR signaling pathway, aminoacyl-tRNA biosynthesis, ABC transporters, D-amino acid metabolism, and arginine biosynthesis. Among them, the mTOR signaling pathway is closely related to various biological processes, including protein synthesis, cellular autophagy (Zhang et al., 2023), and cellular energy metabolism. Notably, uracil, cytosine, and 5-methylcytosine were enriched in pyrimidine metabolism, highlighting their role in XJP's therapeutic effect on BPH. 5-methylcytosine, a crucial epigenetic modification, regulates RNA functions, such as stability, translation, and transcription. Catalyzed by NOL1/NOP2/sun and DNMT2, it mediates cell proliferation, differentiation, apoptosis, and stress response (Li et al., 2022). Abnormal methylation patterns of 5-methylcytosine are linked to the occurrence of tumors, such as prostate cancer (Yang et al., 2013; Munari et al., 2016). Our findings suggest that 5-methylcytosine may promote RNA degradation in BPH, while XJP intervention protects against this trend. When metabolites were compared between the Sham, XJP-H, and Model groups, 5-methylcytosine expression was found to be reduced in the Model group but increased in the XJP-H group, approaching the levels observed in the Sham group. This suggests a regulatory role of 5-methylcytosine in response to XJP. TUNEL staining showed necrotic apoptotic cell death in the prostate tissue of BPH rats, indicating that 5-methylcytosine might promote RNA degradation. However, XJP protected against this degradation trend. We conclude that 5-methylcytosine is crucial in the XJP intervention for treating BPH. Its abnormal expression could impact uracil metabolism, indirectly influencing the occurrence of BPH and potentially serving as a serum metabolic biomarker.

Studies link gut microbiota imbalance to BPH, which is evident in intestinal dysfunction and flora disruption (Tsai et al., 2022). BPH compresses the rectum, causing abdominal distress, such as distension, pain, and constipation, disrupting gut function and flora. It also reduces prostatic fluid and antibacterial agents, fostering microbiota growth and inflammation. Pathogens such as *Neisseria* are found in the urethras of BPH patients (Porter et al., 2018). Prolonged drug use may worsen flora imbalance. 16S rRNA sequencing sheds light on the anti-BPH effects of XJP by analyzing the dynamics of intestinal flora. According to literature reports, *Eggerthellaceae* (*Parvibacter*) aids in food digestion and vitamin synthesis, regulating the intestinal immune system (Little et al., 2024). *Anaerovoracaceae* decomposes complex carbohydrates in food to generate beneficial short-chain fatty acids for intestinal cell energy (Nichols et al., 2017). *Romboutsia*, a Firmicutes bacterium, regulates physiological processes such as blood sugar and lipids, exhibiting probiotic and immune-modulating effects (Yin et al., 2023). Conversely, the overgrowth of certain *Atopobiaceae* bacteria may trigger intestinal inflammation (Wong et al., 2022). The *Prevotellaceae_NK3B31_group* is associated with health issues, including colitis exacerbation (Elinav et al., 2011). Changes in the quantity and activity of *Dorea* bacteria, a gas-producing bacterium, were observed in patients with abnormal sugar metabolism (Zhao et al., 2023). *Frisingicoccus*, a gram-positive bacterium, is depleted in patients with diarrhea-dominant irritable bowel syndrome complicated by functional dyspepsia (Kovaleva et al., 2023).

The integrated analysis of gut microbiota, serum factors, and metabolites offers broader perspectives for identifying BPH markers, enhancing our comprehension of gut microbiota's role in BPH pathogenesis beyond single-omics approaches. Elevated urine PSA levels in BPH patients correlate with increased *Lactobacillus* abundance (Mariotti et al., 2024), as suggested in prior studies. EGF, by inhibiting MAP kinase-mediated JNK2 activation and cytoskeleton remodeling, safeguards intestinal barriers against osmotic stress, which are further strengthened by *Lactobacillus casei* (Samak et al., 2021). Notably, *Lactobacillus* positively correlates with EGF, suggesting a potential synergistic gut-protective effect. Moreover, 5-methylcytosine, modulating pyrimidine metabolism, is strongly linked to *Prevotellaceae_NK3B31_group, Lactobacillus*, and *Chujaibacter*, while uracil and cytosine show significant associations with *Romboutsia* and *Enterorhabdus*, respectively. The findings suggest that gut microbiota dysbiosis disrupts pyrimidine metabolism in BPH rats, emphasizing the need for further metabolomics analyses in urine and prostate tissue. L-arginine, a versatile metabolic biomarker, hints at a potential connection to BPH development. Additionally, serum organic acid metabolites differentiate between prostatitis, BPH, and PCA (He et al., 2023). This study highlights the protective effects of XJP against BPH in rats, which is closely linked to gut microbiota and metabolite shifts.

This study has certain limitations, particularly its reliance on bioinformatics and statistical analyses, which lack direct biological validation. Being prospective in nature, we aim to build upon our animal model findings by conducting future fecal microbiota transplantation studies to substantiate the therapeutic potential of specific gut microbiota in benign prostatic hyperplasia (BPH).

## 5 Conclusion

**Figure 13:**
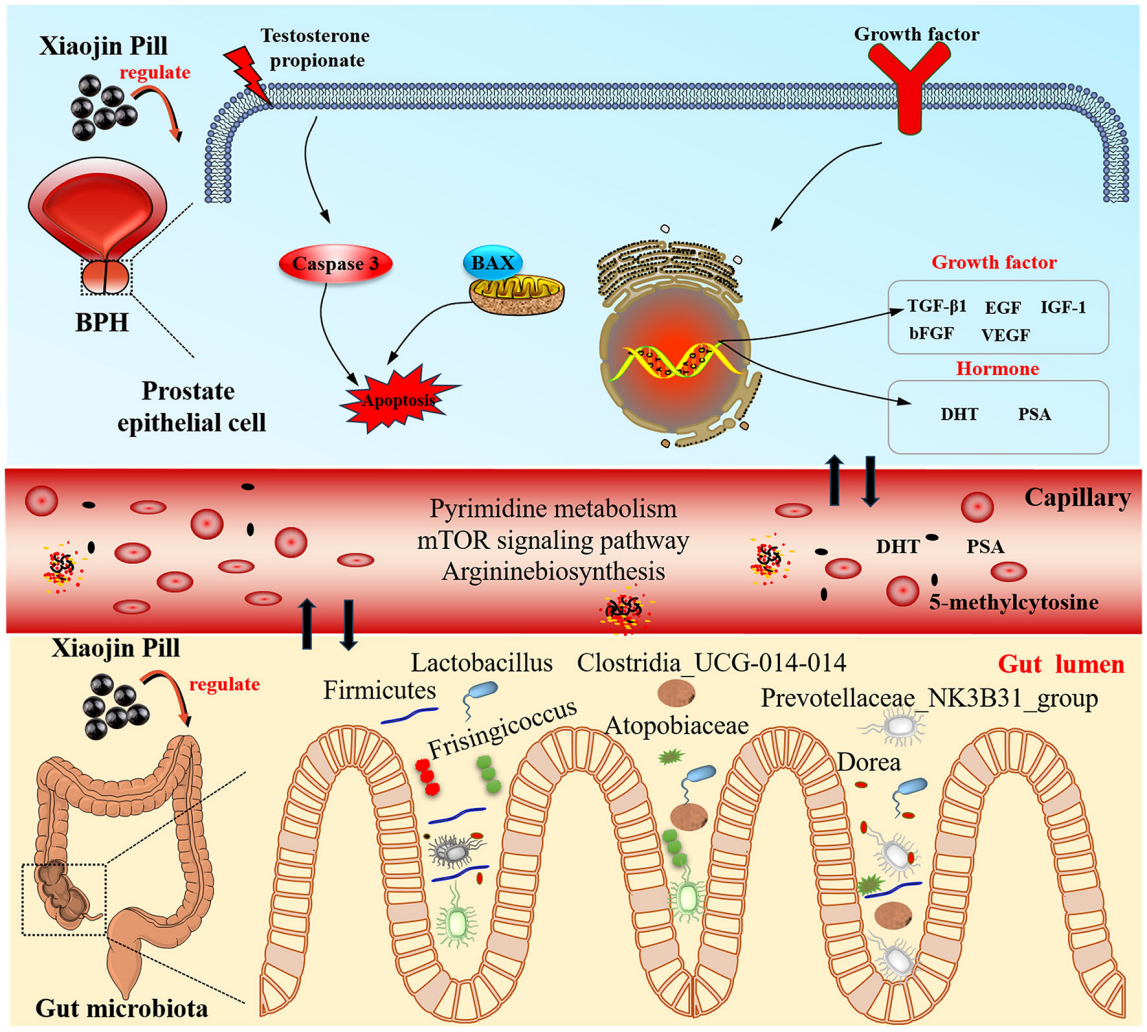
The mechanisms of Xiaojin Pill therapy for treating BPH through metabolomics and gut microbiota analysis.

Our research has revealed that XJP possesses a synergistic anti-BHP effect through multiple components targeting multiple gut microbiota and metabolic pathways (Figure 13). It involves the regulation of sex hormone levels, growth factors, and anti-epithelial cell apoptosis processes, demonstrating the'protective effect of XJP on the prostate tissue as well as PSA, DHT, EGF, bFGF, VEGF, Bax, caspase-3, TGF-β1, and IGF-1 serum levels in BPH rats. It is possible that XJP achieves this effect by participating in the caspase-3 and TGF-β signaling pathways. We focused on elucidating the interaction mechanism between host-regulated specific gut microbiota (such as *Eggerthellaceae, Anaerovoracaceae, Romboutsia, Atopobiaceae, Prevotellaceae_NK3B31_group, Dorea*, and *Frisingicoccus*) and various metabolic pathways (including pyrimidine metabolism, arginine biosynthesis, and the mTOR signaling pathway). In particular, pyrimidine metabolism is closely associated with the bacterial genera *Romboutsia, Prevotellaceae_NK3B31_group, Lactobacillus, Chujaibacter*, and *Enterorhabdus*, with 5-methylcytosine potentially serving as a serum biomarker for BPH. This study provides new insights into the biological mechanisms of XJP in treating BPH and offers data supporting the secondary development of XJP as a traditional Chinese medicine in China.

## Data availability statement

The data presented in the study are deposited in the NCBI repository, accession number PRJNA1144503.

## Ethics statement

The animal study was approved by Sichuan Academy of Traditional Chinese Medicine. The study was conducted in accordance with the local legislation and institutional requirements.

## Author contributions

YY: Writing – review & editing, Writing – original draft. YQ: Writing – original draft. YL: Writing – original draft. JY: Writing – original draft. KC: Writing – original draft. XY: Writing – original draft. HH: Writing – review & editing. LY: Writing – review & editing. JZ: Writing – review & editing. JW: Writing – review & editing.

## Supplementary material

Supplementary Table 1 The chemical component list of Xiaojin Pill.

Supplementary material 1 Metabolomics analysis and 16S rRNA sequencing.

Supplementary material 2 MS identification chart of Xiaojin Pill ingredients.

Supplementary material 3 Potential biomarker and correlation analysis table.

Supplementary material 4 Original identification chart of serum metabolites.
