# Supplementary material for: Exploring the potential mechanism of Xiaojin Pill therapy for benign prostatic hyperplasia through metabolomics and gut microbiota analysis
Source: Front Microbiol. 2024 Aug 21;15:1431954. doi: 10.3389/fmicb.2024.1431954 (PMC11371748; doi:10.3389/fmicb.2024.1431954)
Supplement: Supplementary material 1 — Metabolomics analysis and 16S rRNA sequencing. [file Data_Sheet_2.docx]

Supplementary Material

Exploring the Potential Mechanism of Xiaojin Pill Therapy for Benign Prostatic Hyperplasia Based on Metabolomics and Gut Microbiota Analysis

**Yuying Yang^1,2^, Yunyun Quan^2^, Yunteng Liu^3^, Juhua Yang^3^, Keyu Chen^4^, Xiaozhou You^2^, Hua Hua^2,3^, Liangchun Yan^2^, Junning Zhao^2*^ and Jianbo Wang^1,2,4*^**

^1^School of Pharmacy, Southwest Medical University, Luzhou 646000, China;

^2^Sichuan Institute for Translational Chinese Medicine, SICHUAN ACADEMY OF CHINESE MEDICINE SCIENCES, Key Laboratory of Biological Evaluation of Translational Chinese Medicine（TCM） Quality of National Administration of TCM, Sichuan Key Laboratory of Translational Medicine of TCM, Sichuan Authentic Medicine System Development Engineering Technology Research Center, Sichuan Authentic Medicine Formation Principle and Quality Evaluation Engineering Research Center, Chengdu, China;

^3^Chengdu University, Chengdu 6100413, China;

^4^Shaanxi University of Traditional Chinese Medicine, Xianyang 712046, China）

*** Correspondence: Jianbo WANG**, **E-mail：[yyswjb@fmmu.edu.cn](mailto:yyswjb@fmmu.edu.cn)；Junning ZHAO, E-mail：[zarmy@189.cn](mailto:zarmy@189.cn)**

# Metabolomic Analysis

## Sample Preparation

Five rat serum samples from the Sham, Model, and XJP-H groups were randomly selected, thawed at 4 ℃, and vortexed for 1 minute. An appropriate amount of sample was accurately transferred into a centrifuge tube. 400µL of methanol solution was added, and the mixture was vortexed for 1 minute. The sample was then centrifuged at 12,000 rpm at 4℃ for 10 minutes. The supernatant was transferred to a new centrifuge tube and concentrated to dryness. 150µL of 2-chloro-L-phenylalanine (4 ppm) solution prepared in 80% methanol-water was added to redissolve the sample. The supernatant was filtered through a 0.22 μm membrane, and the filtrate was transferred to an injection vial for analysis.

## Method Validation

An appropriate amount of metabolite solution was taken from each of the 15 samples and mixed into a uniform QC solution. During the continuous detection of 15 samples, a QC solution was injected every two samples.

## UPLC-MS Analysis Conditions

Chromatographic conditions [1]: Using the Thermo Vanquish ultra-high-performance liquid chromatography system, an ACQUITY UPLC® HSS T3 (2.1×100 mm, 1.8µm, Waters) column was employed. The flow rate was set at 0.3 mL/min, and the column temperature was maintained at 40 ℃. The injection volume was 2 μL. The gradient elution program for the positive ion mode was as follows: initially, mobile phase A (0.1% formic acid in water) accounted for 92%, and B (0.1% formic acid in acetonitrile) for 8%. From 1 to 8 minutes, A gradually decreased to 2%, while B increased to 98%. From 8 to 10 minutes, A and B maintained 2% and 98%, respectively. From 10 to 10.1 minutes, A rapidly increased to 98%, and B decreased to 8%. Finally, from 10.1 to 12 minutes, A and B returned to their initial proportions, completing the elution cycle. The negative ion mode gradient elution program was similar: from 0 to 1 minute, mobile phase A (5 mM ammonium formate in water) accounted for 92%, and B (acetonitrile) for 8%. The subsequent changes in A and B were similar to the positive ion mode.

Mass spectrometric conditions [2]: The Thermo Orbitrap Exploris 120 mass spectrometer was used with an electrospray ionization source (ESI). The spray voltages for positive and negative ions were 3.50 kv and -2.50 kv, respectively. The sheath gas flow rate was 40 arb, and the auxiliary gas flow rate was 10 arb. The capillary temperature was set at 325 ℃. The resolution for full MS scans was 60,000, with a scan range of m/z 100~1000. High-energy collision dissociation (HCD) was used for MS/MS fragmentation, with a collision energy of 30%. The secondary resolution was set at 15,000. During MS/MS signal acquisition, the top four ions were preferentially selected for fragmentation, and dynamic exclusion was used to remove unnecessary MS/MS information.

## Data Analysis

Using the MSConvert tool in the Proteowizard software package (v3.0.8789) [3], the original mass spectrometry data files were converted into mzXML file format. The R XCMS software package was used for peak detection, peak filtering, and peak alignment [4], with relevant parameter settings to generate a list of metabolite quantifications. A support vector regression correction method based on QC samples was adopted, and metabolites with a relative standard deviation (RSD) < 30% were selected for subsequent analysis. The molecular weights of primary metabolites were determined based on the parent ion m/z and adduct ion information detected in the quantitative list, and metabolites with ppm < 30 ppm were screened. These metabolites were then matched with databases such as HMDB [5], MassBank [6], LipidMaps [7], mzCloud [8], and KEGG [9] to identify the primary metabolites. Identification of secondary metabolites was achieved by matching the information detected in the secondary spectra of the quantitative list with the fragment ions and other information of each metabolite in the database. Principal component analysis (PCA) and partial least squares discriminant analysis (PLS-DA) were performed on the sample data using the R language Ropls package [10]. When the variable importance in projection (VIP) value of the first principal component of each metabolite was >1 and P < 0.05, the metabolite was considered to have statistically significant differences, and the model was tested for overfitting using a permutation test method. The MetaboAnalyst [11] software package was used to perform functional pathway enrichment and topological analysis on the selected differential metabolites. The enriched pathways were visualized using the KEGG Mapper visualization tool to browse the differential metabolites and pathway maps.

# 16S rRNA Sequencing

## Extraction of Genomic DNA

Randomly selected fecal samples from six rats each from the Sham, Model, and XJP-H groups were used for DNA extraction. The DNA was extracted from the rat feces following the instructions of the MagPure Stool/Soil DNA KF Kit. The purity of the DNA samples was detected using 1% agarose gel electrophoresis, and the concentration and total amount of DNA were calculated using a NanoDrop UV-Vis spectrophotometer to ensure the quality of the extracted DNA. High-throughput sequencing of intestinal microbiota 16S rRNA was performed by Shanghai OE Biotech Co., Ltd.

## PCR Amplification and 16S rRNA Sequencing

PCR amplification involves a two-step PCR amplification procedure, as follows. The V3–V4 region of the 16S rRNA gene was amplified using the 343F (5’-TACGGRAGGCAGCAG-3’) and downstream 798R (5’-AGGGTATCTAATCCT-3’) primers. The first-round PCR reaction system mainly consists of 2×Gflex PCR Buf, 343F and 798R primers, Template DNA, and Tks Gflex DNA Polymerase. The program is as follows: ① Pre-denaturation at 94 ℃ for 5 minutes, 1 cycle; ② Denaturation at 94 ℃ for 30 seconds; ③ Annealing at 56 ℃ for 30 seconds, 26 cycles; ④ Extension at 72 ℃ for 20 seconds; ⑤ Final extension at 72 ℃ for 5 minutes, 1 cycle; ⑥ Holding at 4 ℃. After the thermal cycling is completed, agarose gel electrophoresis is used to identify whether the PCR amplification product has obtained the expected fragment size. Magnetic beads are used to purify the PCR products. For the second-round PCR, Adapter I5 and Adapter I7 are added, and the first-round PCR product is used as the template. Specific nucleotide tags (barcodes) are added to the designated regions of the 343F and 798R primers. The second-round PCR program is as follows: Annealing at 56 ℃ for 30 seconds, 7 cycles, with the remaining steps the same as the first-round PCR program. After the thermal cycling is completed, the PCR products are again electrophoresed and purified using magnetic beads. Finally, the PCR products are precisely quantified using the Qubit dsDNA Assay Kit. Depending on the sequencing requirements of different samples, an equal amount of mixing is performed. After passing the library construction qualification, the NovaSeq 6000 SP Reagent kit v1.5 (500 cycles) sequencing kit is used, and the PE 250 paired-end sequencing method (each DNA fragment is sequenced for 250 base pairs at both ends) is set up. Sequencing is performed using the Illumina NovaSeq 6000 sequencing platform.

## Bioinformatics Analysis

The raw image data obtained through high-throughput sequencing is in the FASTQ format. After base recognition, it is converted into raw data. Using Cutadapt software, the PCR primer sequences in the raw data are trimmed. The processed data is then imported into QIIME 2 (version 2020.11) [12] software, where the DADA2 [13] command is used to perform quality filtering, denoising, assembly, and chimera removal. The resulting valid tags are dereplicated, and sequences with 100% similarity are clustered into a representative sequence. These representative sequences are then aligned with the Silva database (version 138) [14], and analyzed using the default parameters of the q2-feature-classifier software to annotate each representative sequence's phylogenetic information, including Phylum, Class, Order, Family, and Genus, ultimately generating an Amplicon Sequence Variant (ASV) feature table.

QIIME software is employed to statistically analyze the Alpha and Beta diversity of the biological communities. The Chao1, ACE, Observed species, and PD whole tree indices are used to assess the Alpha diversity of the 18 samples, and a one-way ANOVA is performed for group comparisons. Quantitative analysis is carried out using Adonis (PERMANOVA analysis) based on the Binary Jaccard distance algorithm, followed by PCoA analysis to evaluate the Beta diversity by assessing the differences and similarities in the microbial community structures among groups. The relative abundances of the top 15 species at the phylum and genus levels in the intestinal microbiota are statistically analyzed using a one-way ANOVA to identify differences in microbial composition. The LEfSe (Linear Discriminant Analysis Effect Size) method is employed to quantify the contributions of microbial communities to group differences at various taxonomic levels by combining linear discriminant analysis (LDA) (with LDA score (log 10) > 3, P < 0.05) and the Kruskal-Wallis non-parametric test. A higher LDA score (log 10) indicates a more significant contribution of species abundance to the observed differences.

# Reference

1. Zelena E, Dunn W B, Broadhurst D, et al. Development of a robust and repeatable UPLC− MS method for the long-term metabolomic study of human serum[J]. Analytical chemistry, 2009, 81(4): 1357-1364.
2. WANT E J, MASSON P, MICHOPOULOS F, et al. Global metabolic profiling of animal and human tissues via UPLC-MS [J]. Nature Protocols, 2013, 8(1): 17-32.
3. Navarro-Reig M, Jaumot J, García-Reiriz A, et al. Evaluation of changes induced in rice metabolome by Cd and Cu exposure using LC-MS with XCMS and MCR-ALS data analysis strategies[J]. Analytical and bioanalytical chemistry, 2015, 407: 8835-8847.
4. Want E J, Masson P, Michopoulos F, et al. Global metabolic profiling of animal and human tissues via UPLC-MS[J]. Nature protocols, 2013, 8(1): 17-32.
5. Trygg J, Wold S. Orthogonal projections to latent structures (O‐PLS)[J]. Journal of Chemometrics: A Journal of the Chemometrics Society, 2002, 16(3): 119-128.
6. Kieffer D A, Piccolo B D, Vaziri N D, et al. Resistant starch alters gut microbiome and metabolomic profiles concurrent with amelioration of chronic kidney disease in rats[J]. American Journal of Physiology-Renal Physiology, 2016, 310(9): F857-F871.
7. Wishart D S, Tzur D, Knox C, et al. HMDB: the human metabolome database[J]. Nucleic acids research, 2007, 35(suppl_1): D521-D526.
8. Horai H, Arita M, Kanaya S, et al. MassBank: a public repository for sharing mass spectral data for life sciences[J]. Journal of mass spectrometry, 2010, 45(7): 703-714.
9. Sud M, Fahy E, Cotter D, et al. Lmsd: Lipid maps structure database[J]. Nucleic acids research, 2007, 35(suppl_1): D527-D532.
10. Aurélie R, Ying X, Eric E, et al. Analysis of the Human Adult Urinary Metabolome Variations with Age, Body Mass Index, and Gender by Implementing a Comprehensive Workflow for Univariate and OPLS Statistical Analyses[J]. 2015.
11. **a J, Wishart D S. Web-based inference of biological patterns, functions and pathways from metabolomic data using MetaboAnalyst[J]. Nature protocols, 2011, 6(6): 743-760.
12. Callahan B J , Mcmurdie P J , Rosen M J , et al. DADA2: High-resolution sample inference from Illumina amplicon data[J]. Nature Methods,2016.
13. Reproducible, interactive, scalable and extensible microbiome data science using QIIME 2[J]. Nature Biotechnology, 37(8):852-857
14. Quast C, Pruesse E, Yilmaz P, et al. The SILVA ribosomal RNA gene database project: improved data processing and web-based tools. Nucleic Acids Research, 2013, 41: D590-D596.
