# Supplementary figures and images for: Exploring the potential mechanism of Xiaojin Pill therapy for benign prostatic hyperplasia through metabolomics and gut microbiota analysis (part 1 of 2)
Source: Front Microbiol. 2024 Aug 21;15:1431954. doi: 10.3389/fmicb.2024.1431954 (PMC11371748; doi:10.3389/fmicb.2024.1431954)

Caffeic acid

Relative intensity

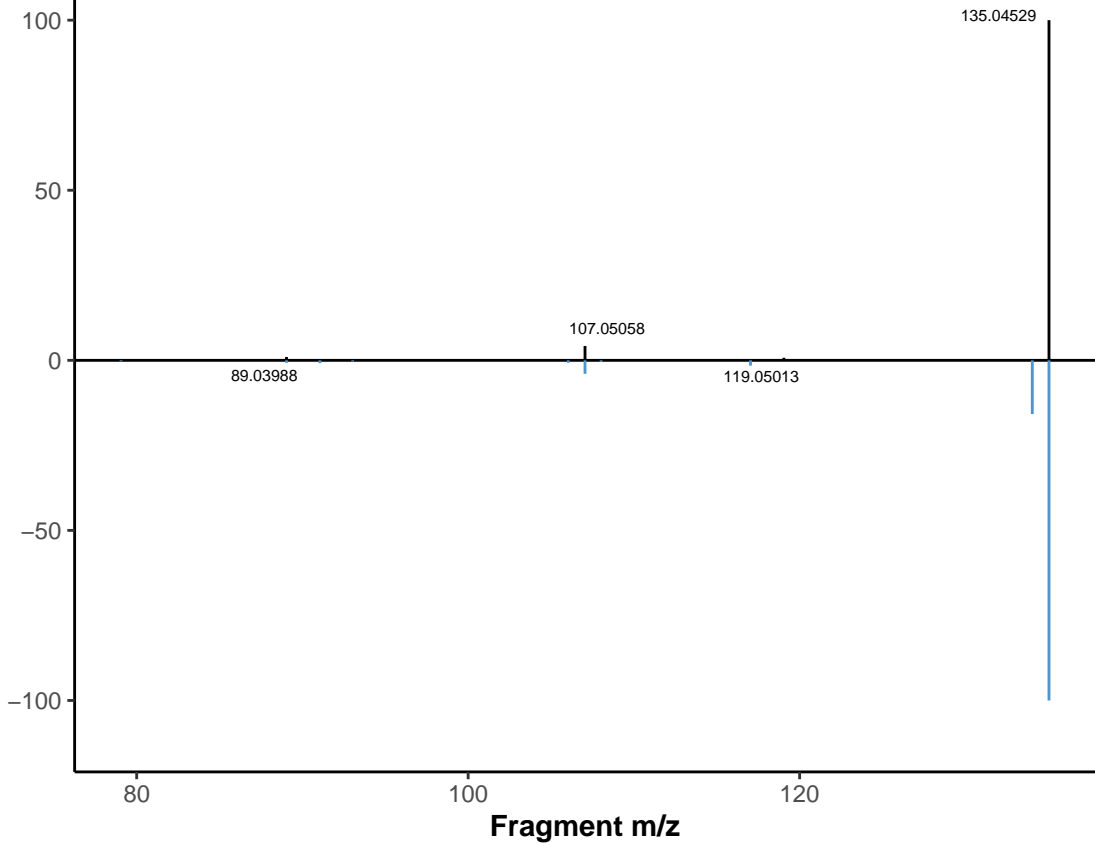

Supplement: Supplementary material 2 — MS identification chart of Xiaojin Pill ingredients. [file Data_Sheet_3.zip › Supplementary Material S2/Negative-1013.pdf]

# Azelaic acid

Relative intensity

100  
50  
0  
-50  
-100

100

150

Fragment m/z

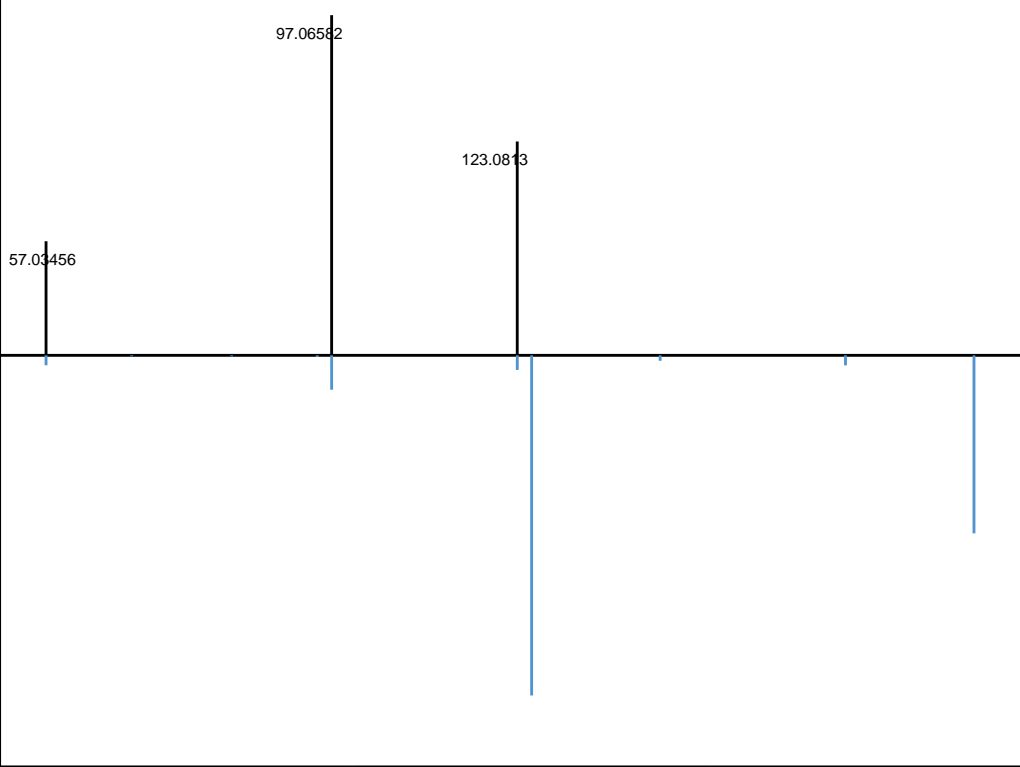

Supplement: Supplementary material 2 — MS identification chart of Xiaojin Pill ingredients. [file Data_Sheet_3.zip › Supplementary Material S2/Negative-1137.pdf]

Quinic acid

Relative intensity

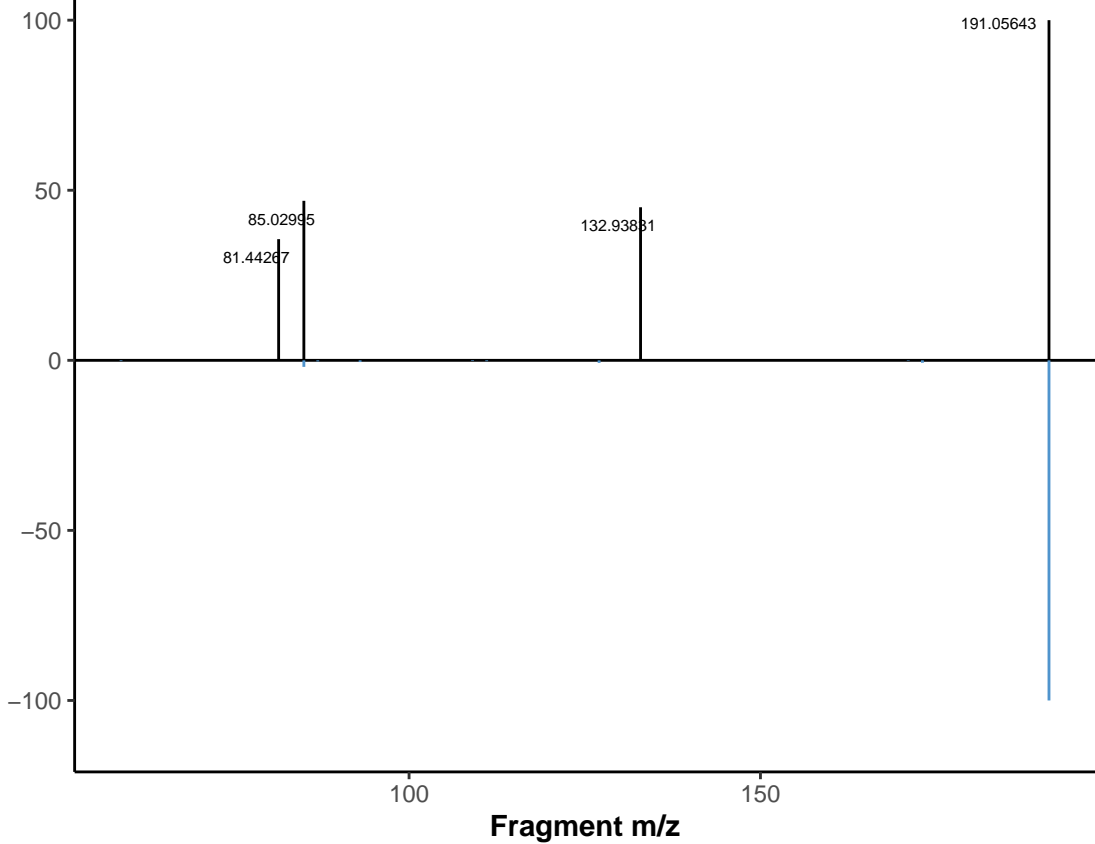

Supplement: Supplementary material 2 — MS identification chart of Xiaojin Pill ingredients. [file Data_Sheet_3.zip › Supplementary Material S2/Negative-1194.pdf]

Cinnamic acid

Relative intensity

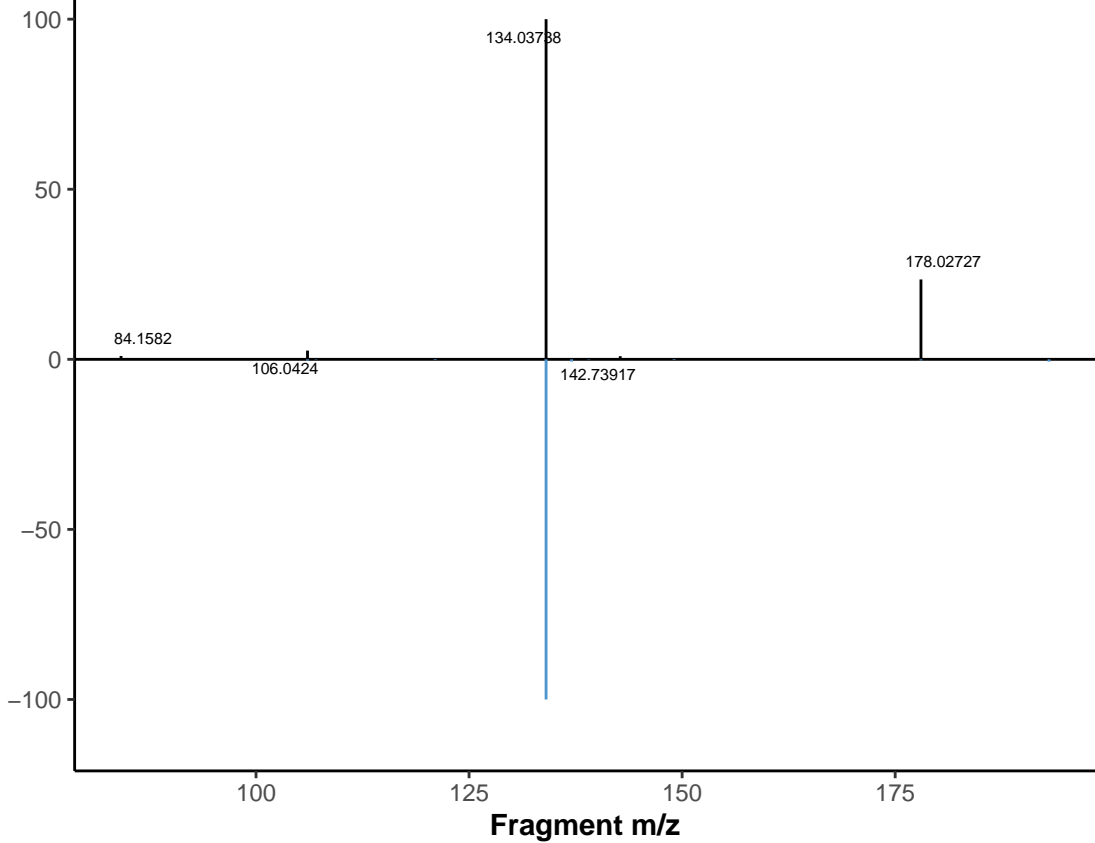

Supplement: Supplementary material 2 — MS identification chart of Xiaojin Pill ingredients. [file Data_Sheet_3.zip › Supplementary Material S2/Negative-1225.pdf]

Bergaptol

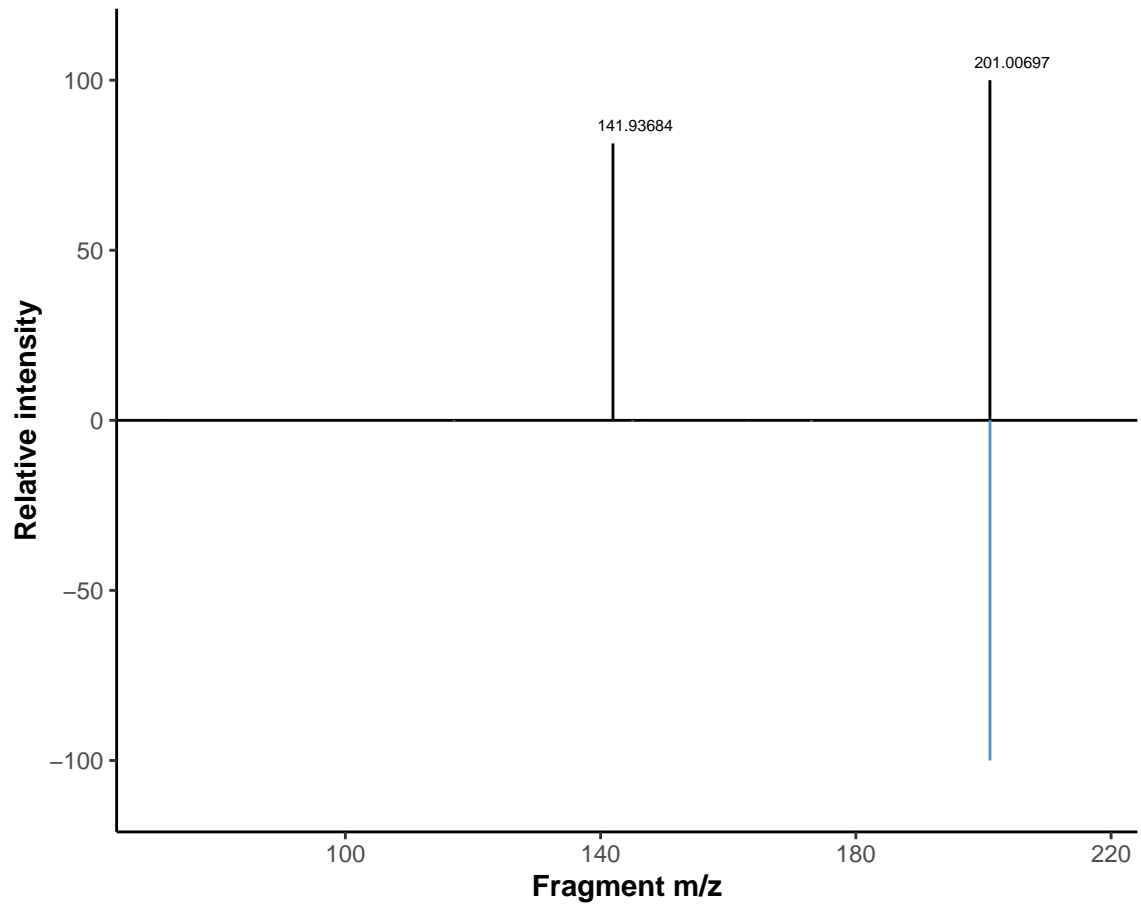

Supplement: Supplementary material 2 — MS identification chart of Xiaojin Pill ingredients. [file Data_Sheet_3.zip › Supplementary Material S2/Negative-1357.pdf]

# Calcium pantothenate

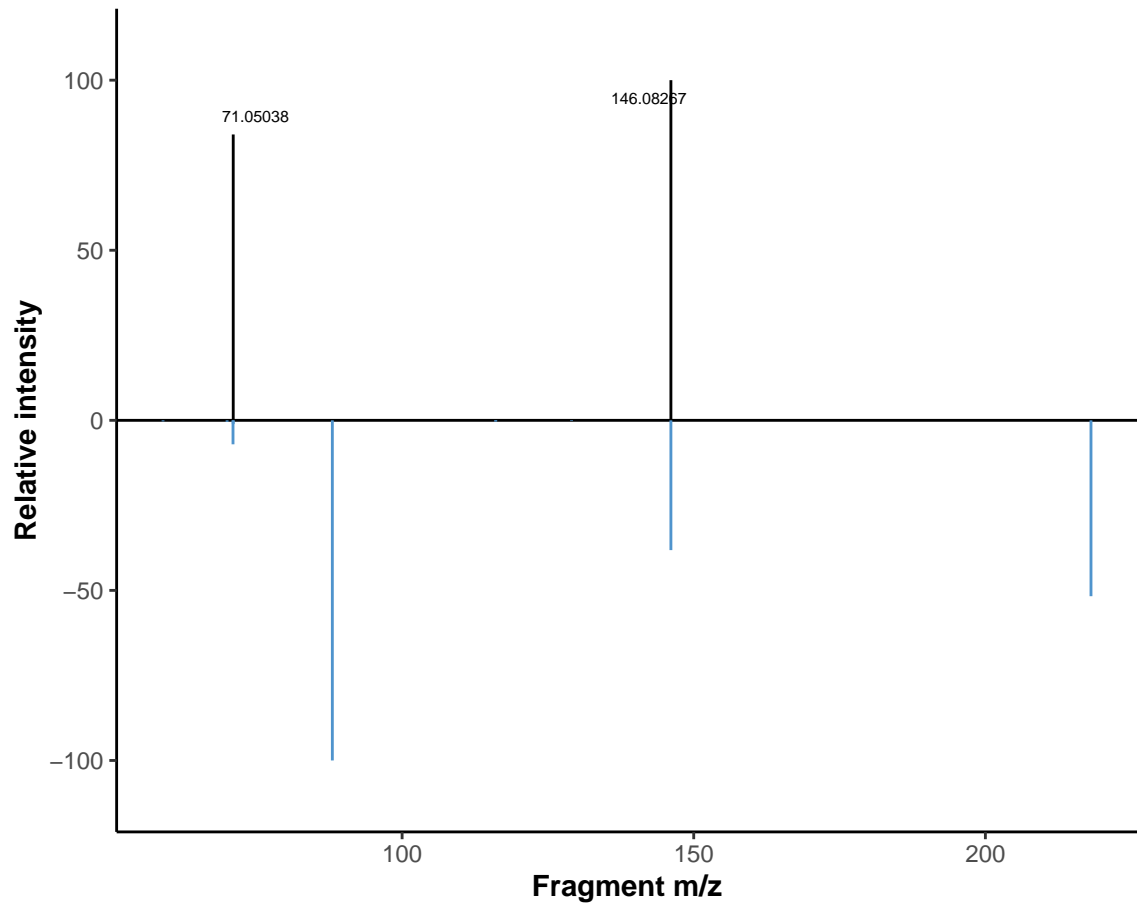

Supplement: Supplementary material 2 — MS identification chart of Xiaojin Pill ingredients. [file Data_Sheet_3.zip › Supplementary Material S2/Negative-1656.pdf]

Dihydroartemisinin acid

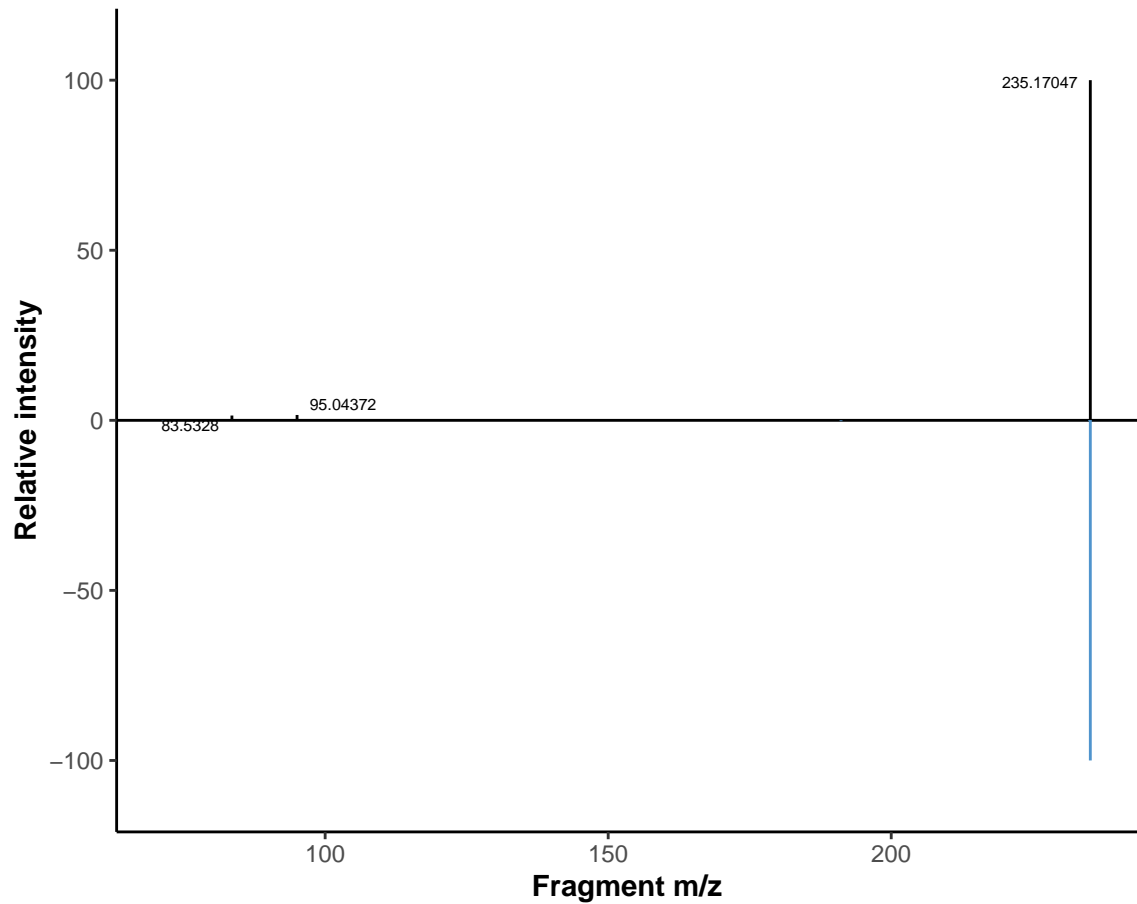

Supplement: Supplementary material 2 — MS identification chart of Xiaojin Pill ingredients. [file Data_Sheet_3.zip › Supplementary Material S2/Negative-1967.pdf]

# Naringenin chalcone

Relative intensity

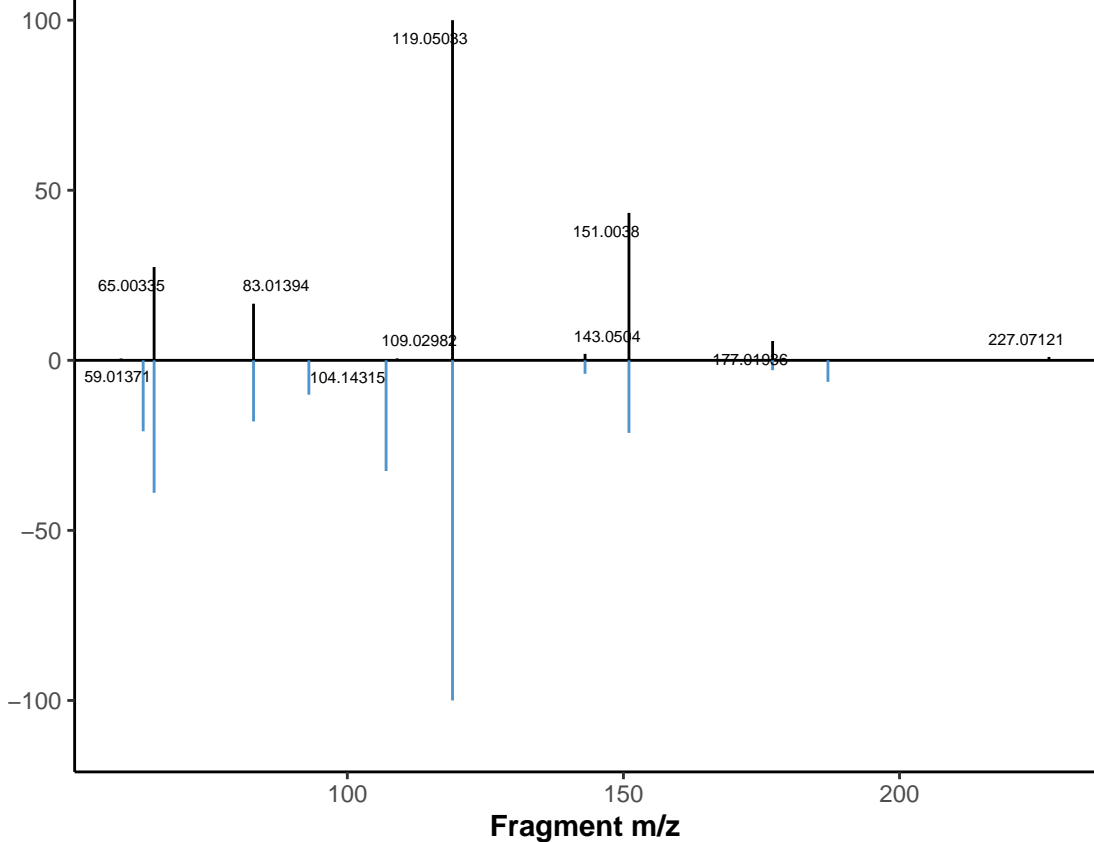

Supplement: Supplementary material 2 — MS identification chart of Xiaojin Pill ingredients. [file Data_Sheet_3.zip › Supplementary Material S2/Negative-2637.pdf]

Octyl gallate

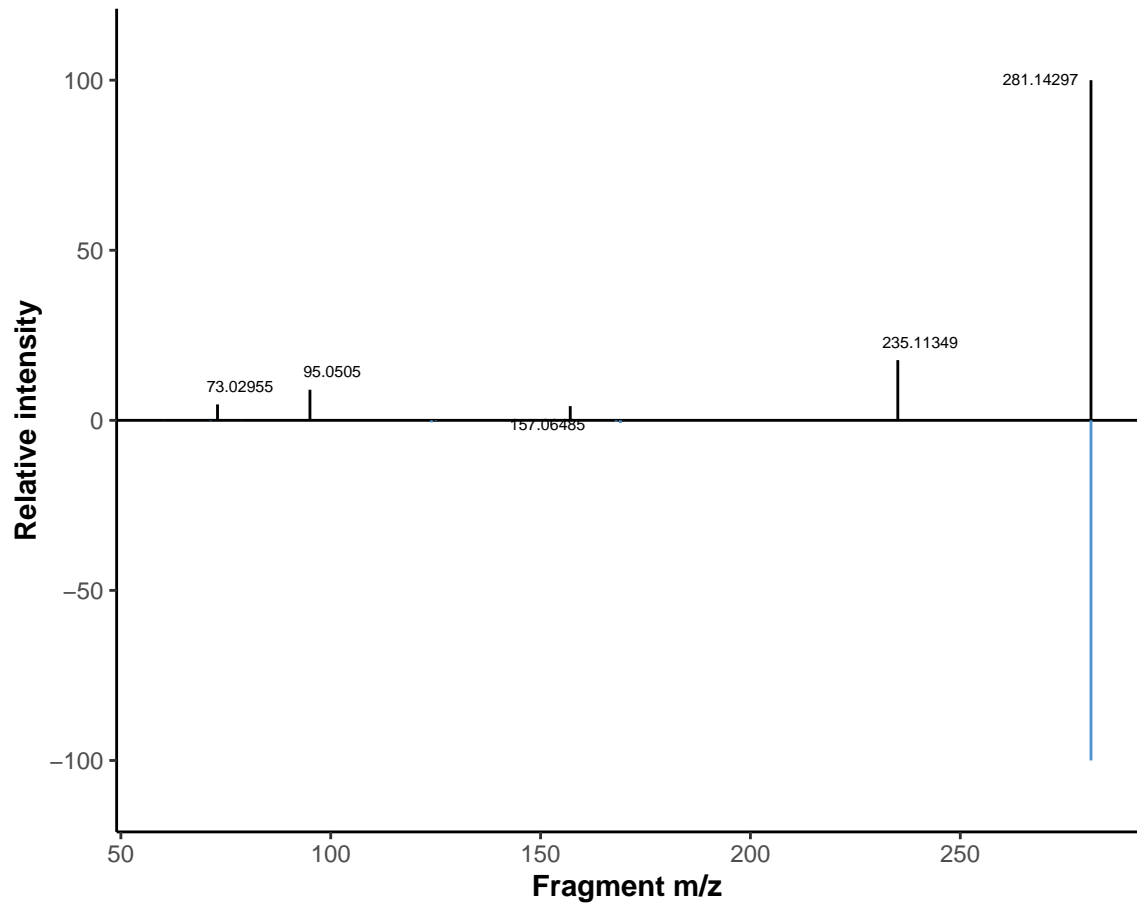

Supplement: Supplementary material 2 — MS identification chart of Xiaojin Pill ingredients. [file Data_Sheet_3.zip › Supplementary Material S2/Negative-2851.pdf]

# Isosakuranetin

Relative intensity

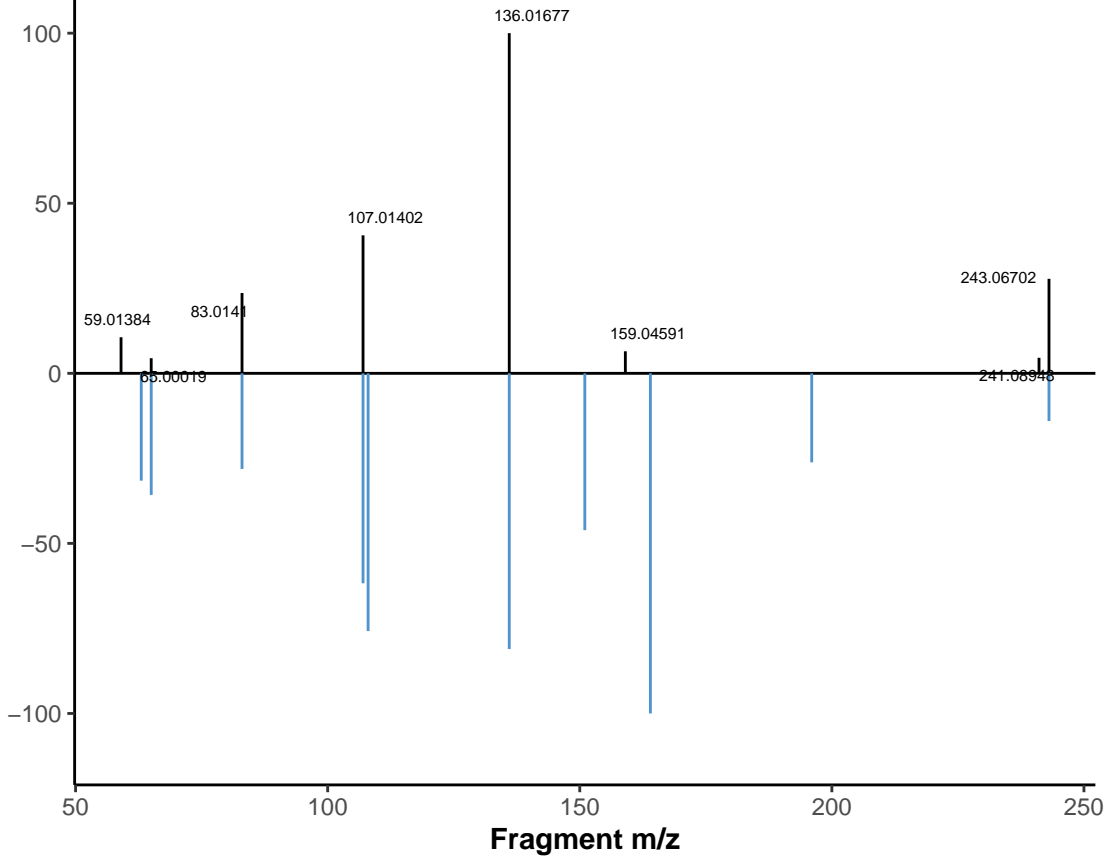

Supplement: Supplementary material 2 — MS identification chart of Xiaojin Pill ingredients. [file Data_Sheet_3.zip › Supplementary Material S2/Negative-2924.pdf]

p-Hydroxybenzaldehyde

Relative intensity

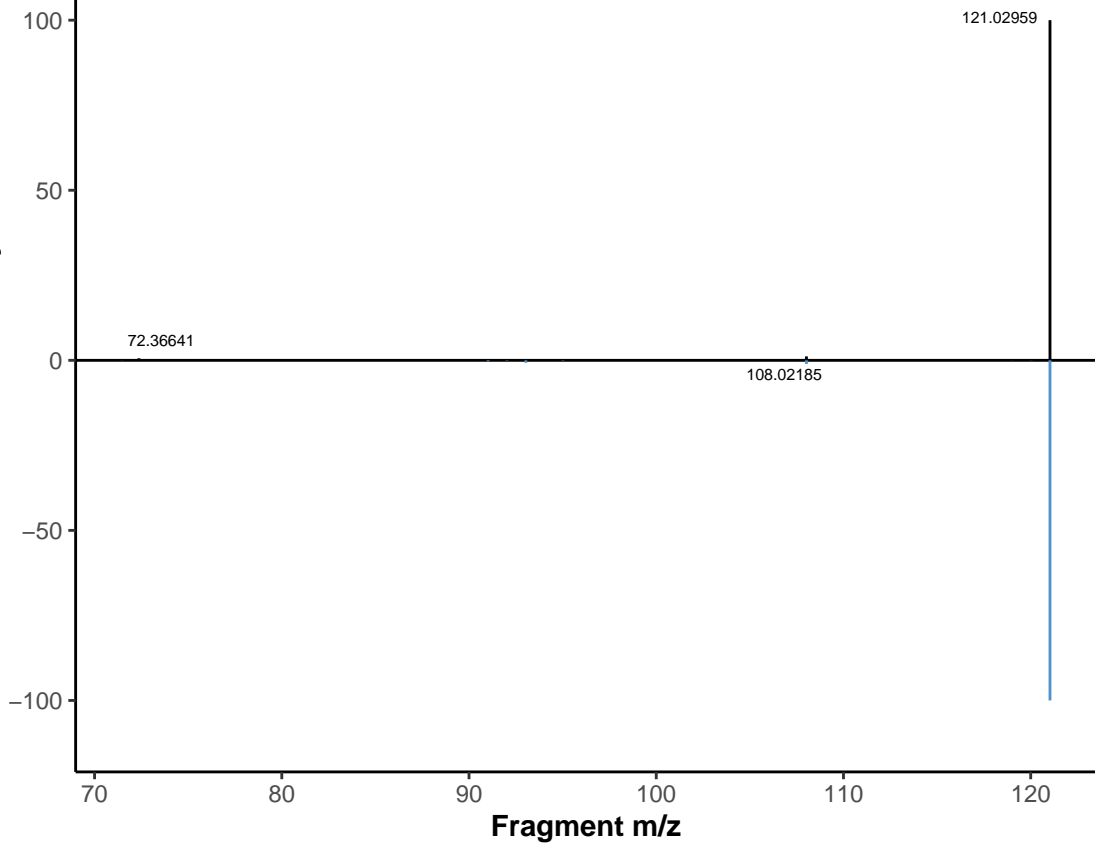

Supplement: Supplementary material 2 — MS identification chart of Xiaojin Pill ingredients. [file Data_Sheet_3.zip › Supplementary Material S2/Negative-329.pdf]

# Methyl hexadecanoate

Relative intensity

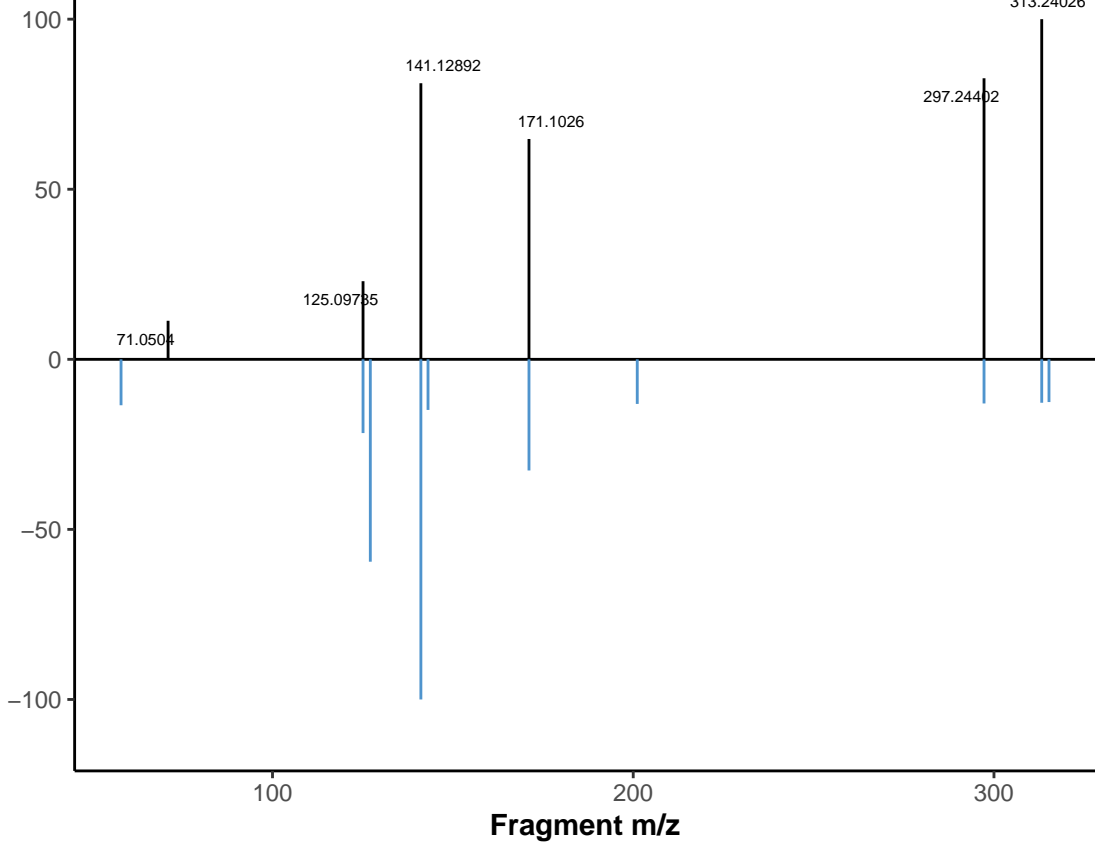

Supplement: Supplementary material 2 — MS identification chart of Xiaojin Pill ingredients. [file Data_Sheet_3.zip › Supplementary Material S2/Negative-3596.pdf]

# Ginkgolic acid (C13:0)

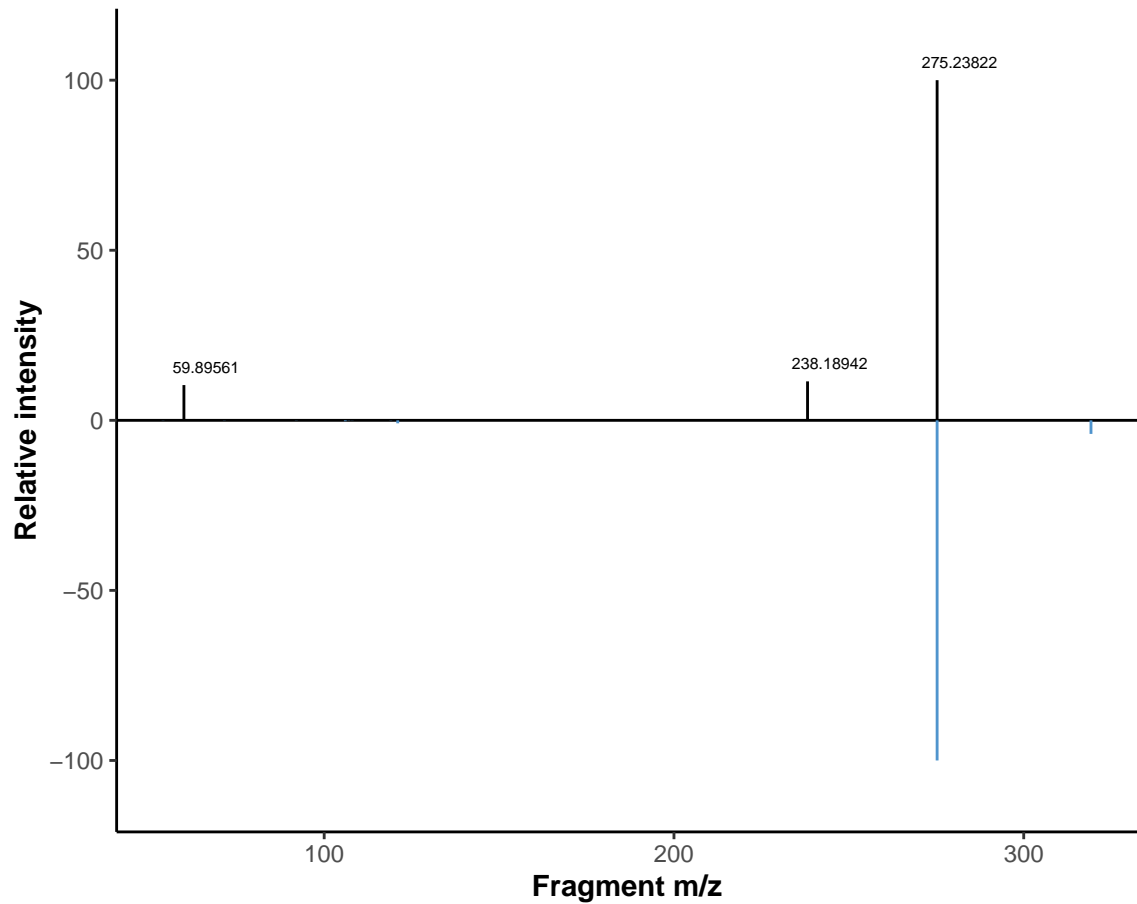

Supplement: Supplementary material 2 — MS identification chart of Xiaojin Pill ingredients. [file Data_Sheet_3.zip › Supplementary Material S2/Negative-3688.pdf]

# Sucrose

Relative intensity

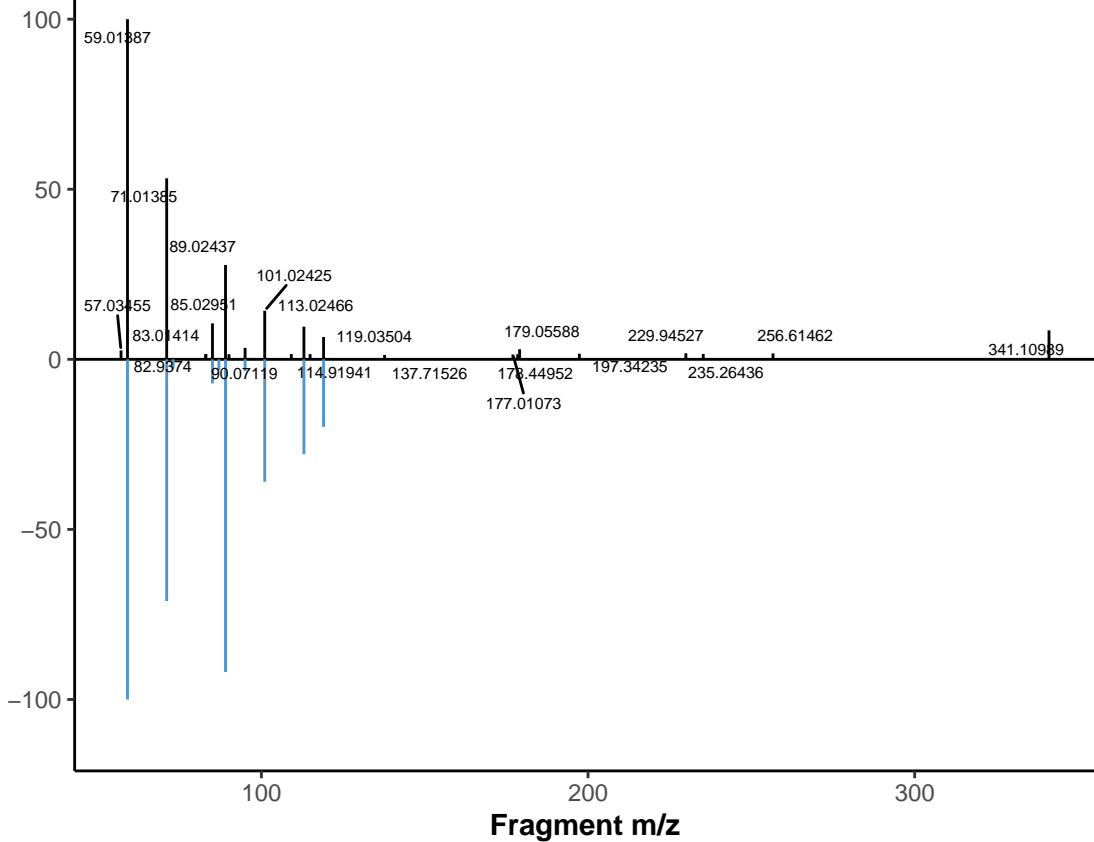

Supplement: Supplementary material 2 — MS identification chart of Xiaojin Pill ingredients. [file Data_Sheet_3.zip › Supplementary Material S2/Negative-4142.pdf]

# Ginkgolic Acid C15:1

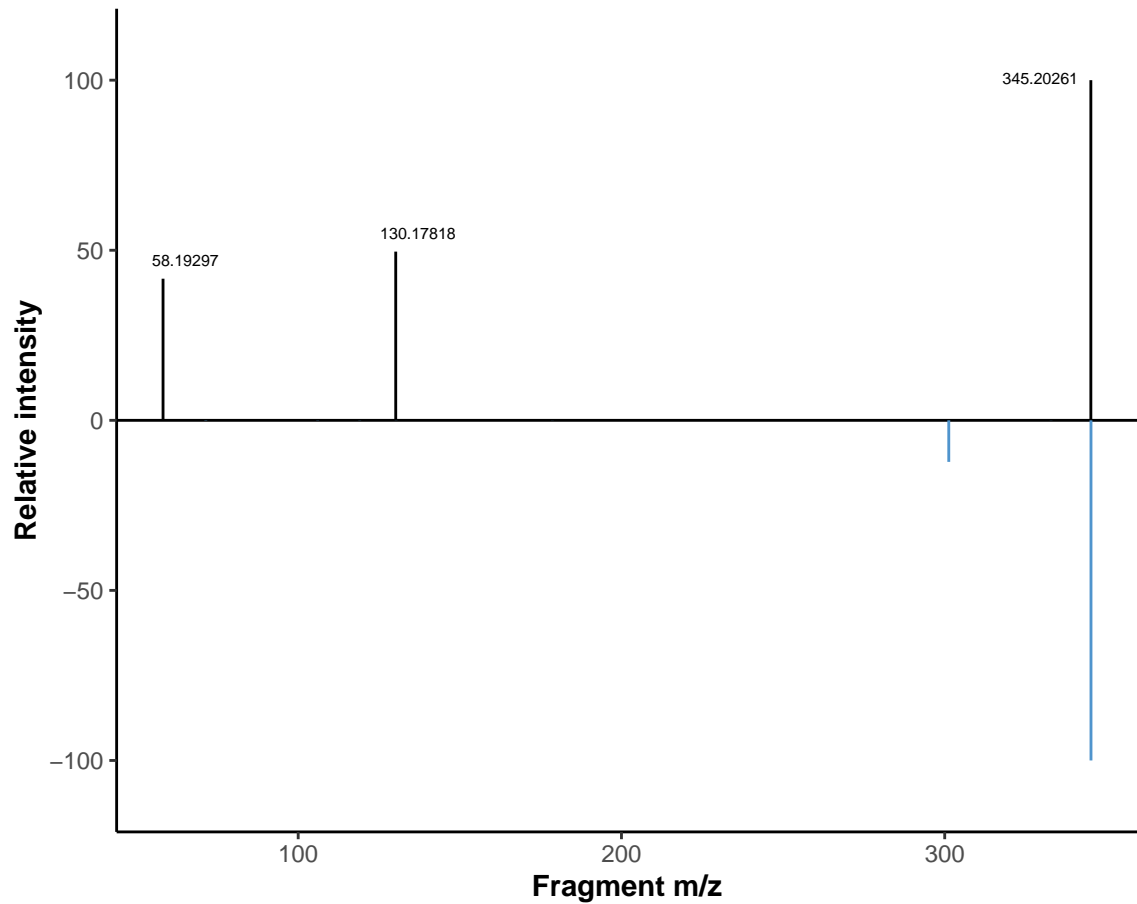

Supplement: Supplementary material 2 — MS identification chart of Xiaojin Pill ingredients. [file Data_Sheet_3.zip › Supplementary Material S2/Negative-4232.pdf]

# Andrographolide

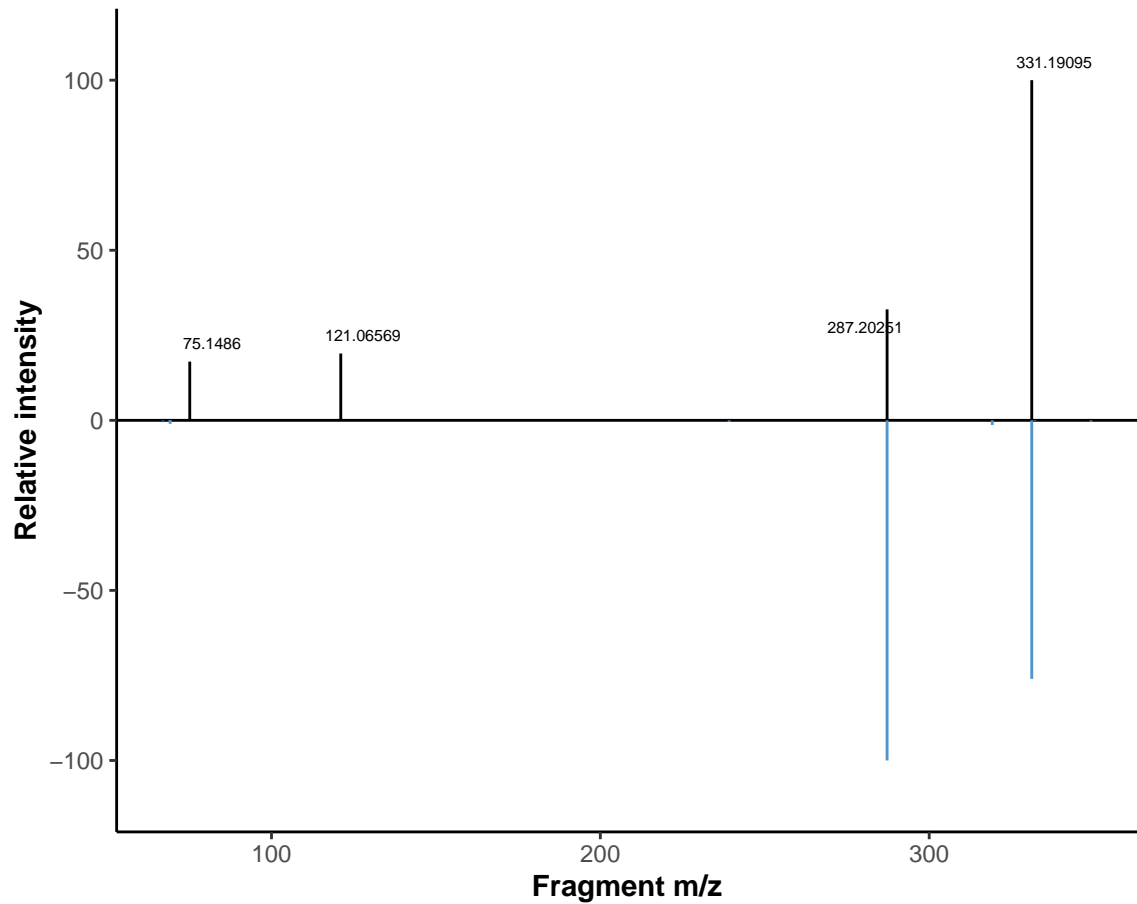

Supplement: Supplementary material 2 — MS identification chart of Xiaojin Pill ingredients. [file Data_Sheet_3.zip › Supplementary Material S2/Negative-4303.pdf]

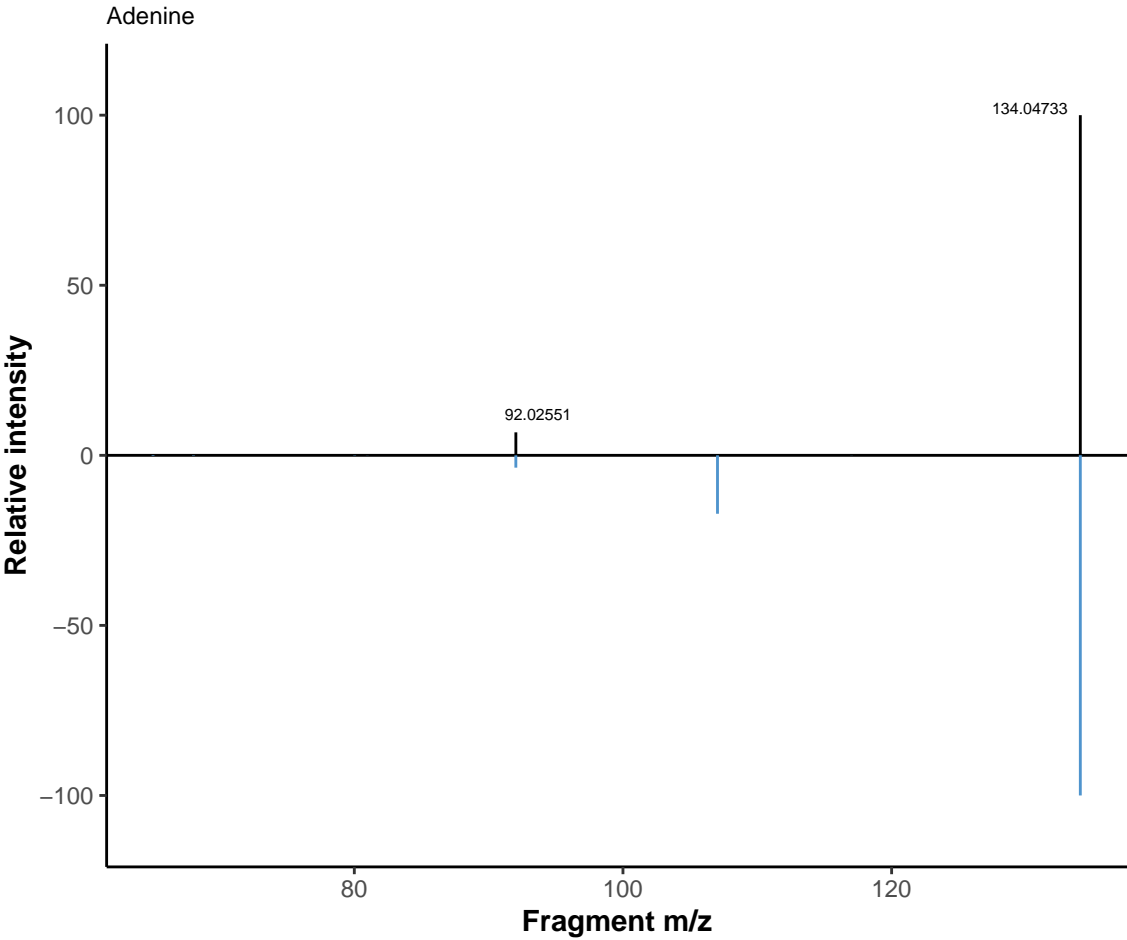

Supplement: Supplementary material 2 — MS identification chart of Xiaojin Pill ingredients. [file Data_Sheet_3.zip › Supplementary Material S2/Negative-461.pdf]

# Ginkgolic acid C17-1

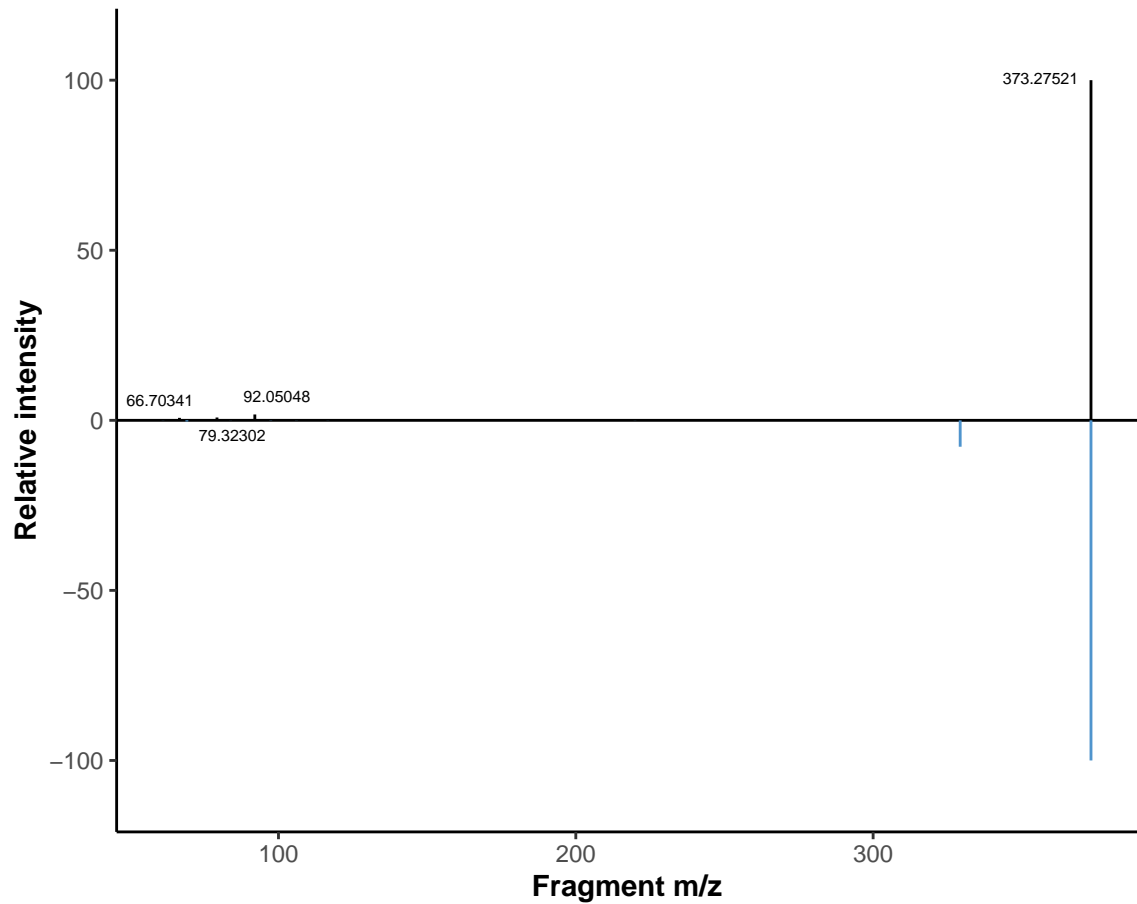

Supplement: Supplementary material 2 — MS identification chart of Xiaojin Pill ingredients. [file Data_Sheet_3.zip › Supplementary Material S2/Negative-4790.pdf]

# Salicylic acid

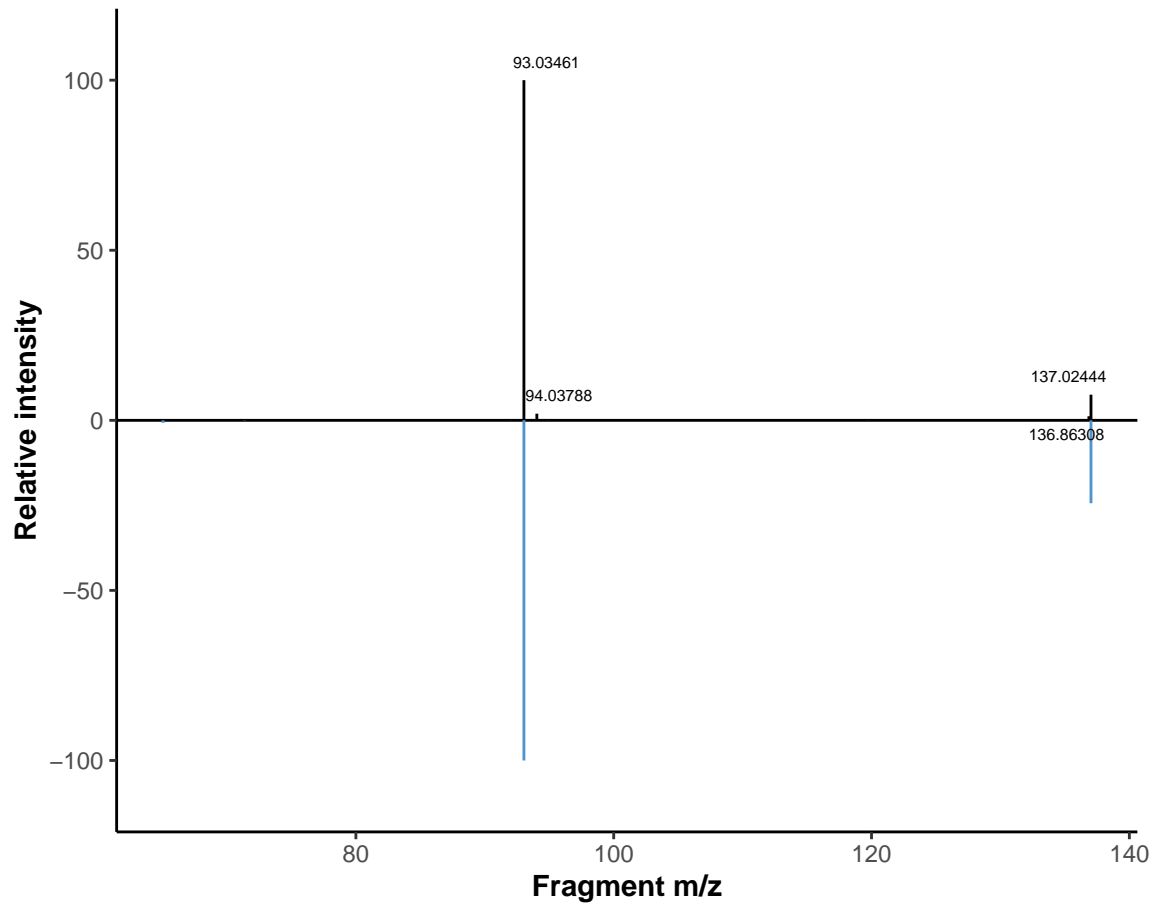

Supplement: Supplementary material 2 — MS identification chart of Xiaojin Pill ingredients. [file Data_Sheet_3.zip › Supplementary Material S2/Negative-501.pdf]

Protocatechualdehyde

Relative intensity

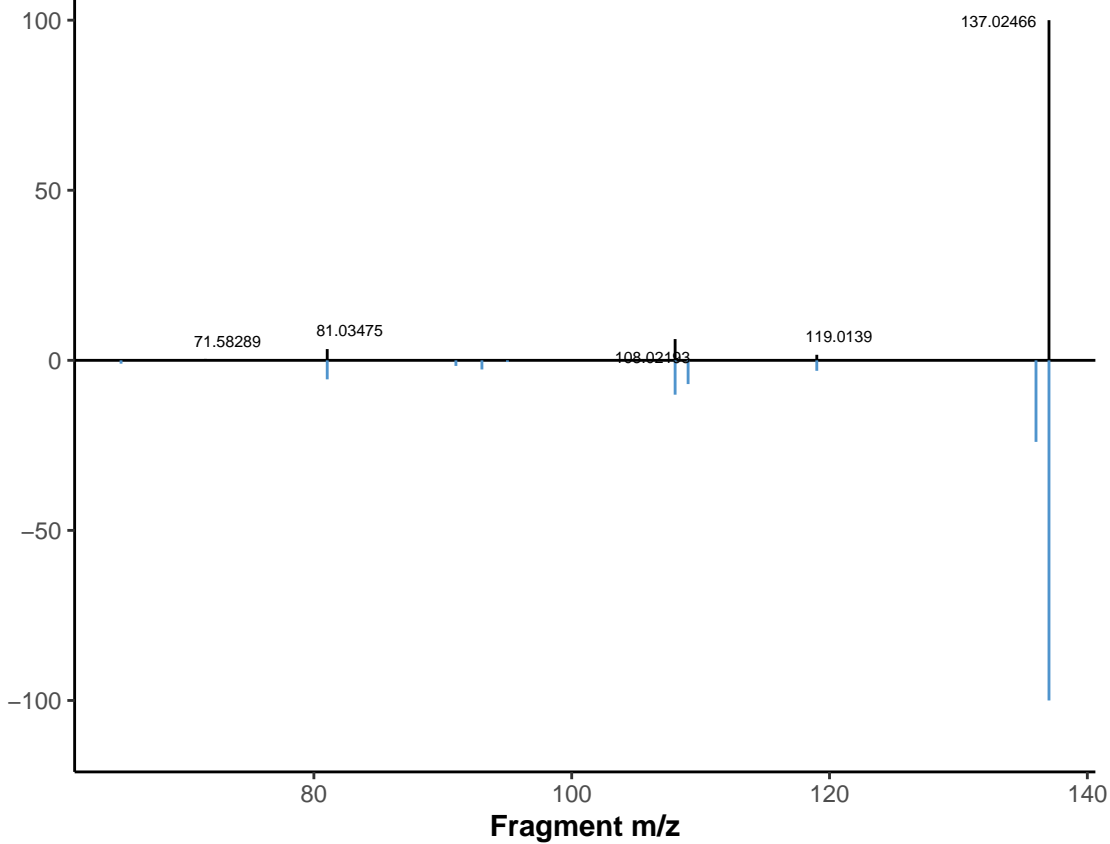

Supplement: Supplementary material 2 — MS identification chart of Xiaojin Pill ingredients. [file Data_Sheet_3.zip › Supplementary Material S2/Negative-502.pdf]

# Hydoxycholeic acid

Relative intensity

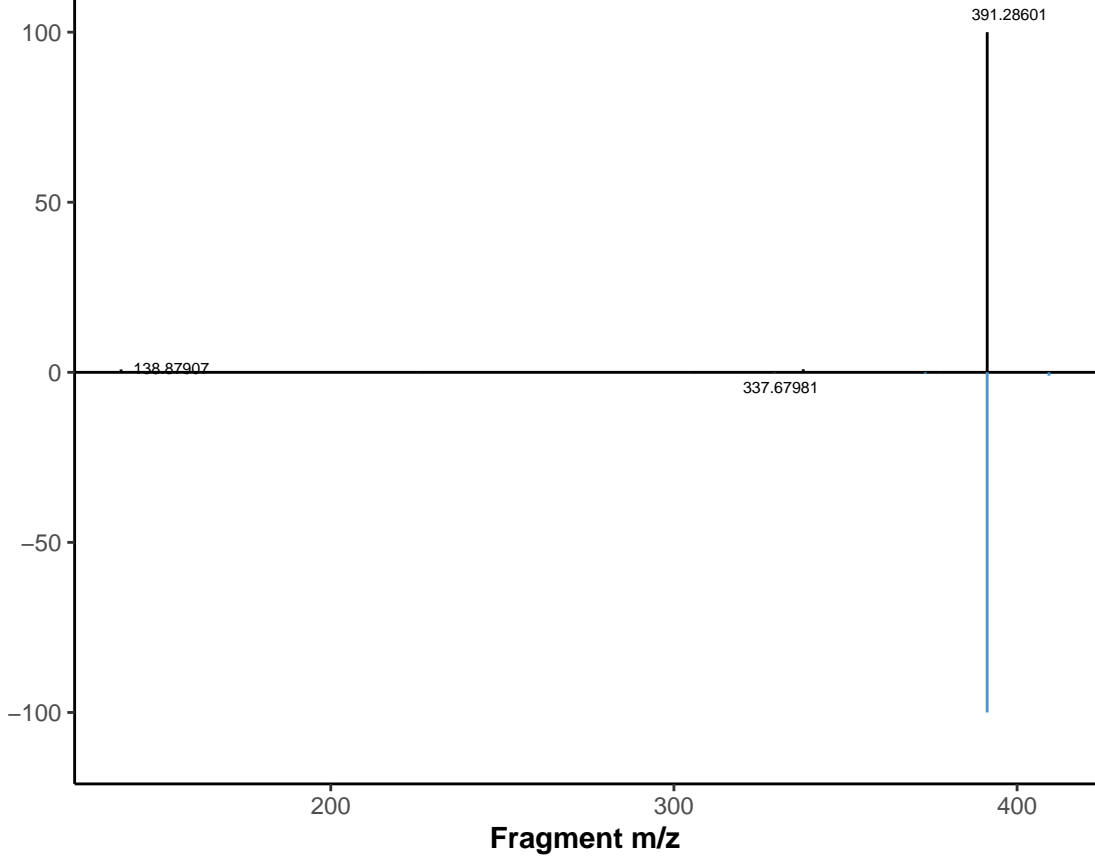

Supplement: Supplementary material 2 — MS identification chart of Xiaojin Pill ingredients. [file Data_Sheet_3.zip › Supplementary Material S2/Negative-5132.pdf]

beta-Elementonic acid

Relative intensity

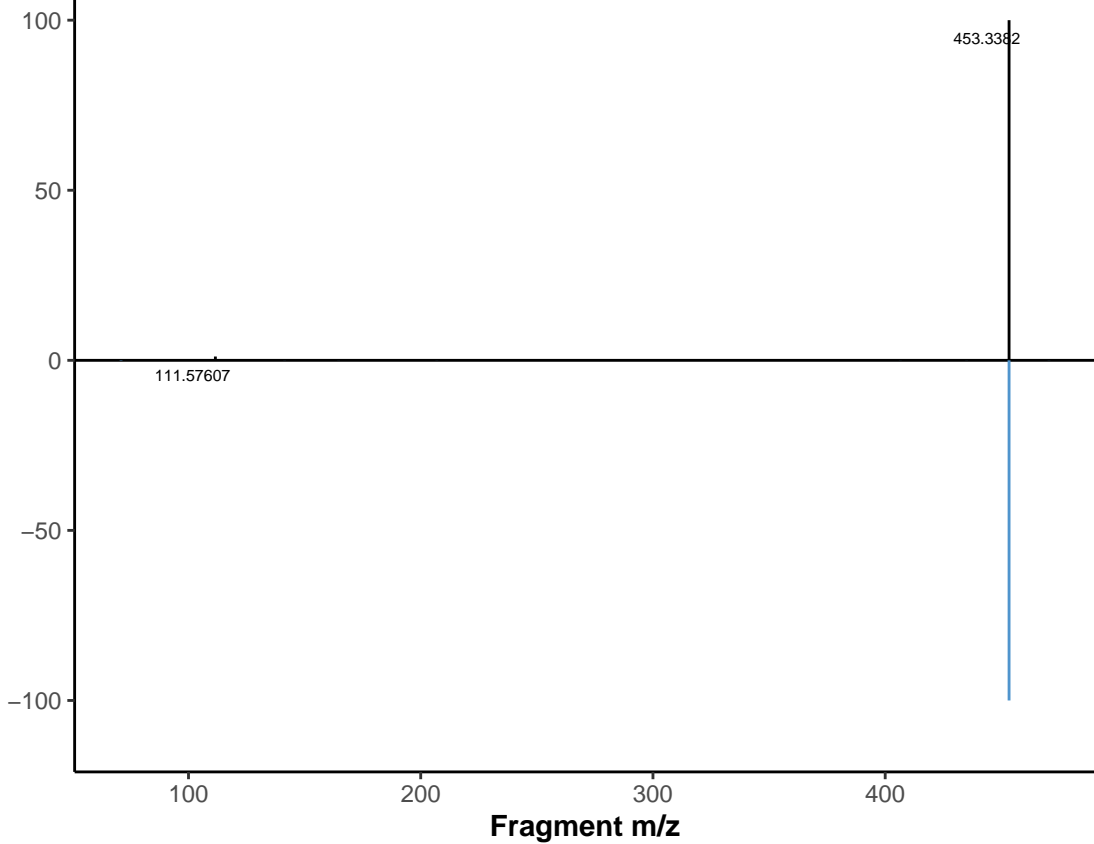

Supplement: Supplementary material 2 — MS identification chart of Xiaojin Pill ingredients. [file Data_Sheet_3.zip › Supplementary Material S2/Negative-6255.pdf]

Ursonic acid

Relative intensity

100  
50  
0  
-50  
-100

211.63155

375.26904

453.33795

Fragment m/z

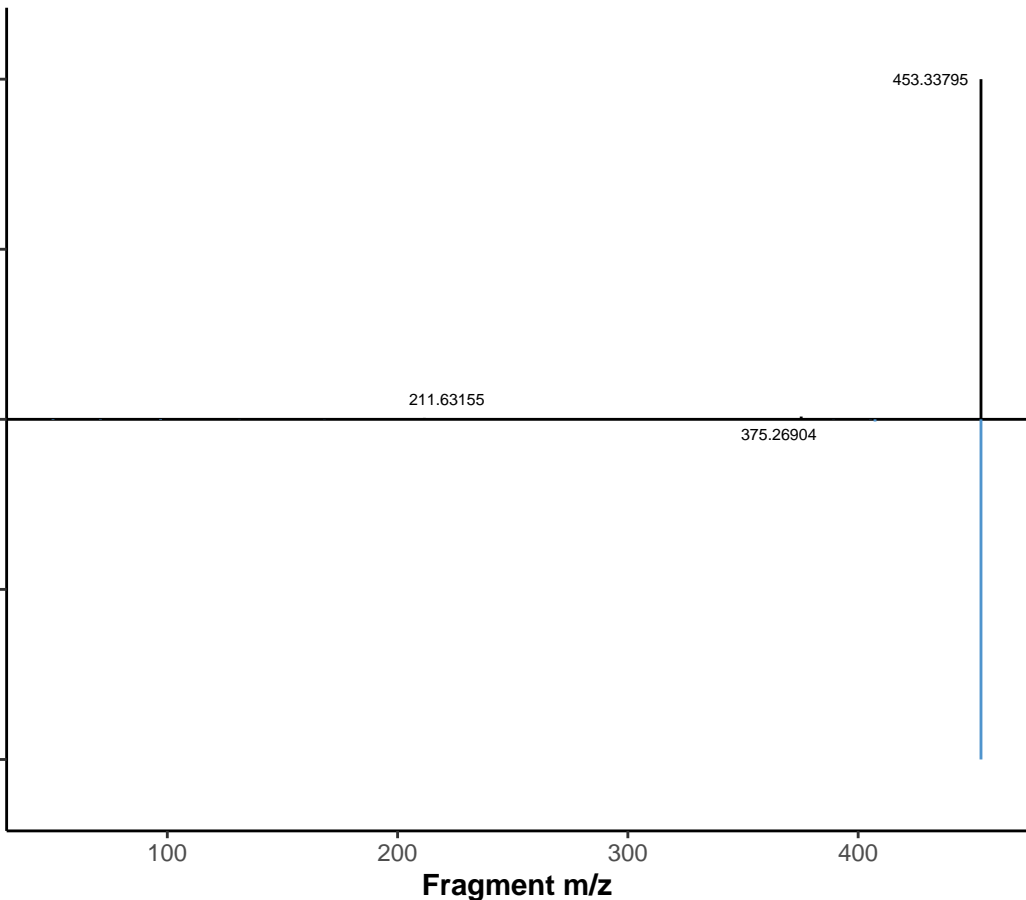

Supplement: Supplementary material 2 — MS identification chart of Xiaojin Pill ingredients. [file Data_Sheet_3.zip › Supplementary Material S2/Negative-6256.pdf]

# Vanillin

Relative intensity

100  
50  
0  
-50  
-100

80

Fragment m/z

100

120

140

89.34351

95.01402

136.01669

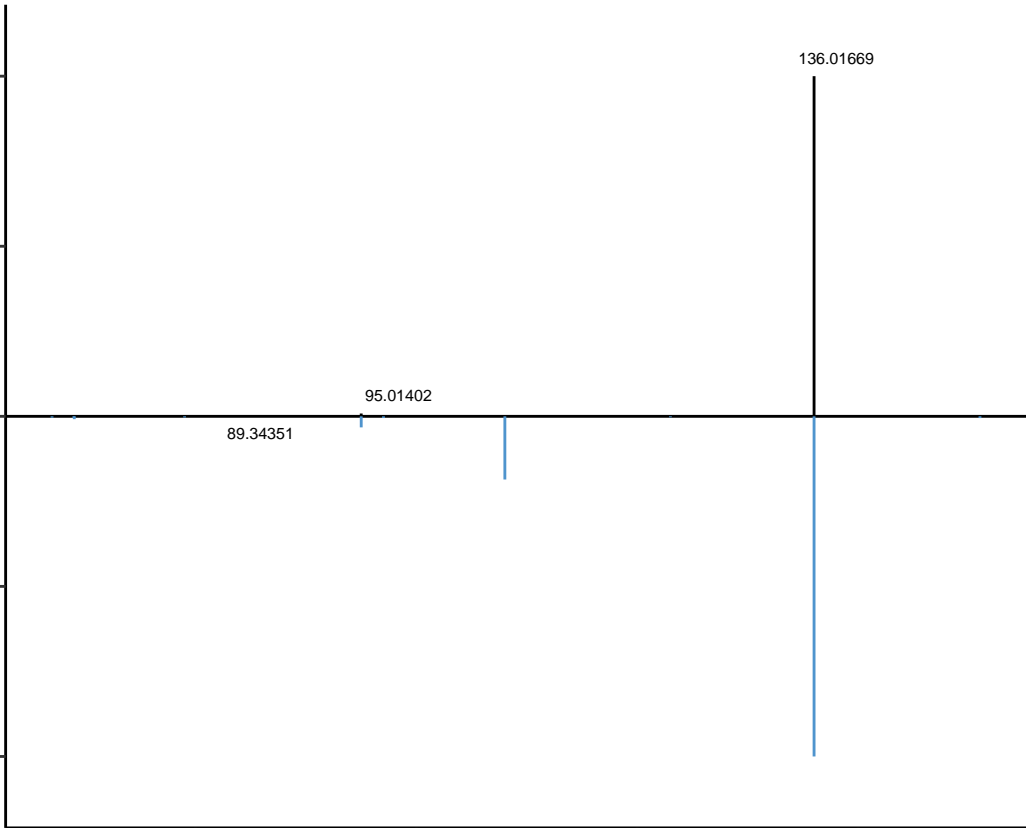

Supplement: Supplementary material 2 — MS identification chart of Xiaojin Pill ingredients. [file Data_Sheet_3.zip › Supplementary Material S2/Negative-644.pdf]

# Glabrolide

Relative intensity

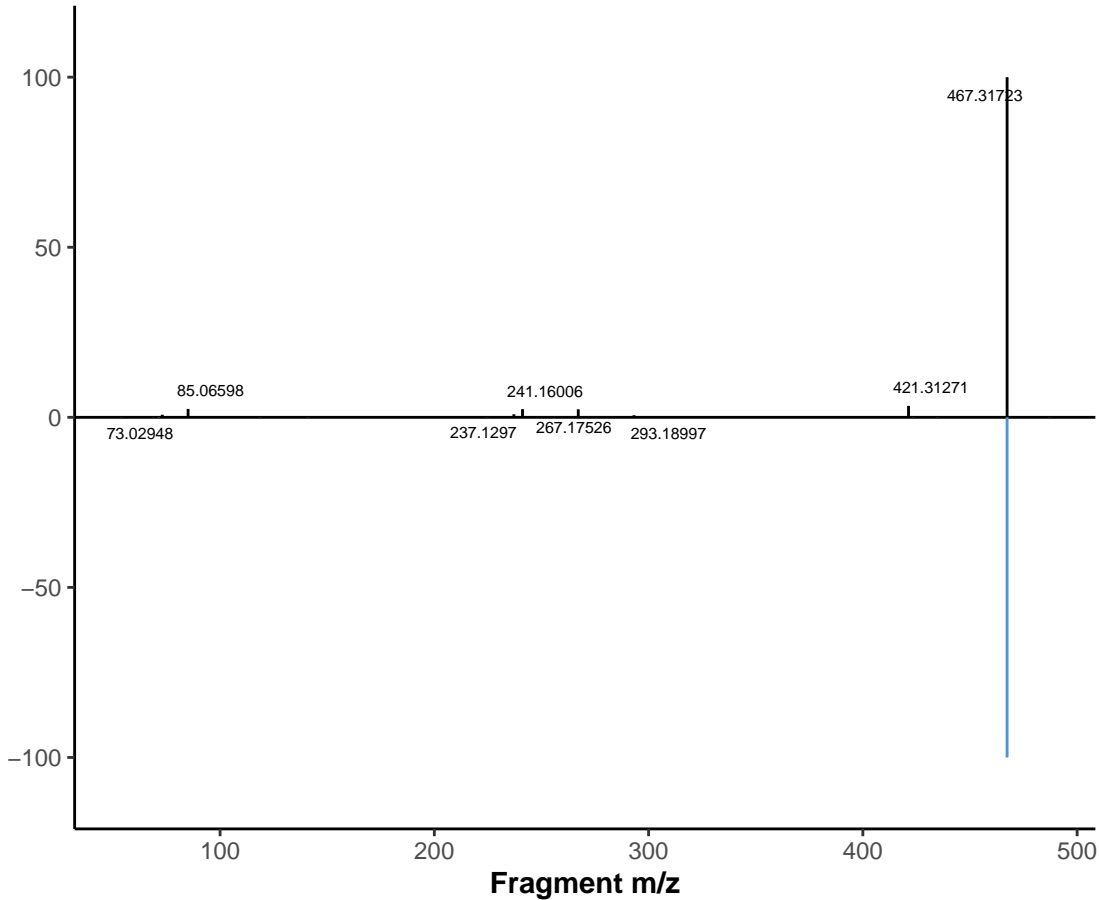

Supplement: Supplementary material 2 — MS identification chart of Xiaojin Pill ingredients. [file Data_Sheet_3.zip › Supplementary Material S2/Negative-6498.pdf]

# Echinocystic acid

Relative intensity

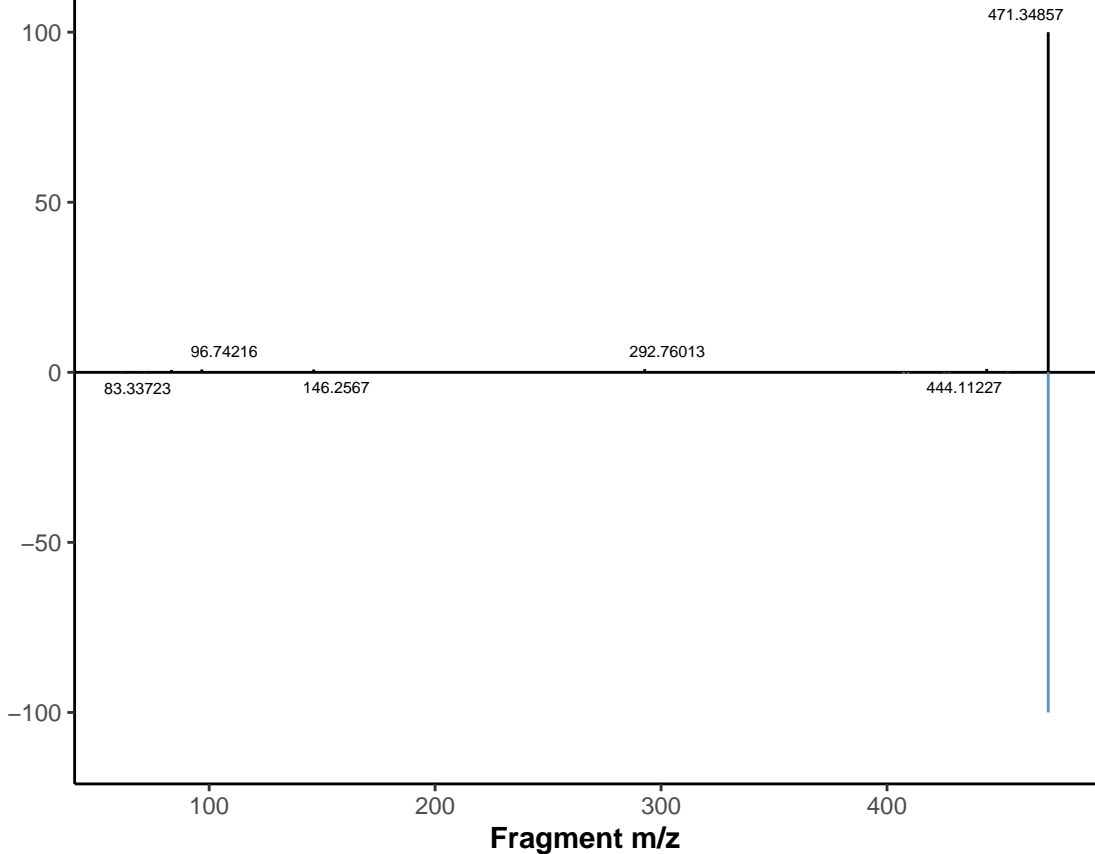

Supplement: Supplementary material 2 — MS identification chart of Xiaojin Pill ingredients. [file Data_Sheet_3.zip › Supplementary Material S2/Negative-6581.pdf]

Protocatechuic acid

Relative intensity

100  
50  
0  
-50  
-100

Fragment m/z

109.02955

65.03953

81.03465

103.0437

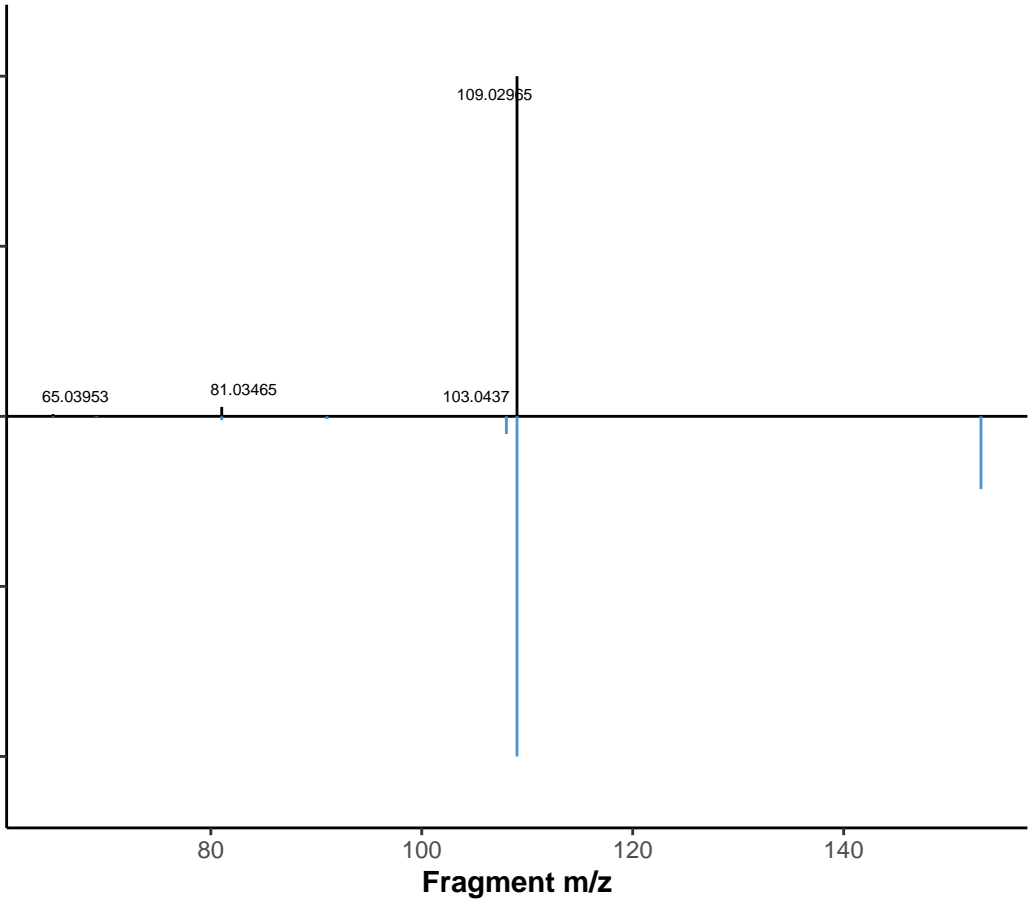

Supplement: Supplementary material 2 — MS identification chart of Xiaojin Pill ingredients. [file Data_Sheet_3.zip › Supplementary Material S2/Negative-671.pdf]

Acetyl-11-keto-beta-boswellic acid

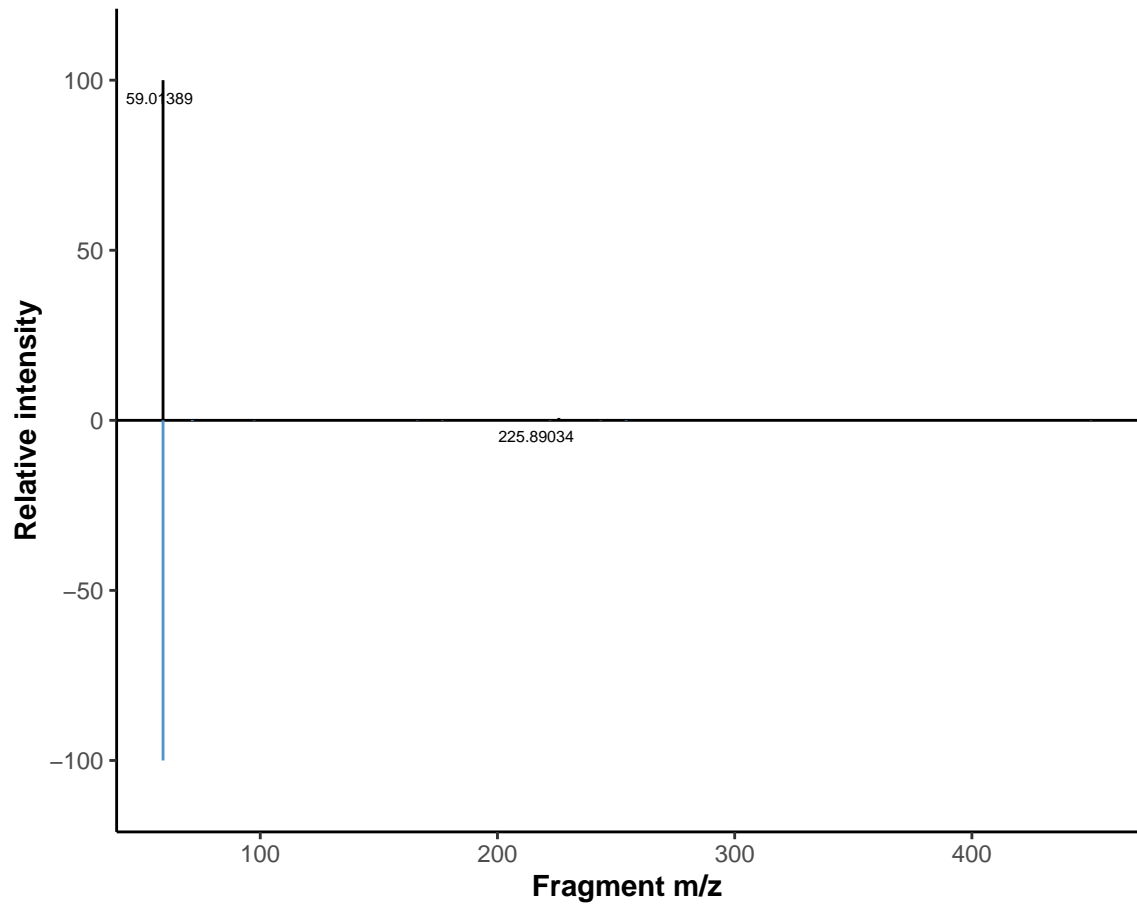

Supplement: Supplementary material 2 — MS identification chart of Xiaojin Pill ingredients. [file Data_Sheet_3.zip › Supplementary Material S2/Negative-7218.pdf]

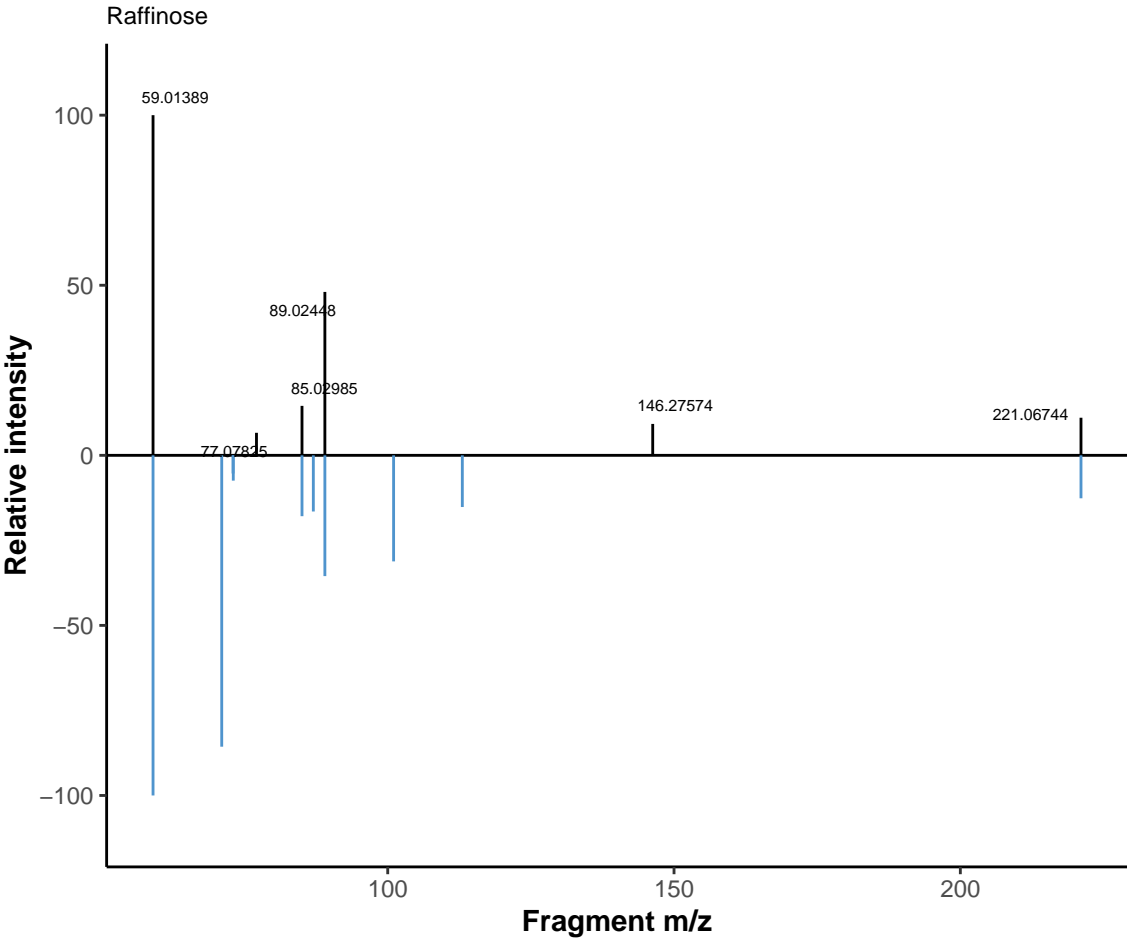

Supplement: Supplementary material 2 — MS identification chart of Xiaojin Pill ingredients. [file Data_Sheet_3.zip › Supplementary Material S2/Negative-7754.pdf]

# Curcubitacin IIa

Relative intensity

59.0139

69.03472

271.17261

359.25836

-100

Fragment m/z

100

200

300

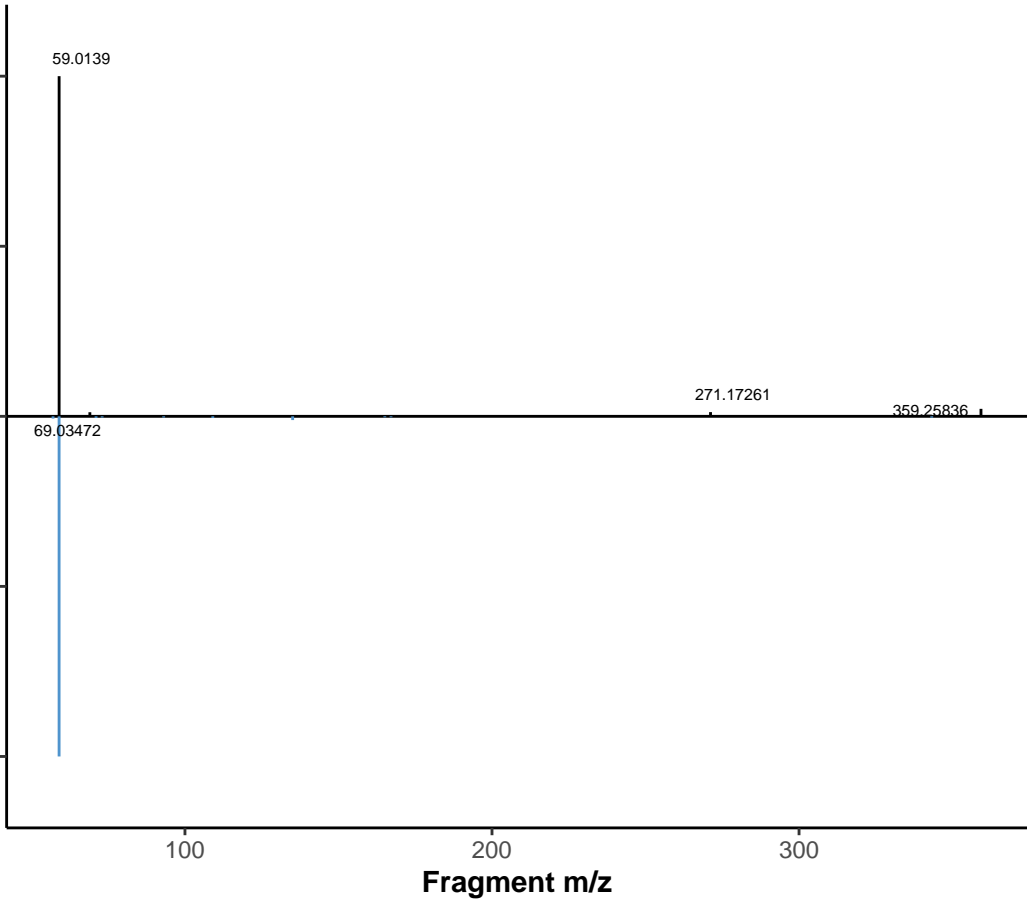

Supplement: Supplementary material 2 — MS identification chart of Xiaojin Pill ingredients. [file Data_Sheet_3.zip › Supplementary Material S2/Negative-7939.pdf]

p-Hydroxy-cinnamic acid

Relative intensity

100  
50  
0  
-50  
-100

79.93408

119.05082

90

Fragment m/z

120

150

180

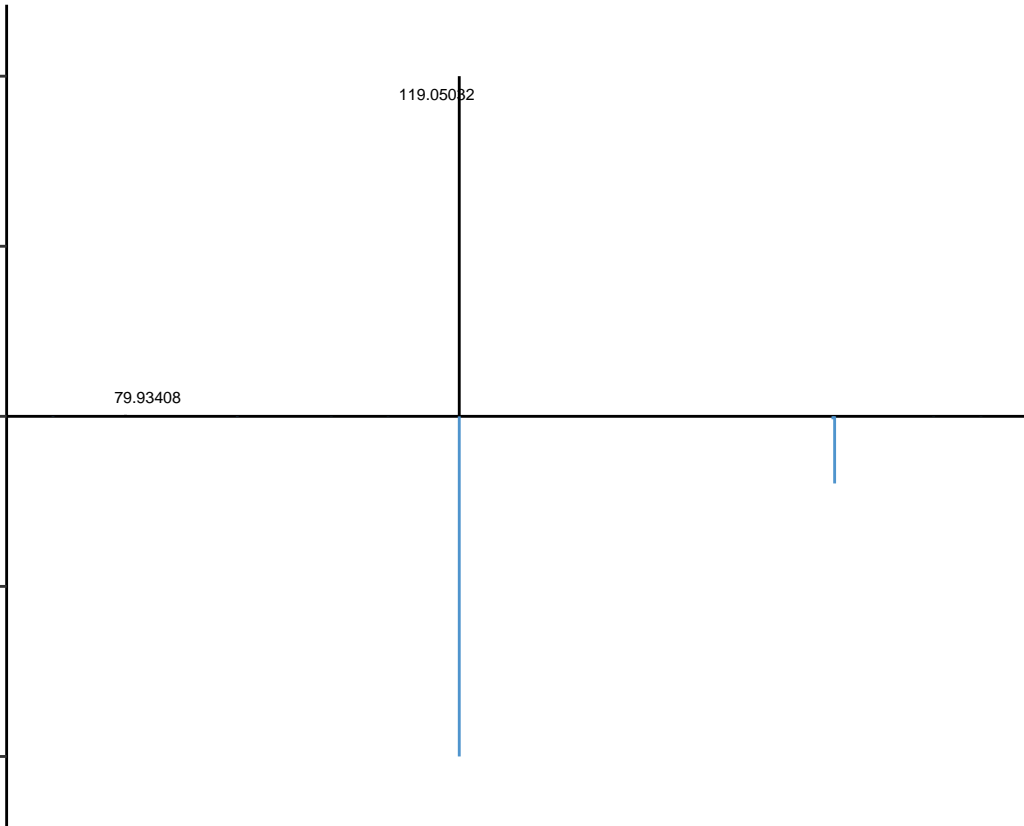

Supplement: Supplementary material 2 — MS identification chart of Xiaojin Pill ingredients. [file Data_Sheet_3.zip › Supplementary Material S2/Negative-802.pdf]

# Ferulaldehyde

Relative intensity

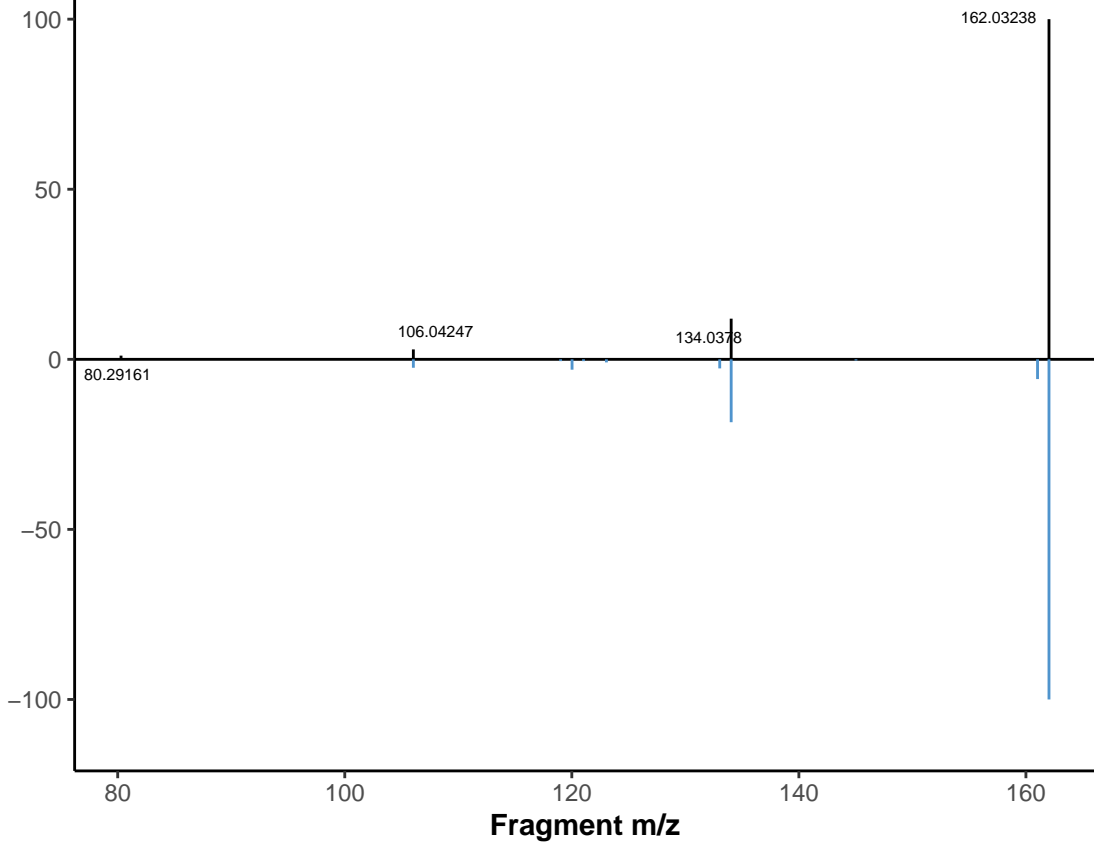

Supplement: Supplementary material 2 — MS identification chart of Xiaojin Pill ingredients. [file Data_Sheet_3.zip › Supplementary Material S2/Negative-985.pdf]

# Ligustilide

Relative intensity

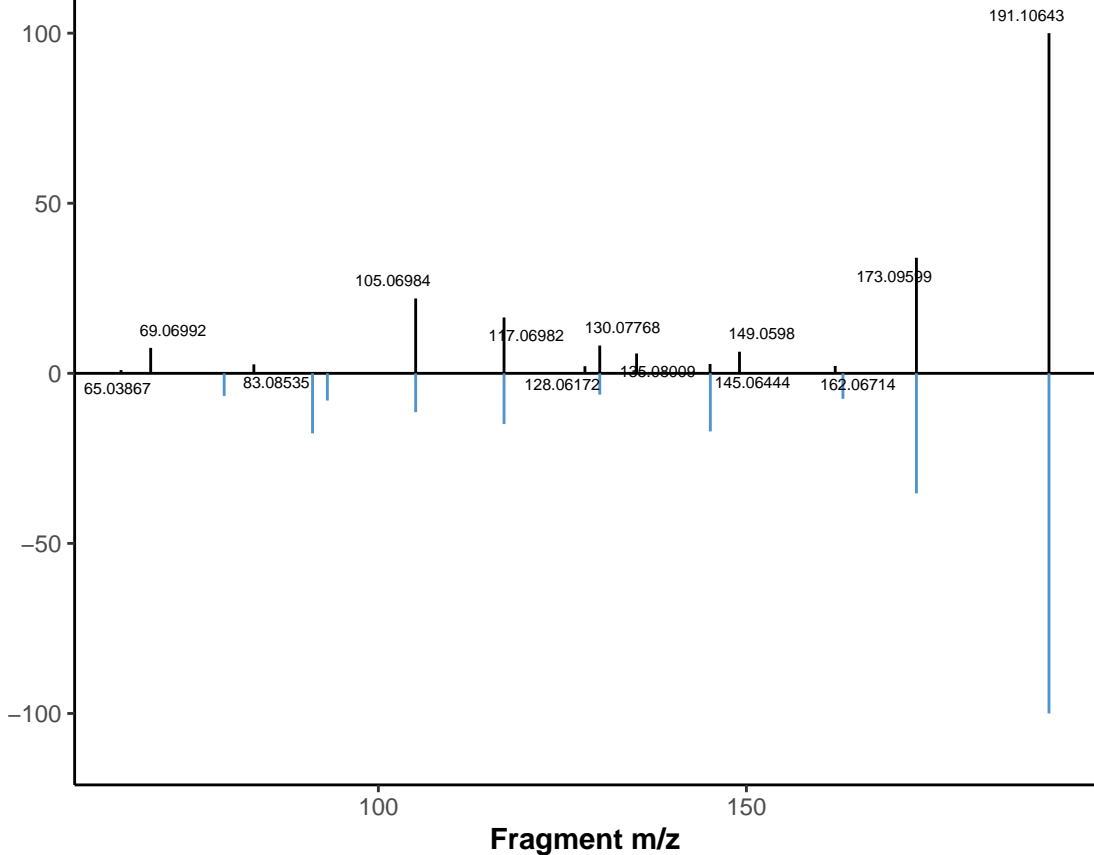

Supplement: Supplementary material 2 — MS identification chart of Xiaojin Pill ingredients. [file Data_Sheet_3.zip › Supplementary Material S2/Positive-1326.pdf]

# 3-n-Butylphthalide

Relative intensity

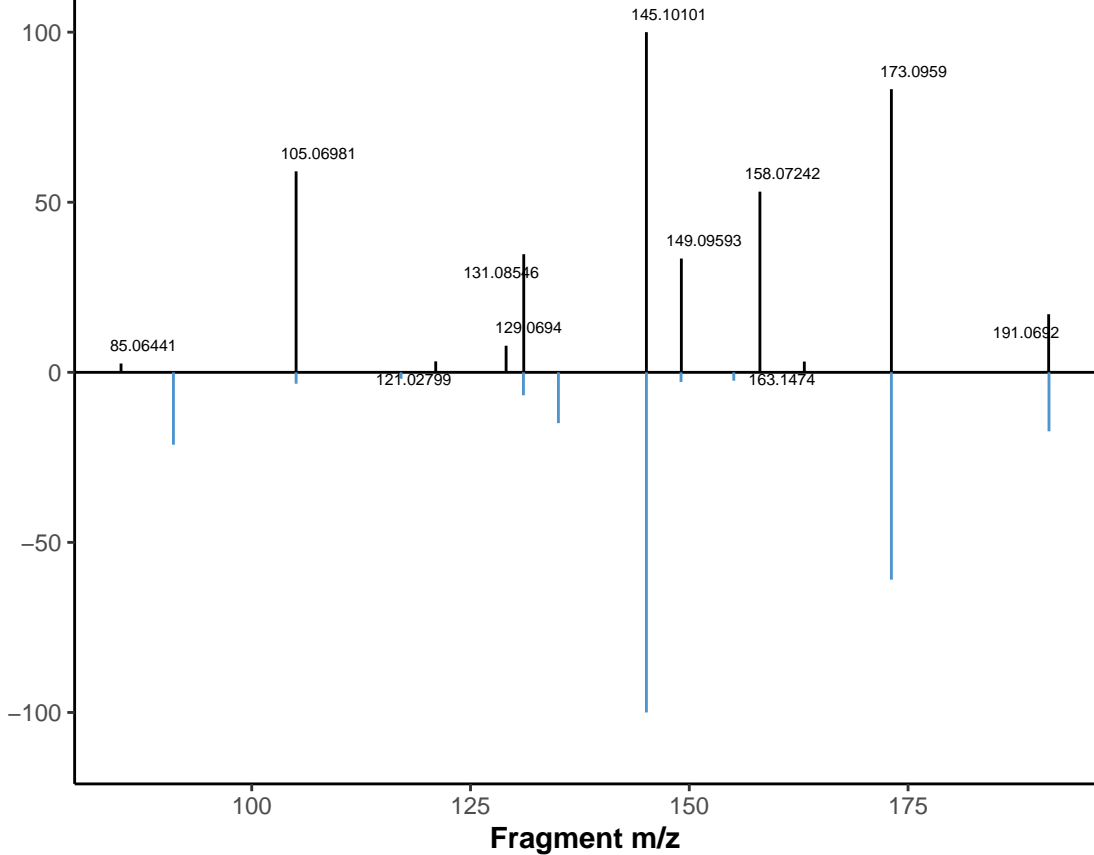

Supplement: Supplementary material 2 — MS identification chart of Xiaojin Pill ingredients. [file Data_Sheet_3.zip › Supplementary Material S2/Positive-1330.pdf]

# 4-Methyl-6,7-dihydroxycoumarin

Relative intensity

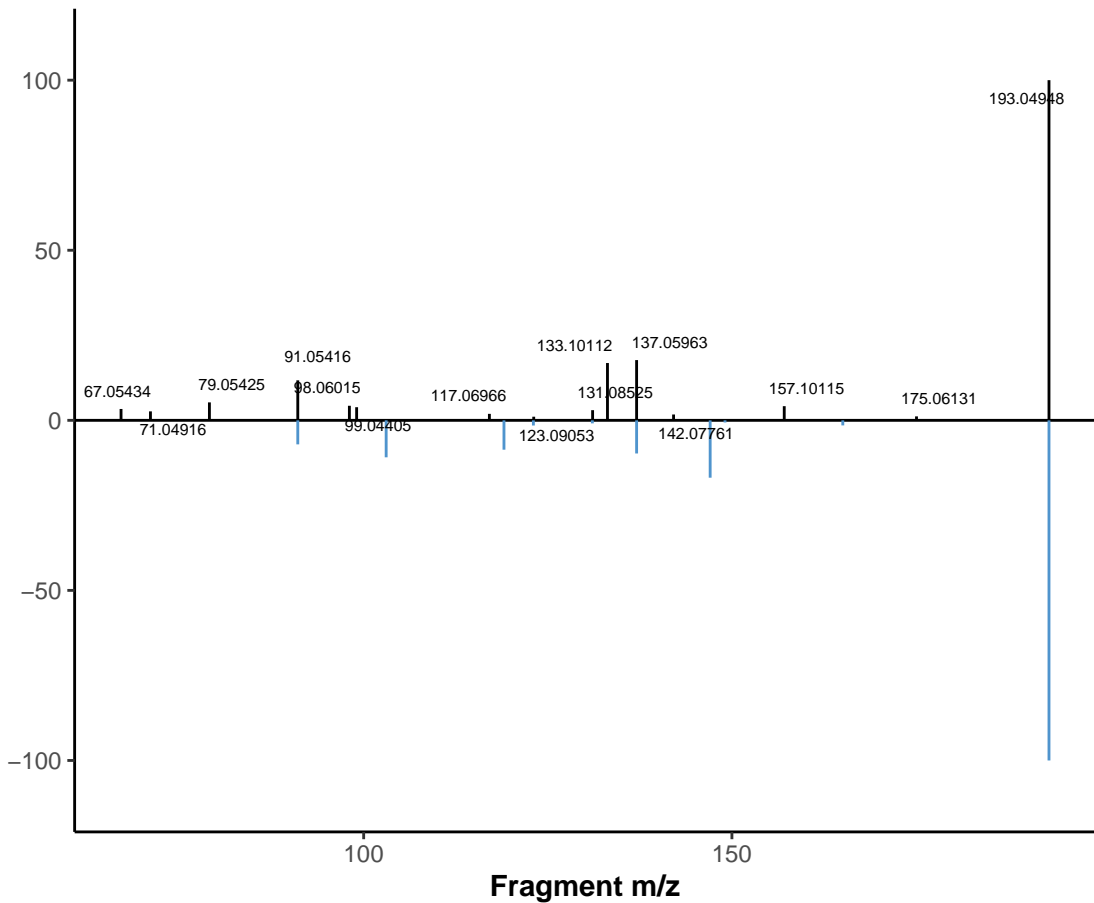

Supplement: Supplementary material 2 — MS identification chart of Xiaojin Pill ingredients. [file Data_Sheet_3.zip › Supplementary Material S2/Positive-1361.pdf]

# L-Tryptophan

Relative intensity

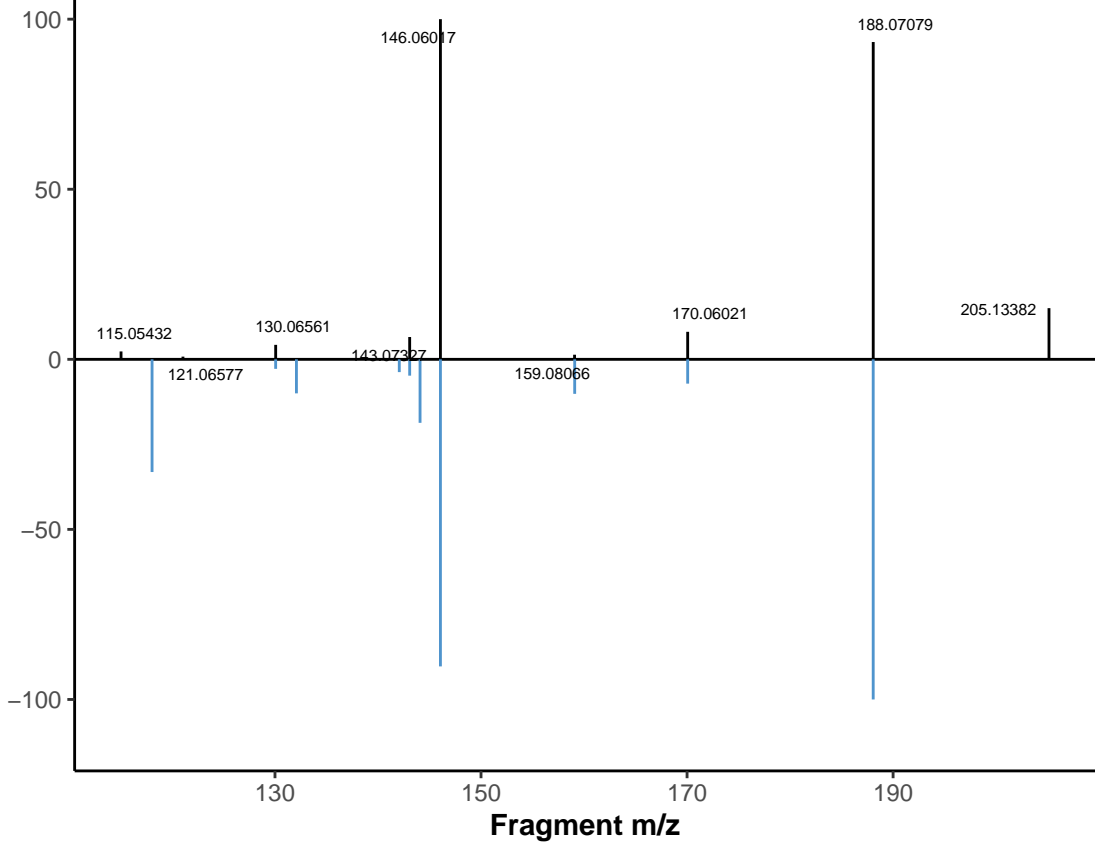

Supplement: Supplementary material 2 — MS identification chart of Xiaojin Pill ingredients. [file Data_Sheet_3.zip › Supplementary Material S2/Positive-1560.pdf]

# Citropten

Relative intensity

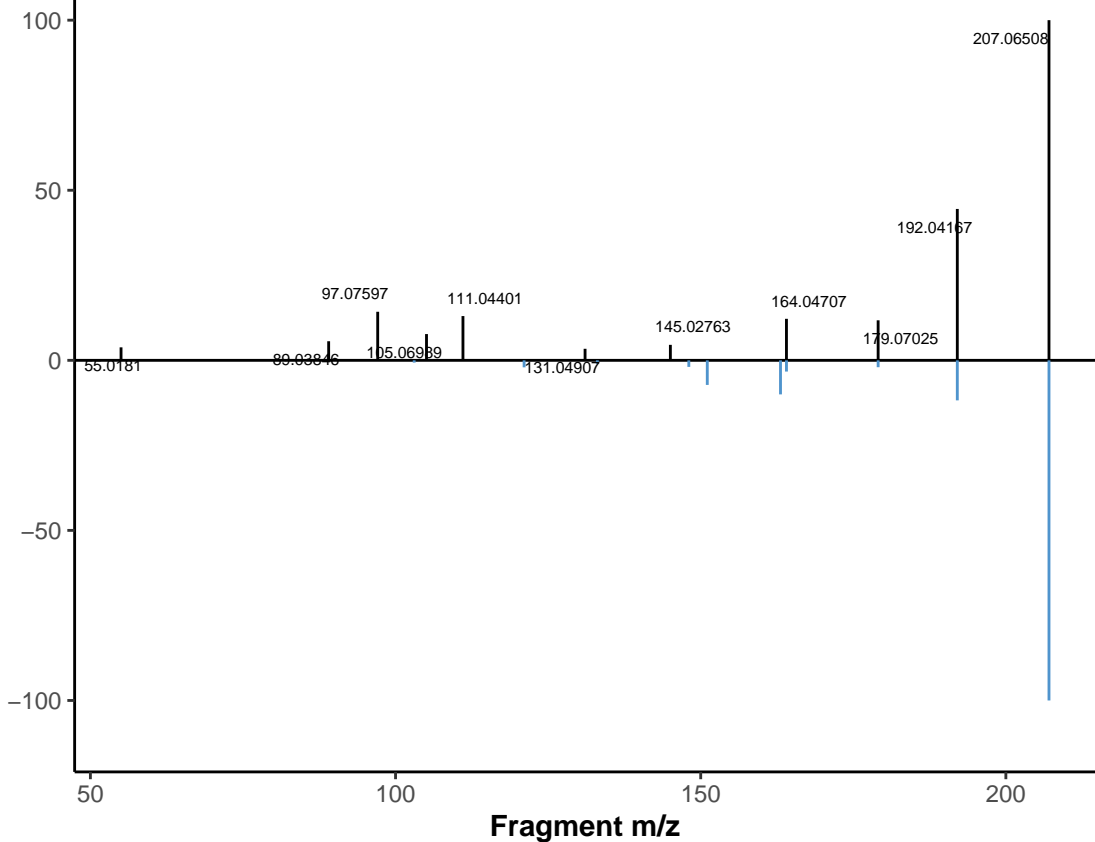

Supplement: Supplementary material 2 — MS identification chart of Xiaojin Pill ingredients. [file Data_Sheet_3.zip › Supplementary Material S2/Positive-1595.pdf]

(+)-Nootkatone

Relative intensity

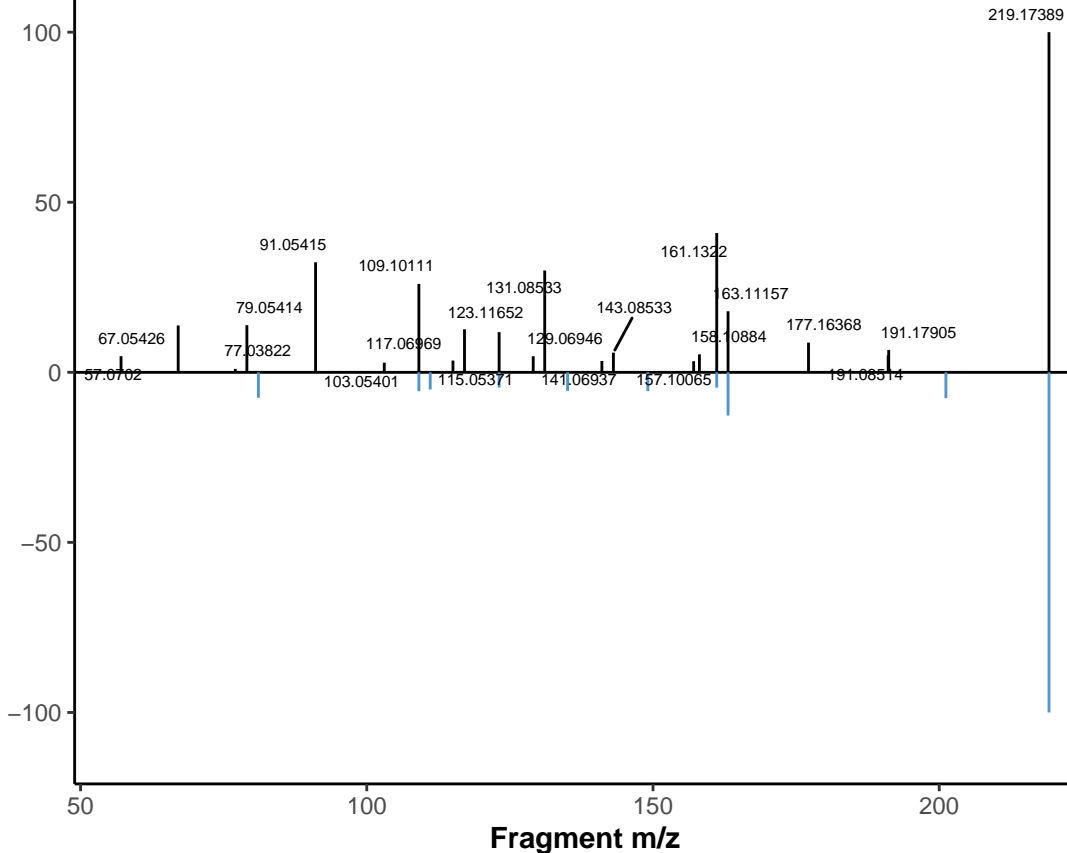

Supplement: Supplementary material 2 — MS identification chart of Xiaojin Pill ingredients. [file Data_Sheet_3.zip › Supplementary Material S2/Positive-1820.pdf]

# Germacrone

Relative intensity

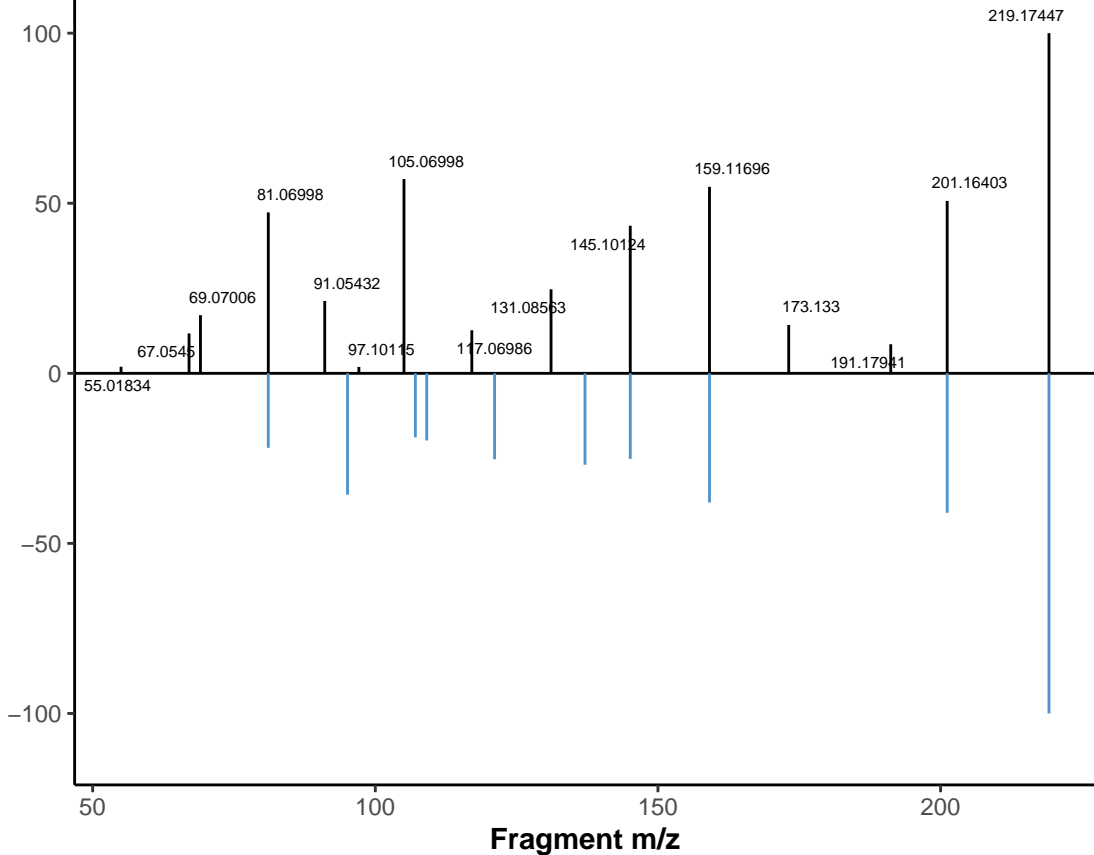

Supplement: Supplementary material 2 — MS identification chart of Xiaojin Pill ingredients. [file Data_Sheet_3.zip › Supplementary Material S2/Positive-1824.pdf]

# Atractylenolide I

Relative intensity

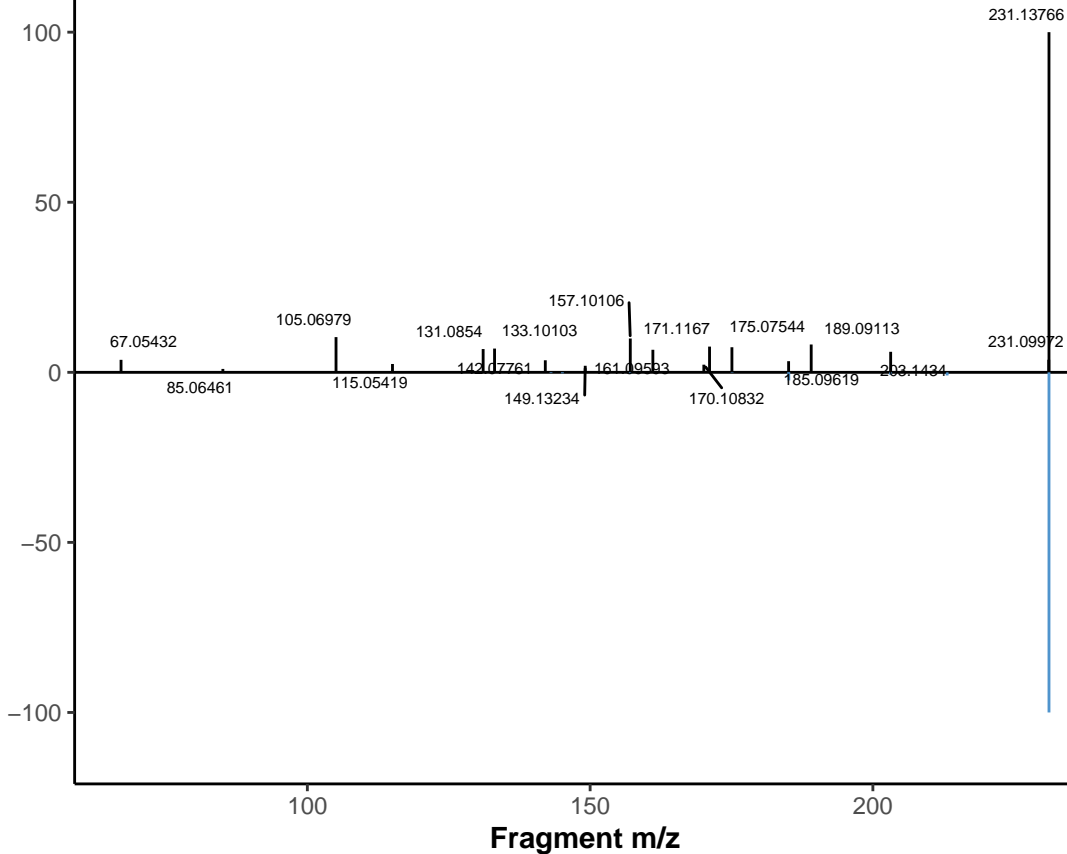

Supplement: Supplementary material 2 — MS identification chart of Xiaojin Pill ingredients. [file Data_Sheet_3.zip › Supplementary Material S2/Positive-2051.pdf]

# Lindenenol

Relative intensity

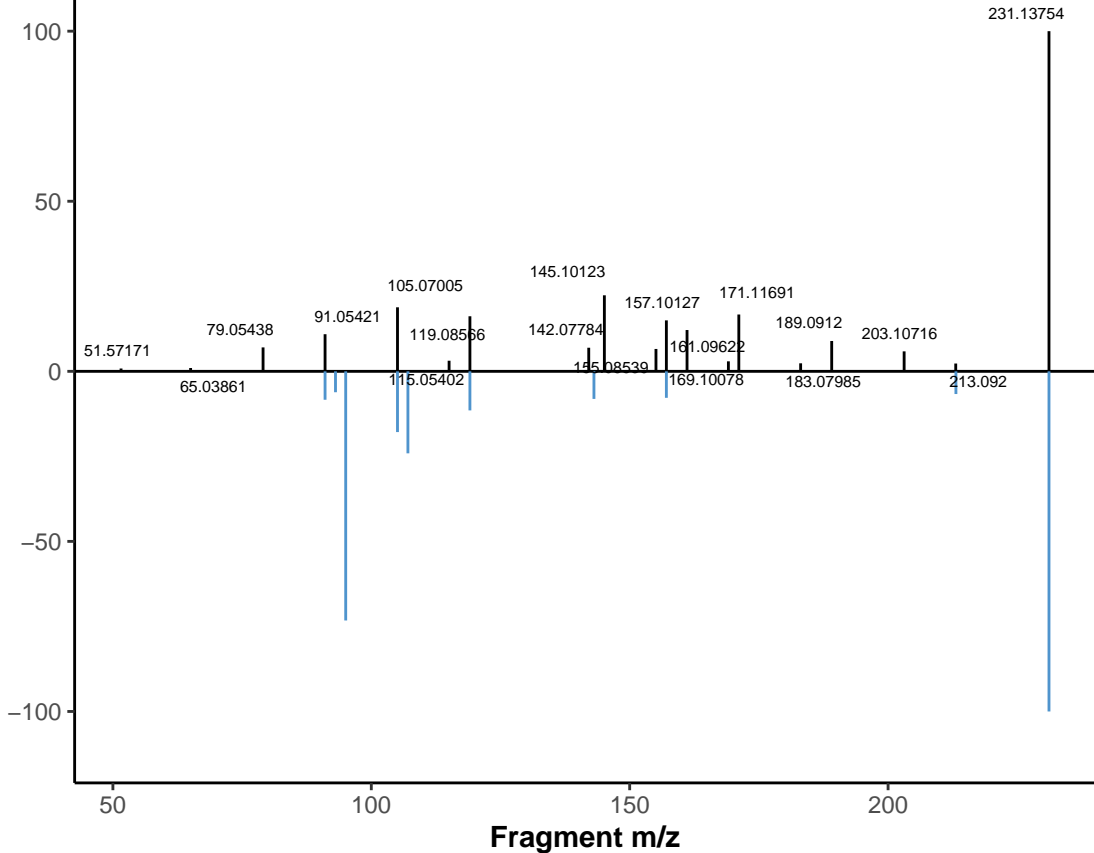

Supplement: Supplementary material 2 — MS identification chart of Xiaojin Pill ingredients. [file Data_Sheet_3.zip › Supplementary Material S2/Positive-2052.pdf]

# Atractylenolide II

Relative intensity

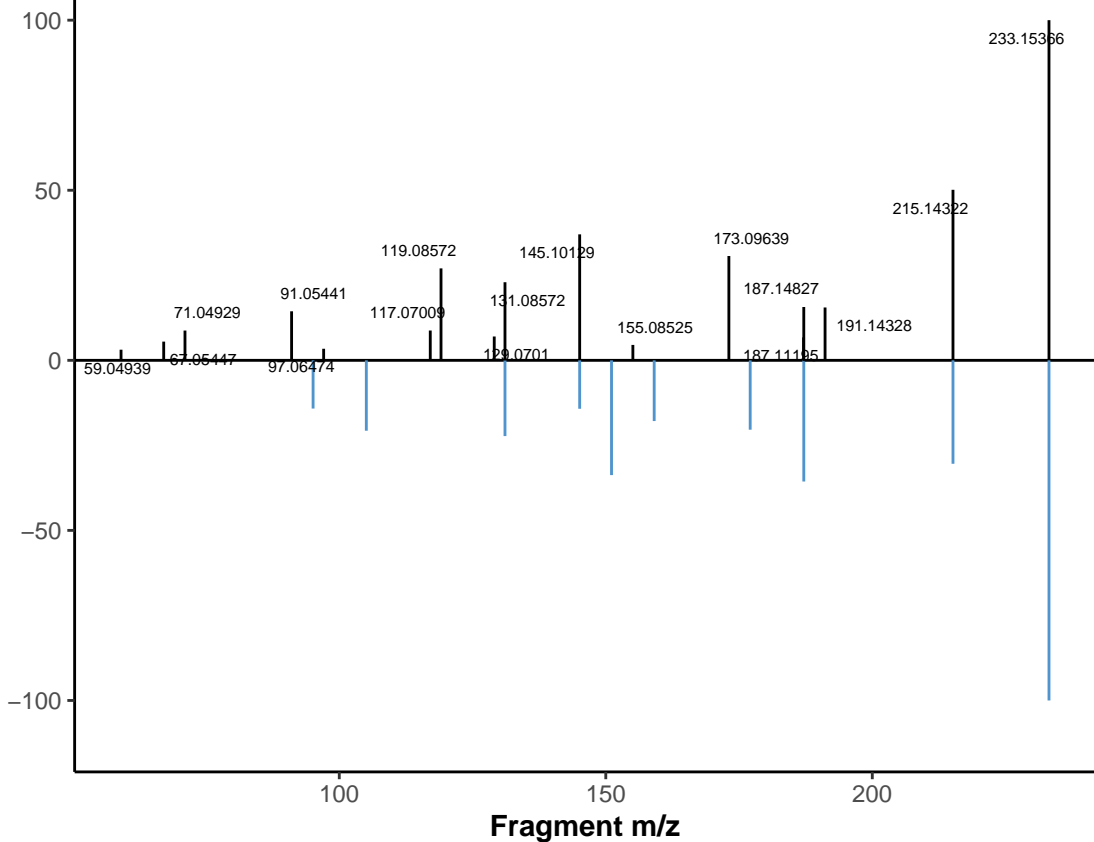

Supplement: Supplementary material 2 — MS identification chart of Xiaojin Pill ingredients. [file Data_Sheet_3.zip › Supplementary Material S2/Positive-2093.pdf]

# Isoalantolactone

Relative intensity

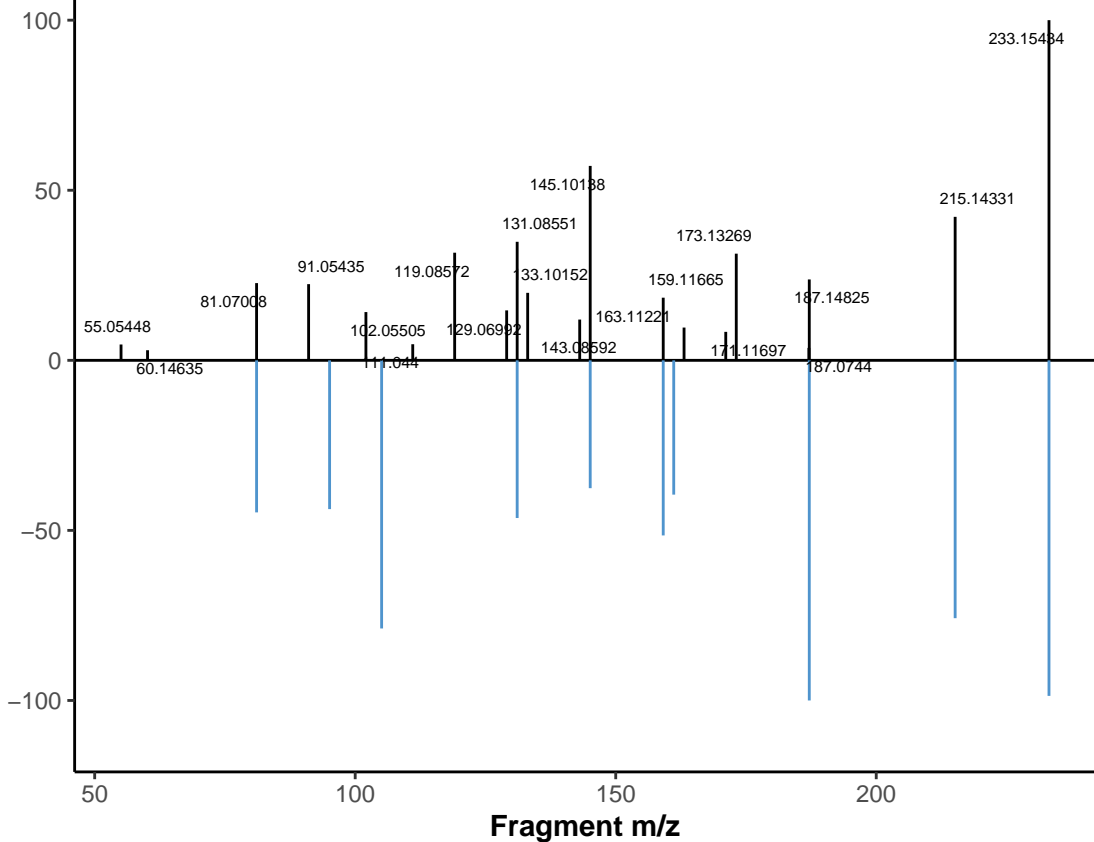

Supplement: Supplementary material 2 — MS identification chart of Xiaojin Pill ingredients. [file Data_Sheet_3.zip › Supplementary Material S2/Positive-2096.pdf]

## Curcumenol

Relative intensity

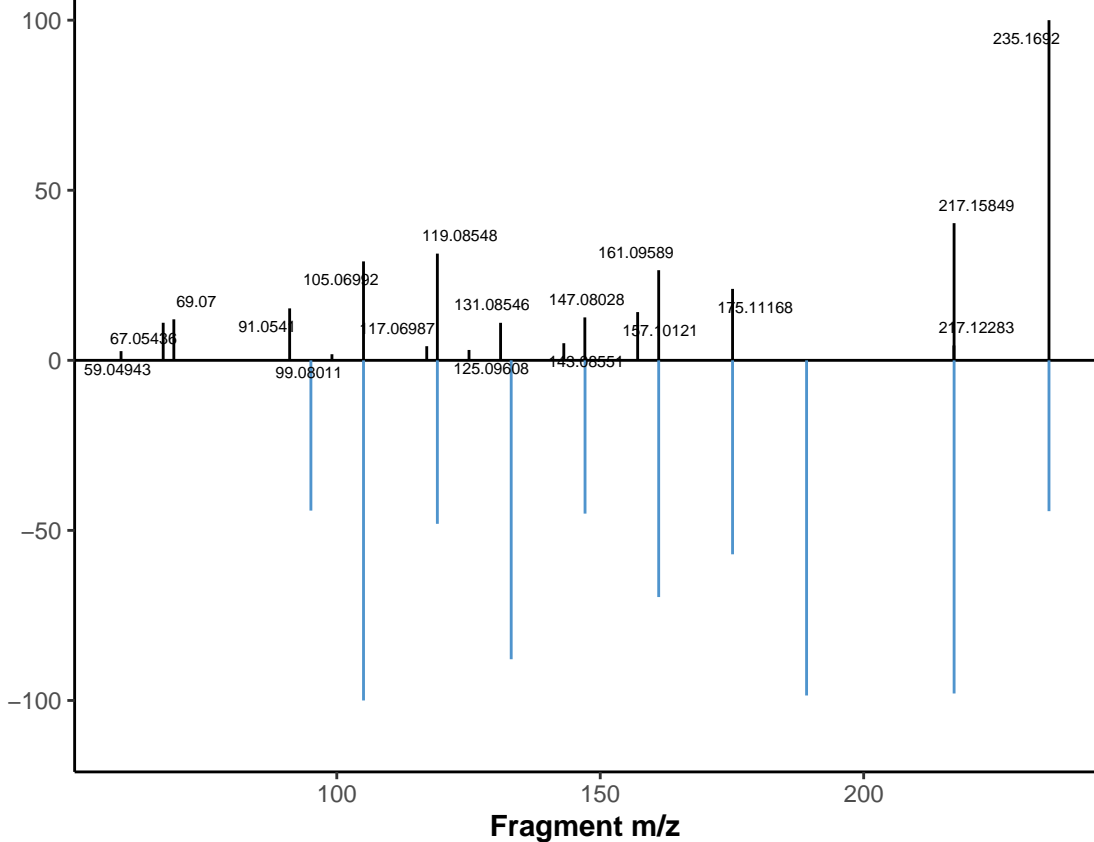

Supplement: Supplementary material 2 — MS identification chart of Xiaojin Pill ingredients. [file Data_Sheet_3.zip › Supplementary Material S2/Positive-2136.pdf]

## Curcumol

Relative intensity

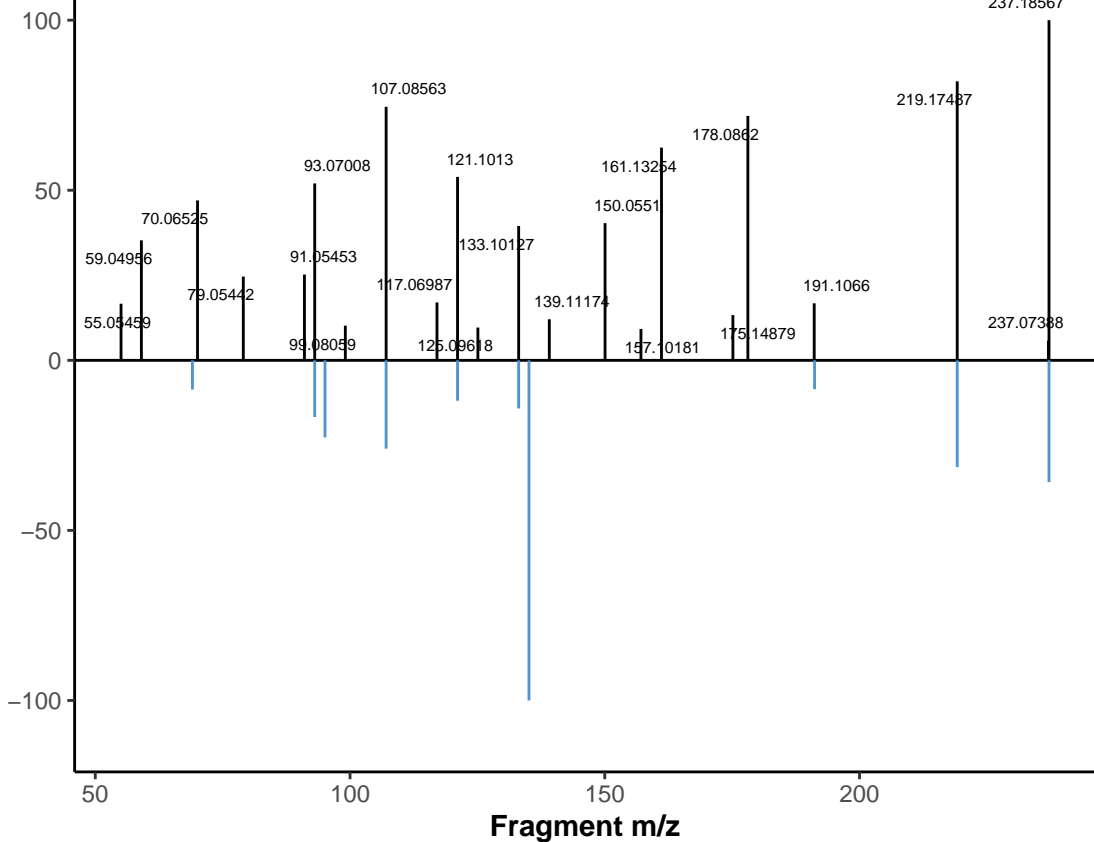

Supplement: Supplementary material 2 — MS identification chart of Xiaojin Pill ingredients. [file Data_Sheet_3.zip › Supplementary Material S2/Positive-2163.pdf]

# Linderalactone

Relative intensity

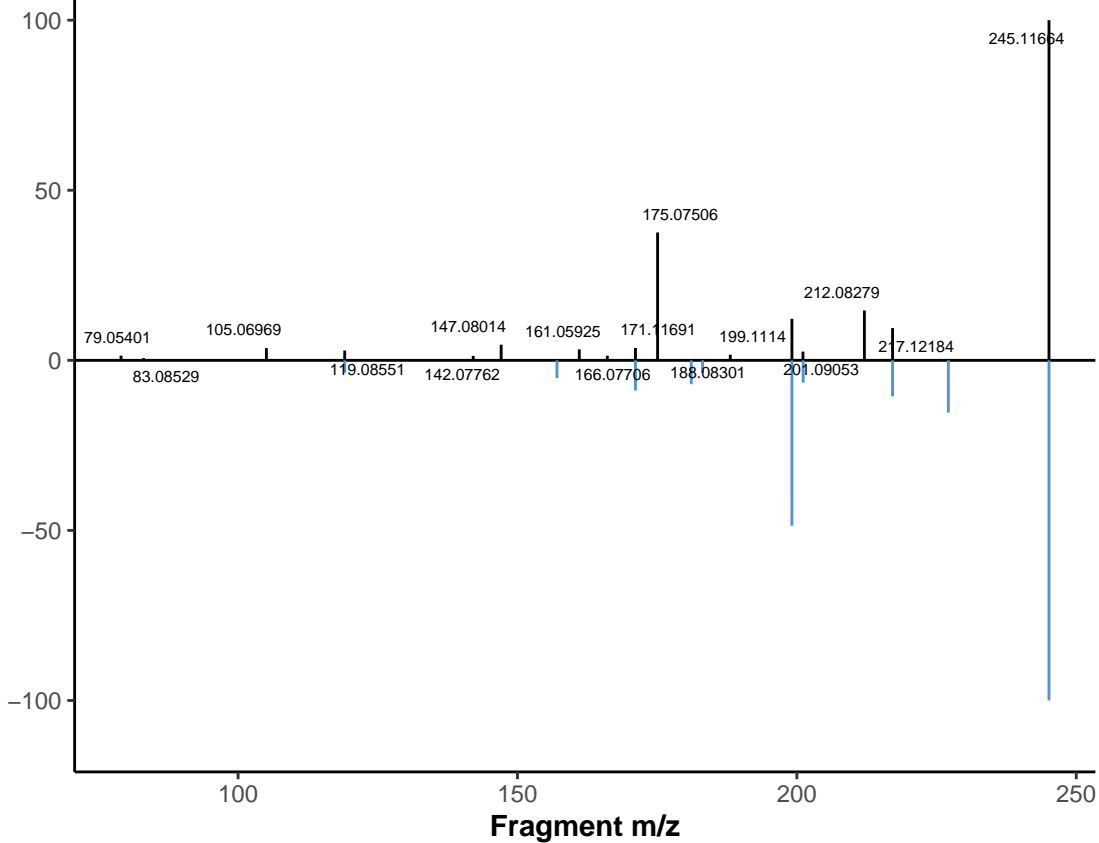

Supplement: Supplementary material 2 — MS identification chart of Xiaojin Pill ingredients. [file Data_Sheet_3.zip › Supplementary Material S2/Positive-2322.pdf]

# Suberosin

Relative intensity

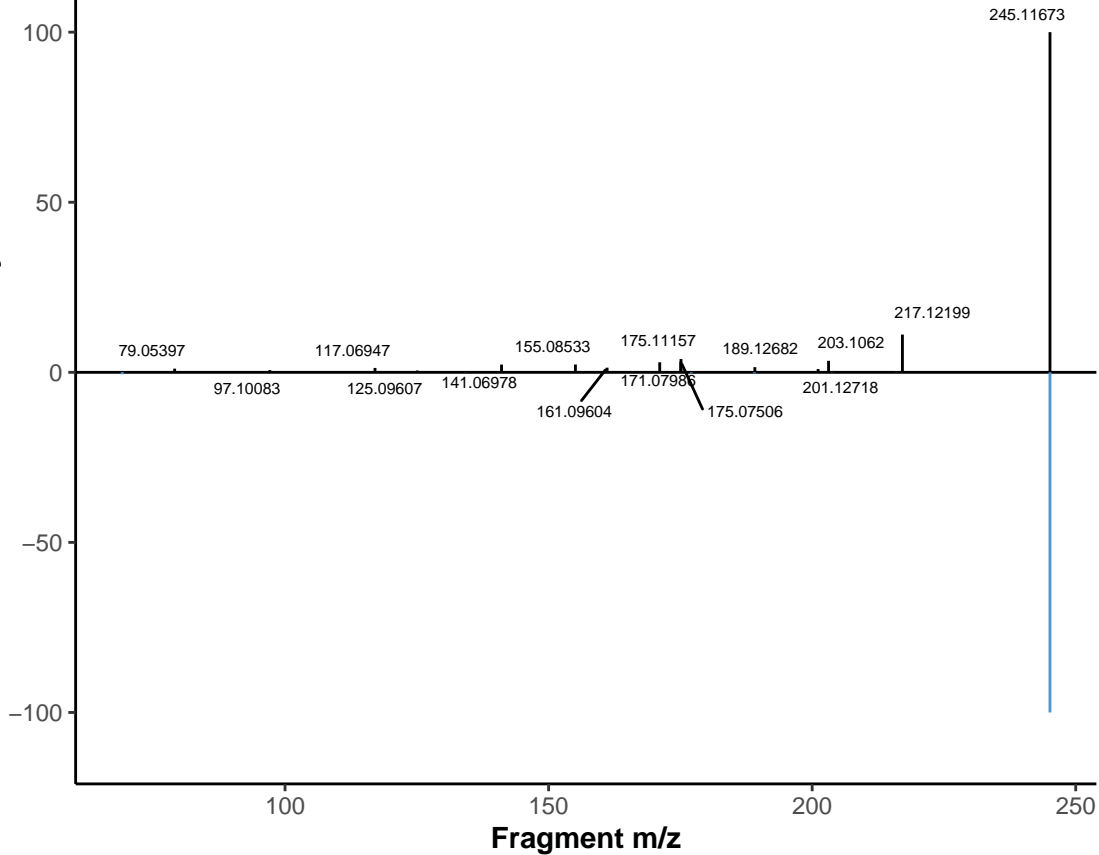

Supplement: Supplementary material 2 — MS identification chart of Xiaojin Pill ingredients. [file Data_Sheet_3.zip › Supplementary Material S2/Positive-2325.pdf]

# Isopimpinellin

Relative intensity

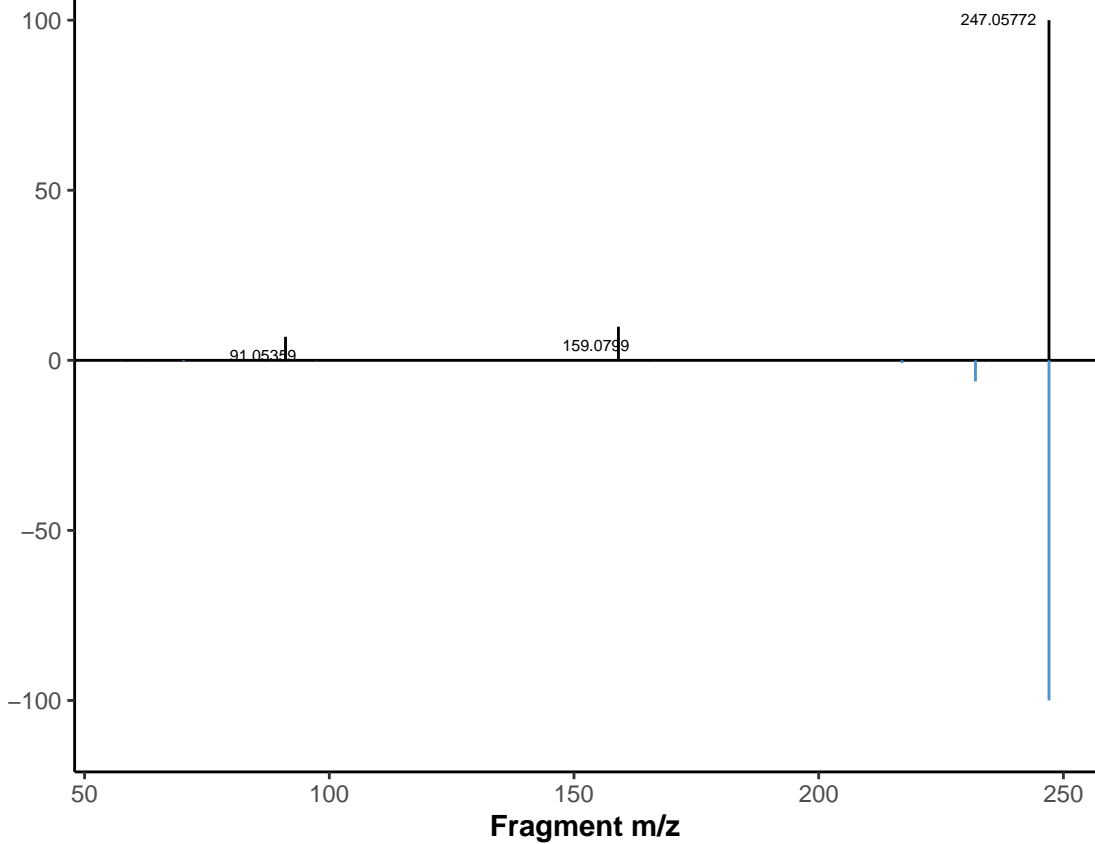

Supplement: Supplementary material 2 — MS identification chart of Xiaojin Pill ingredients. [file Data_Sheet_3.zip › Supplementary Material S2/Positive-2351.pdf]

# Parthenolide

Relative intensity

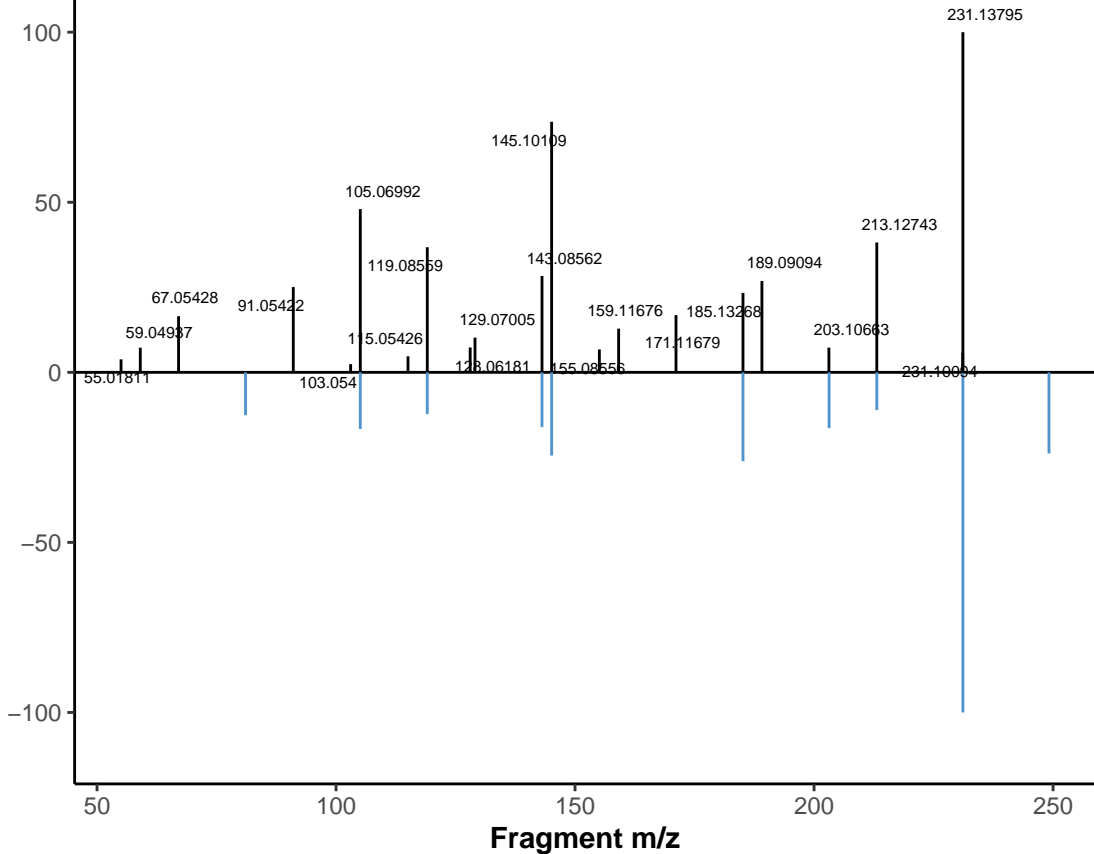

Supplement: Supplementary material 2 — MS identification chart of Xiaojin Pill ingredients. [file Data_Sheet_3.zip › Supplementary Material S2/Positive-2405.pdf]

# Cytosine

Relative intensity

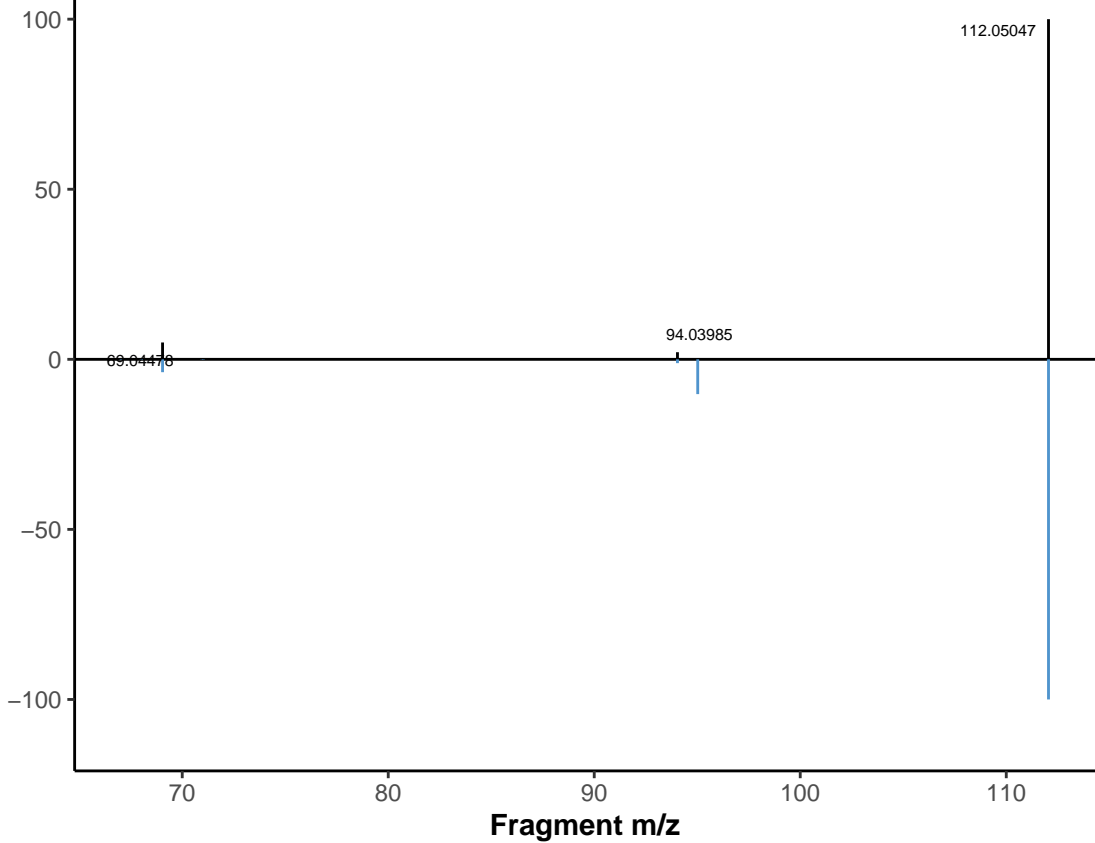

Supplement: Supplementary material 2 — MS identification chart of Xiaojin Pill ingredients. [file Data_Sheet_3.zip › Supplementary Material S2/Positive-255.pdf]

# Huperzine B

Relative intensity

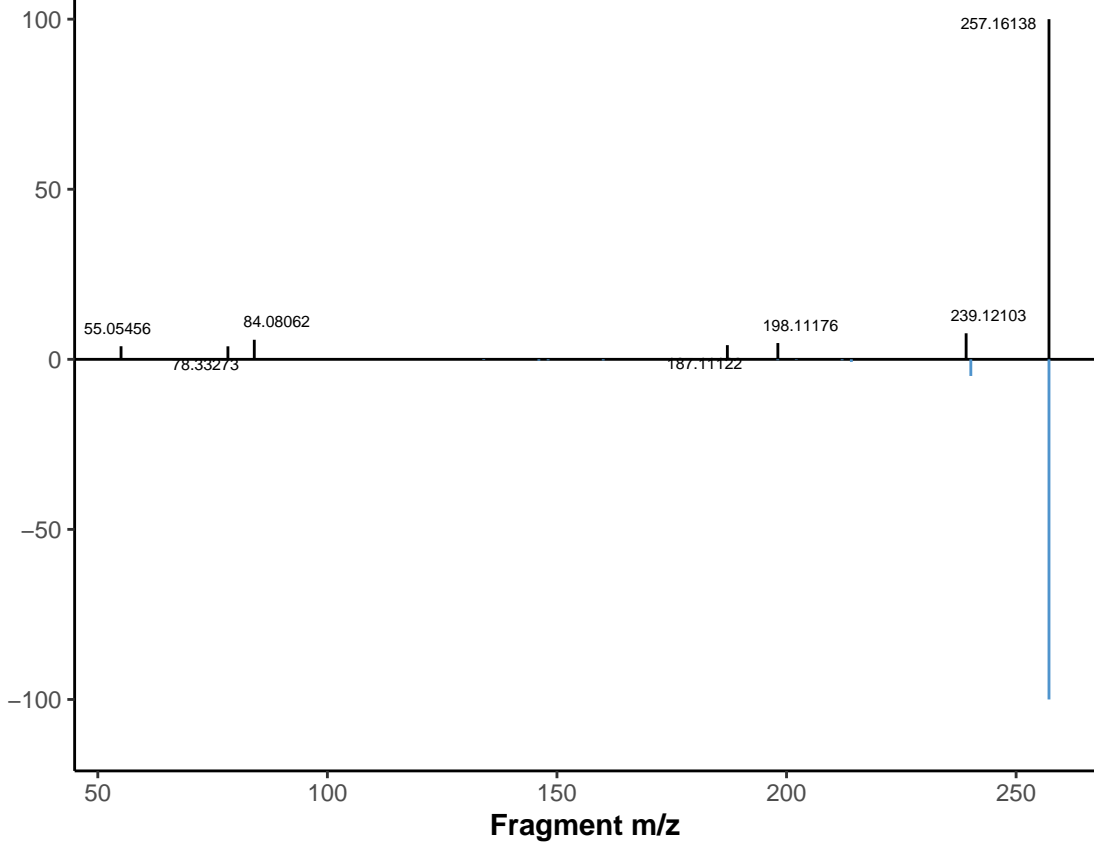

Supplement: Supplementary material 2 — MS identification chart of Xiaojin Pill ingredients. [file Data_Sheet_3.zip › Supplementary Material S2/Positive-2555.pdf]

# Linderane

Relative intensity

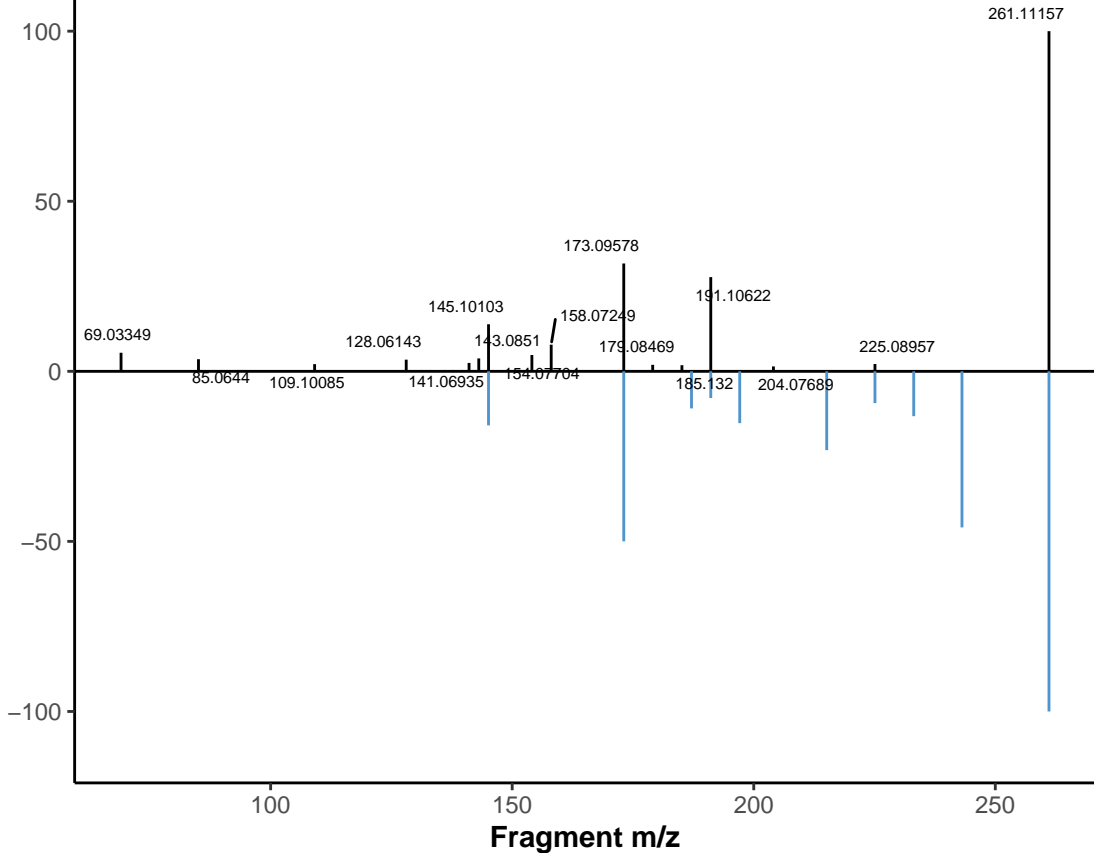

Supplement: Supplementary material 2 — MS identification chart of Xiaojin Pill ingredients. [file Data_Sheet_3.zip › Supplementary Material S2/Positive-2623.pdf]

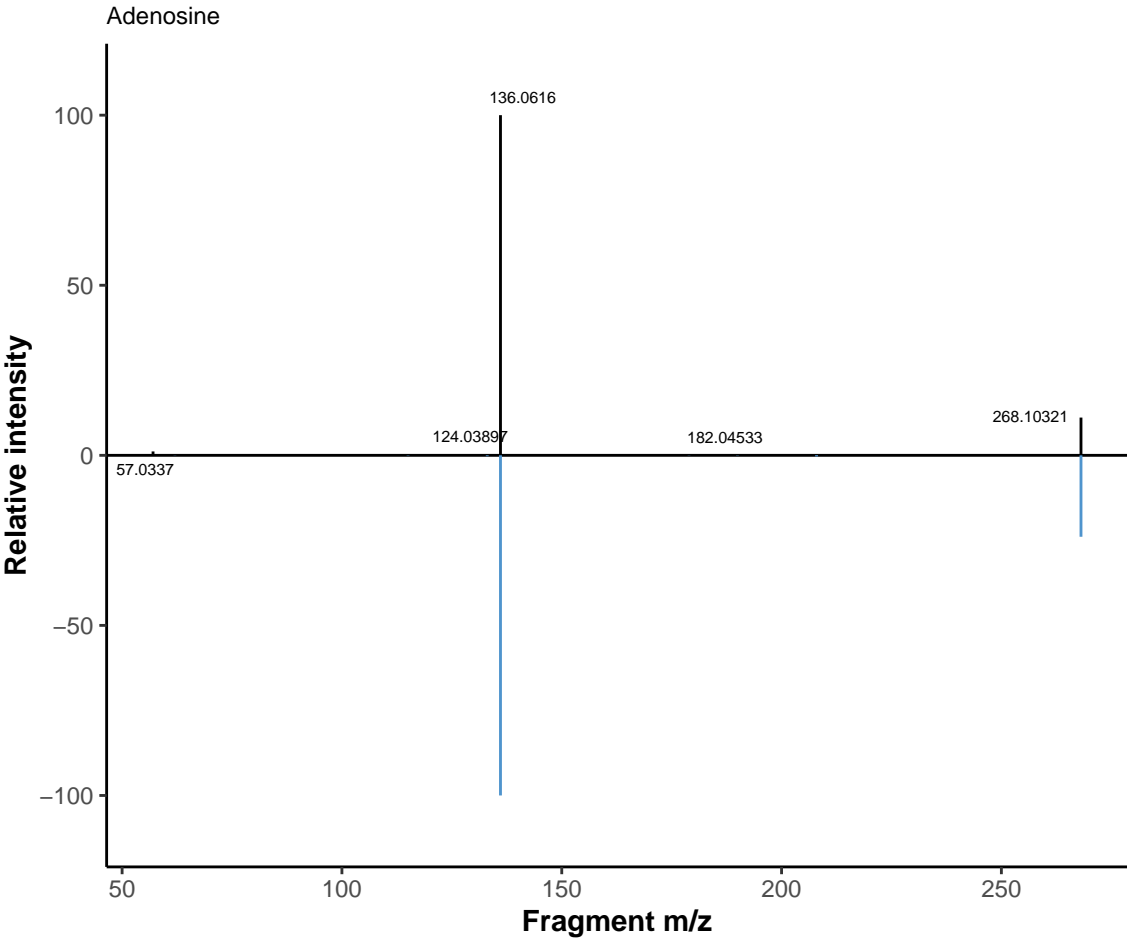

Supplement: Supplementary material 2 — MS identification chart of Xiaojin Pill ingredients. [file Data_Sheet_3.zip › Supplementary Material S2/Positive-2749.pdf]

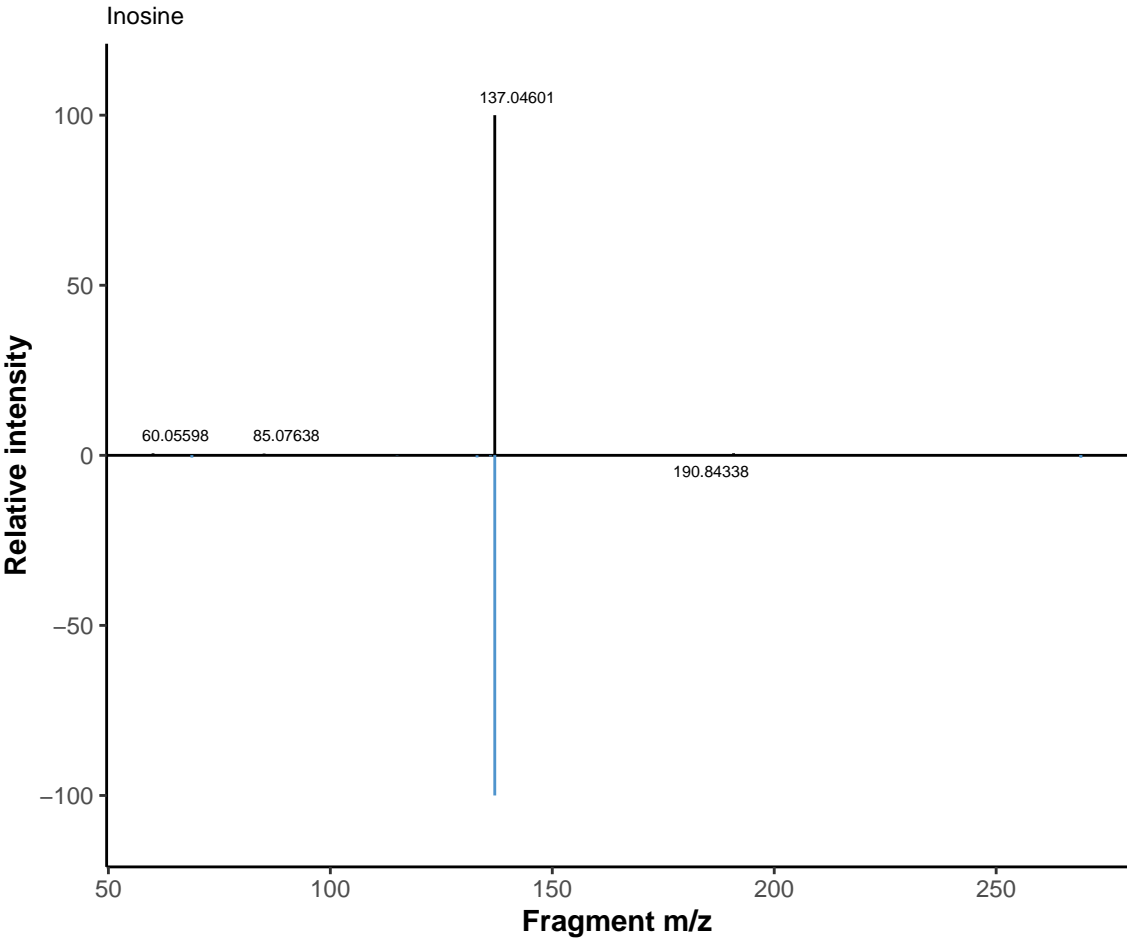

Supplement: Supplementary material 2 — MS identification chart of Xiaojin Pill ingredients. [file Data_Sheet_3.zip › Supplementary Material S2/Positive-2763.pdf]

2-Pyrrolidinecarboxylic acid

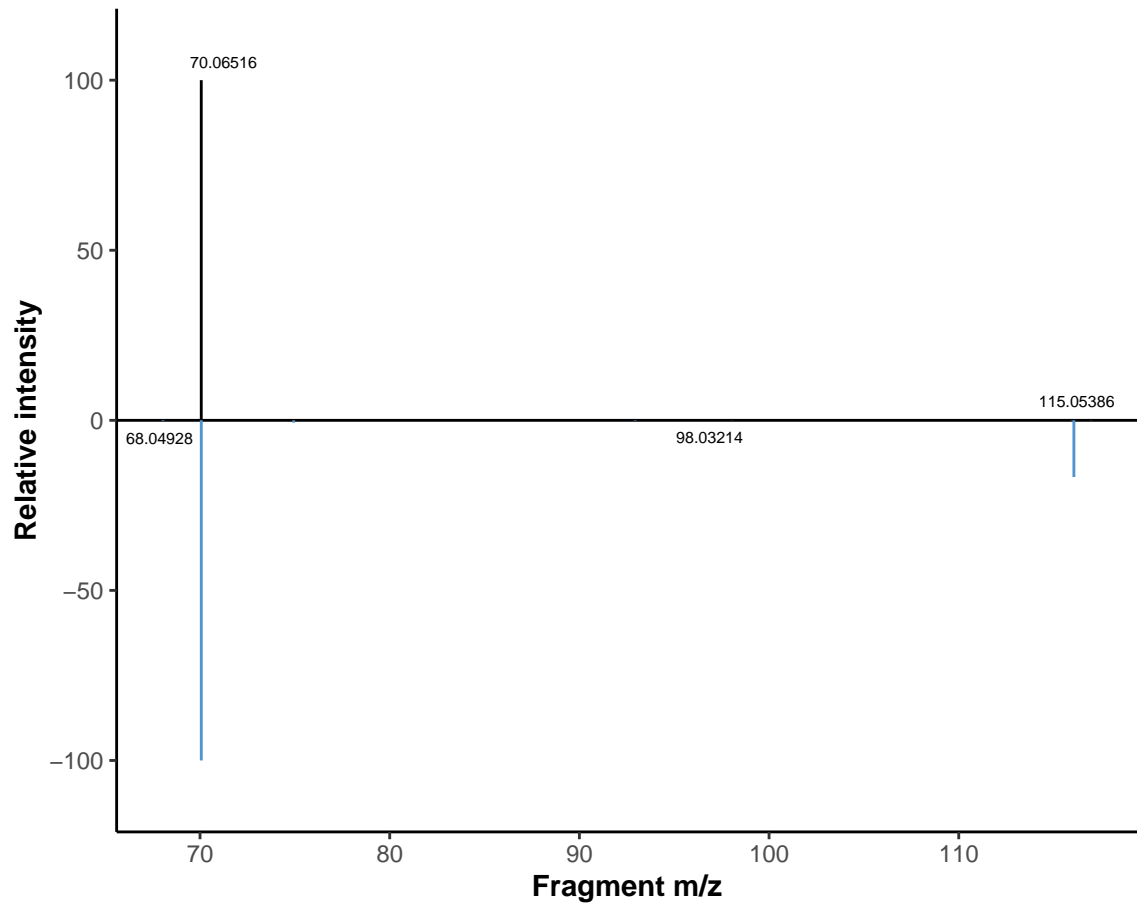

Supplement: Supplementary material 2 — MS identification chart of Xiaojin Pill ingredients. [file Data_Sheet_3.zip › Supplementary Material S2/Positive-299.pdf]

# Guanosine

Relative intensity

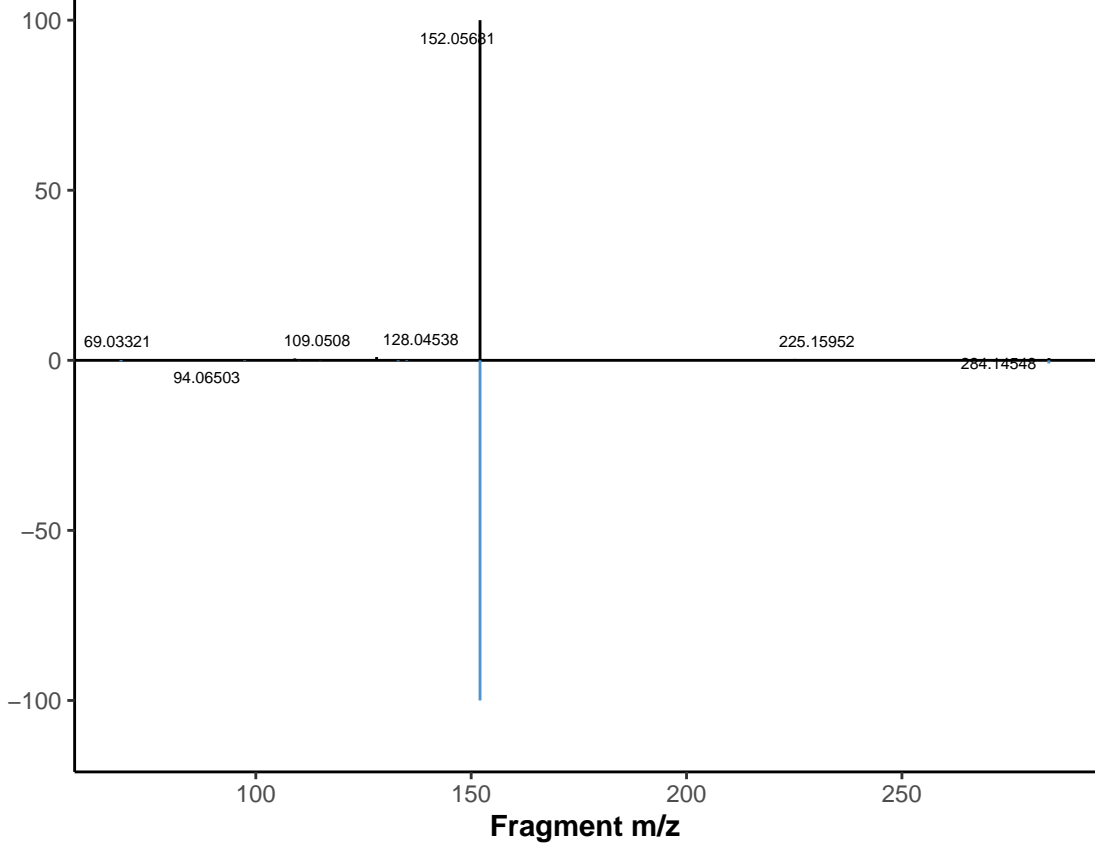

Supplement: Supplementary material 2 — MS identification chart of Xiaojin Pill ingredients. [file Data_Sheet_3.zip › Supplementary Material S2/Positive-3064.pdf]

# Cryptotanshinone

Relative intensity

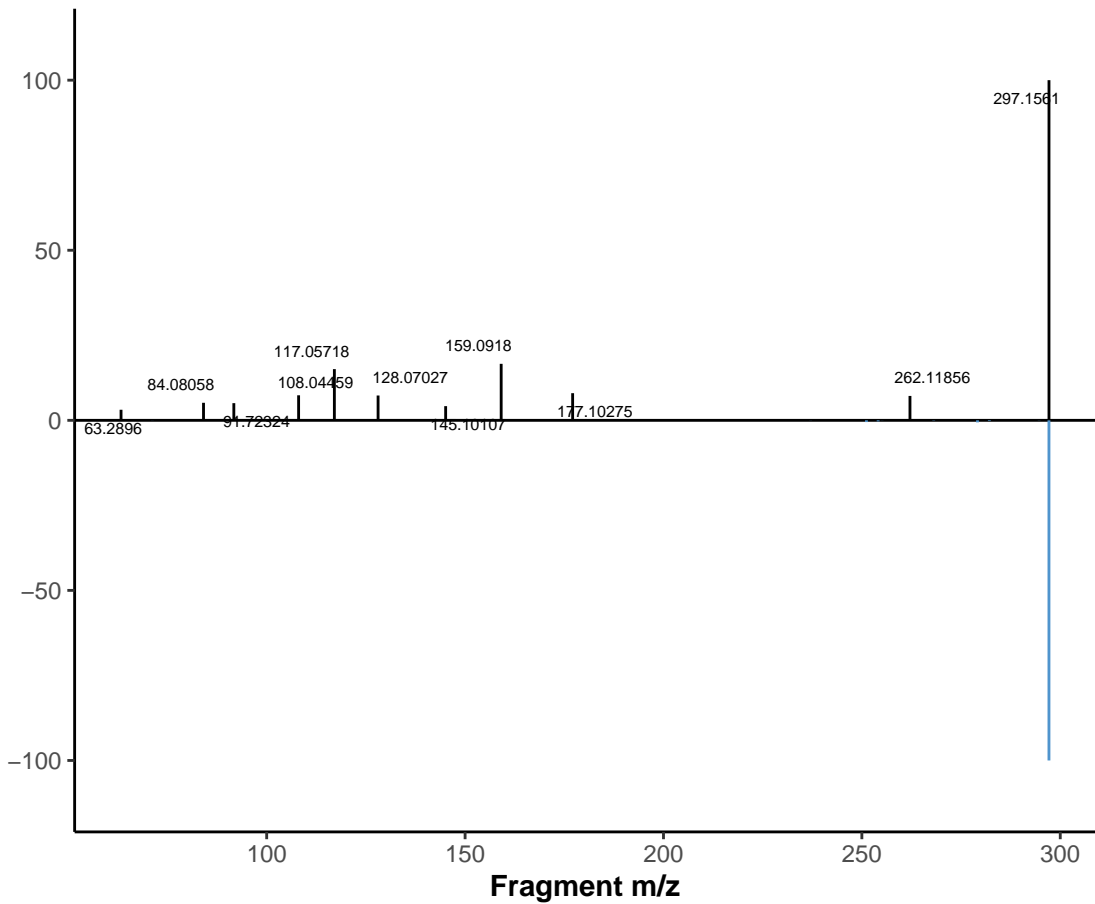

Supplement: Supplementary material 2 — MS identification chart of Xiaojin Pill ingredients. [file Data_Sheet_3.zip › Supplementary Material S2/Positive-3312.pdf]

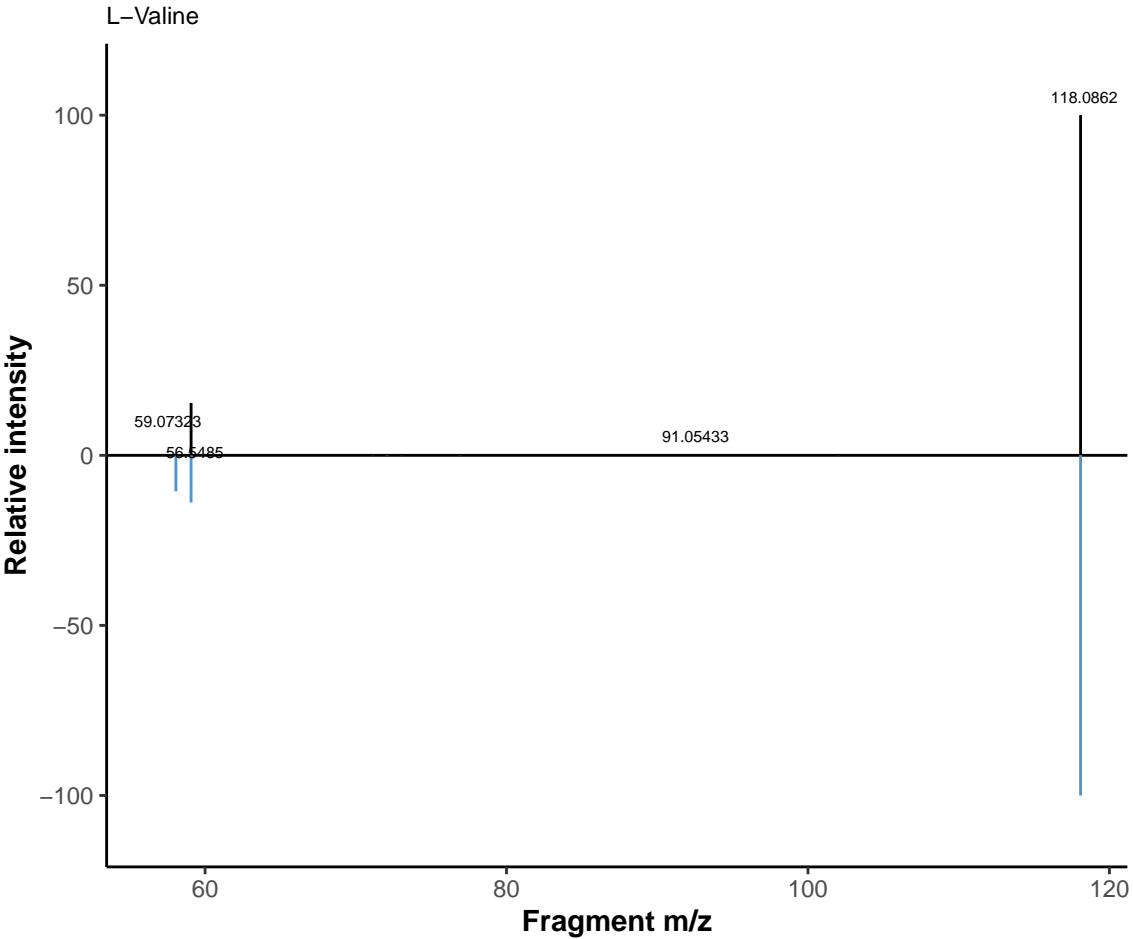

Supplement: Supplementary material 2 — MS identification chart of Xiaojin Pill ingredients. [file Data_Sheet_3.zip › Supplementary Material S2/Positive-332.pdf]

# Kahweol

Relative intensity

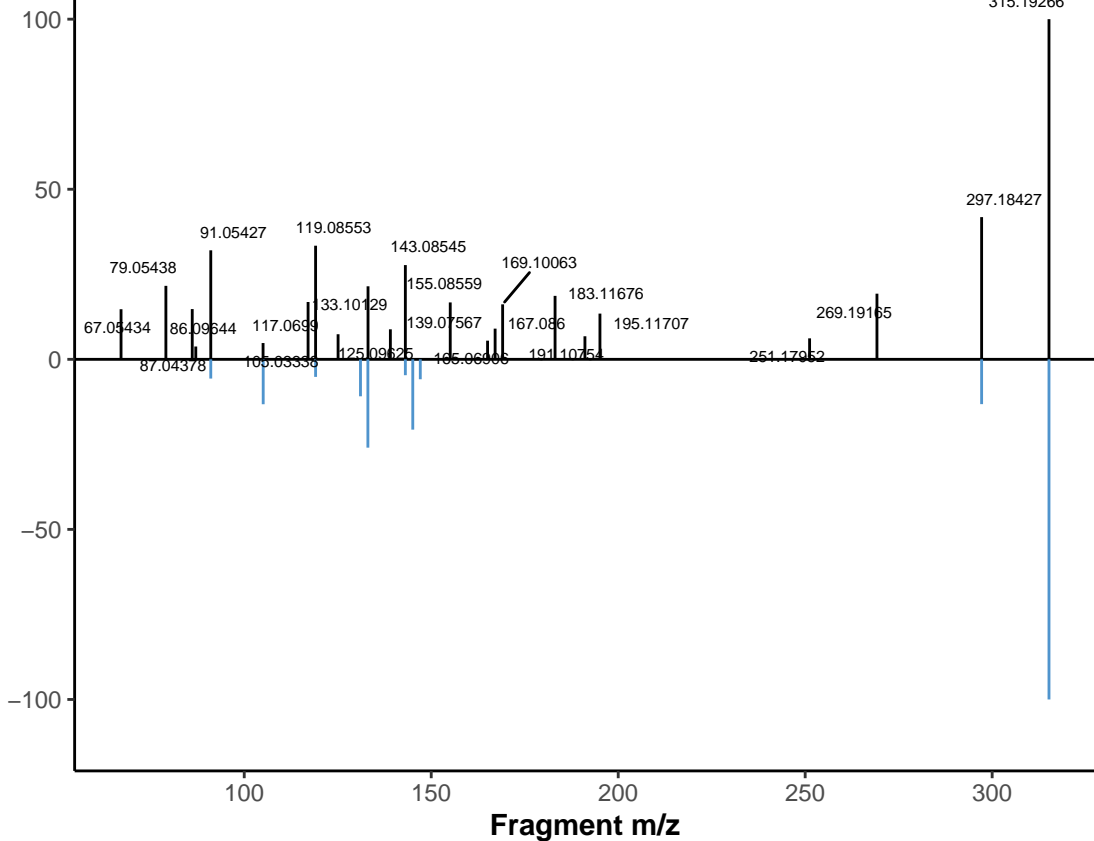

Supplement: Supplementary material 2 — MS identification chart of Xiaojin Pill ingredients. [file Data_Sheet_3.zip › Supplementary Material S2/Positive-3640.pdf]

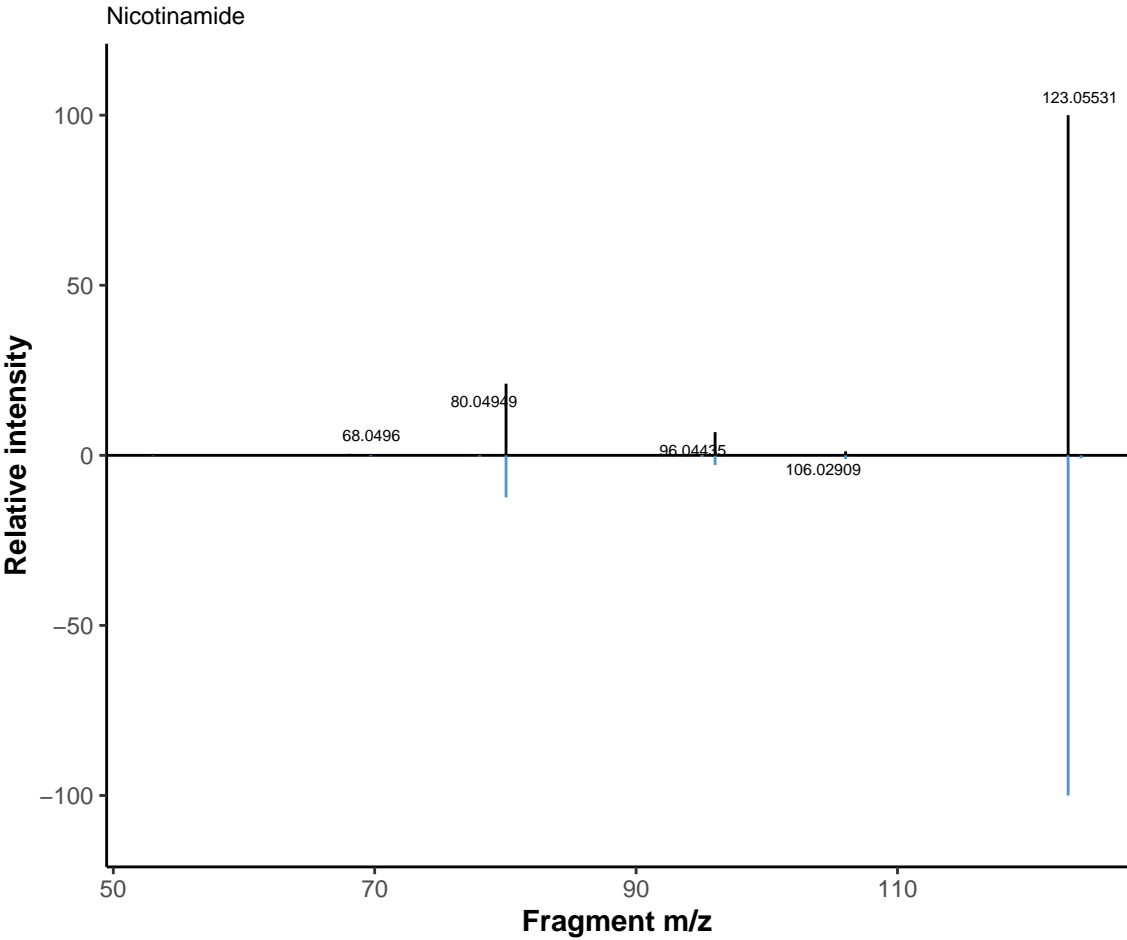

Supplement: Supplementary material 2 — MS identification chart of Xiaojin Pill ingredients. [file Data_Sheet_3.zip › Supplementary Material S2/Positive-372.pdf]

Nicotinic acid

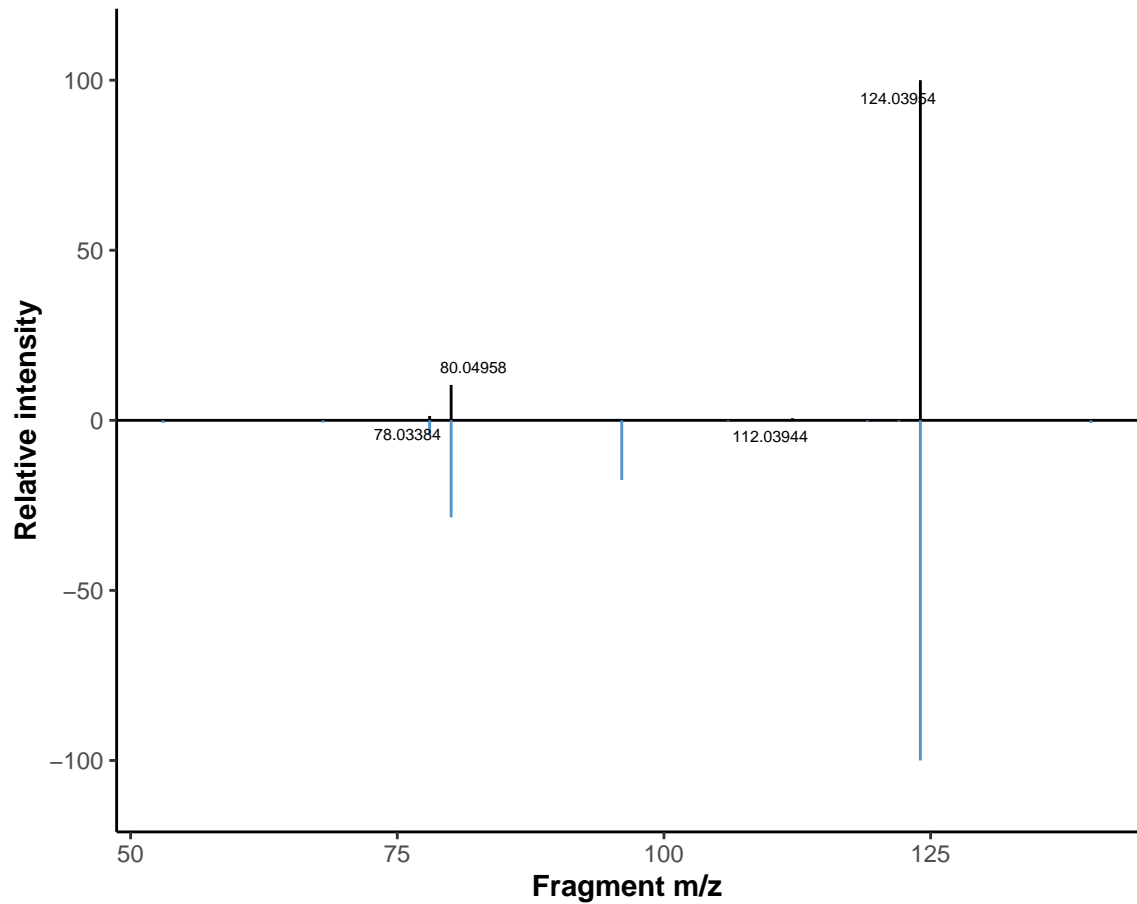

Supplement: Supplementary material 2 — MS identification chart of Xiaojin Pill ingredients. [file Data_Sheet_3.zip › Supplementary Material S2/Positive-384.pdf]

# 14-Deoxyandrographolide

Relative intensity

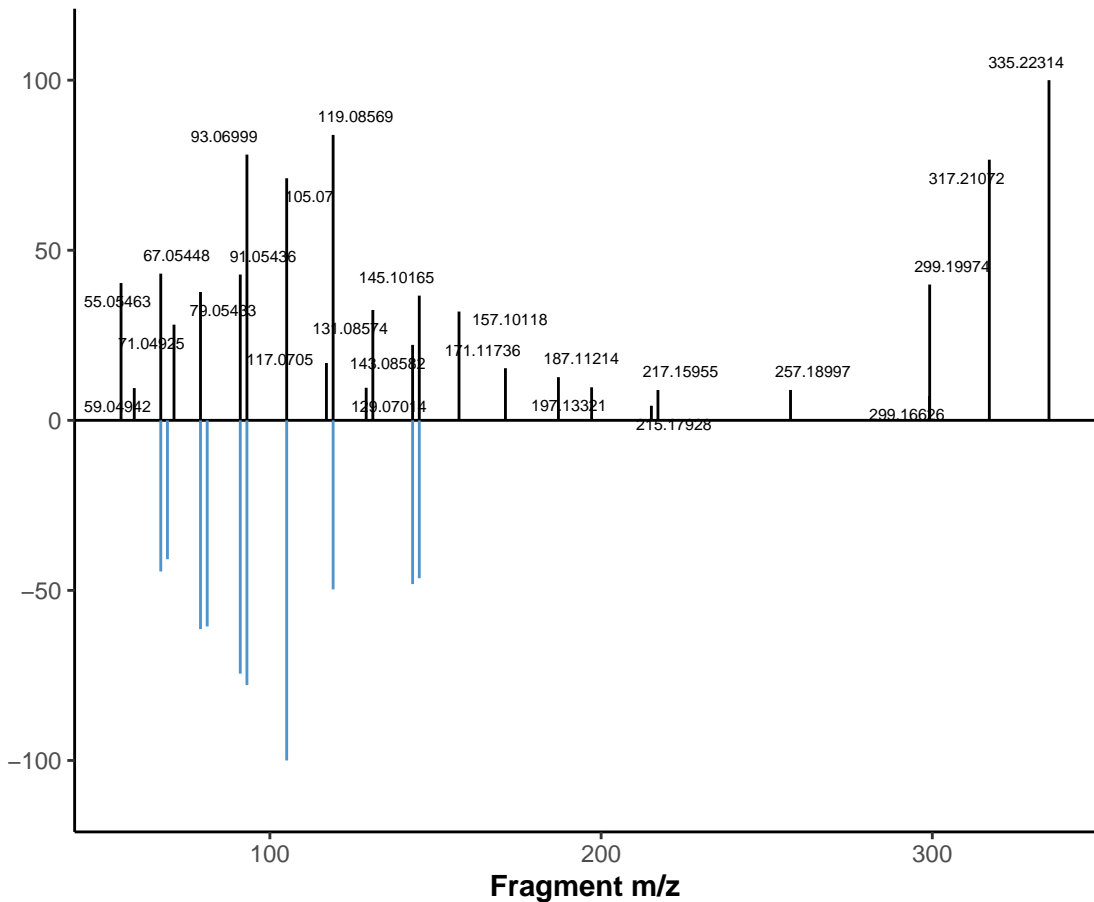

Supplement: Supplementary material 2 — MS identification chart of Xiaojin Pill ingredients. [file Data_Sheet_3.zip › Supplementary Material S2/Positive-3965.pdf]

# Berberine

Relative intensity

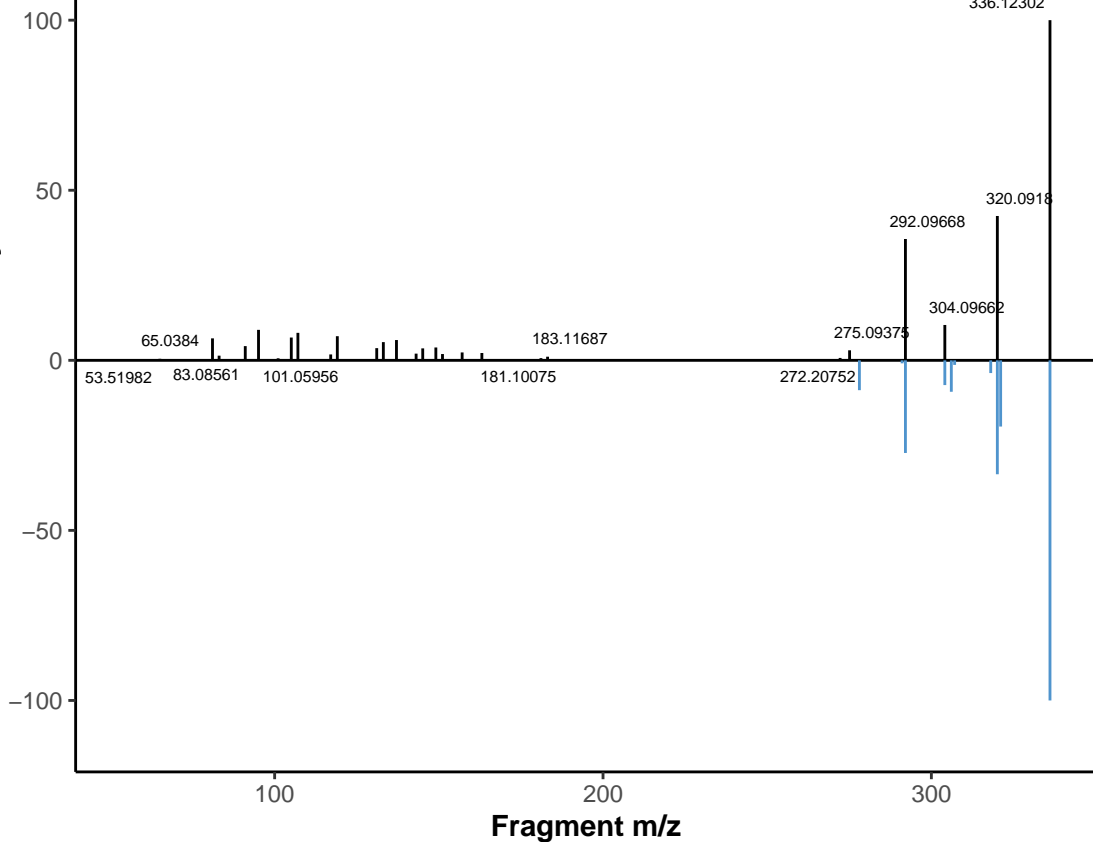

Supplement: Supplementary material 2 — MS identification chart of Xiaojin Pill ingredients. [file Data_Sheet_3.zip › Supplementary Material S2/Positive-3971.pdf]

(+)-Magnoflorine

Relative intensity

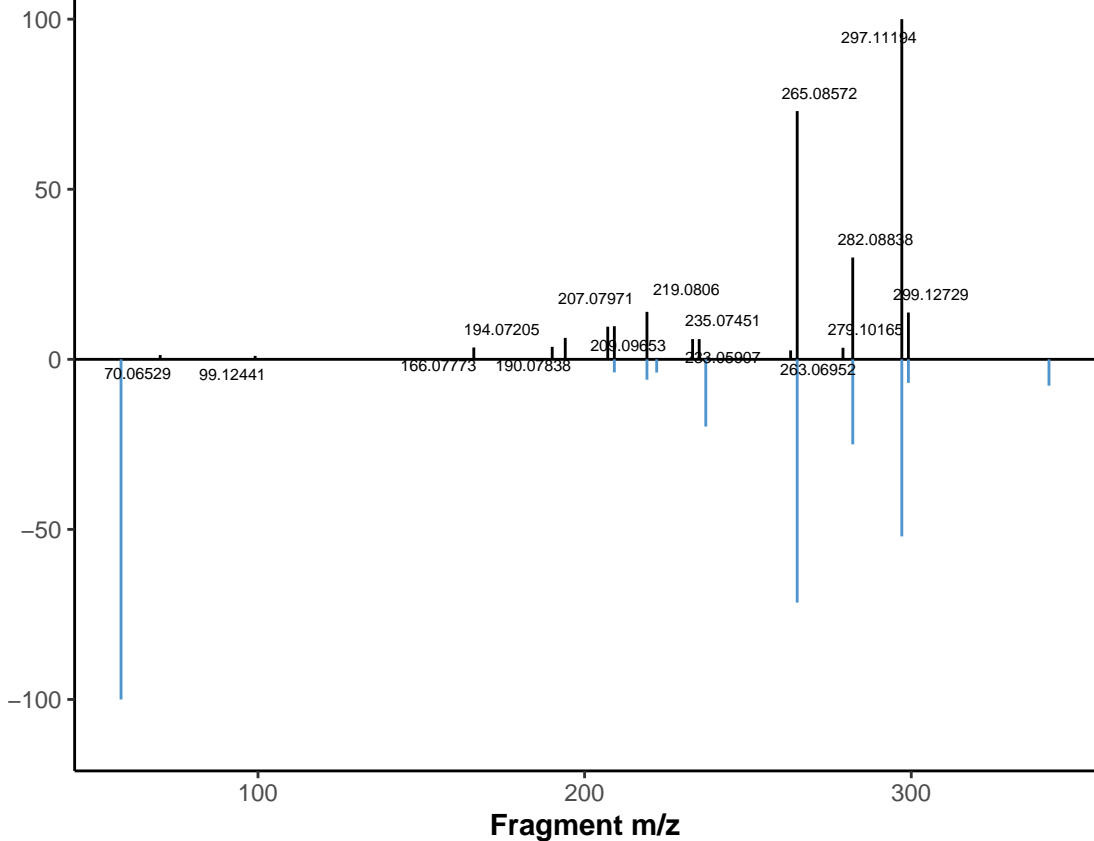

Supplement: Supplementary material 2 — MS identification chart of Xiaojin Pill ingredients. [file Data_Sheet_3.zip › Supplementary Material S2/Positive-4077.pdf]

# Bullatine A

Relative intensity

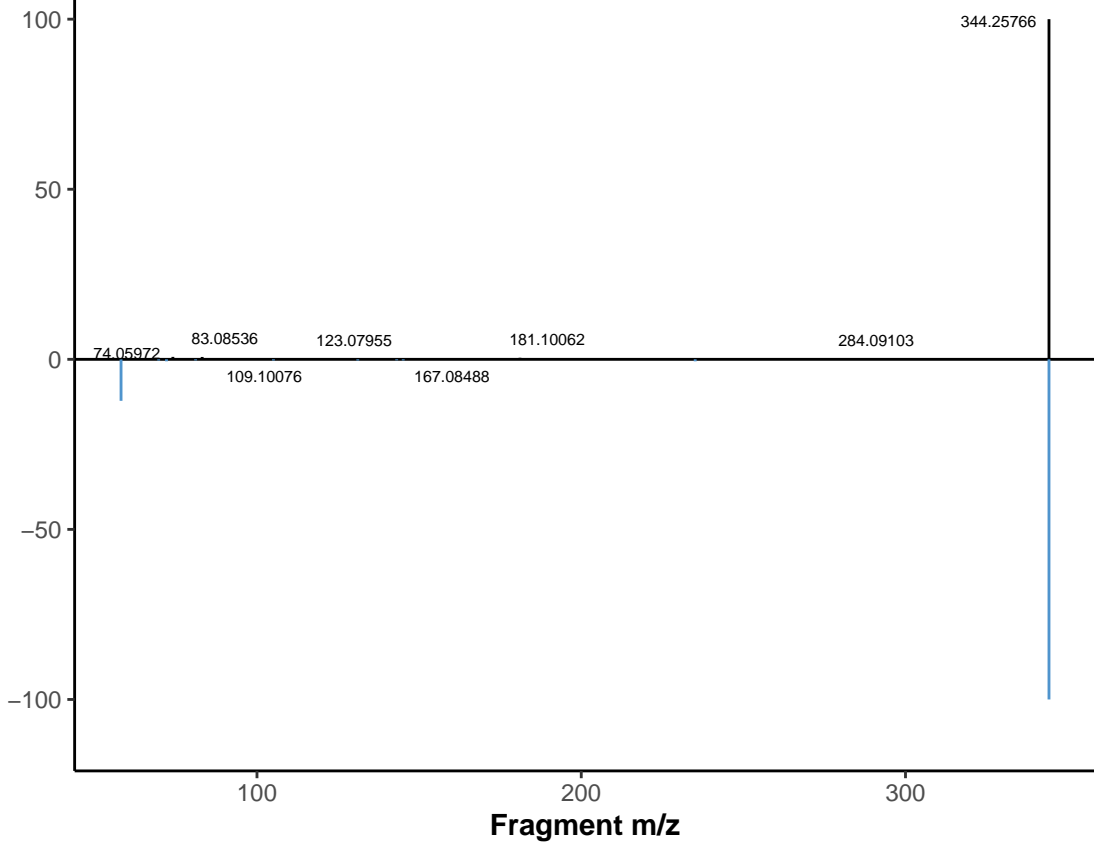

Supplement: Supplementary material 2 — MS identification chart of Xiaojin Pill ingredients. [file Data_Sheet_3.zip › Supplementary Material S2/Positive-4103.pdf]

5-Hydroxymethylfurfural

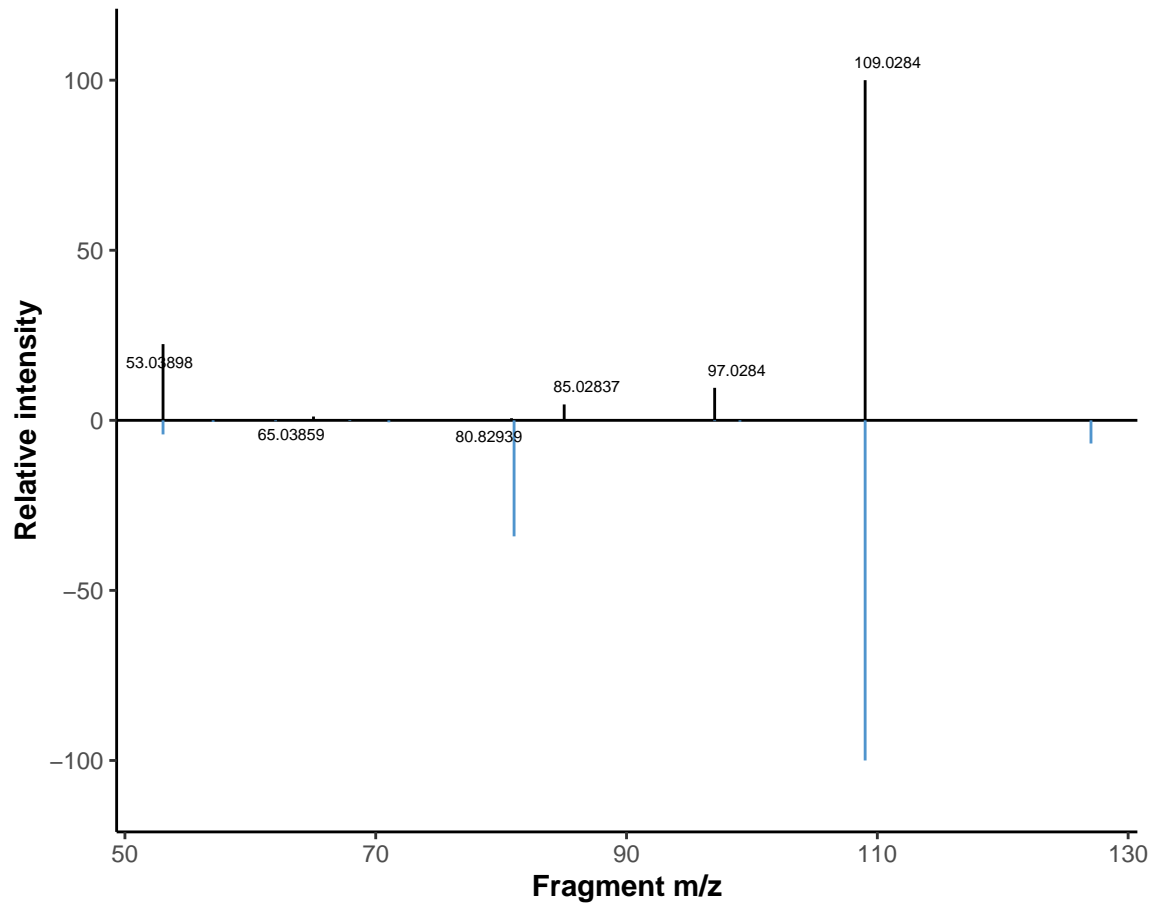

Supplement: Supplementary material 2 — MS identification chart of Xiaojin Pill ingredients. [file Data_Sheet_3.zip › Supplementary Material S2/Positive-415.pdf]

# Bullatine G

Relative intensity

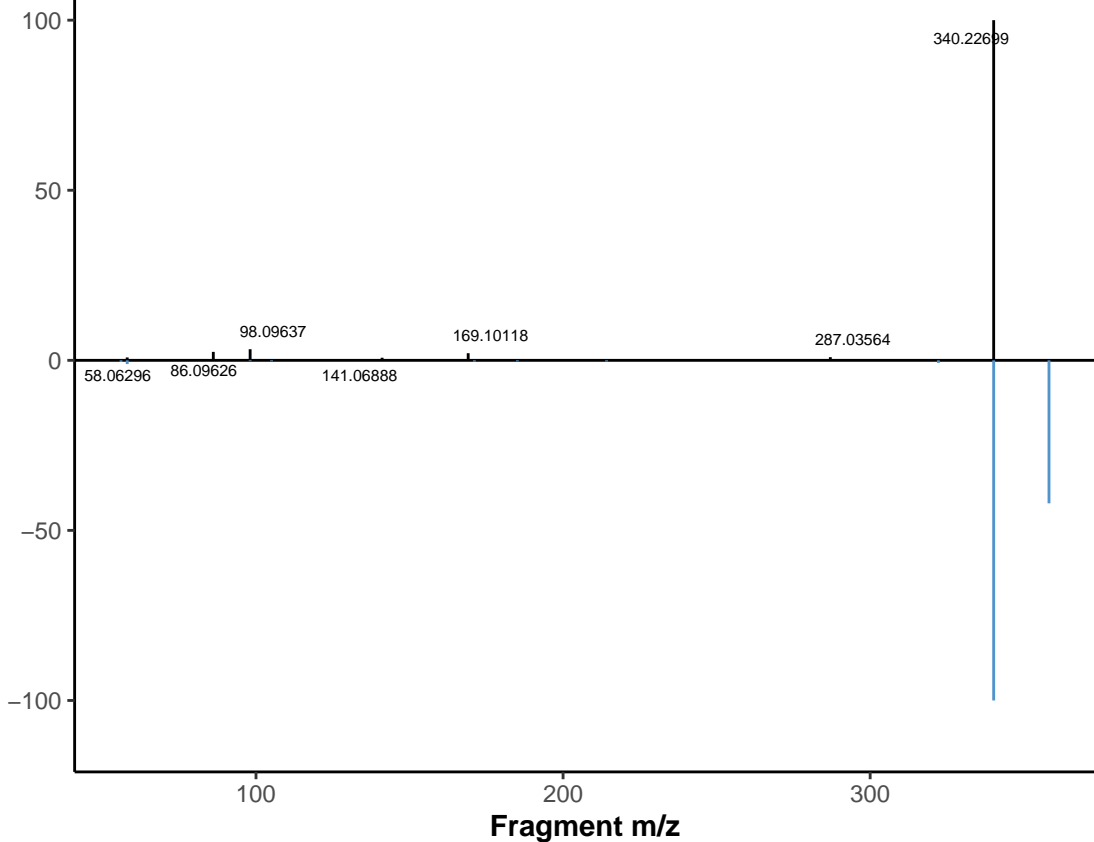

Supplement: Supplementary material 2 — MS identification chart of Xiaojin Pill ingredients. [file Data_Sheet_3.zip › Supplementary Material S2/Positive-4348.pdf]

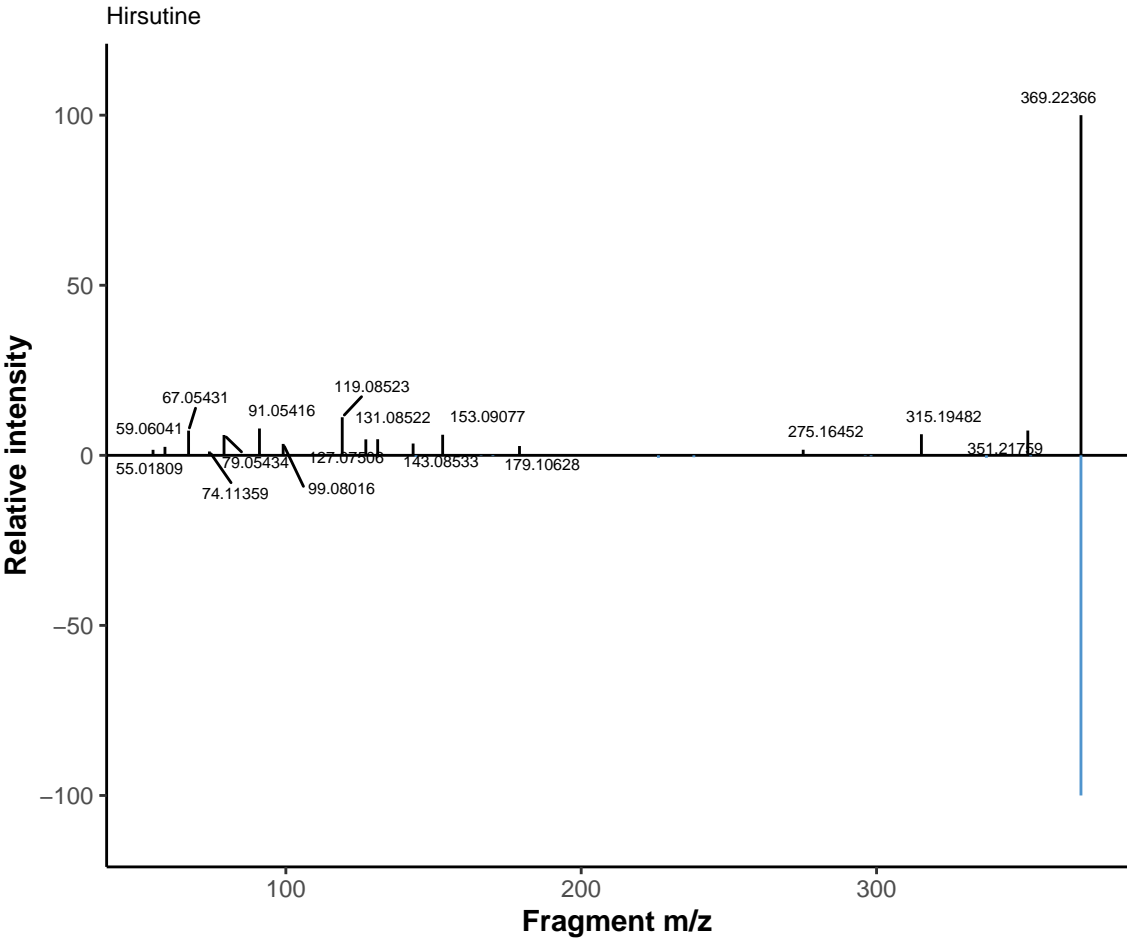

Supplement: Supplementary material 2 — MS identification chart of Xiaojin Pill ingredients. [file Data_Sheet_3.zip › Supplementary Material S2/Positive-4505.pdf]

# 7-Ethylcamptothecin

Relative intensity

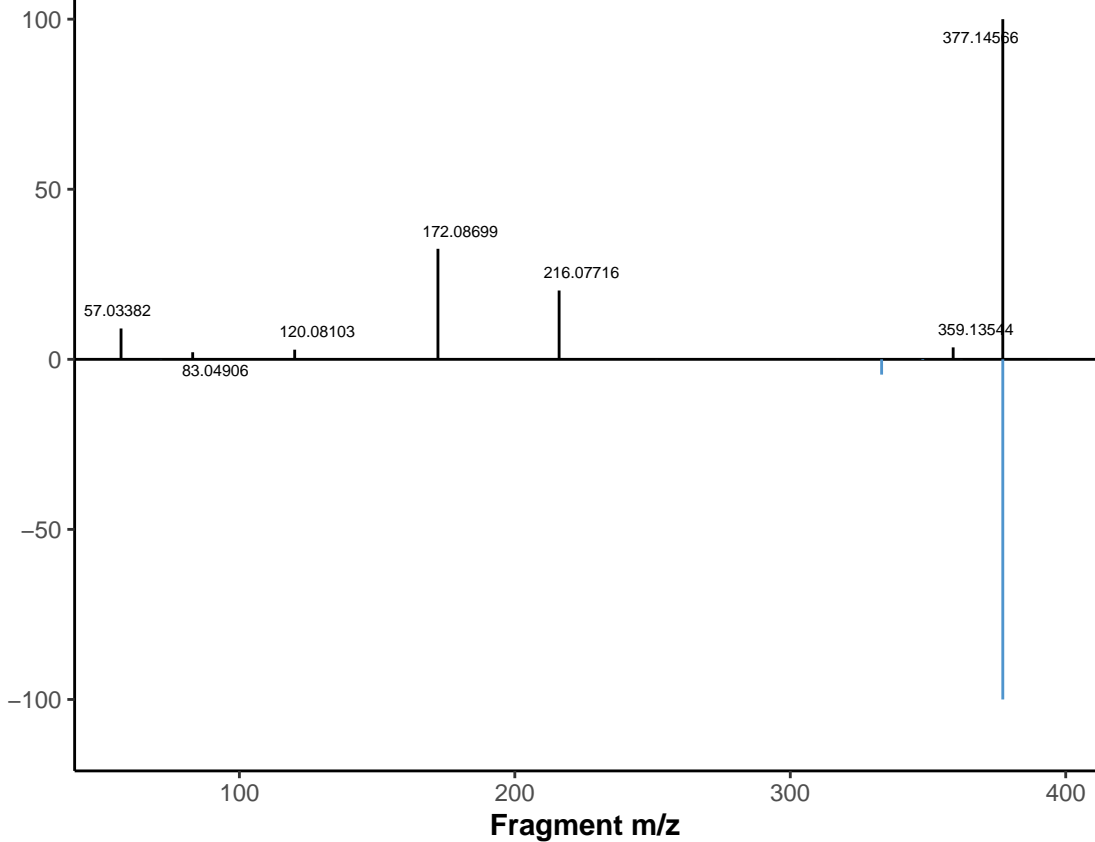

Supplement: Supplementary material 2 — MS identification chart of Xiaojin Pill ingredients. [file Data_Sheet_3.zip › Supplementary Material S2/Positive-4631.pdf]

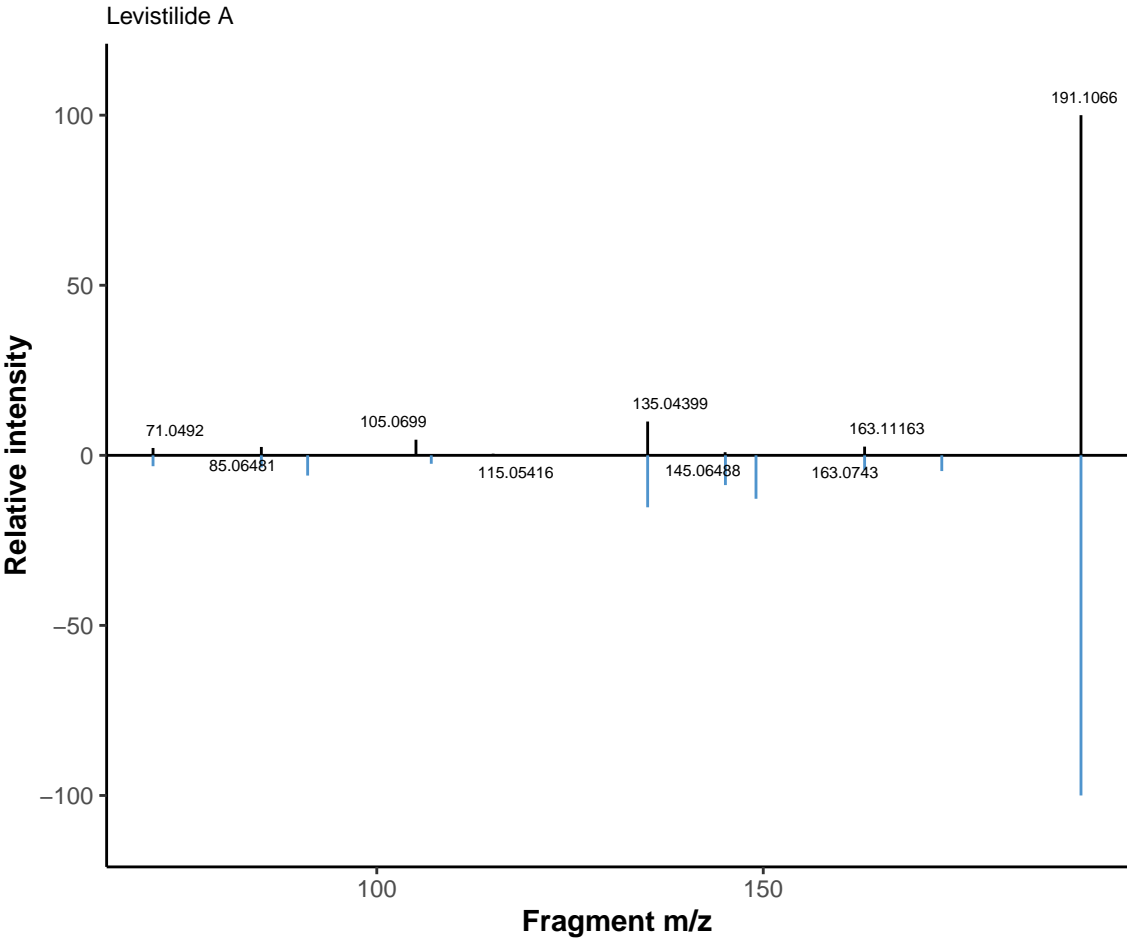

Supplement: Supplementary material 2 — MS identification chart of Xiaojin Pill ingredients. [file Data_Sheet_3.zip › Supplementary Material S2/Positive-4697.pdf]

# Corynoxetine

Relative intensity

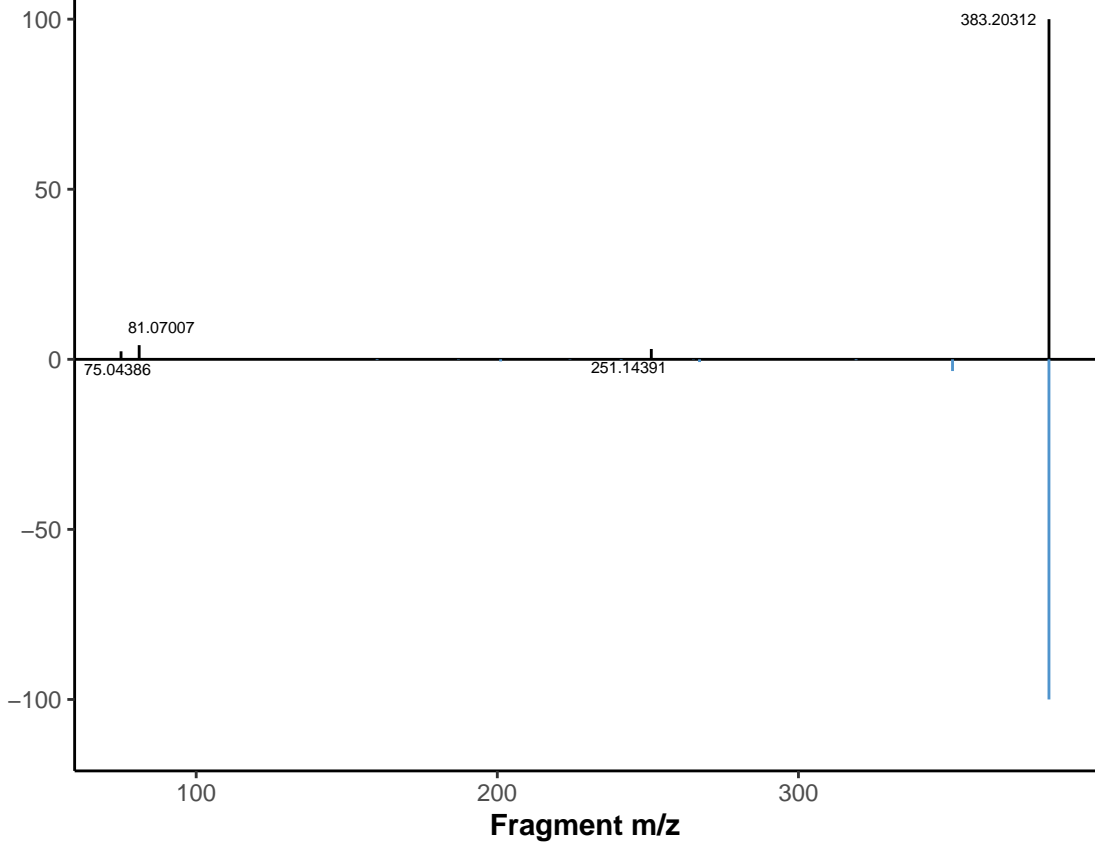

Supplement: Supplementary material 2 — MS identification chart of Xiaojin Pill ingredients. [file Data_Sheet_3.zip › Supplementary Material S2/Positive-4728.pdf]

# L-Leucine

Relative intensity

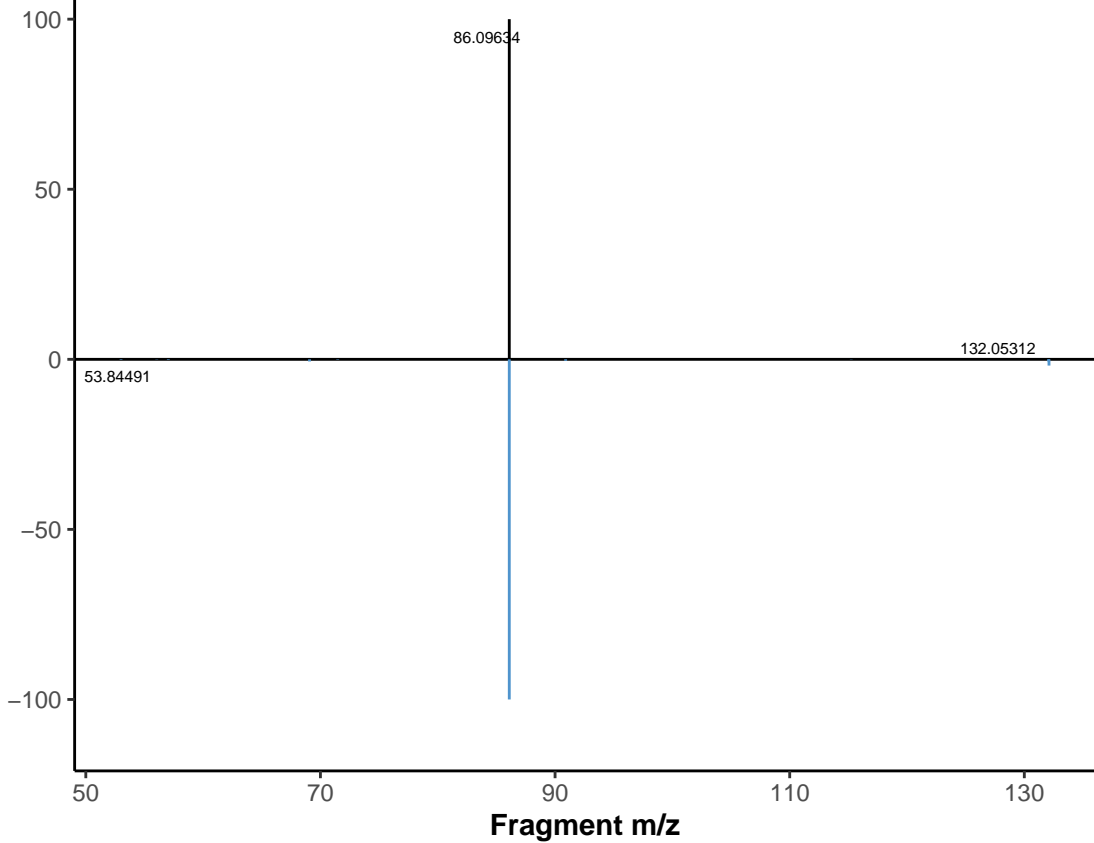

Supplement: Supplementary material 2 — MS identification chart of Xiaojin Pill ingredients. [file Data_Sheet_3.zip › Supplementary Material S2/Positive-475.pdf]

# Cinnamaldehyde

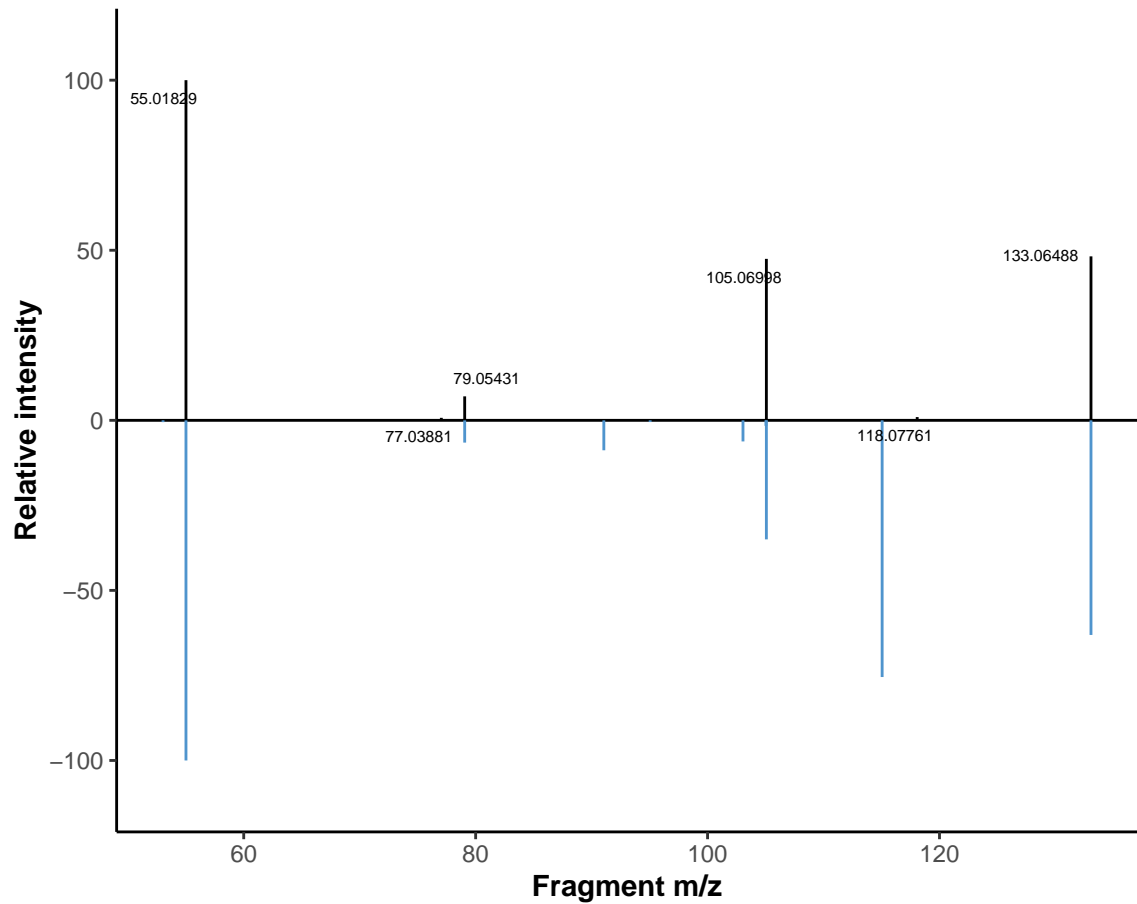

Supplement: Supplementary material 2 — MS identification chart of Xiaojin Pill ingredients. [file Data_Sheet_3.zip › Supplementary Material S2/Positive-485.pdf]

# Telocinobufagin

Relative intensity

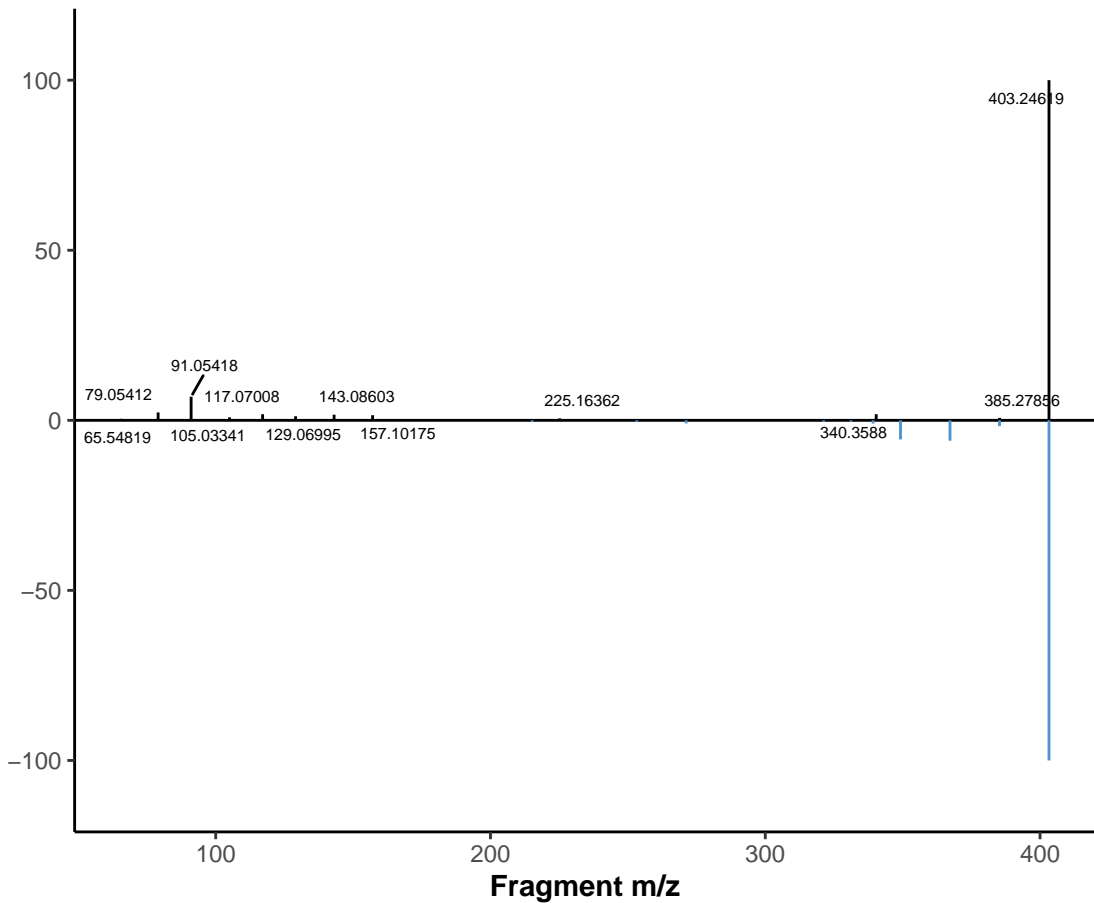

Supplement: Supplementary material 2 — MS identification chart of Xiaojin Pill ingredients. [file Data_Sheet_3.zip › Supplementary Material S2/Positive-5029.pdf]

# Arenobufagin

Relative intensity

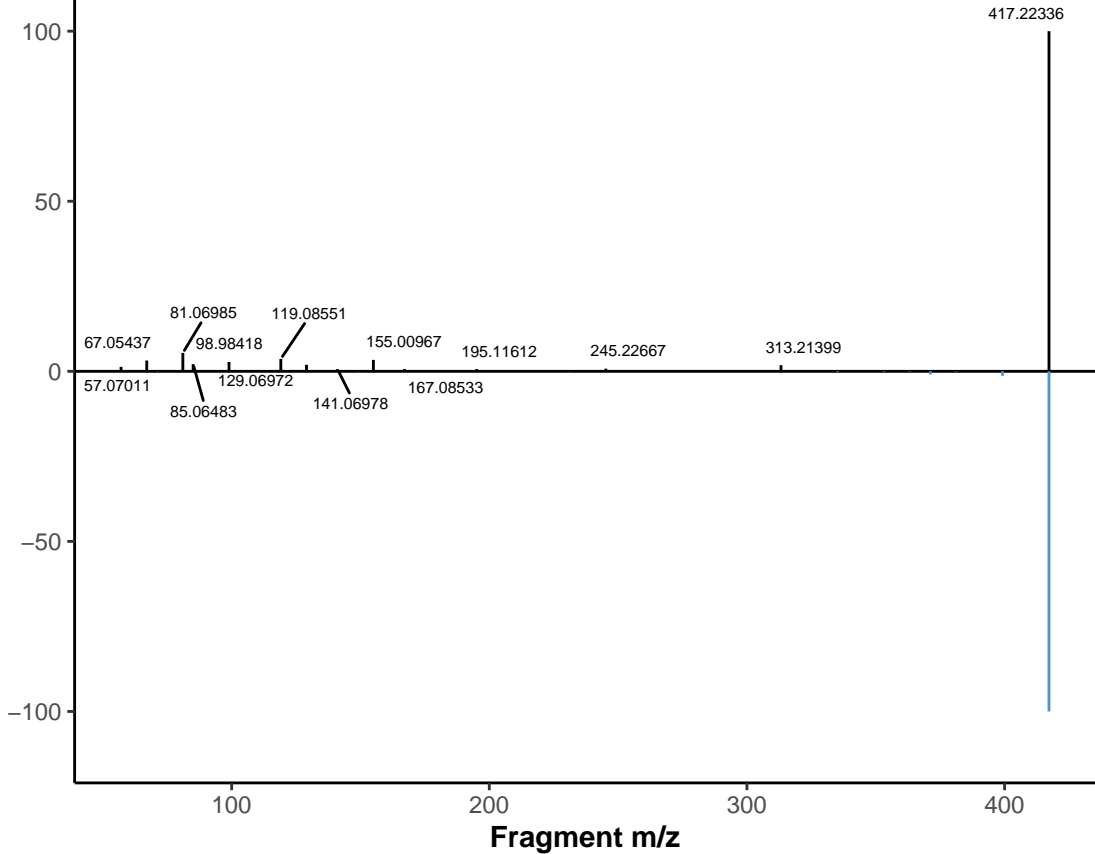

Supplement: Supplementary material 2 — MS identification chart of Xiaojin Pill ingredients. [file Data_Sheet_3.zip › Supplementary Material S2/Positive-5253.pdf]

# Talatisamine

Relative intensity

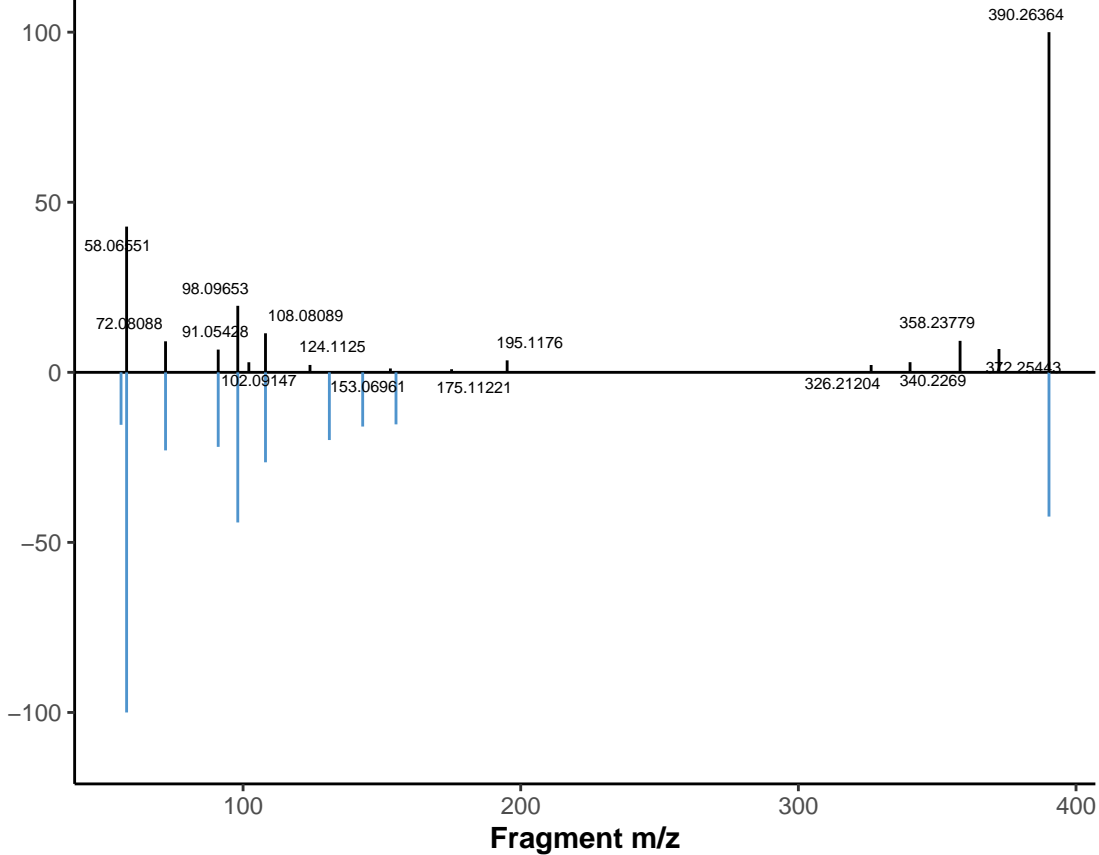

Supplement: Supplementary material 2 — MS identification chart of Xiaojin Pill ingredients. [file Data_Sheet_3.zip › Supplementary Material S2/Positive-5317.pdf]

Ligustrazine HCl

Relative intensity

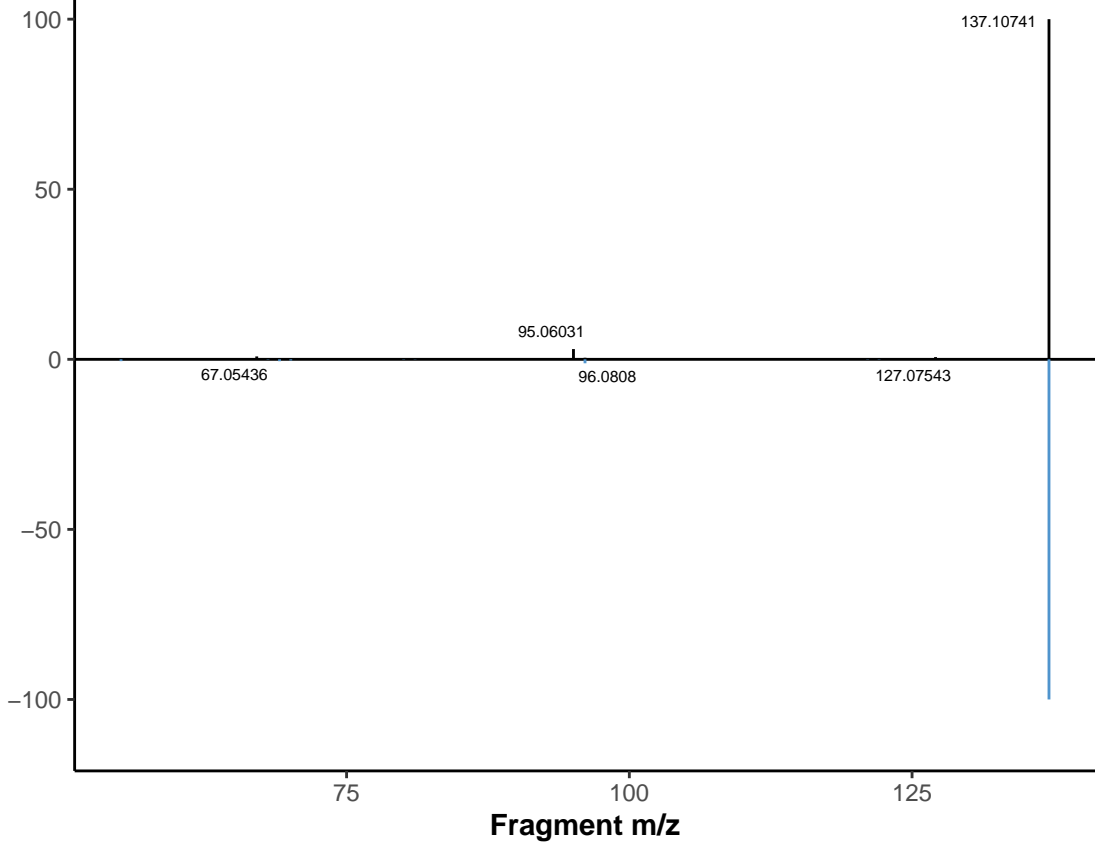

Supplement: Supplementary material 2 — MS identification chart of Xiaojin Pill ingredients. [file Data_Sheet_3.zip › Supplementary Material S2/Positive-540.pdf]

# Trigonelline HCl

Relative intensity

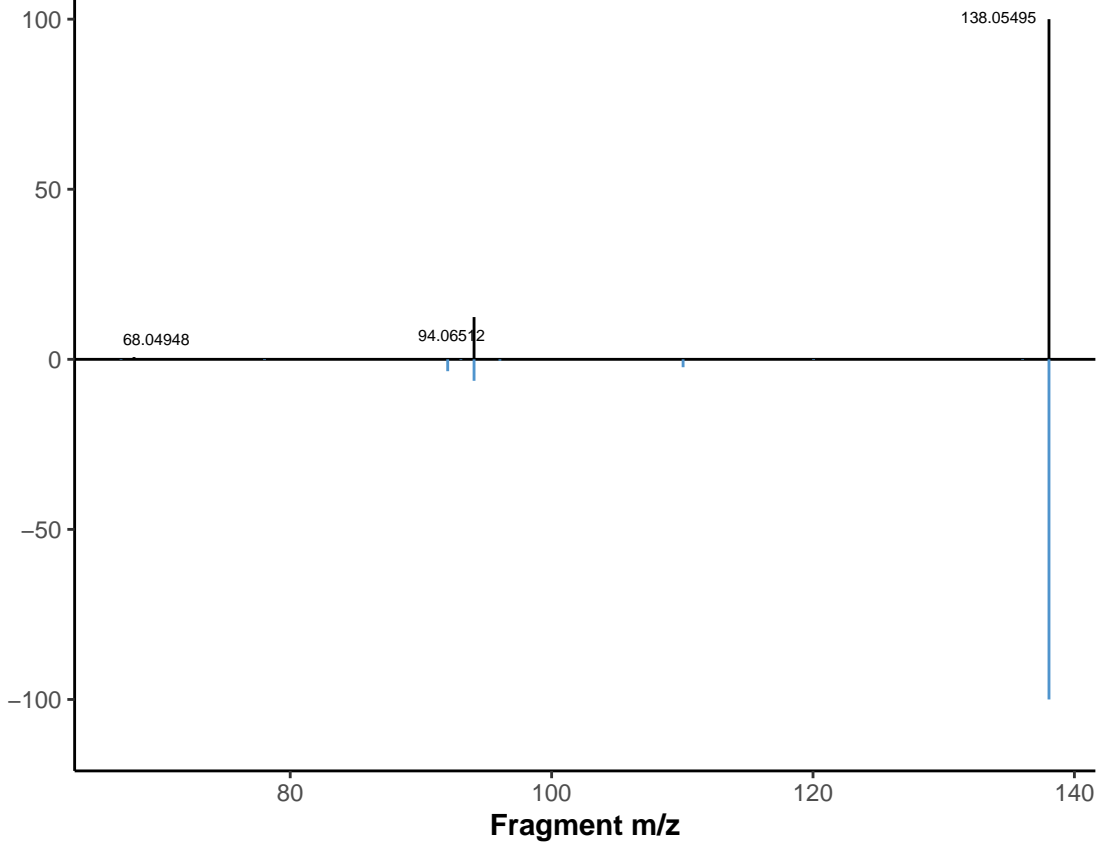

Supplement: Supplementary material 2 — MS identification chart of Xiaojin Pill ingredients. [file Data_Sheet_3.zip › Supplementary Material S2/Positive-548.pdf]

# Neoline/Bullatine B

Relative intensity

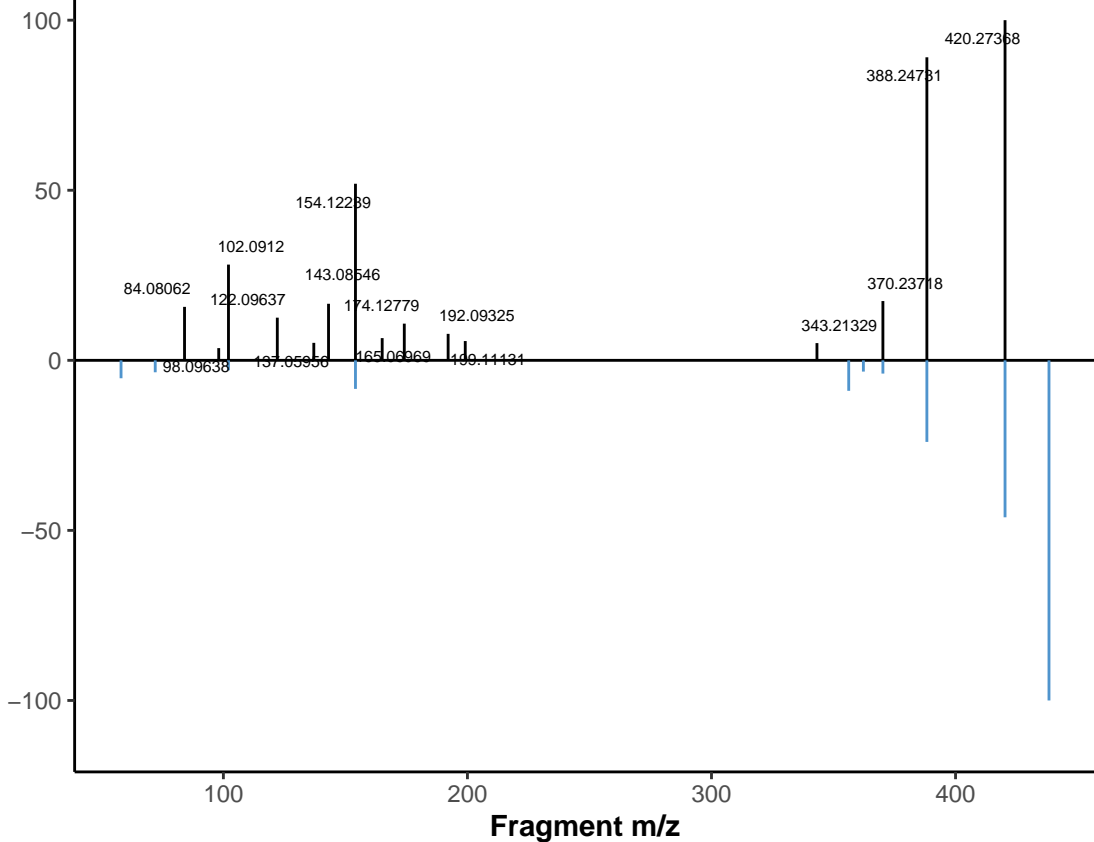

Supplement: Supplementary material 2 — MS identification chart of Xiaojin Pill ingredients. [file Data_Sheet_3.zip › Supplementary Material S2/Positive-5531.pdf]

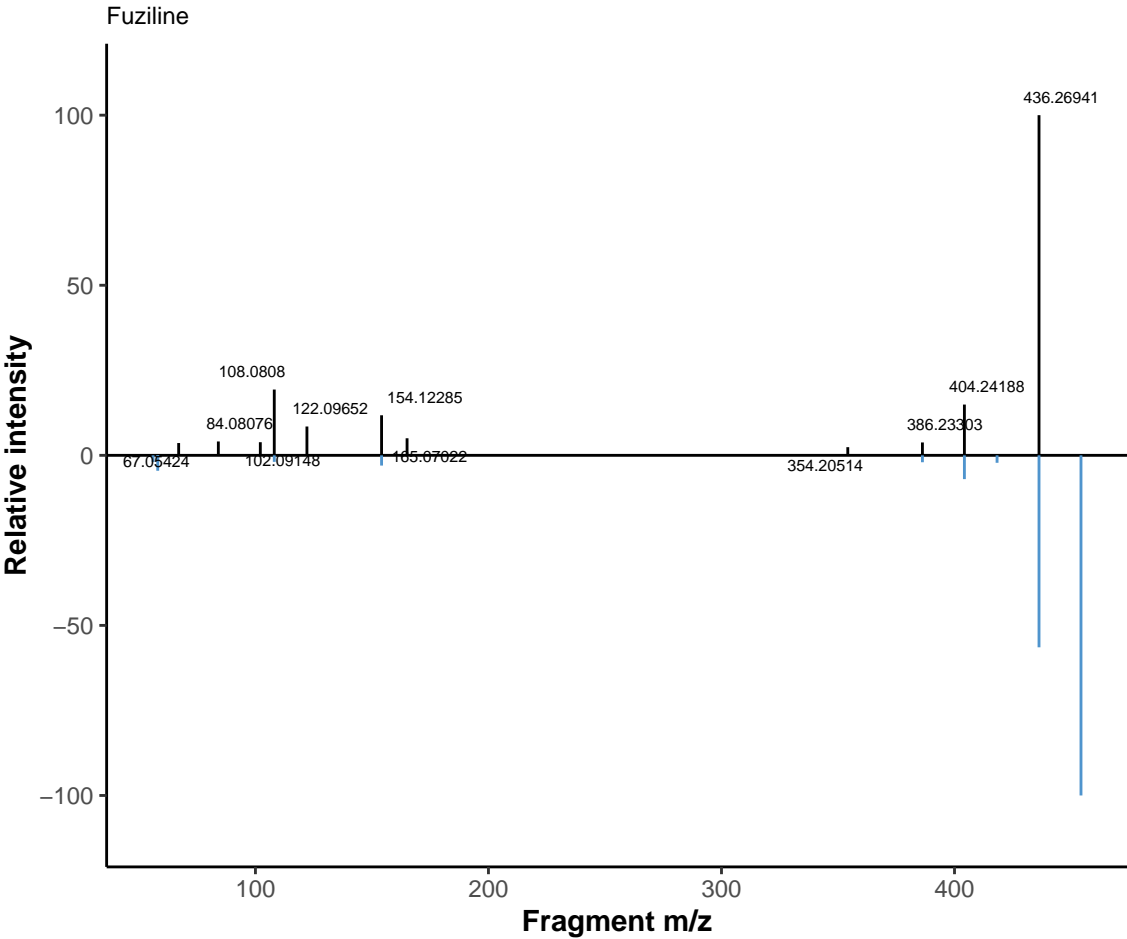

Supplement: Supplementary material 2 — MS identification chart of Xiaojin Pill ingredients. [file Data_Sheet_3.zip › Supplementary Material S2/Positive-5749.pdf]

# Oleanonic acid

Relative intensity

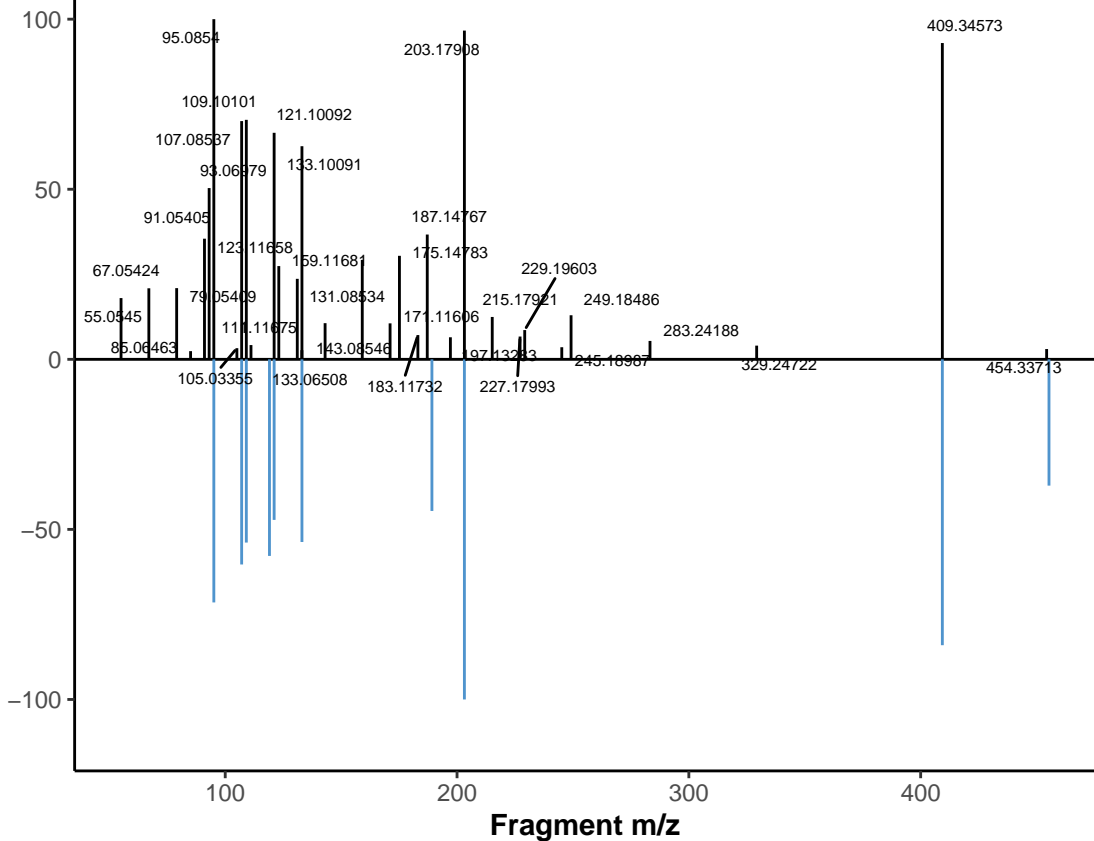

Supplement: Supplementary material 2 — MS identification chart of Xiaojin Pill ingredients. [file Data_Sheet_3.zip › Supplementary Material S2/Positive-5764.pdf]

# Cephaeline

Relative intensity

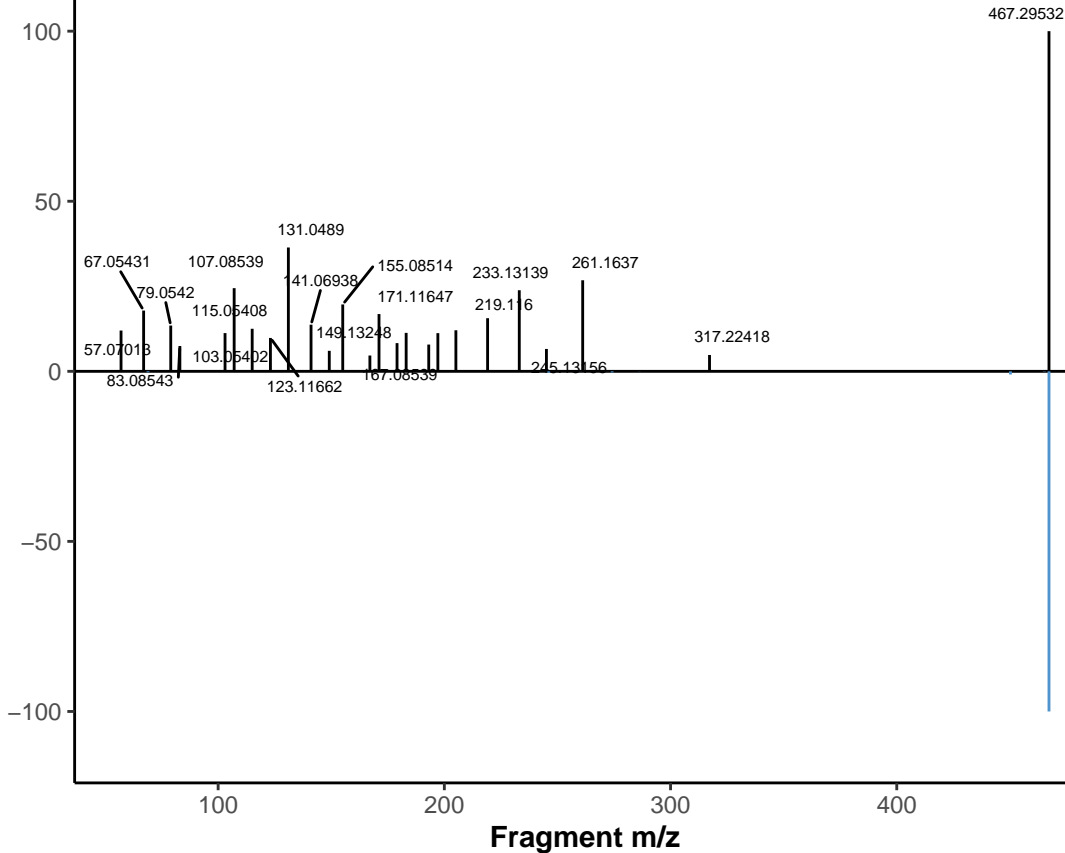

Supplement: Supplementary material 2 — MS identification chart of Xiaojin Pill ingredients. [file Data_Sheet_3.zip › Supplementary Material S2/Positive-5903.pdf]

# 18 beta-Glycyrrhetinic Acid

Relative intensity

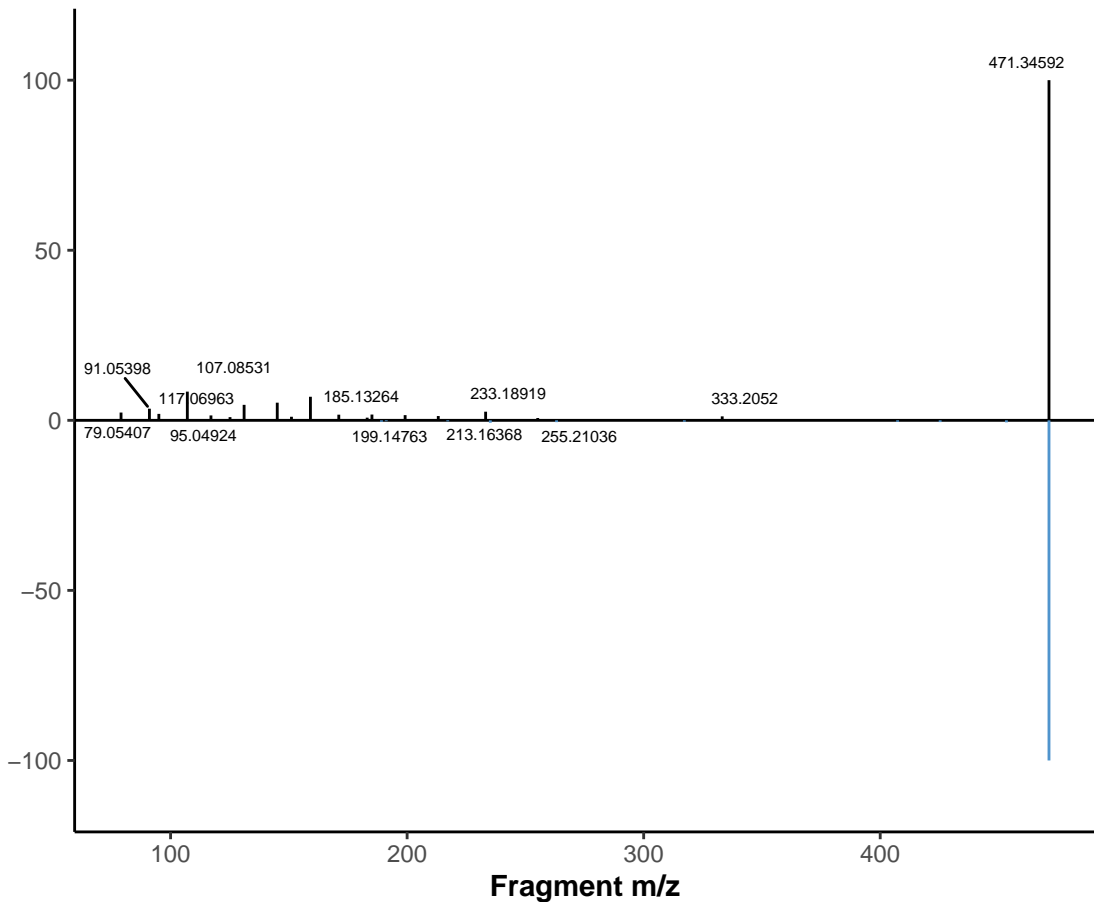

Supplement: Supplementary material 2 — MS identification chart of Xiaojin Pill ingredients. [file Data_Sheet_3.zip › Supplementary Material S2/Positive-5947.pdf]

# Stachydrine

Relative intensity

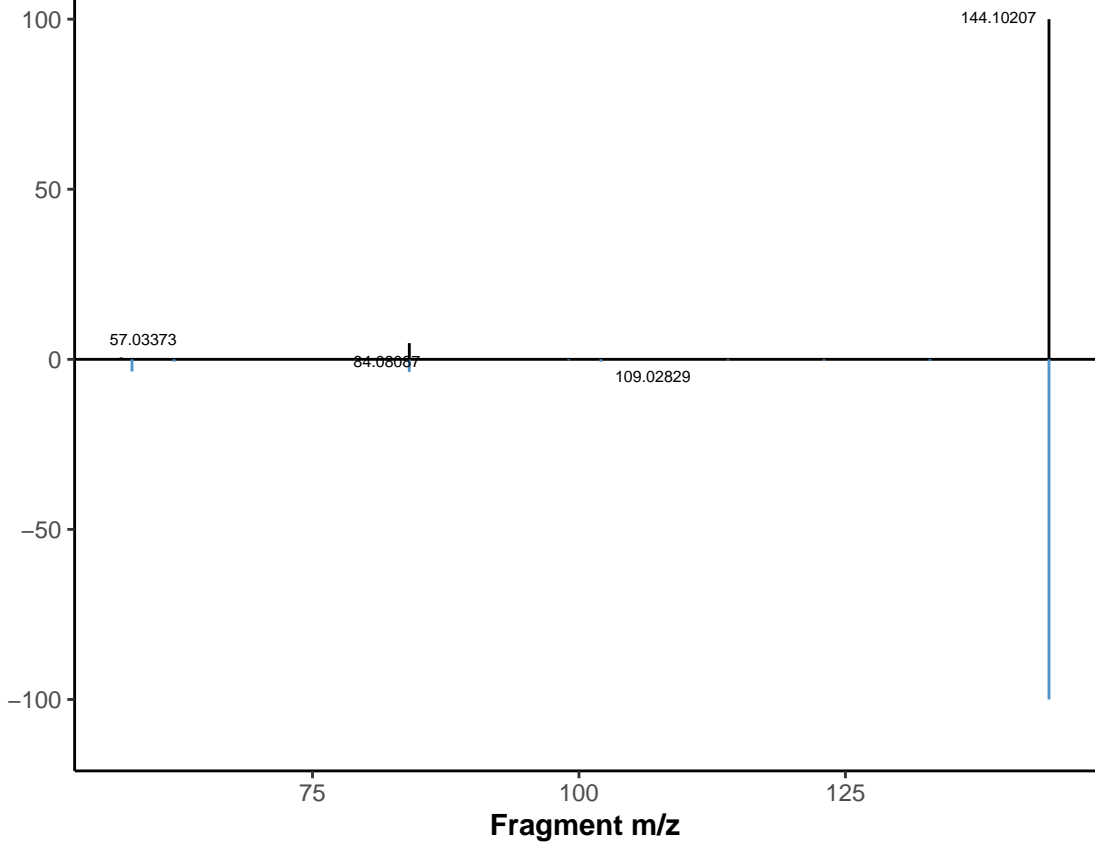

Supplement: Supplementary material 2 — MS identification chart of Xiaojin Pill ingredients. [file Data_Sheet_3.zip › Supplementary Material S2/Positive-620.pdf]

# Benzoylhypaconine

Relative intensity

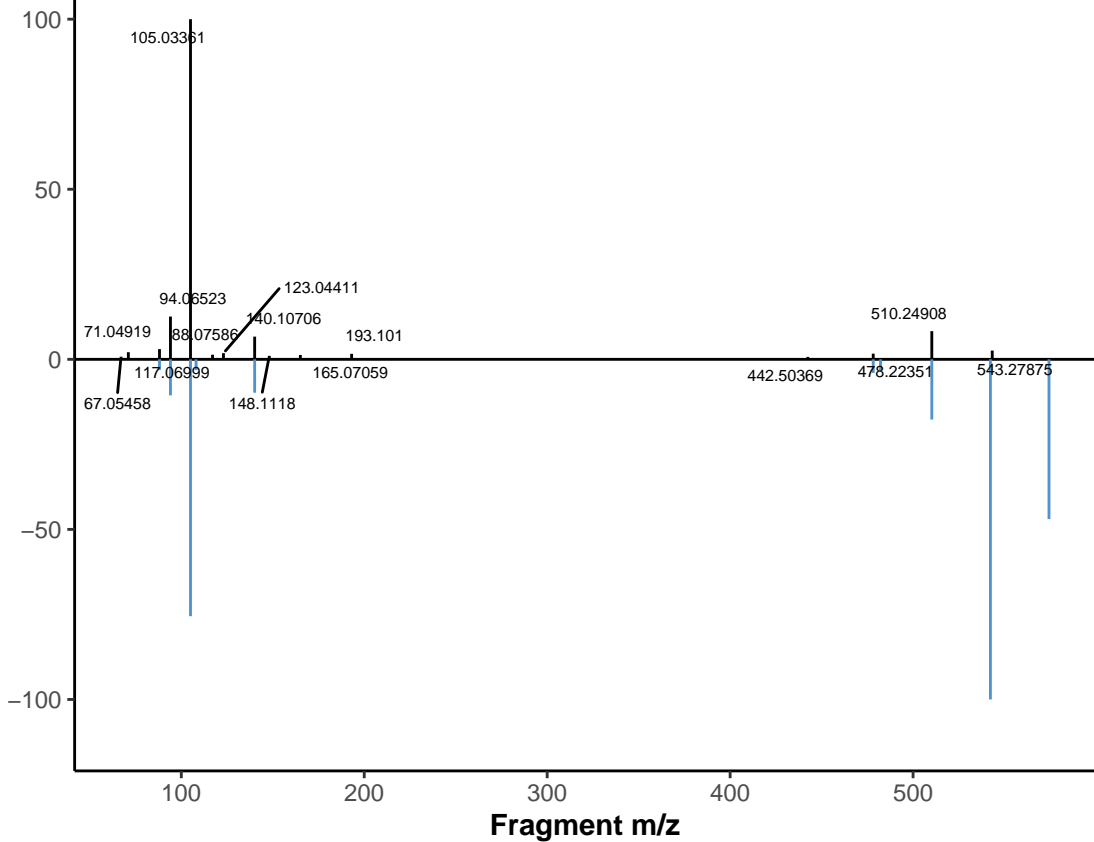

Supplement: Supplementary material 2 — MS identification chart of Xiaojin Pill ingredients. [file Data_Sheet_3.zip › Supplementary Material S2/Positive-6875.pdf]

# Benzoylmesaconine

Relative intensity

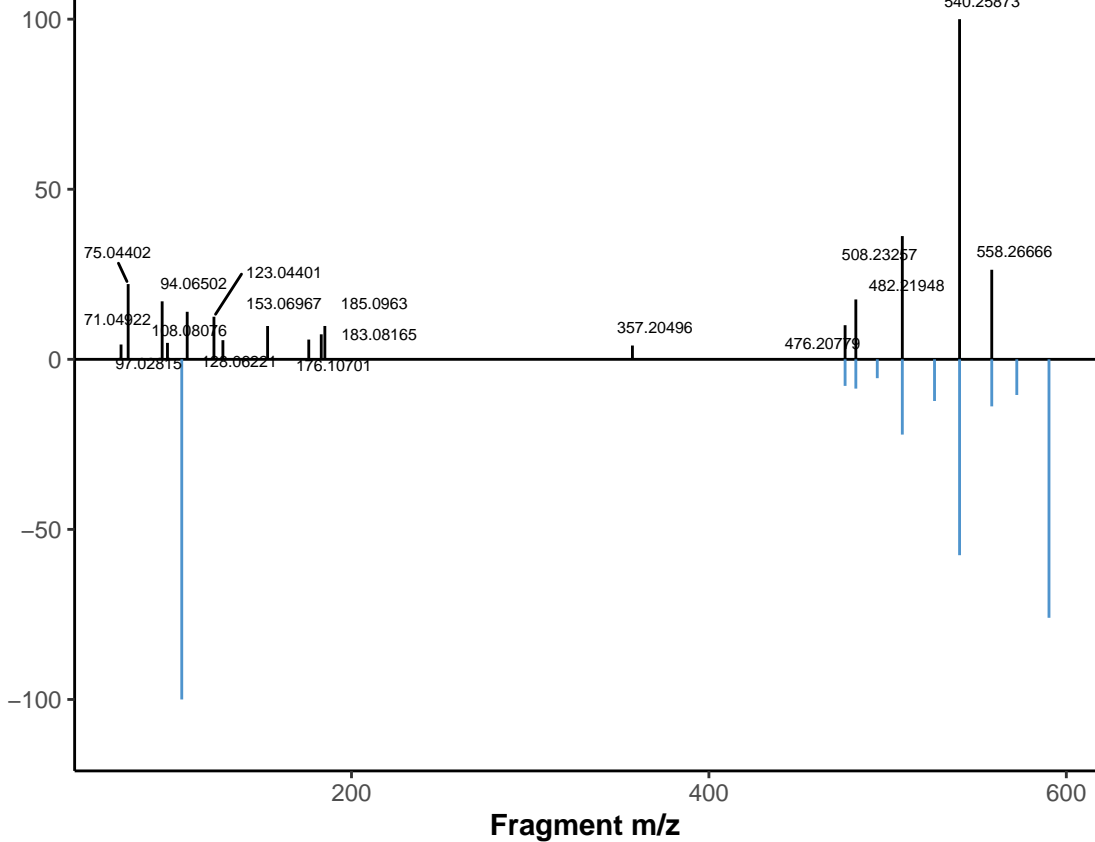

Supplement: Supplementary material 2 — MS identification chart of Xiaojin Pill ingredients. [file Data_Sheet_3.zip › Supplementary Material S2/Positive-6996.pdf]

# 3-Deoxyaconitine

Relative intensity

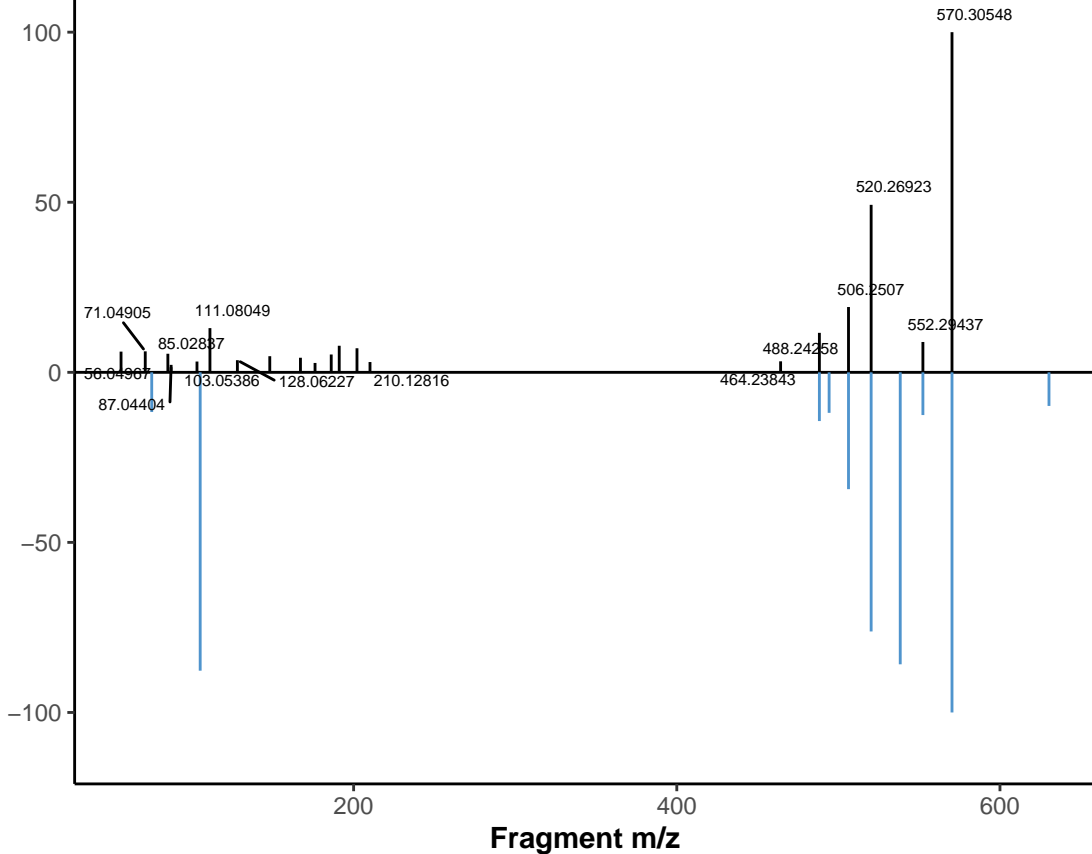

Supplement: Supplementary material 2 — MS identification chart of Xiaojin Pill ingredients. [file Data_Sheet_3.zip › Supplementary Material S2/Positive-7293.pdf]

# Yunaconitine

Relative intensity

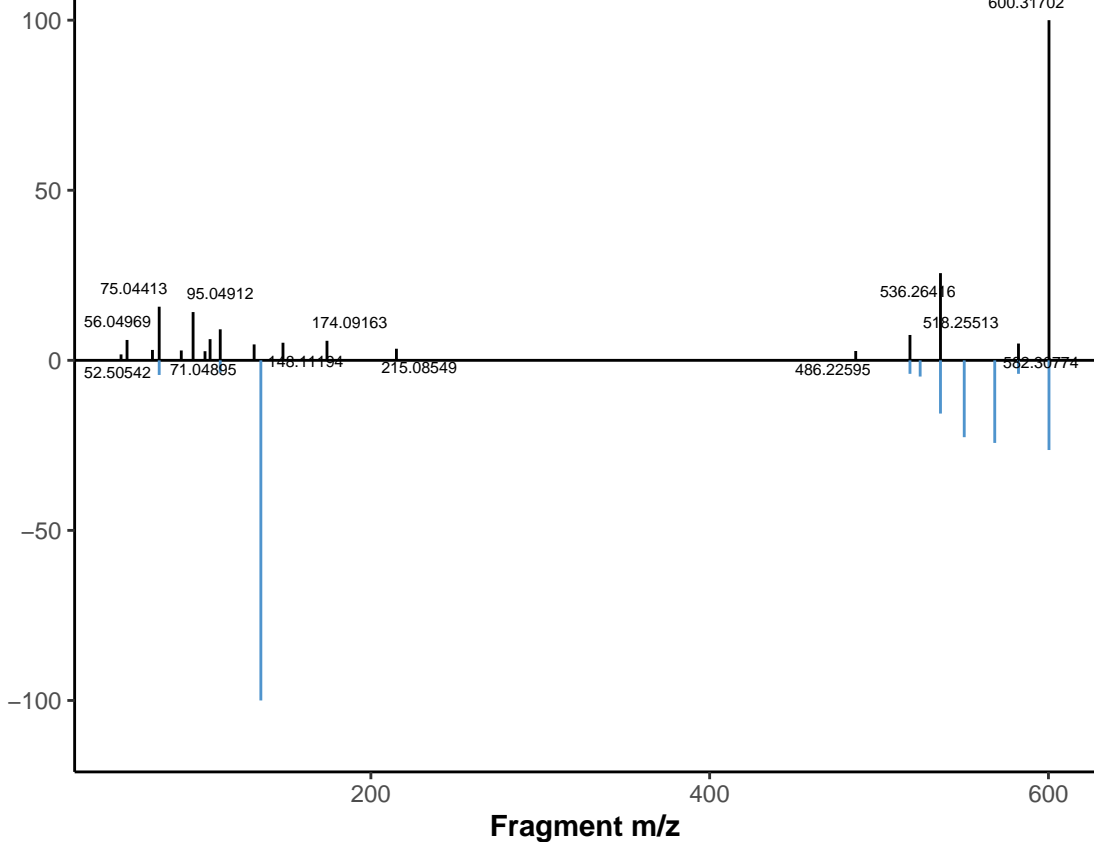

Supplement: Supplementary material 2 — MS identification chart of Xiaojin Pill ingredients. [file Data_Sheet_3.zip › Supplementary Material S2/Positive-7470.pdf]

# L(-)-Carnitine

Relative intensity

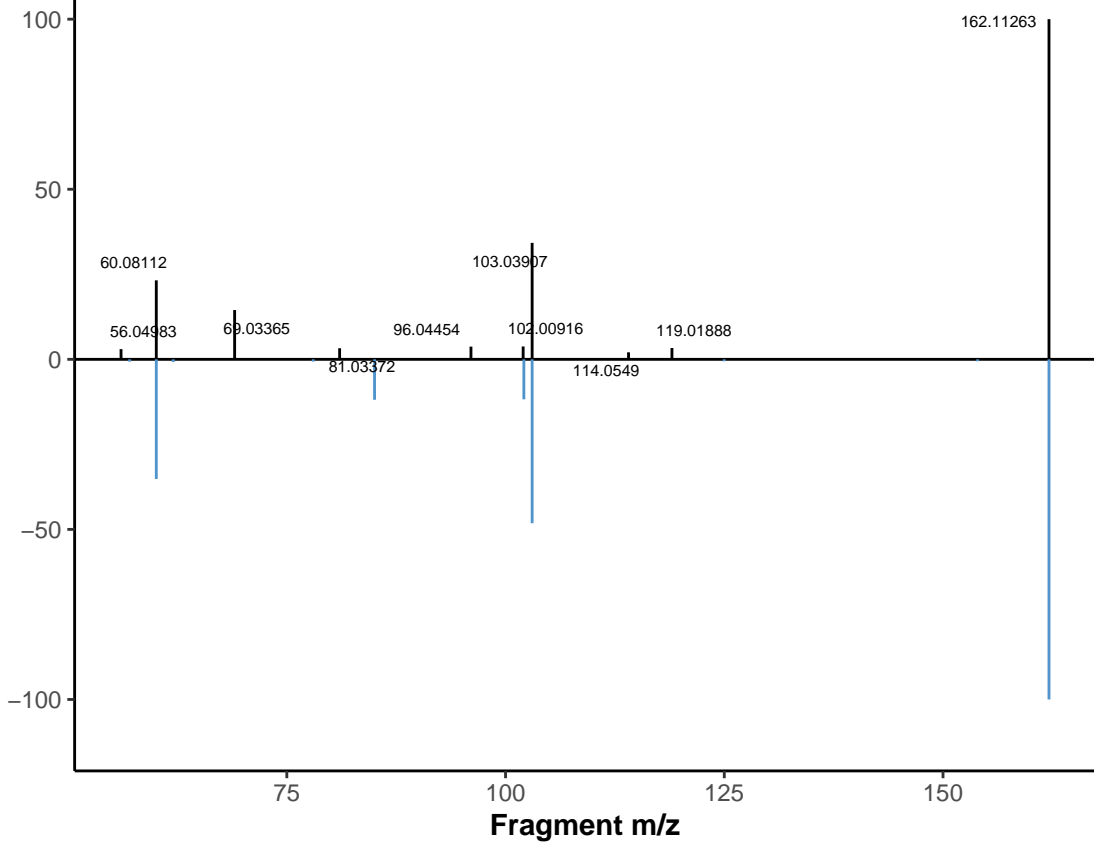

Supplement: Supplementary material 2 — MS identification chart of Xiaojin Pill ingredients. [file Data_Sheet_3.zip › Supplementary Material S2/Positive-883.pdf]

# L-Phenylalanine

Relative intensity

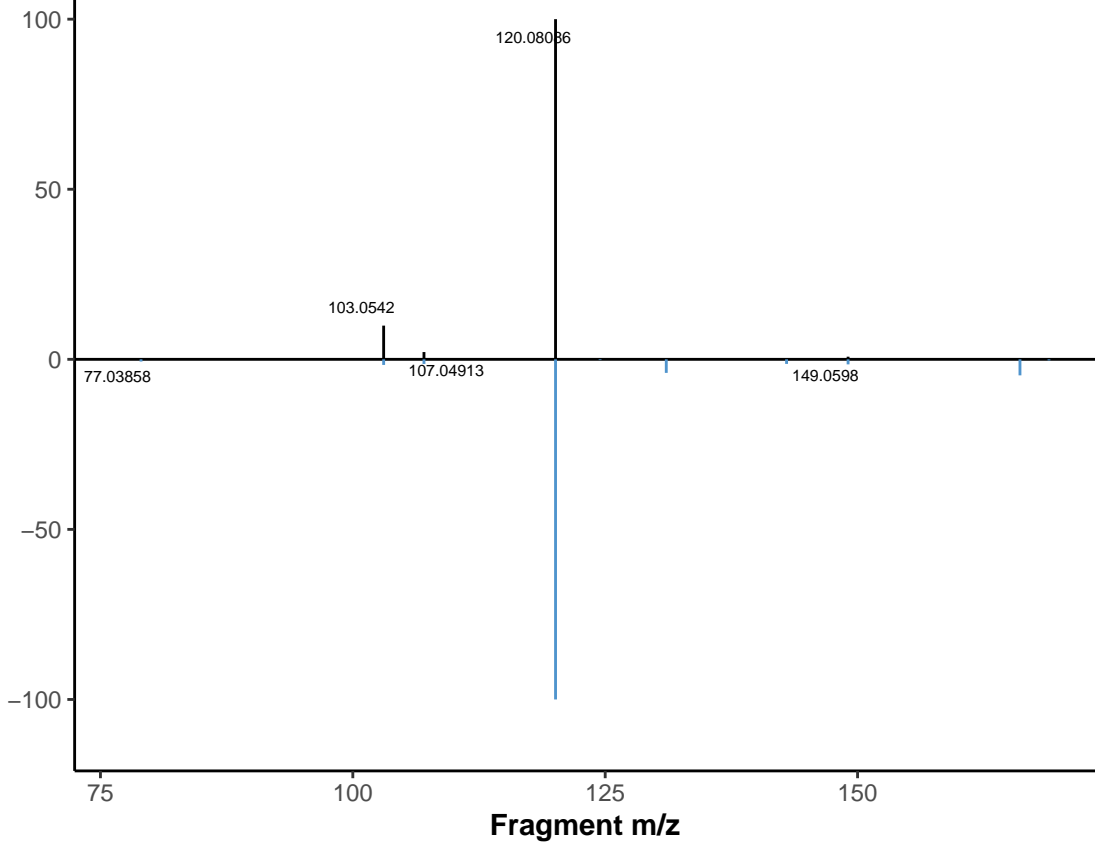

Supplement: Supplementary material 2 — MS identification chart of Xiaojin Pill ingredients. [file Data_Sheet_3.zip › Supplementary Material S2/Positive-938.pdf]

# Perillartine

Relative intensity

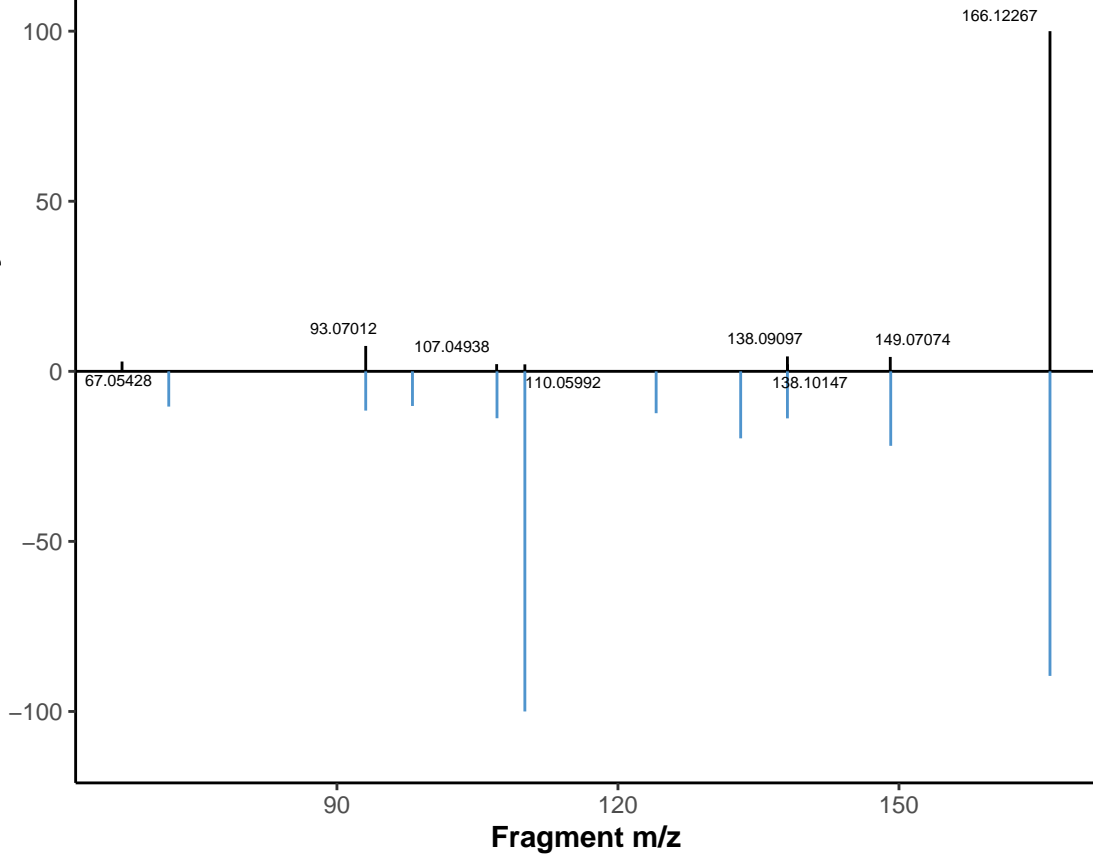

Supplement: Supplementary material 2 — MS identification chart of Xiaojin Pill ingredients. [file Data_Sheet_3.zip › Supplementary Material S2/Positive-942.pdf]

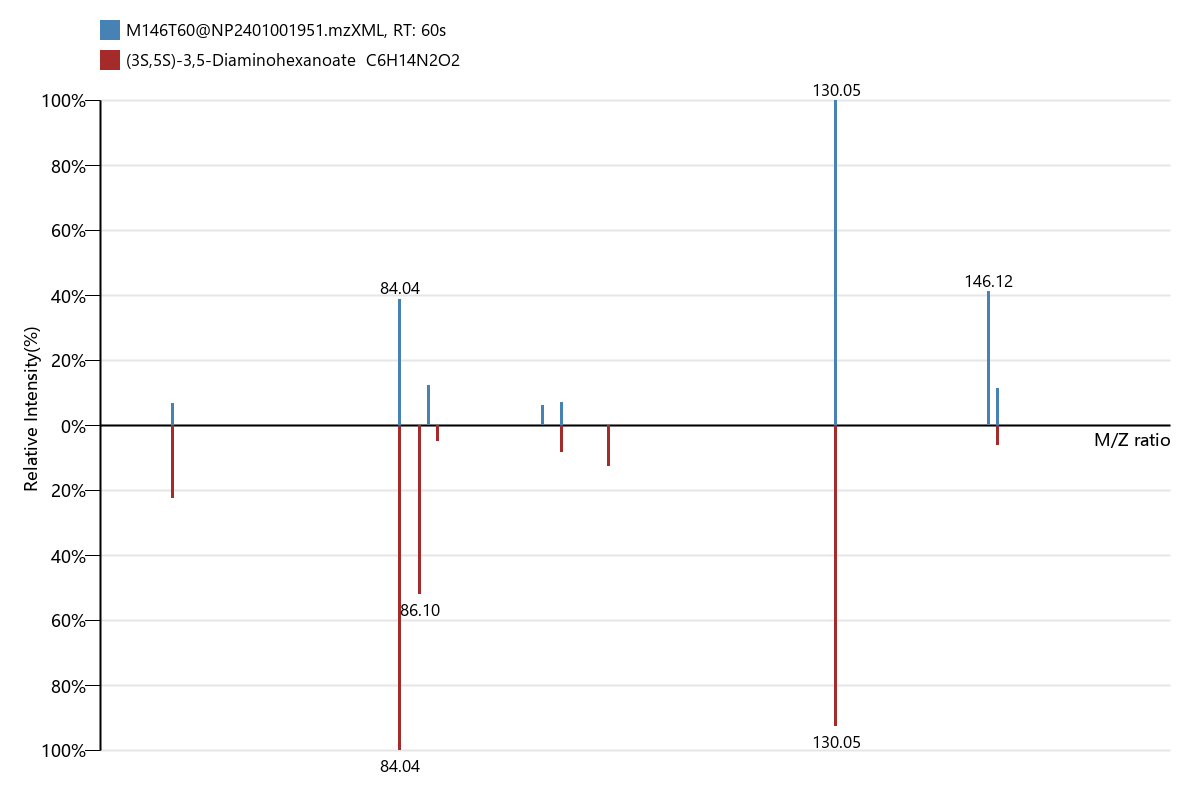

Supplement: Supplementary material 4 — Original identification chart of serum metabolites. [file Data_Sheet_5.zip › Supplementary Material S4/Sham and Model Group/(3S,5S)-3,5-Diaminohexanoate.png]

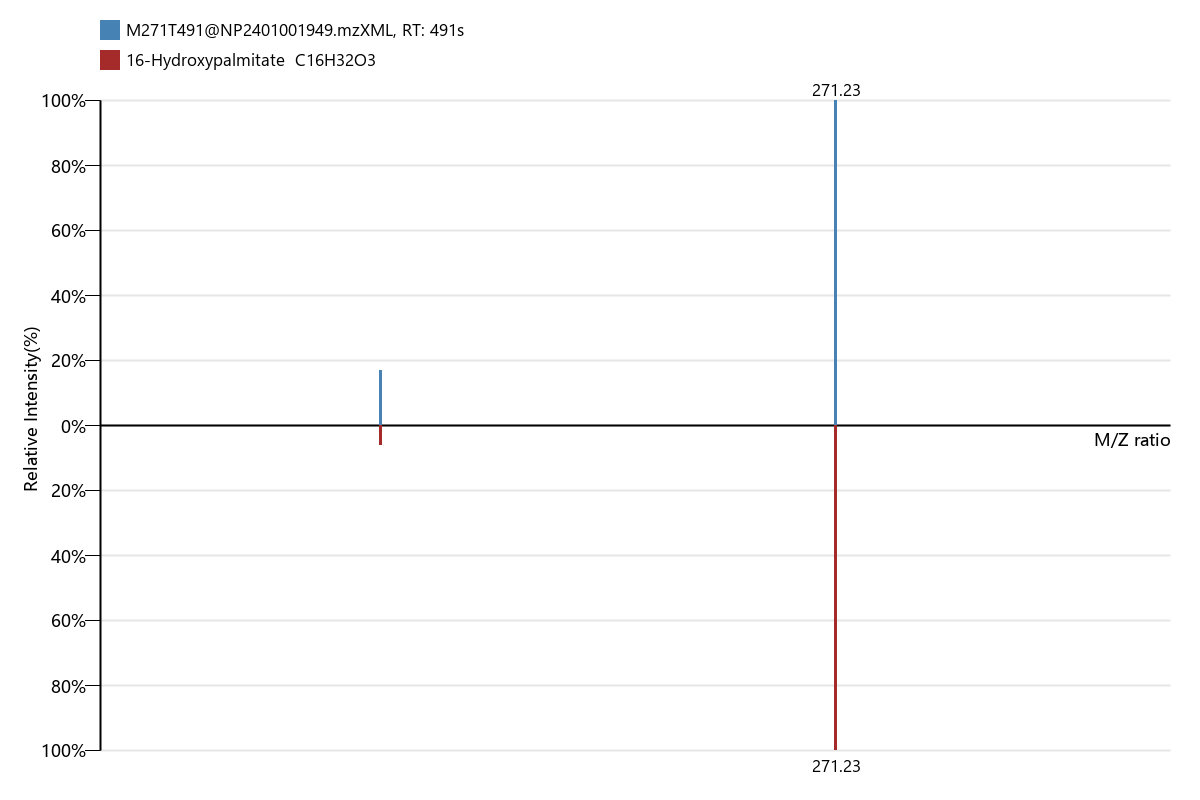

Supplement: Supplementary material 4 — Original identification chart of serum metabolites. [file Data_Sheet_5.zip › Supplementary Material S4/Sham and Model Group/16-Hydroxypalmitate.png]

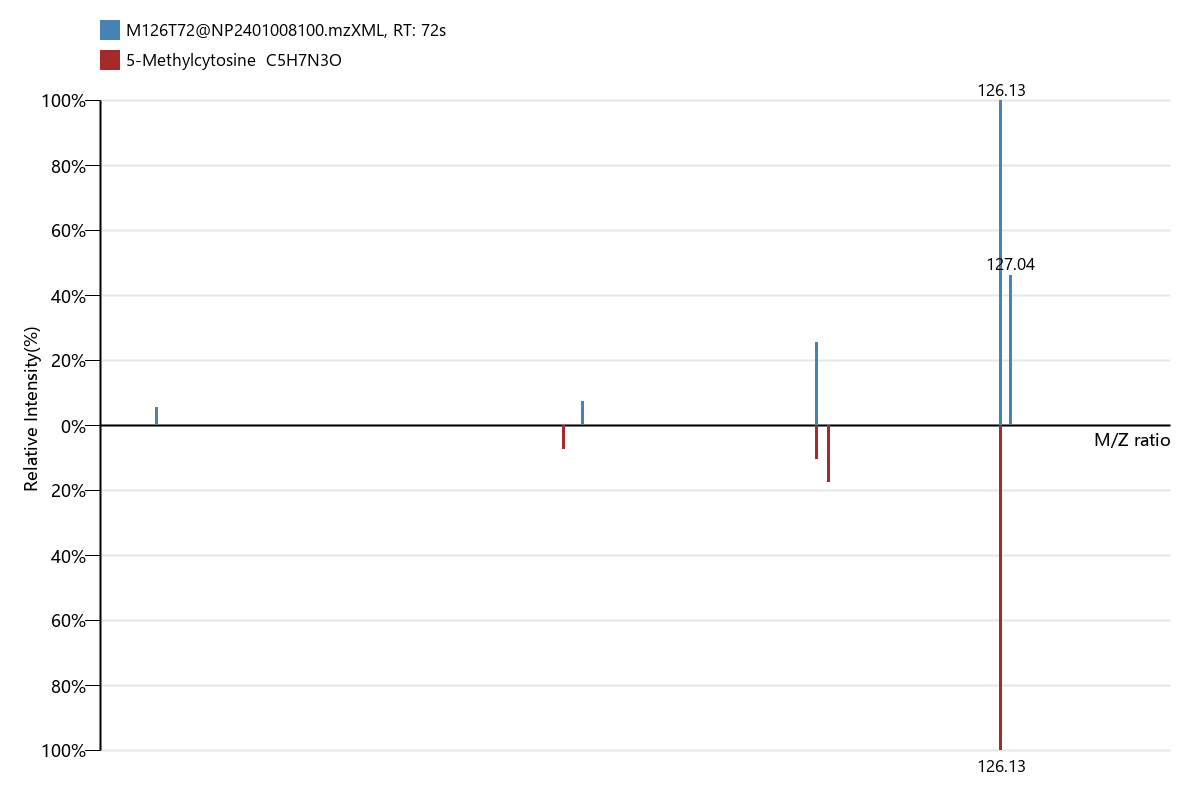

Supplement: Supplementary material 4 — Original identification chart of serum metabolites. [file Data_Sheet_5.zip › Supplementary Material S4/Sham and Model Group/5-Methylcytosine.png]

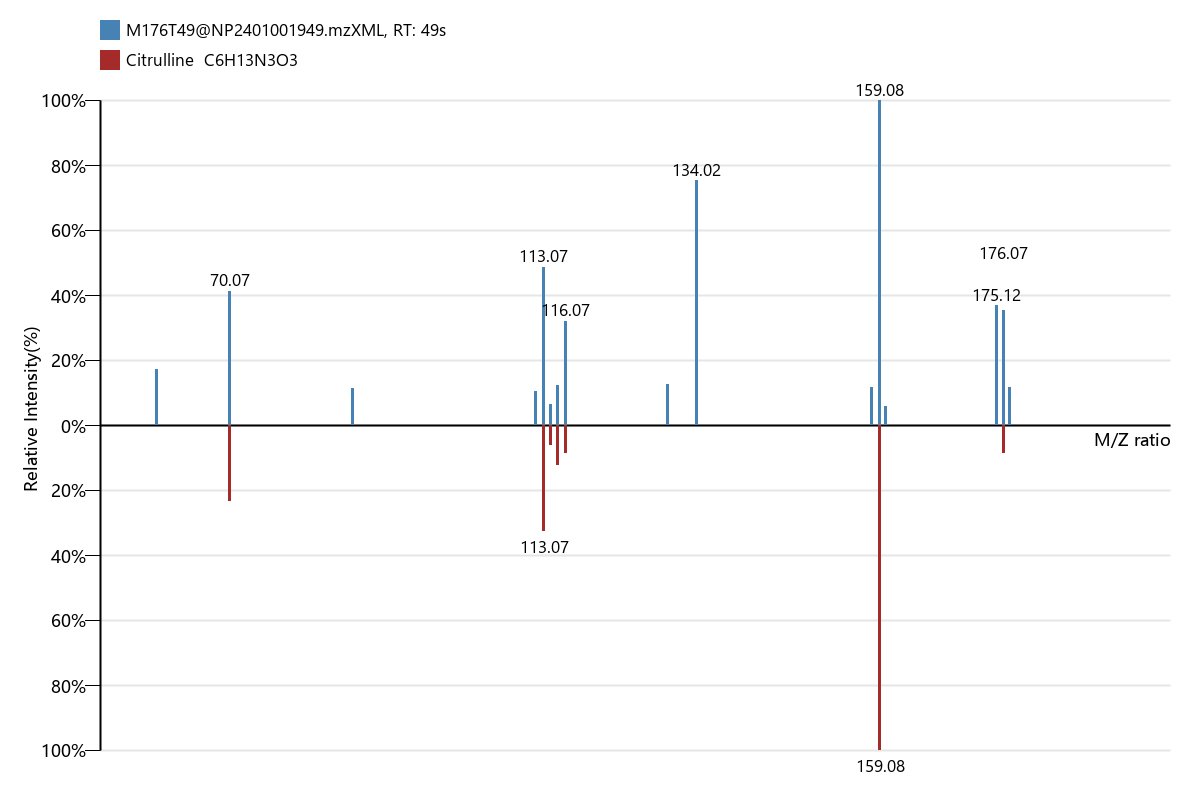

Supplement: Supplementary material 4 — Original identification chart of serum metabolites. [file Data_Sheet_5.zip › Supplementary Material S4/Sham and Model Group/Citrulline.png]

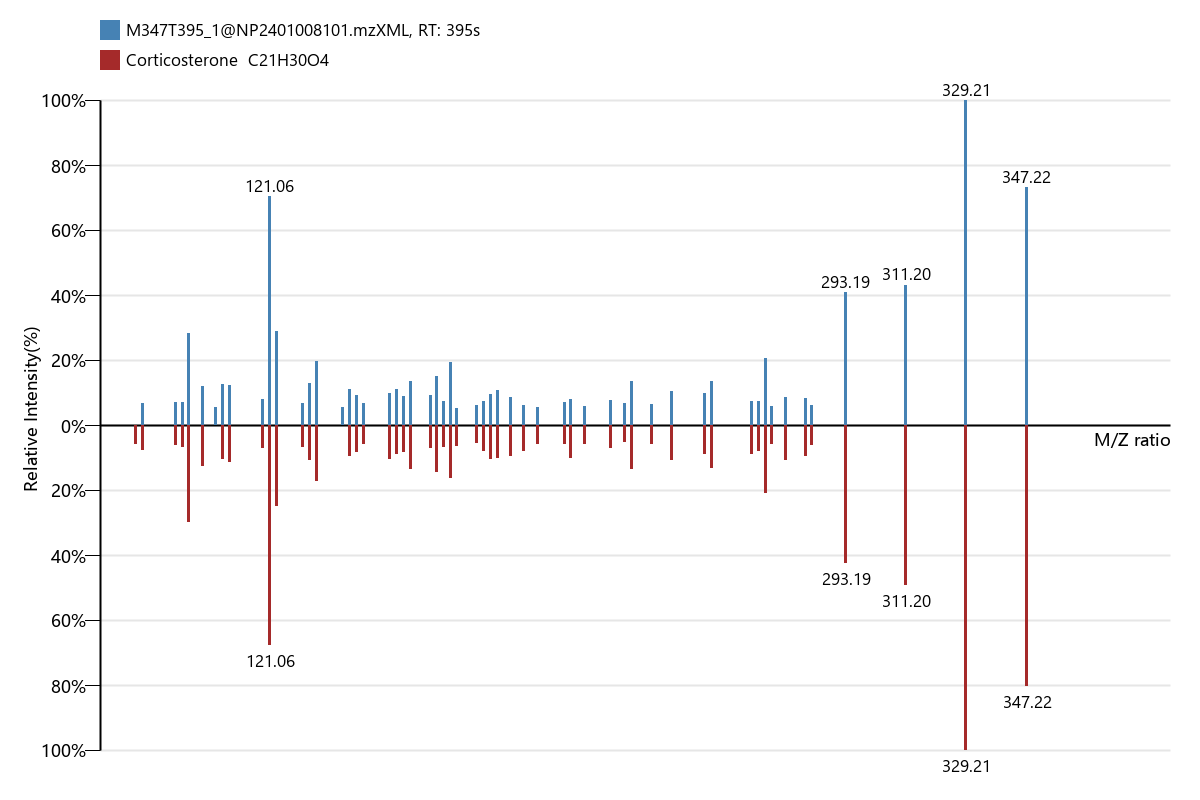

Supplement: Supplementary material 4 — Original identification chart of serum metabolites. [file Data_Sheet_5.zip › Supplementary Material S4/Sham and Model Group/Corticosterone.png]

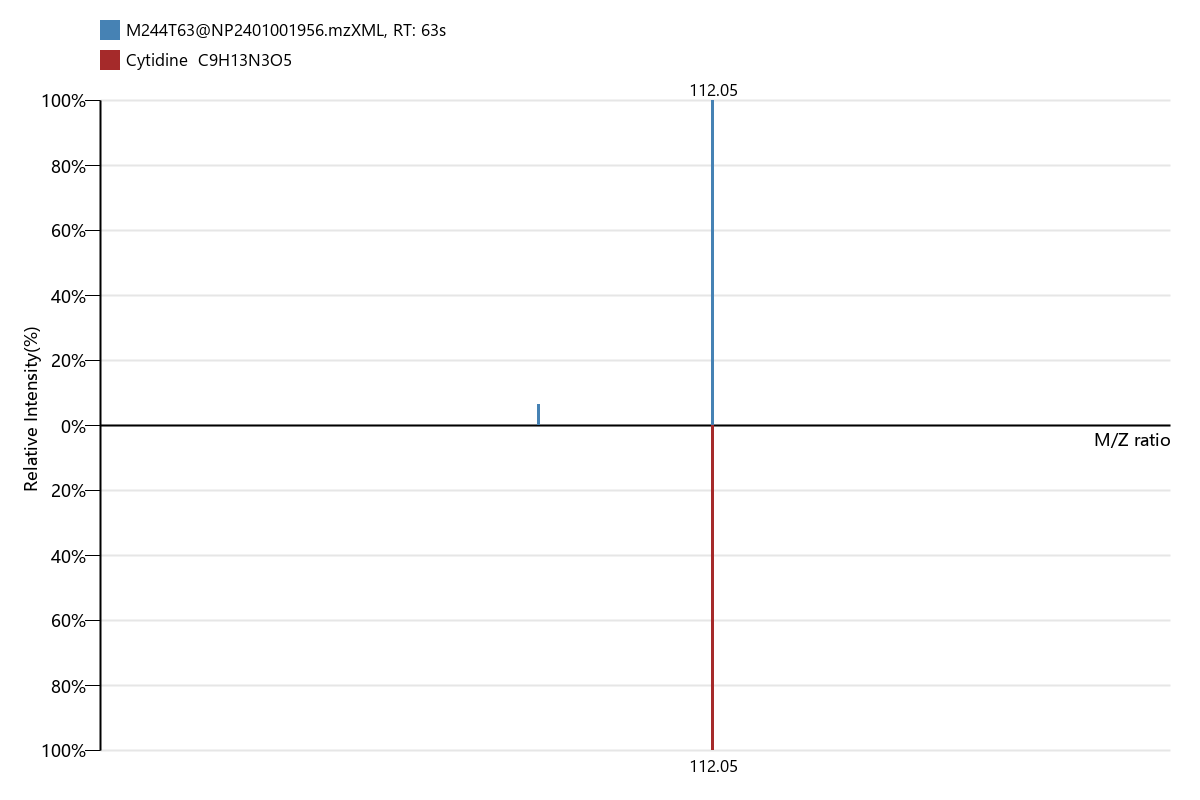

Supplement: Supplementary material 4 — Original identification chart of serum metabolites. [file Data_Sheet_5.zip › Supplementary Material S4/Sham and Model Group/Cytidine.png]

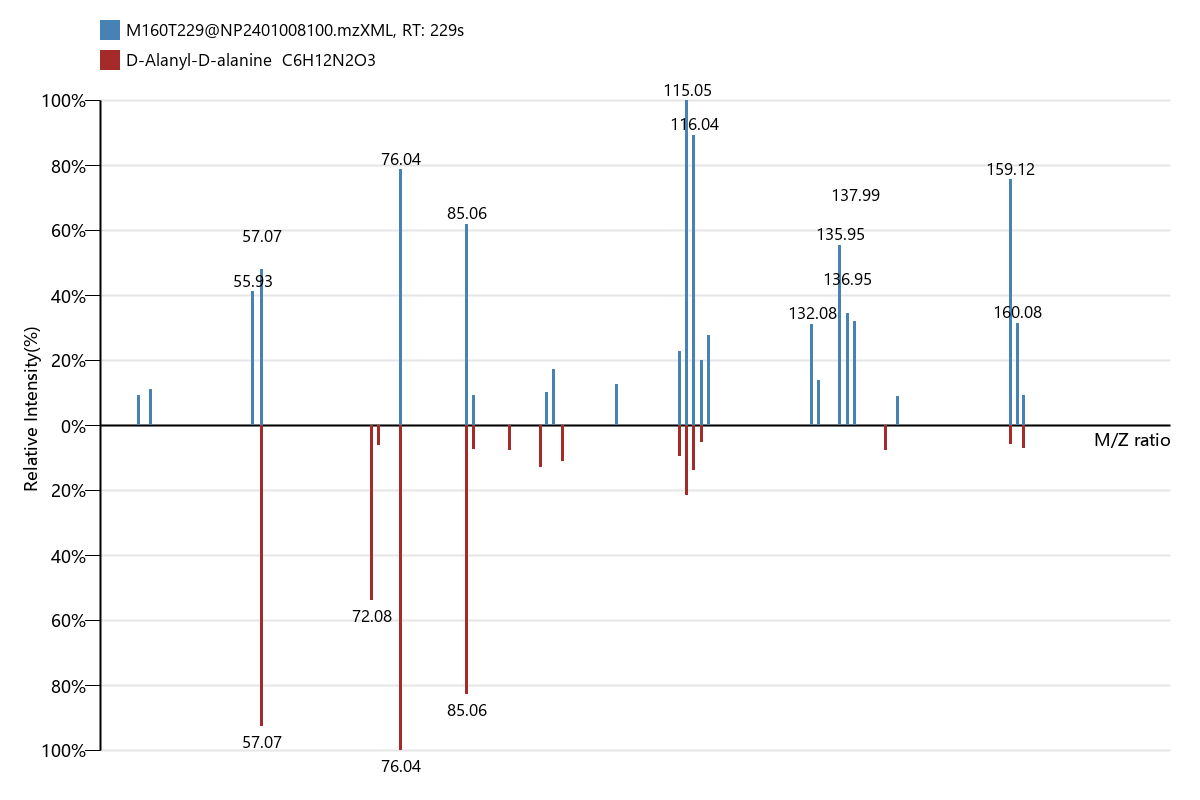

Supplement: Supplementary material 4 — Original identification chart of serum metabolites. [file Data_Sheet_5.zip › Supplementary Material S4/Sham and Model Group/D-Alanyl-D-alanine.png]

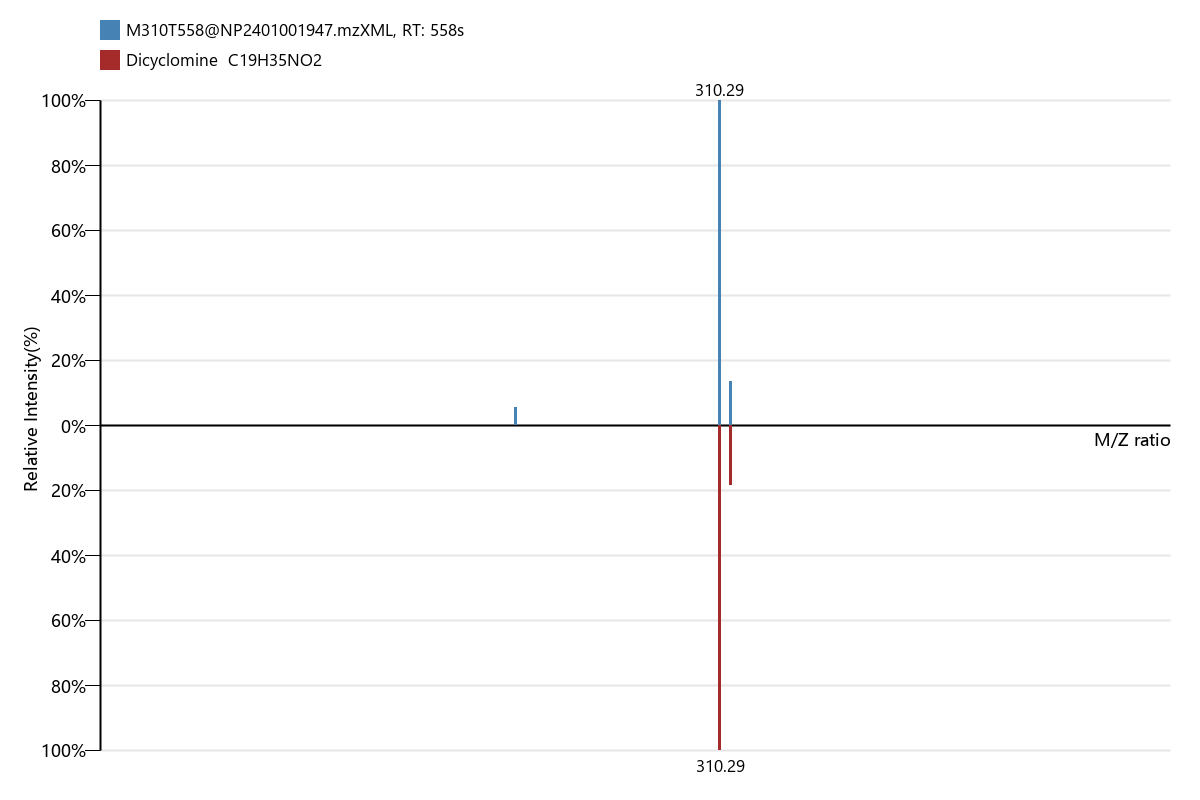

Supplement: Supplementary material 4 — Original identification chart of serum metabolites. [file Data_Sheet_5.zip › Supplementary Material S4/Sham and Model Group/Dicyclomine.png]

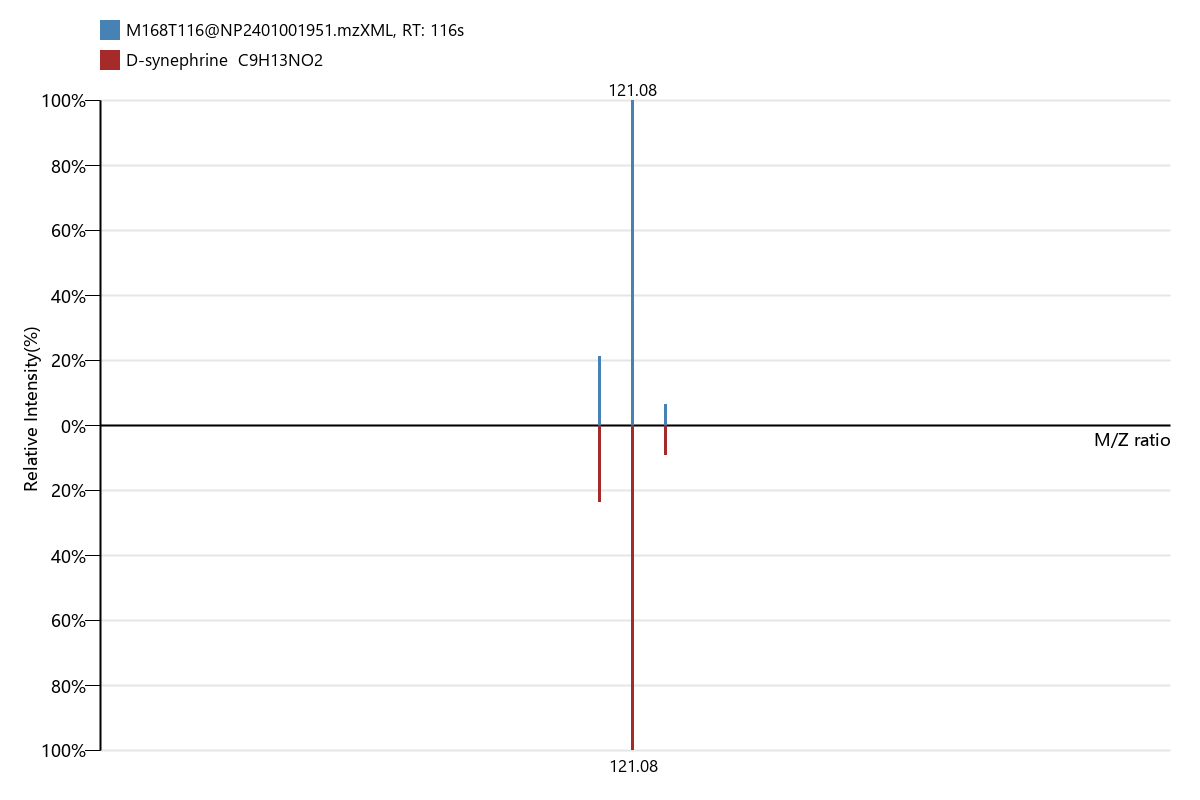

Supplement: Supplementary material 4 — Original identification chart of serum metabolites. [file Data_Sheet_5.zip › Supplementary Material S4/Sham and Model Group/D-synephrine.png]
