# Supplementary material for: Exploring the potential mechanism of Xiaojin Pill therapy for benign prostatic hyperplasia through metabolomics and gut microbiota analysis
Source: Front Microbiol. 2024 Aug 21;15:1431954. doi: 10.3389/fmicb.2024.1431954 (PMC11371748; doi:10.3389/fmicb.2024.1431954)
Supplement: Supplementary material 3 — Potential biomarker and correlation analysis table. [file Data_Sheet_4.docx]

Supplementary Material

Exploring the Potential Mechanism of Xiaojin Pill Therapy for Benign Prostatic Hyperplasia Based on Metabolomics and Gut Microbiota Analysis

**Yuying Yang^1,2^, Yunyun Quan^2^, Yunteng Liu^3^, Juhua Yang^3^, Keyu Chen^4^, Xiaozhou You^2^, Hua Hua^2,3^, Liangchun Yan^2^, Junning Zhao^2*^ and Jianbo Wang^1,2,4*^**

^1^School of Pharmacy, Southwest Medical University, Luzhou 646000, China;

^2^Sichuan Institute for Translational Chinese Medicine, SICHUAN ACADEMY OF CHINESE MEDICINE SCIENCES, Key Laboratory of Biological Evaluation of Translational Chinese Medicine（TCM） Quality of National Administration of TCM, Sichuan Key Laboratory of Translational Medicine of TCM, Sichuan Authentic Medicine System Development Engineering Technology Research Center, Sichuan Authentic Medicine Formation Principle and Quality Evaluation Engineering Research Center, Chengdu, China;

^3^Chengdu University, Chengdu 6100413, China;

^4^Shaanxi University of Traditional Chinese Medicine, Xianyang 712046, China）

*** Correspondence: Jianbo WANG**, **E-mail：[yyswjb@fmmu.edu.cn](mailto:yyswjb@fmmu.edu.cn)；Junning ZHAO, E-mail：[zarmy@189.cn](mailto:zarmy@189.cn)**

TABLE 2 Potential Biomarkers in Rat Serum between Sham and Model Group

| Name | m/z | rt（s） | Formula | -log10（P） | VIP | log2（FC） |
| --- | --- | --- | --- | --- | --- | --- |
| 5-Methylcytosine | 126.0657 | 72.4 | C₅H₇N₃O | 2.38 | 2.45 | 0.74 |
| Pipecolic acid | 130.0493 | 72.9 | C₆H₁₁NO₂ | 1.61 | 2.1 | 0.37 |
| (3S,5S)-3,5-Diaminohexanoate | 146.117 | 60.3 | C₆H₁₄N₂O₂ | 1.33 | 1.9 | -1.02 |
| Uracil 5-carboxylate | 156.9644 | 36 | C₅H₄N₂O₄ | 1.38 | 1.95 | 0.26 |
| D-Alanyl-D-alanine | 160.0962 | 228.6 | C₆H₁₂N₂O₃ | 1.39 | 1.96 | -0.65 |
| D-synephrine | 168.0911 | 115.6 | C₉H₁₃NO₂ | 1.65 | 2.02 | -0.6 |
| Citrulline | 176.1022 | 49.4 | C₆H₁₃N₃O₃ | 1.72 | 2.18 | -0.38 |
| Cytidine | 244.0913 | 63.2 | C₉H₁₃N₃O₅ | 1.42 | 1.92 | 0.51 |
| N,N-Dimethylsphing-4-enine | 310.3077 | 555.6 | C₂₀H₄₁NO₂ | 1.53 | 1.99 | 1.01 |
| Dicyclomine | 310.2722 | 557.7 | C₁₉H₃₅NO₂ | 2 | 2.23 | -0.96 |
| Corticosterone | 347.2185 | 394.8 | C₂₁H₃₀O₄ | 2.2 | 2.34 | 0.62 |
| 16-Hydroxypalmitate | 271.2247 | 491.1 | C₁₆H₃₂O₃ | 1.94 | 2.28 | -0.71 |

TABLE 3 Potential Biomarkers in Rat Serum between XJP-H and Model Group

| Name | m/z | rt（s） | Formula | -log10（P） | VIP | log2（FC） |
| --- | --- | --- | --- | --- | --- | --- |
| Ethylmethylacetic acid | 102.1267 | 683.3 | C₅H₁₀O₂ | 2.25 | 2.34 | 0.84 |
| Cytosine | 112.0499 | 53.7 | C₄H₅N₃O | 1.57 | 2.03 | -0.43 |
| Taurine | 126.0214 | 49.7 | C₂H₇NO₃S | 1.46 | 1.98 | -0.65 |
| 5-Methylcytosine | 126.0657 | 72.4 | C₅H₇N₃O | 1.5 | 1.96 | 0.33 |
| 5,6-Dihydro-5-fluorouracil | 133.0312 | 72.6 | C₄H₅FN₂O₂ | 1.31 | 1.86 | 0.37 |
| (3S)-3,6-Diaminohexanoate | 146.0919 | 54.4 | C₆H₁₄N₂O₂ | 1.42 | 1.95 | -0.33 |
| 2-Hydroxyglutarate | 148.0422 | 79.3 | C₅H₈O₅ | 1.48 | 1.97 | -0.43 |
| (+)-cis-Isopulegone | 153.138 | 120.9 | C₁₀H₁₆O | 1.55 | 2.2 | -1.48 |
| L-Phenylalanine | 166.0855 | 115.5 | C₉H₁₁NO₂ | 1.99 | 2.24 | 0.28 |
| Normetanephrine | 166.0837 | 54.2 | C₉H₁₃NO₃ | 2 | 2.23 | 0.94 |
| Dihydroxyacetone phosphate | 171.148 | 38.2 | C₃H₇O₆P | 1.91 | 2.23 | 0.74 |
| L-Arginine | 175.1183 | 44.3 | C₆H₁₄N₄O₂ | 1.77 | 2.17 | -1.94 |
| 4-Hydroxycinnamoylagmatine | 276.1426 | 77.9 | C₁₄H₂₀N₄O₂ | 1.84 | 2.37 | 2.4 |
| Indican | 294.9369 | 44.9 | C₁₄H₁₇NO₆ | 1.8 | 2.15 | -1.21 |
| GMP | 362.9225 | 44.9 | C₁₀H₁₄N₅O₈P | 1.35 | 2.06 | -1.82 |
| 3-Epiecdysone | 464.2787 | 394.9 | C₂₇H₄₄O₆ | 2.2 | 2.33 | 2.45 |
| Avermectin B1b aglycone | 570.3556 | 530 | C₃₃H₄₆O₈ | 1.44 | 1.95 | -0.36 |
| Uracil | 111.0186 | 70.3 | C₄H₄N₂O₂ | 2.57 | 2.43 | -0.25 |
| alpha-Ketoisovaleric acid | 115.0387 | 71.1 | C₅H₈O₃ | 2.82 | 2.54 | 1.78 |
| Citric acid | 190.9244 | 33.3 | C₆H₈O₇ | 1.47 | 2.01 | 1.21 |

TABLE 4 The correlation between Serum factors and Gut microbes (|r|＞0.6, P<0.05).

| Serum factors | Gut microbes | r | p |
| --- | --- | --- | --- |
| PSA | Lactobacillus | 0.61 | 0.0077 |
| PSA | Fusicatenibacter | -0.70 | 0.0011 |
| bFGF | Lactobacillus | 0.75 | 0.0004 |
| bFGF | Dorea | 0.61 | 0.0076 |
| bFGF | Frisingicoccus | 0.62 | 0.0062 |
| bFGF | Prevotellaceae_NK3B31_group | 0.72 | 0.0008 |
| DHT | Lactobacillus | 0.69 | 0.0014 |
| DHT | Dorea | 0.63 | 0.0048 |
| DHT | Prevotellaceae_NK3B31_group | 0.67 | 0.0022 |
| EGF | Fusicatenibacter | -0.63 | 0.0052 |
| EGF | Lactobacillus | 0.61 | 0.0075 |
| EGF | Dorea | 0.73 | 0.0006 |
| EGF | Frisingicoccus | 0.62 | 0.0062 |
| EGF | Prevotellaceae_NK3B31_group | 0.64 | 0.0045 |
| VEGF | Fusicatenibacter | -0.64 | 0.0043 |
| VEGF | Lactobacillus | 0.64 | 0.0045 |
| VEGF | Dorea | 0.72 | 0.0007 |

TABLE 5 The correlation between Differential metabolites and Gut microbes (|r|＞0.6, P<0.05).

| Differential metabolites | Gut microbes | r | p |
| --- | --- | --- | --- |
| Avermectin B1b aglycone | Romboutsia | -0.63 | 0.0121 |
| Taurine | Eubacterium__brachy_group | -0.63 | 0.0121 |
| Taurine | Chujaibacter | -0.77 | 0.0008 |
| Taurine | Enterobacter | -0.77 | 0.0008 |
| L-Arginine | Romboutsia | -0.61 | 0.0156 |
| Uracil | Romboutsia | -0.83 | 0.0001 |
| Cytosine | Romboutsia | -0.65 | 0.0082 |
| Cytosine | Enterorhabdus | -0.64 | 0.0103 |
| (3S)-3,6-Diaminohexanoate | Romboutsia | -0.69 | 0.0048 |
| D-Alanyl-D-alanine | Chujaibacter | 0.66 | 0.0069 |
| Dicyclomine | Lactobacillus | 0.63 | 0.0127 |
| Normetanephrine | Enterorhabdus | 0.69 | 0.0045 |
| Normetanephrine | Eubacterium__brachy_group | 0.65 | 0.0083 |
| 4-Hydroxycinnamoylagmatine | Romboutsia | 0.65 | 0.0087 |
| Cytidine | Enterobacter | -0.61 | 0.0161 |
| 5-Methylcytosine | Chujaibacter | -0.63 | 0.0121 |
| 5-Methylcytosine | Lactobacillus | -0.74 | 0.0018 |
| 5-Methylcytosine | Prevotellaceae_NK3B31_group | -0.61 | 0.0156 |
| Dihydroxyacetone phosphate | Romboutsia | 0.63 | 0.0121 |
| Dihydroxyacetone phosphate | Dorea | -0.66 | 0.0070 |
| L-Phenylalanine | Enterorhabdus | 0.65 | 0.0082 |
| 5,6-Dihydro-5-fluorouracil | Romboutsia | 0.63 | 0.0121 |
| 5,6-Dihydro-5-fluorouracil | Frisingicoccus | -0.70 | 0.0037 |
| alpha-Ketoisovaleric acid | Romboutsia | 0.78 | 0.0006 |
| alpha-Ketoisovaleric acid | Dorea | -0.63 | 0.0119 |
