# Supplementary figures and images for: Exploring the potential mechanism of Xiaojin Pill therapy for benign prostatic hyperplasia through metabolomics and gut microbiota analysis (part 2 of 2)
Source: Front Microbiol. 2024 Aug 21;15:1431954. doi: 10.3389/fmicb.2024.1431954 (PMC11371748; doi:10.3389/fmicb.2024.1431954)

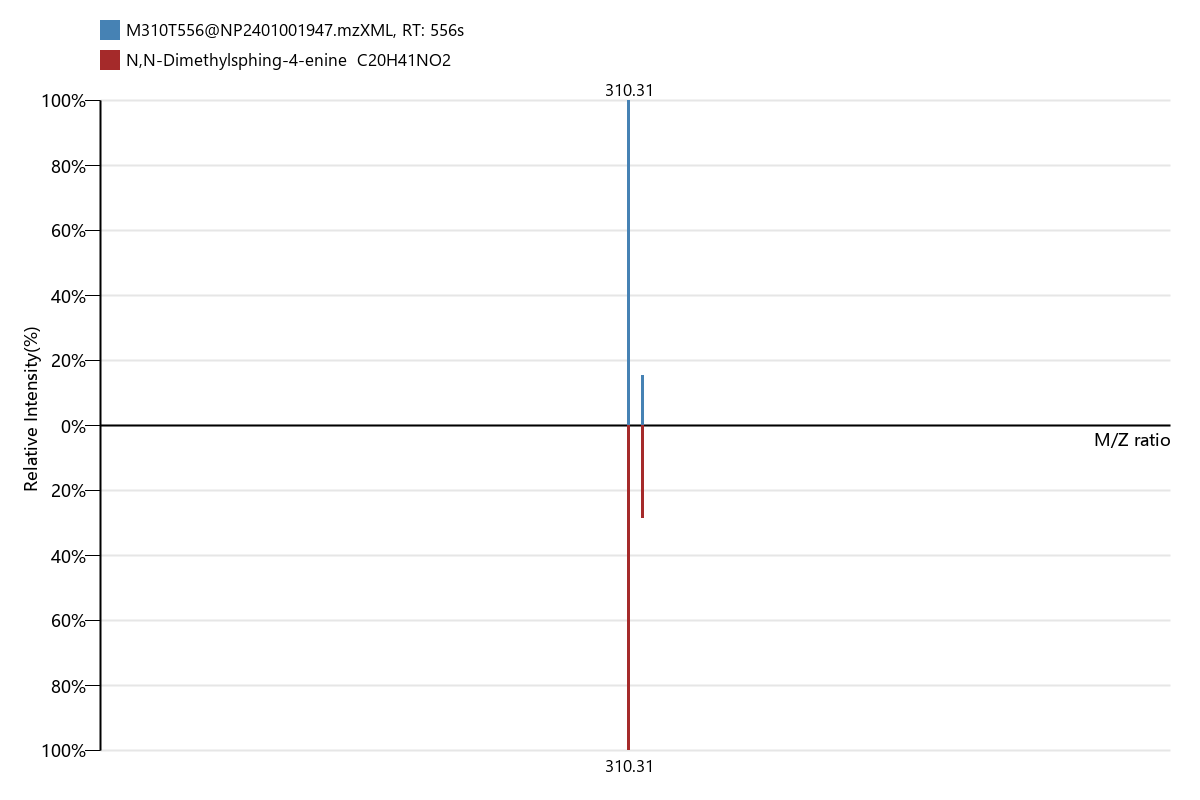

Supplement: Supplementary material 4 — Original identification chart of serum metabolites. [file Data_Sheet_5.zip › Supplementary Material S4/Sham and Model Group/N,N-Dimethylsphing-4-enine.png]

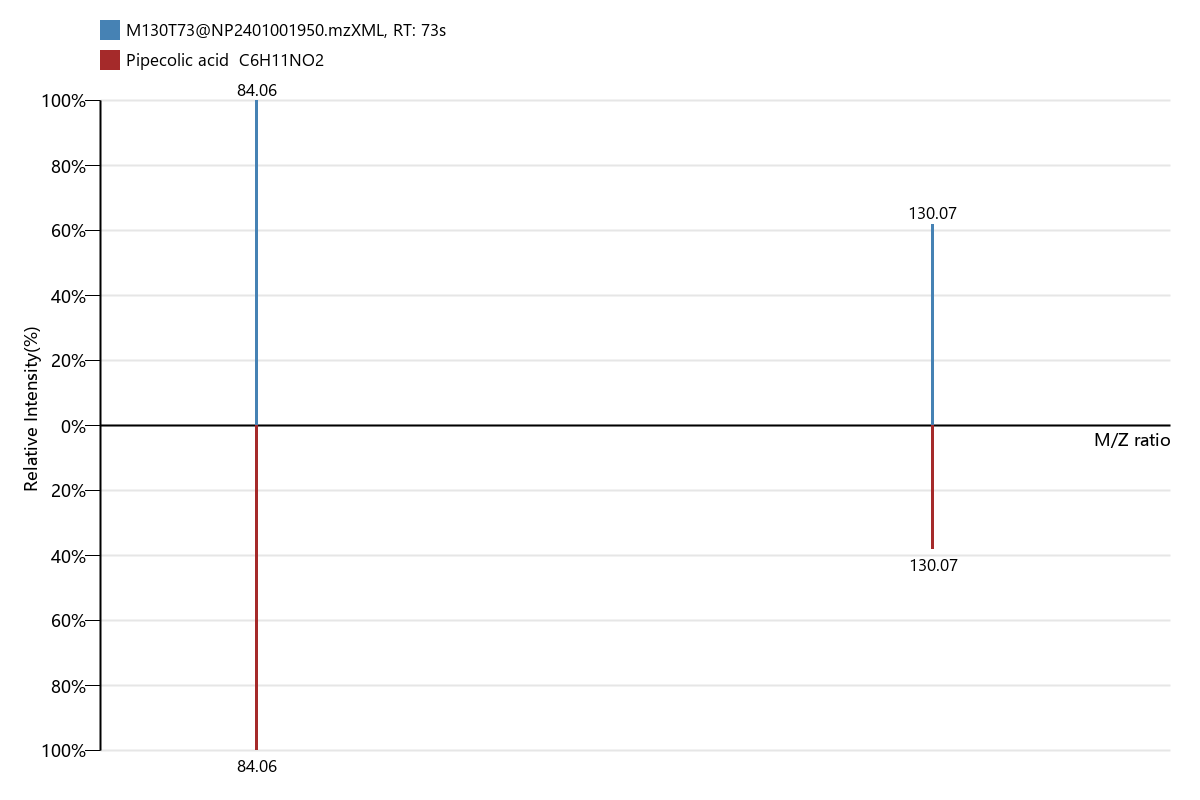

Supplement: Supplementary material 4 — Original identification chart of serum metabolites. [file Data_Sheet_5.zip › Supplementary Material S4/Sham and Model Group/Pipecolic acid.png]

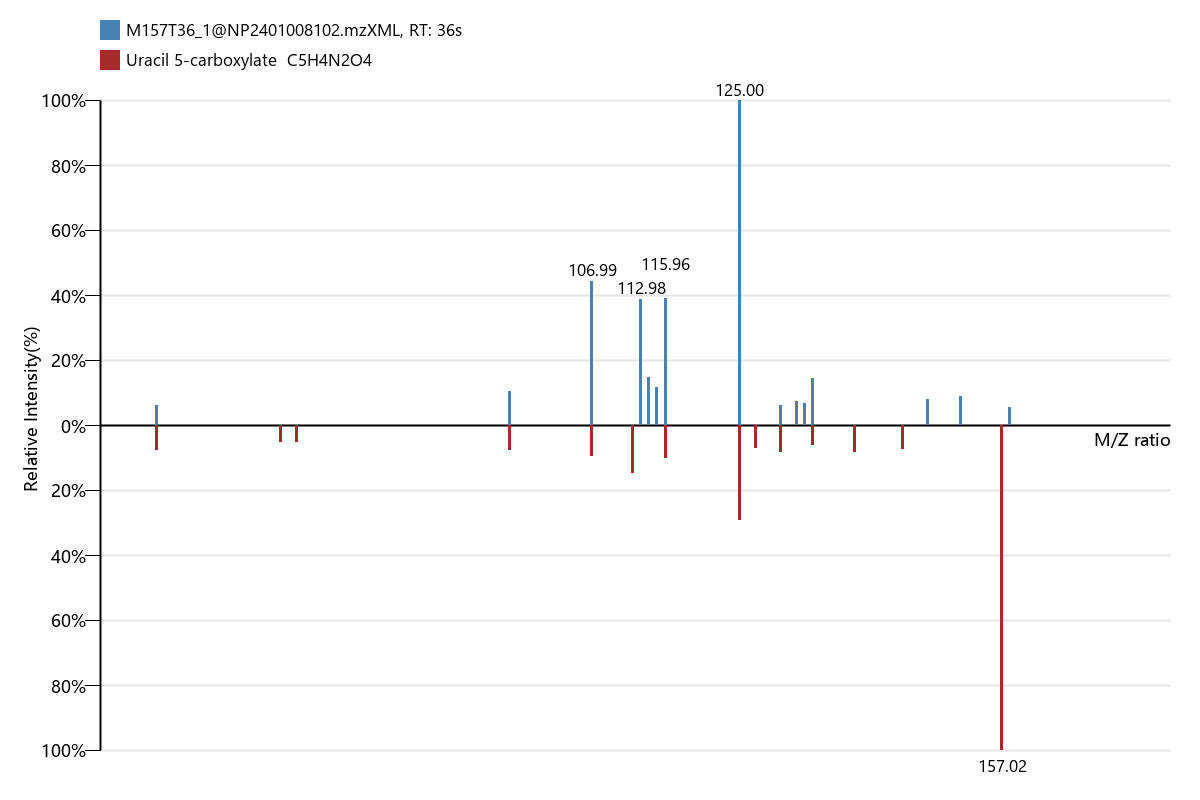

Supplement: Supplementary material 4 — Original identification chart of serum metabolites. [file Data_Sheet_5.zip › Supplementary Material S4/Sham and Model Group/Uracil 5-carboxylate.png]

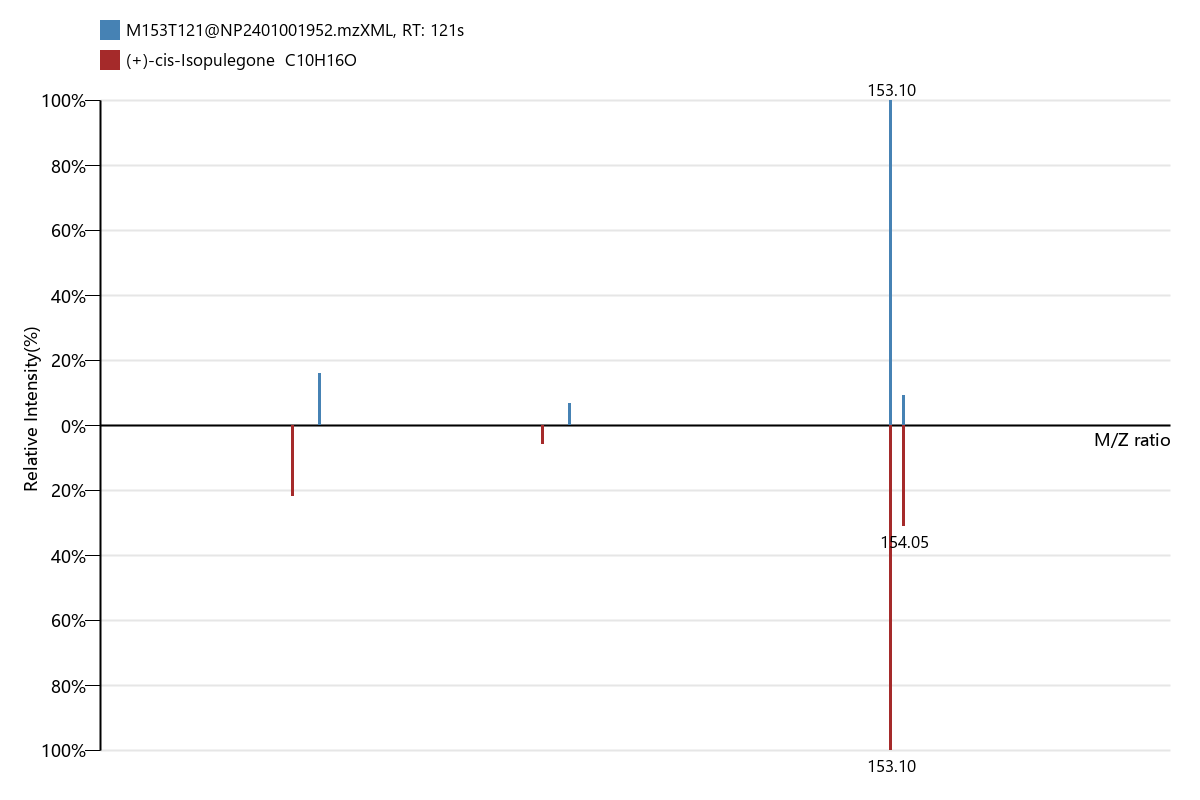

Supplement: Supplementary material 4 — Original identification chart of serum metabolites. [file Data_Sheet_5.zip › Supplementary Material S4/XJP-H and Model Group/(+)-cis-Isopulegone.png]

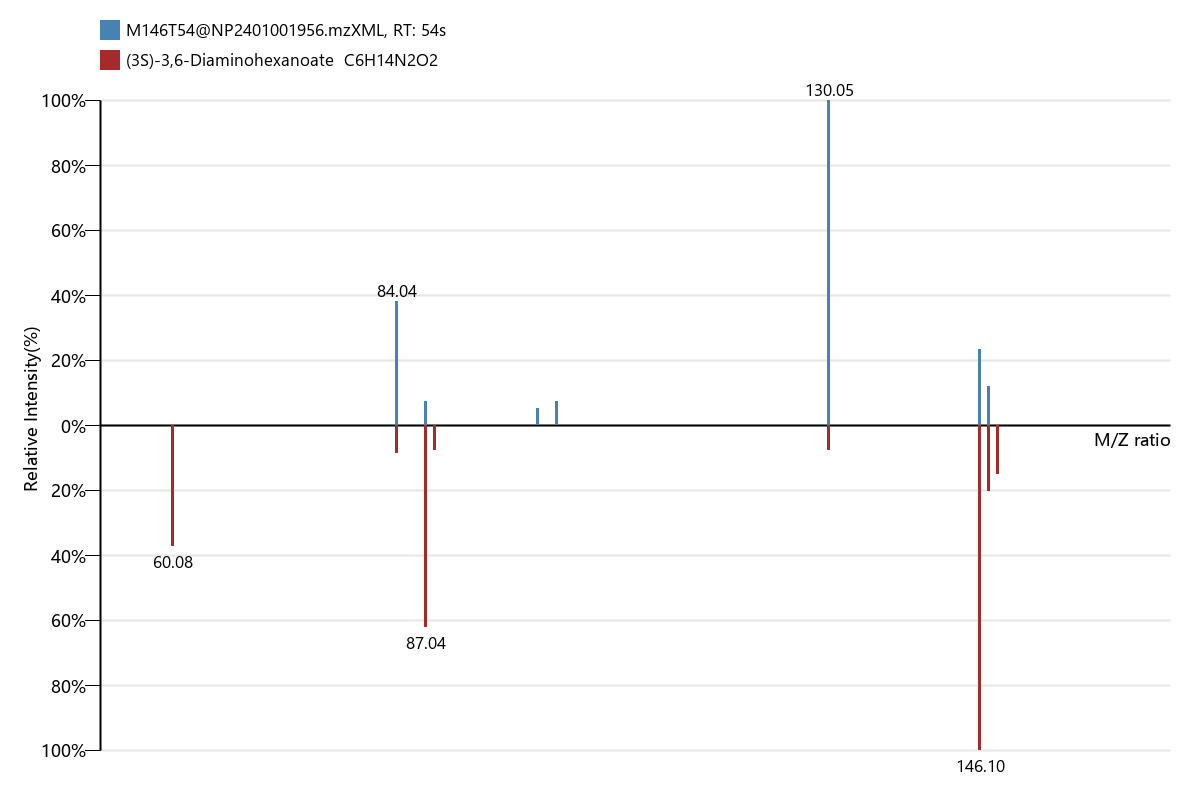

Supplement: Supplementary material 4 — Original identification chart of serum metabolites. [file Data_Sheet_5.zip › Supplementary Material S4/XJP-H and Model Group/(3S)-3,6-Diaminohexanoate.png]

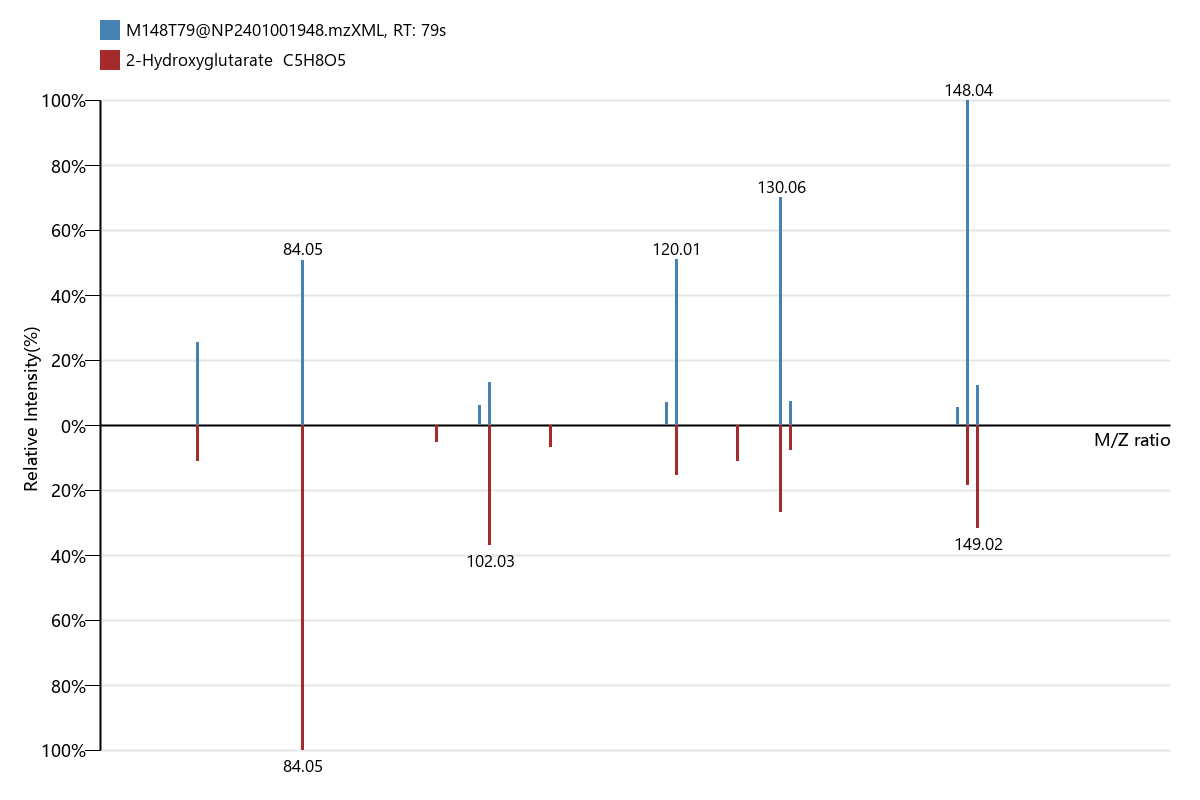

Supplement: Supplementary material 4 — Original identification chart of serum metabolites. [file Data_Sheet_5.zip › Supplementary Material S4/XJP-H and Model Group/2-Hydroxyglutarate.png]

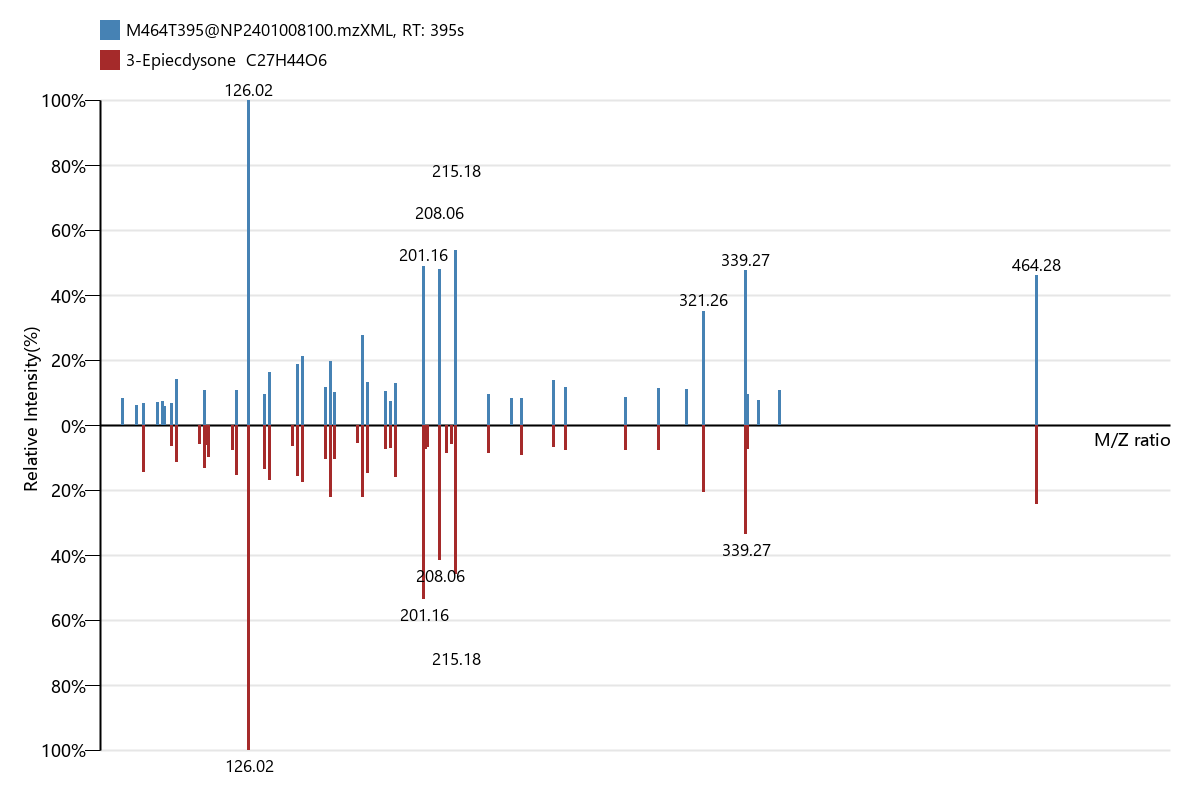

Supplement: Supplementary material 4 — Original identification chart of serum metabolites. [file Data_Sheet_5.zip › Supplementary Material S4/XJP-H and Model Group/3-Epiecdysone.png]

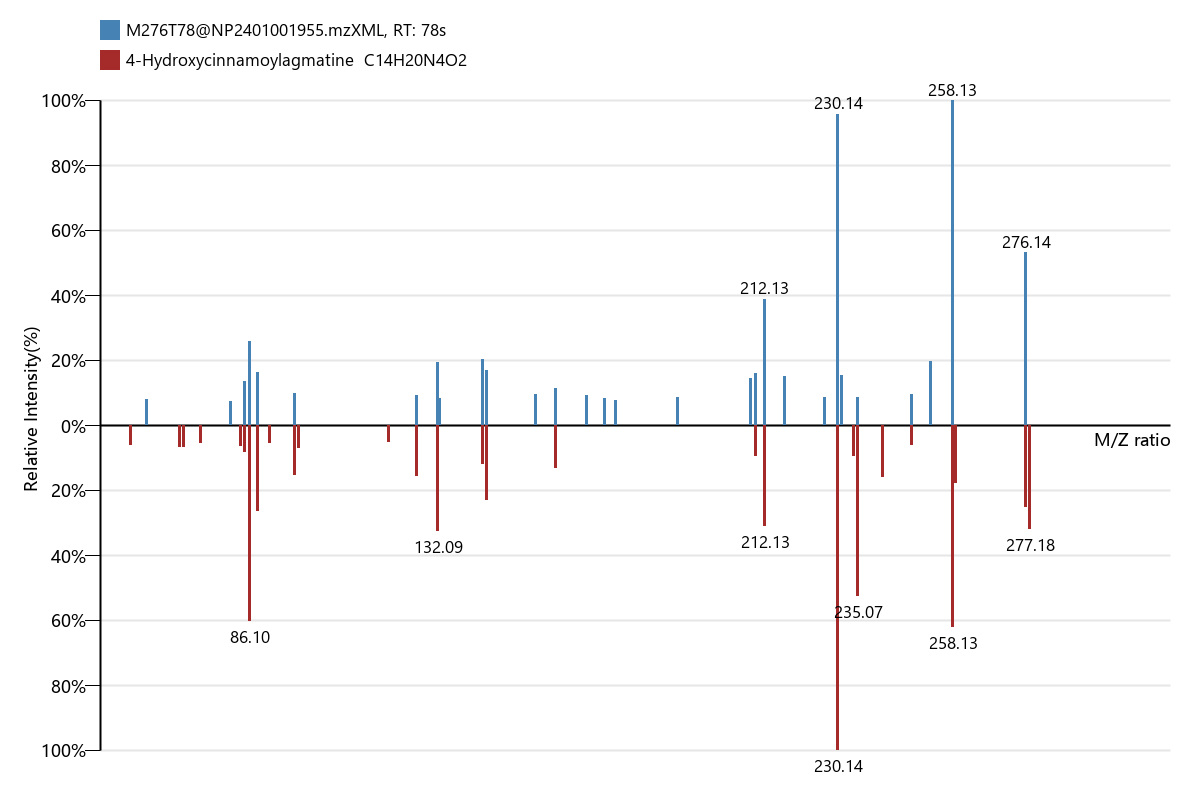

Supplement: Supplementary material 4 — Original identification chart of serum metabolites. [file Data_Sheet_5.zip › Supplementary Material S4/XJP-H and Model Group/4-Hydroxycinnamoylagmatine.png]

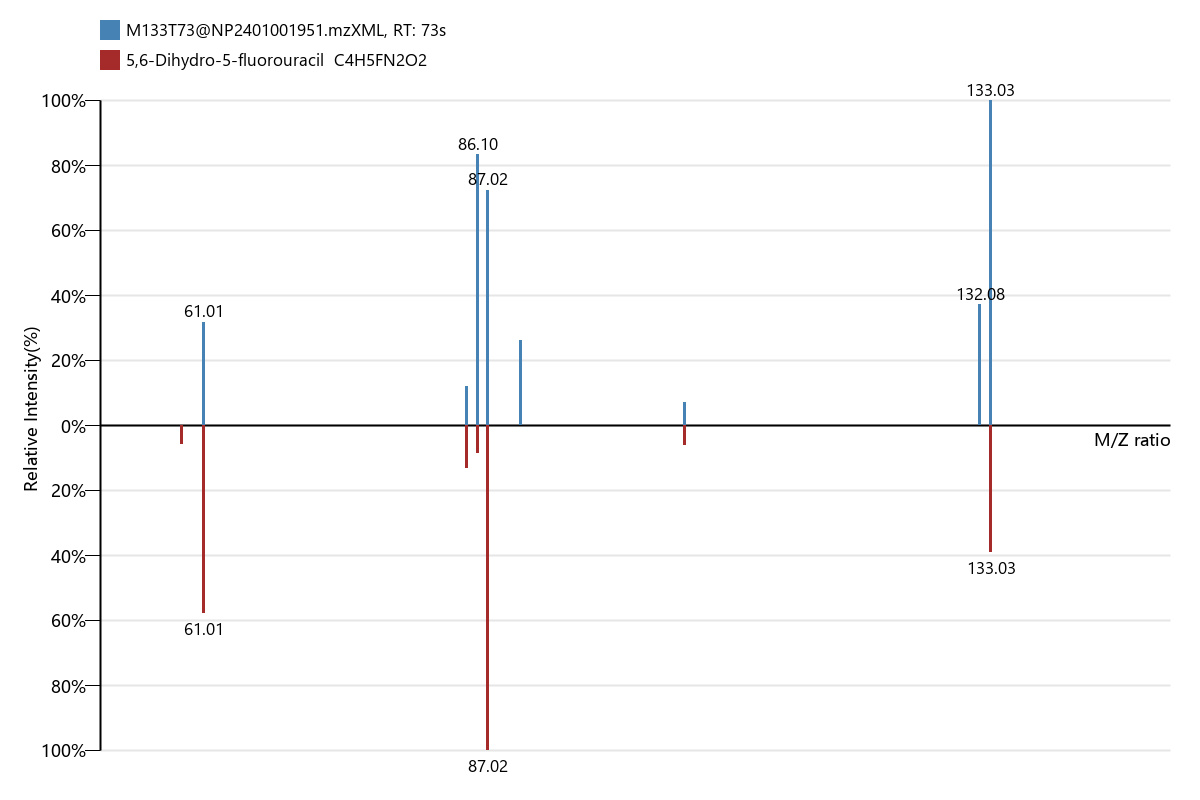

Supplement: Supplementary material 4 — Original identification chart of serum metabolites. [file Data_Sheet_5.zip › Supplementary Material S4/XJP-H and Model Group/5,6-Dihydro-5-fluorouracil.png]

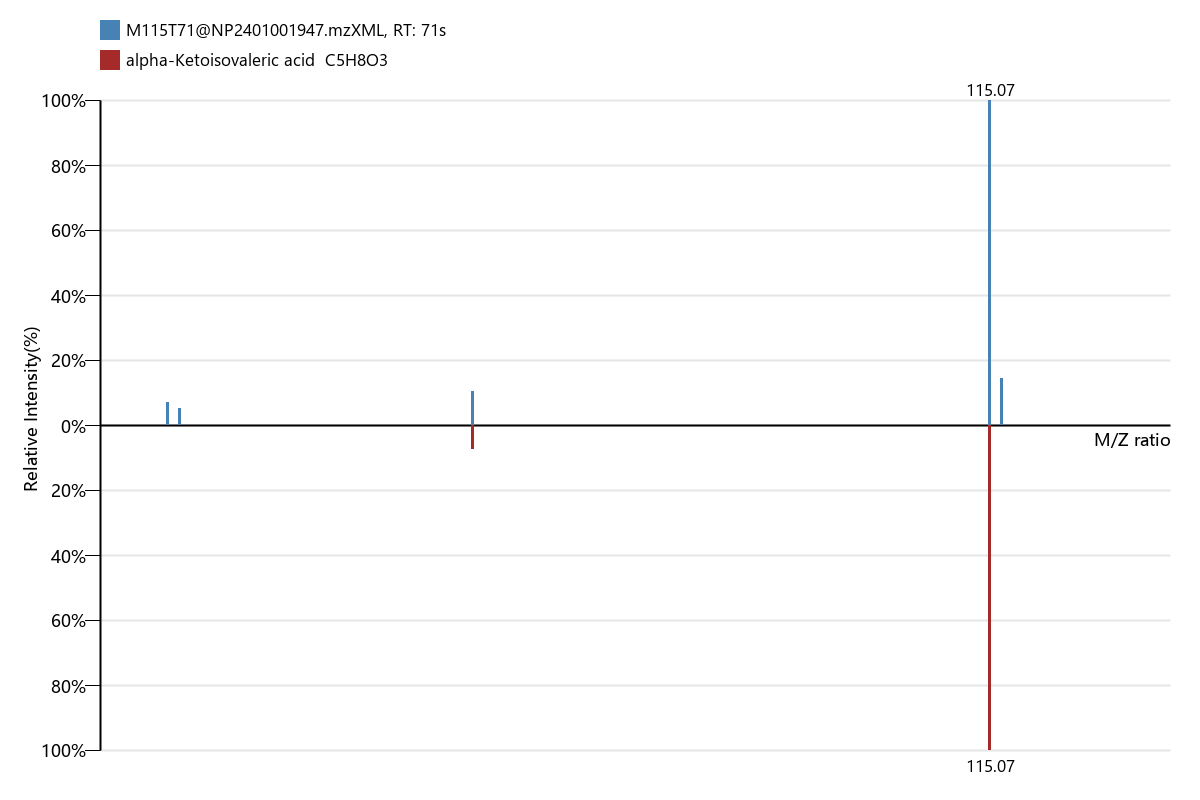

Supplement: Supplementary material 4 — Original identification chart of serum metabolites. [file Data_Sheet_5.zip › Supplementary Material S4/XJP-H and Model Group/alpha-Ketoisovaleric acid.png]

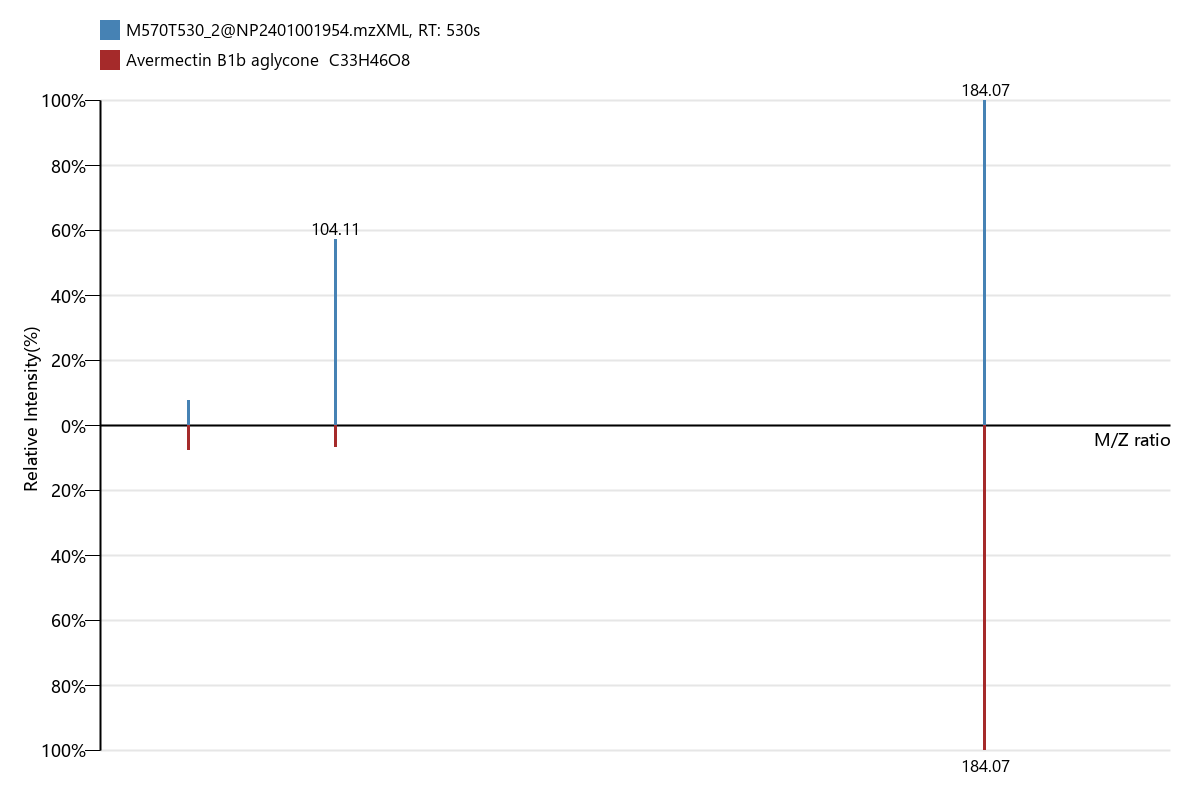

Supplement: Supplementary material 4 — Original identification chart of serum metabolites. [file Data_Sheet_5.zip › Supplementary Material S4/XJP-H and Model Group/Avermectin B1b aglycone.png]

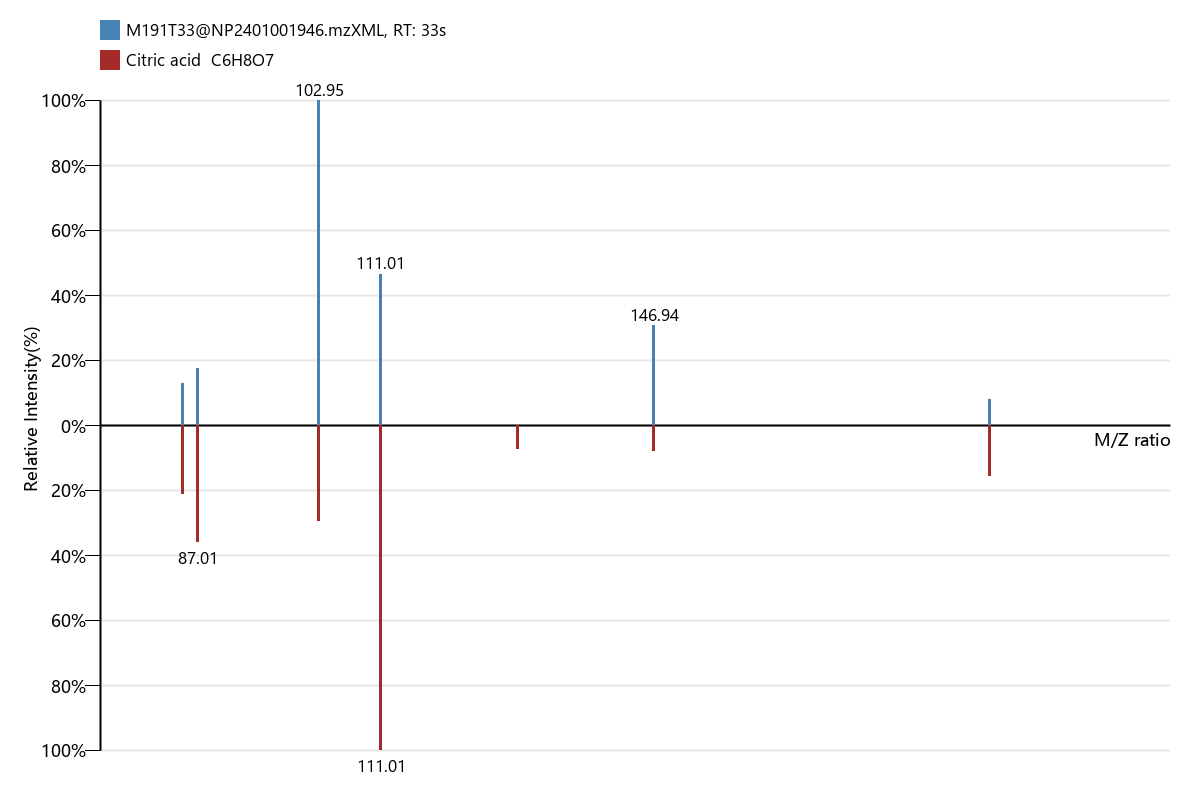

Supplement: Supplementary material 4 — Original identification chart of serum metabolites. [file Data_Sheet_5.zip › Supplementary Material S4/XJP-H and Model Group/Citric acid.png]

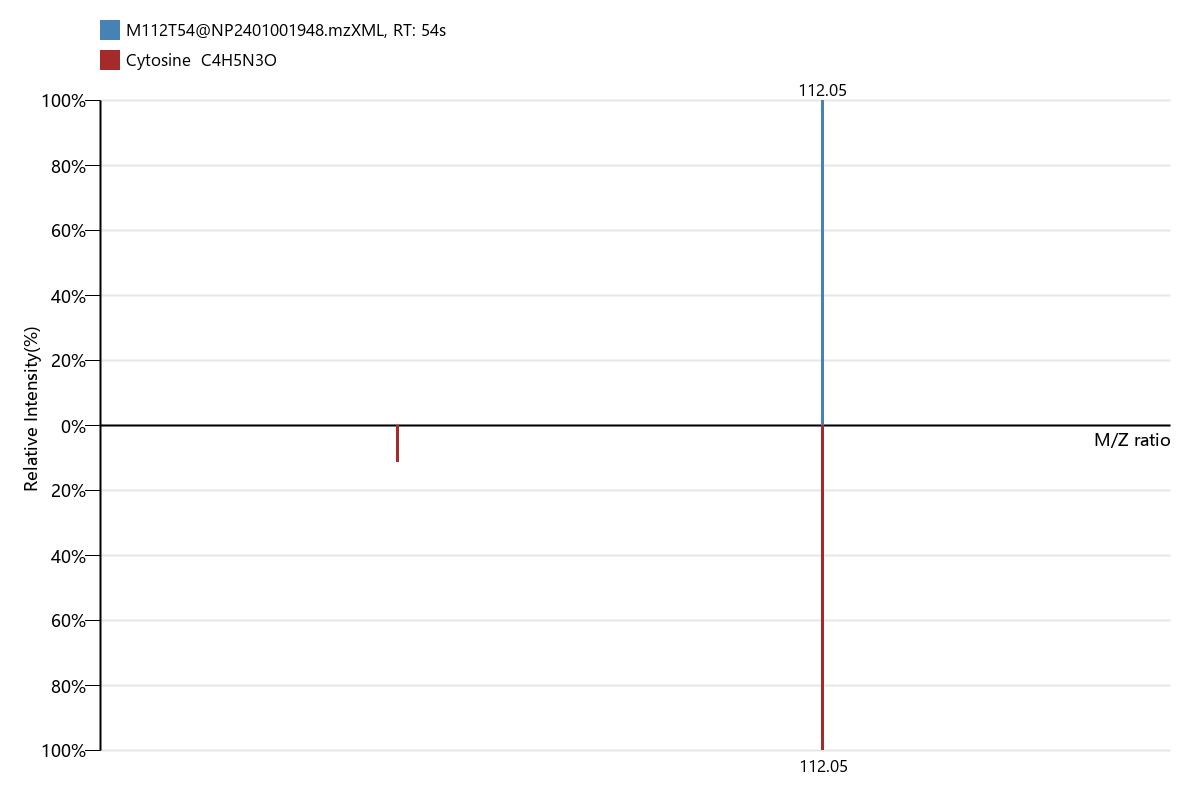

Supplement: Supplementary material 4 — Original identification chart of serum metabolites. [file Data_Sheet_5.zip › Supplementary Material S4/XJP-H and Model Group/Cytosine.png]

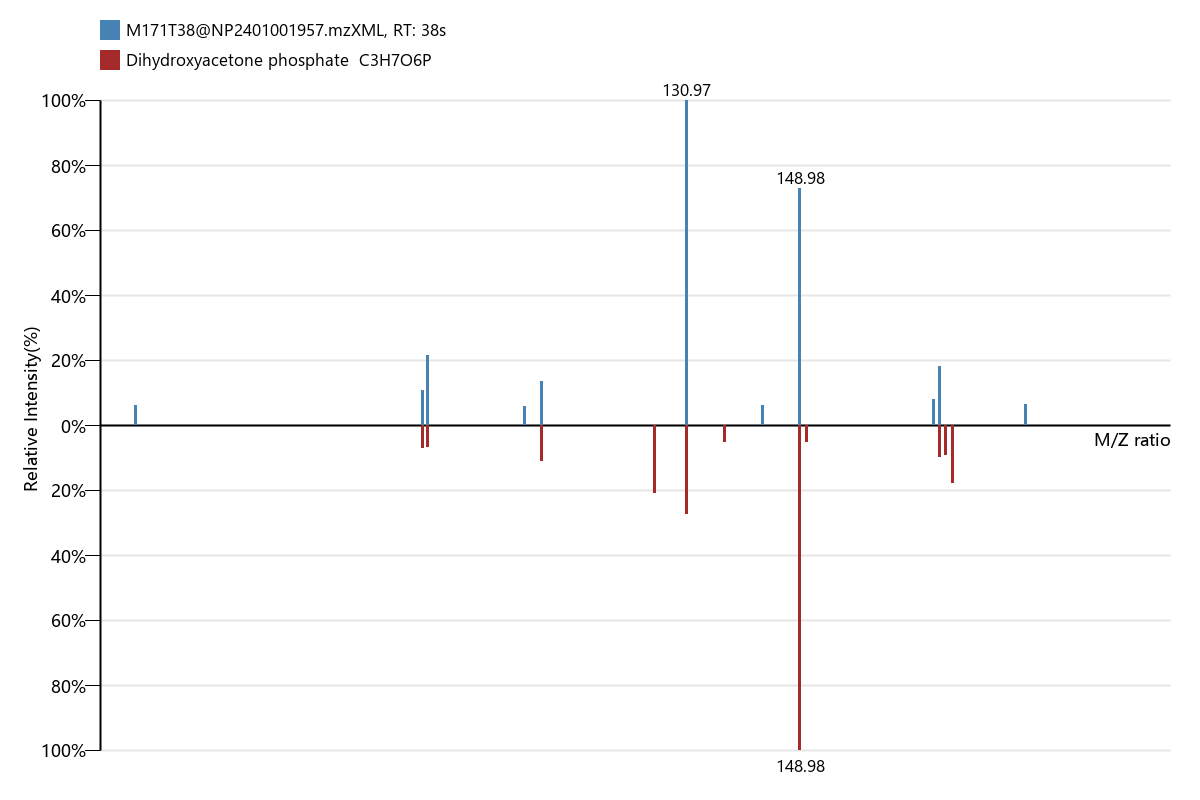

Supplement: Supplementary material 4 — Original identification chart of serum metabolites. [file Data_Sheet_5.zip › Supplementary Material S4/XJP-H and Model Group/Dihydroxyacetone phosphate.png]

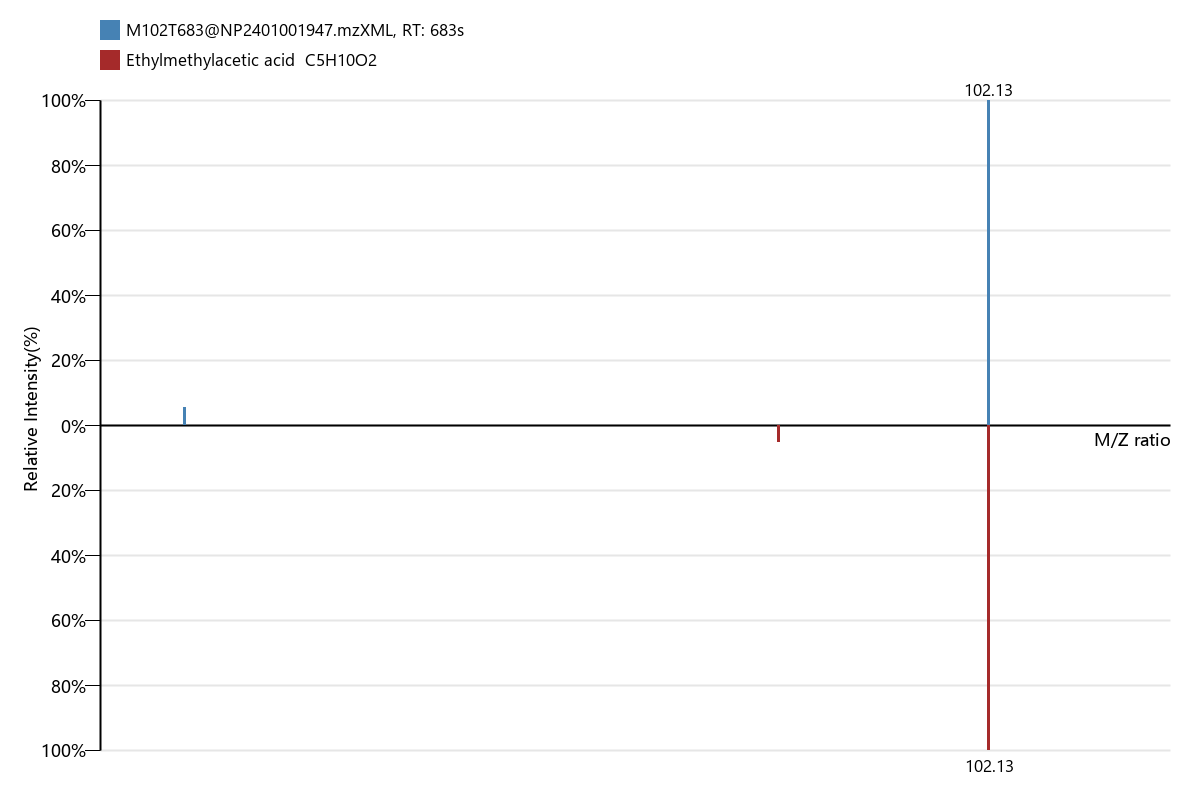

Supplement: Supplementary material 4 — Original identification chart of serum metabolites. [file Data_Sheet_5.zip › Supplementary Material S4/XJP-H and Model Group/Ethylmethylacetic acid.png]

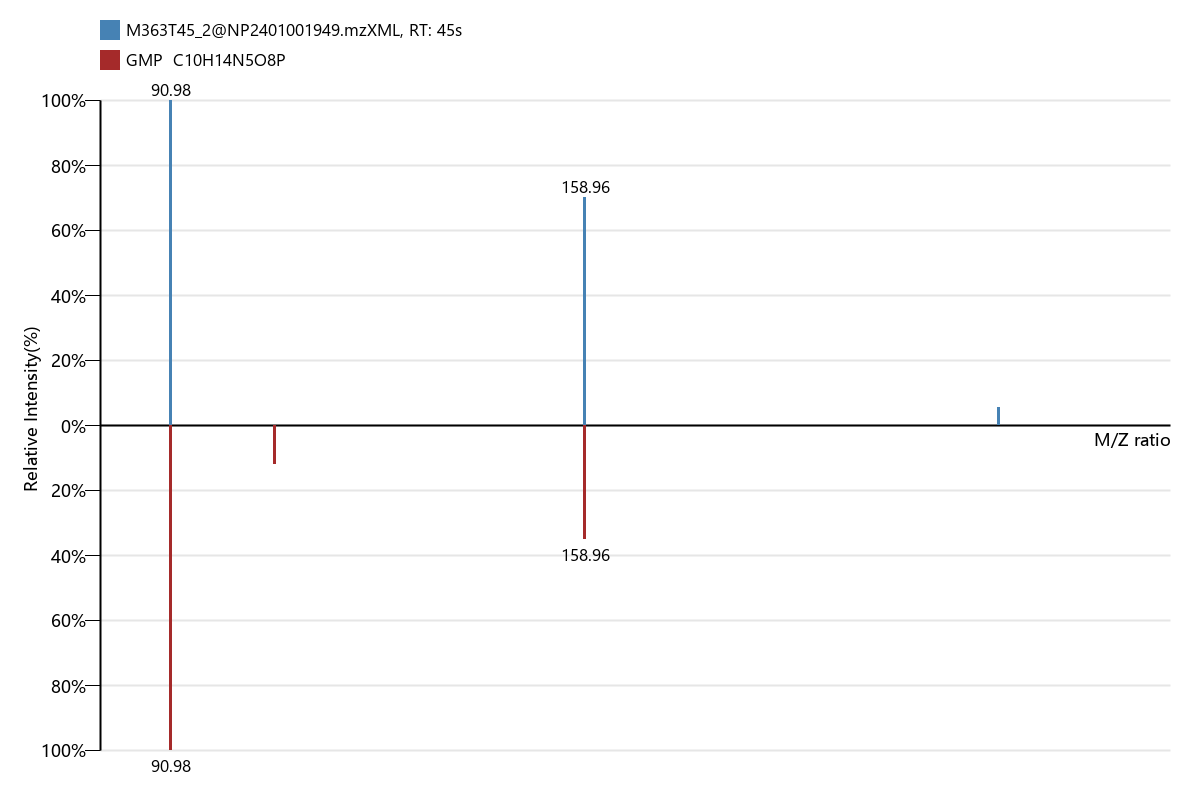

Supplement: Supplementary material 4 — Original identification chart of serum metabolites. [file Data_Sheet_5.zip › Supplementary Material S4/XJP-H and Model Group/GMP.png]

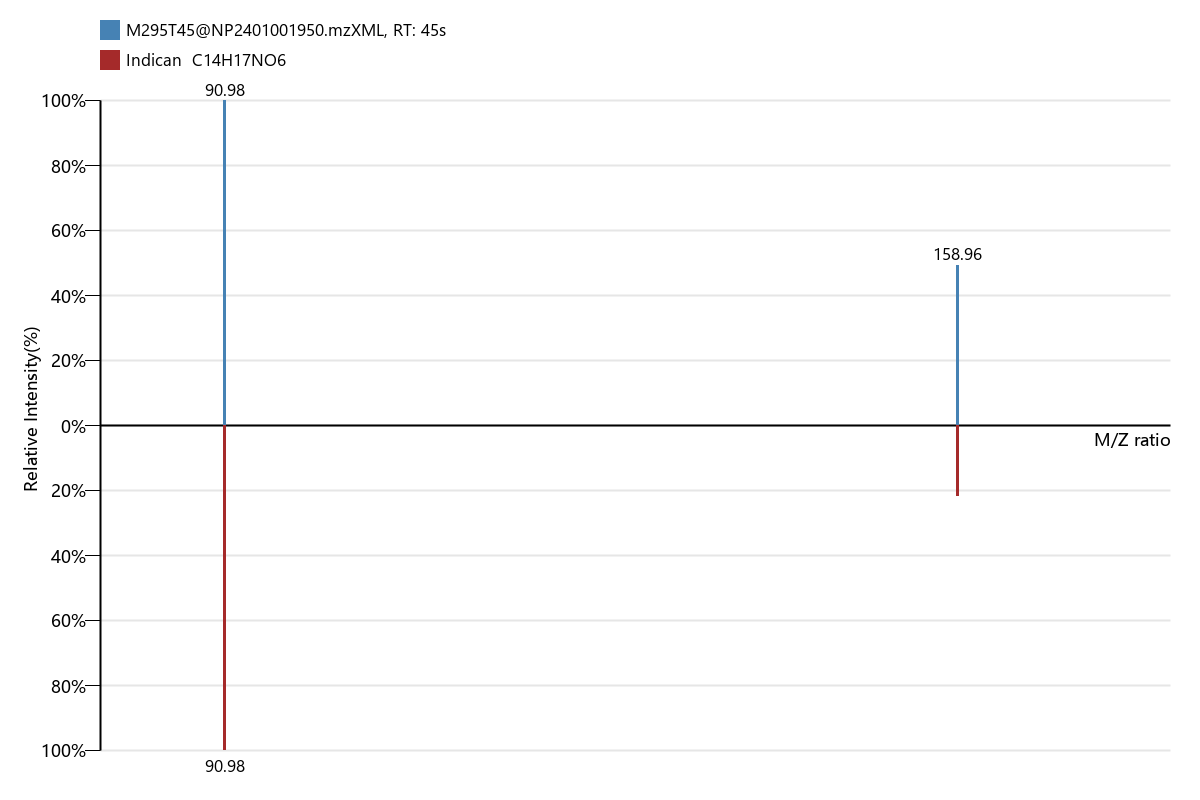

Supplement: Supplementary material 4 — Original identification chart of serum metabolites. [file Data_Sheet_5.zip › Supplementary Material S4/XJP-H and Model Group/Indican.png]

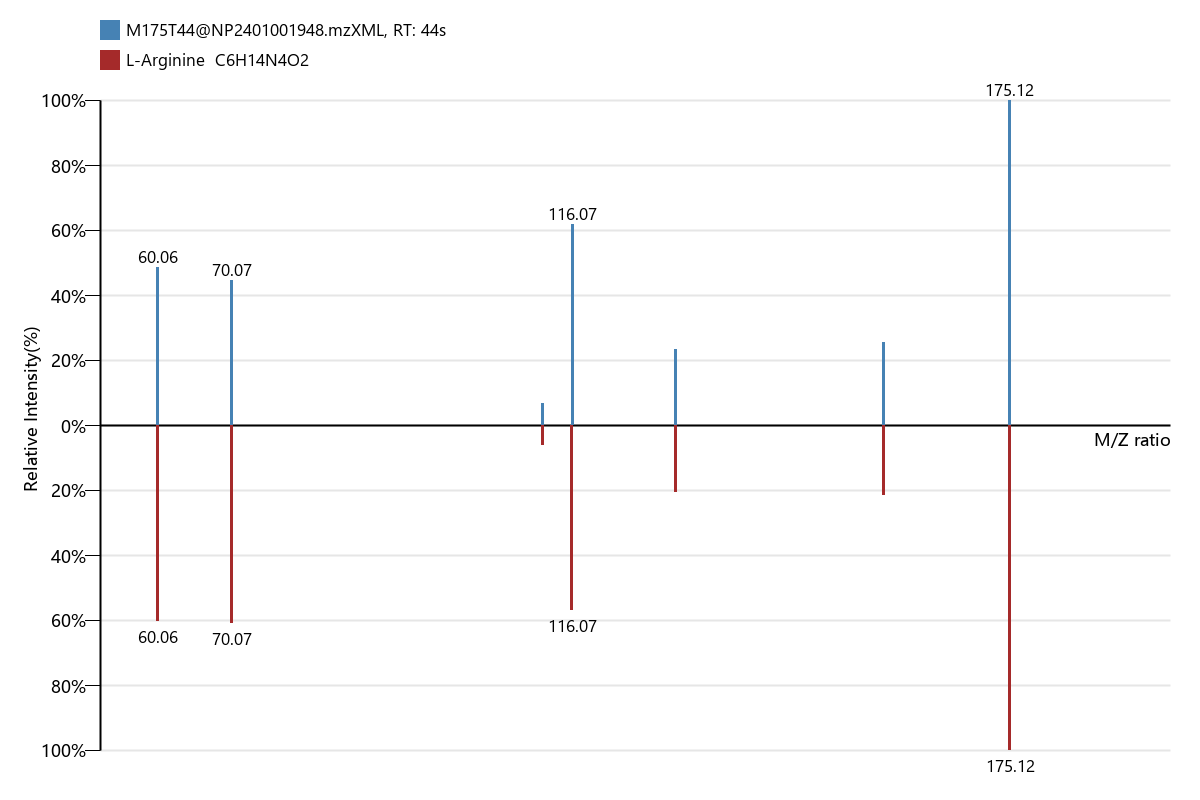

Supplement: Supplementary material 4 — Original identification chart of serum metabolites. [file Data_Sheet_5.zip › Supplementary Material S4/XJP-H and Model Group/L-Arginine.png]

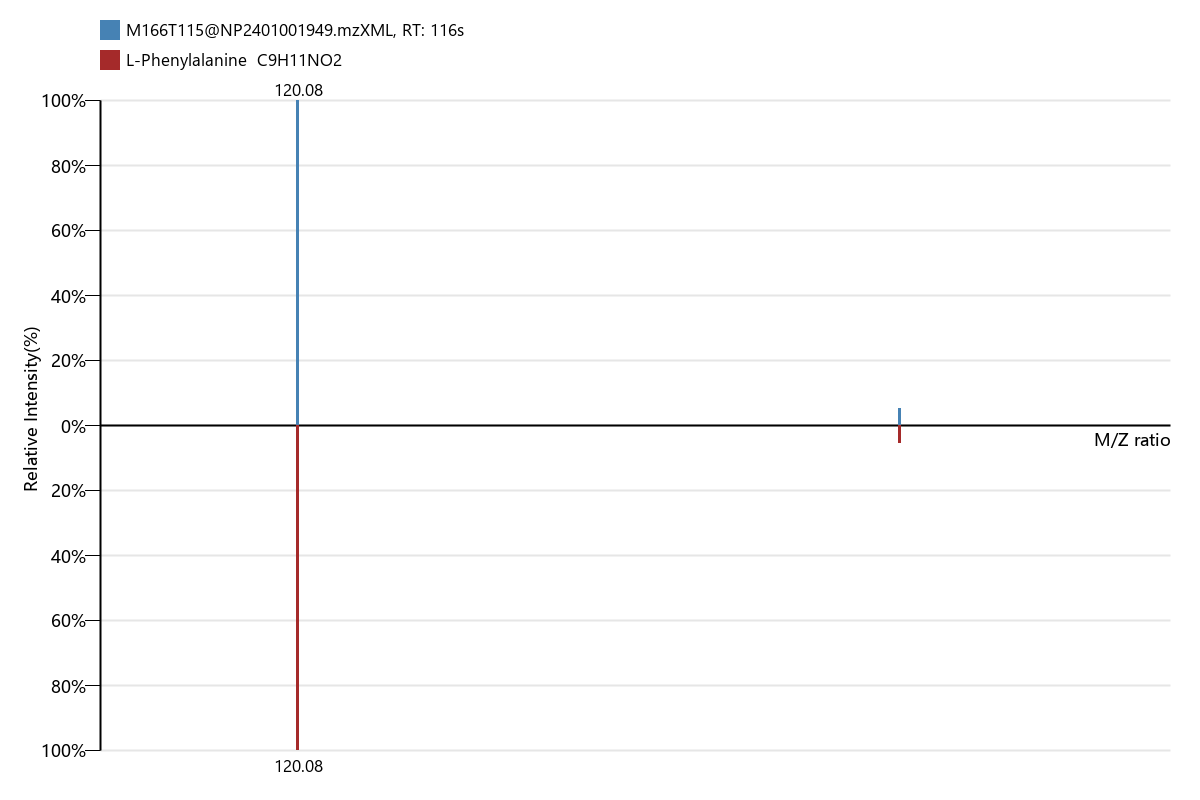

Supplement: Supplementary material 4 — Original identification chart of serum metabolites. [file Data_Sheet_5.zip › Supplementary Material S4/XJP-H and Model Group/L-Phenylalanine.png]

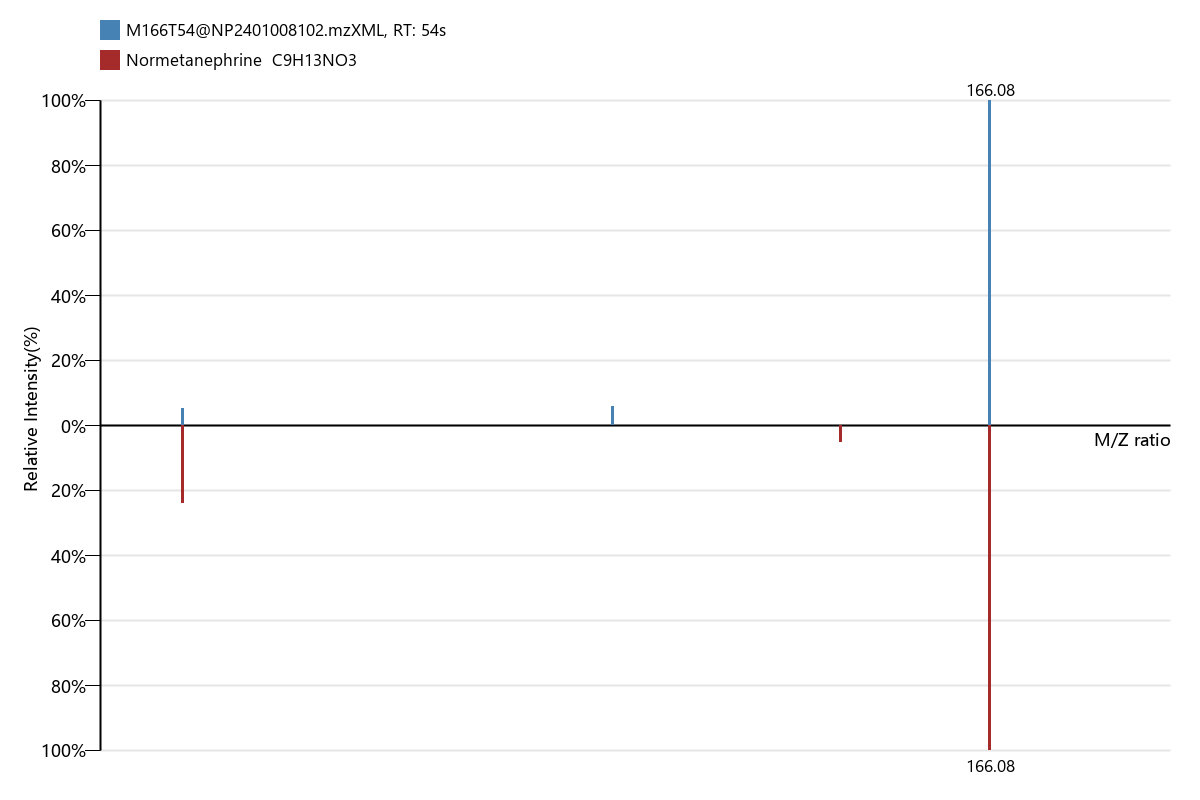

Supplement: Supplementary material 4 — Original identification chart of serum metabolites. [file Data_Sheet_5.zip › Supplementary Material S4/XJP-H and Model Group/Normetanephrine.png]

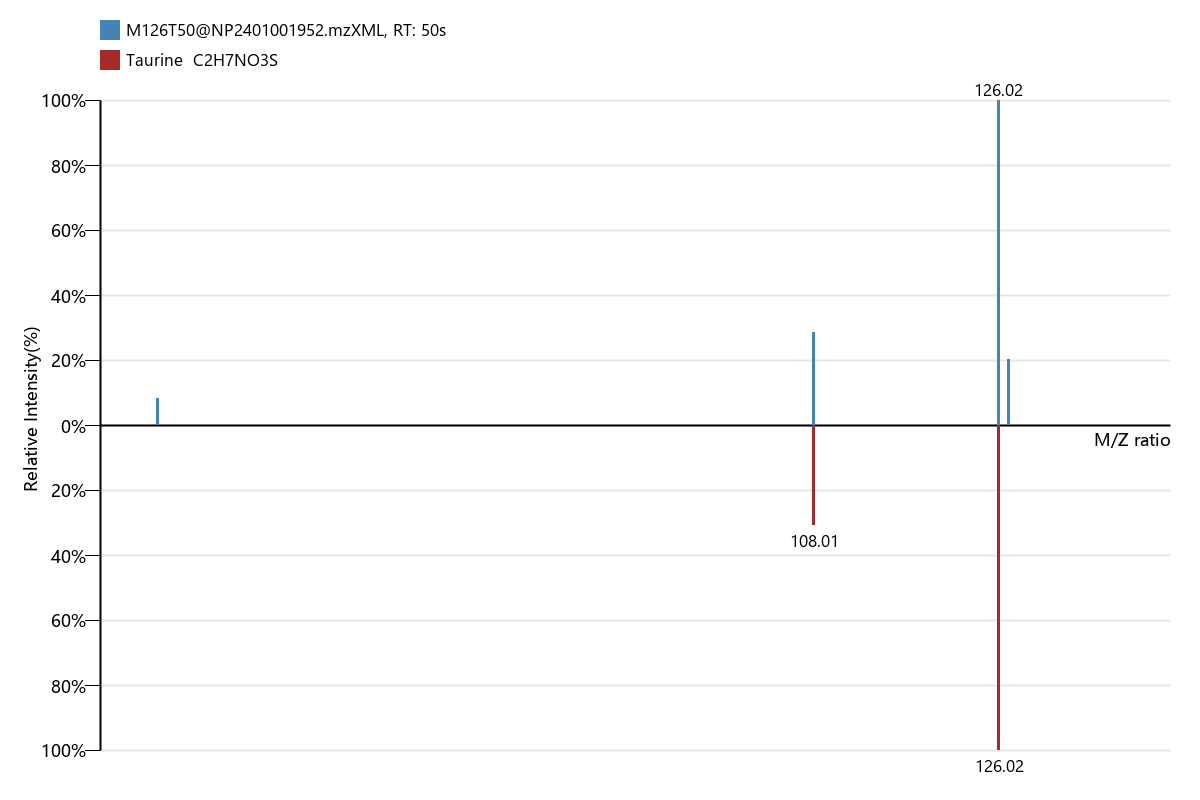

Supplement: Supplementary material 4 — Original identification chart of serum metabolites. [file Data_Sheet_5.zip › Supplementary Material S4/XJP-H and Model Group/Taurine.png]

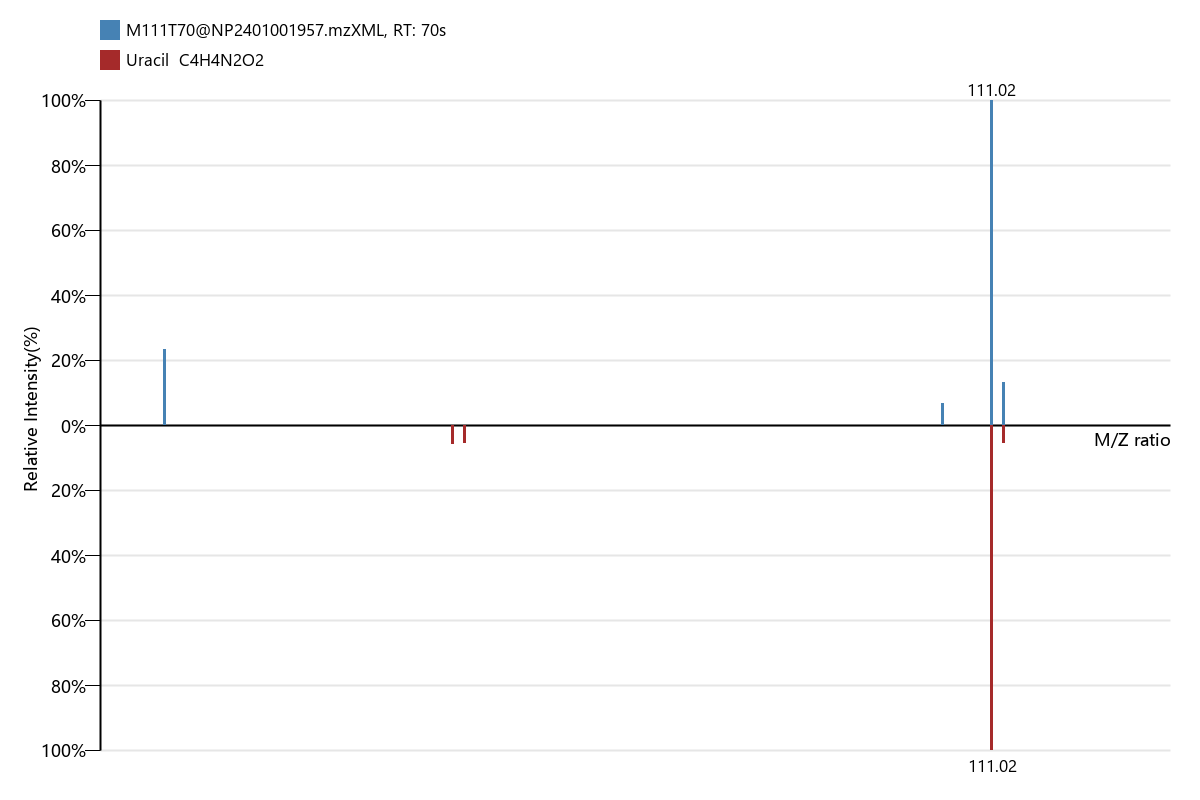

Supplement: Supplementary material 4 — Original identification chart of serum metabolites. [file Data_Sheet_5.zip › Supplementary Material S4/XJP-H and Model Group/Uracil.png]
